# Functional profiling of ovarian cancer models reveals Bcl-xL/NOTCH targeting to overcome resistance

**DOI:** 10.1038/s41698-026-01628-2

**Published:** 2026-08-04

**Authors:** Greta Gudoityte, Olena Berkovska, Lukas M. Orre, Elisabeth Moussaud-Lamodière, Rebecka Bergström, Johan Lindberg, Martin Haraldsson, Sacha Bou Nafeh, Méline Louhaur, Olli Kallioniemi, Josefin Fernebro, Ulrika Joneborg, Brinton Seashore-Ludlow

**Affiliations:** 1 Science for Life Laboratory (SciLifeLab), Karolinska Institutet, Stockholm, Sweden; 2 Department of Oncology and Pathology, Karolinska Institutet, Stockholm, Sweden; 3 Department of Clinical Science, Intervention and Technology, Karolinska Institutet, Stockholm, Sweden; 4 Department of Gynecology and Reproductive Medicine, Karolinska University Hospital, Stockholm, Sweden; 5 Department of Molecular Epidemiology and Biostatistics, Karolinska Institutet, Stockholm, Sweden; 6 Chemical Biology Consortium Sweden, Science for Life Laboratory, Department of Medical Biochemistry and Biophysics, Karolinska Institutet, Stockholm, Sweden; 7 Institute for Molecular Medicine Finland, University of Helsinki, Helsinki, Finland; 8 Department of Pelvic Cancer, Karolinska University Hospital, Stockholm, Sweden; 9 Department of Women’s and Children’s Health, Karolinska Institutet, Stockholm, Sweden

**Keywords:** Cancer, Drug discovery, Oncology

## Abstract

Platinum resistance remains a major therapeutic challenge in ovarian cancer (OC) and is one of the main causes leading to disease relapse and mortality. Although PARP inhibitors have improved outcomes for a subset of patients, most women continue to rely on platinum-taxol based chemotherapy and ultimately develop recurrent, treatment-resistant disease with limited further therapy options. Therefore, there is a critical need to identify actionable, patient-specific vulnerabilities that would help as an alternative for chemoresistant patients. To identify such therapeutic opportunities, we established 35 patient-derived models from 22 OC patients, representing seven OC subtypes and preserving key molecular features of individual patients. High-throughput drug profiling across a library of 528 oncology-focused compounds generated over 29,000 drug response measurements, revealing inter-patient heterogeneity and sensitivity patterns. Among these, a subset of models exhibited a pronounced dependency on the anti-apoptotic protein Bcl-xL with minimal effects observed on patient-derived fibroblasts and healthy bone marrow, suggesting a therapeutic window. Proteomics-based comparison of Bcl-xL-sensitive and -resistant subclones identified activation of NOTCH signaling as a determinant to reduced response to Bcl-xL inhibition. Blocking of NOTCH signaling with gamma-secretase inhibitors restored sensitivity to Bcl-xL targeting and resensitized resistant cells to Carboplatin, resulting in sustained cytotoxicity in long-term washout assays and ex vivo cultures. Similar effects were observed with both Bcl-xL protein degrader and small molecule inhibitor, supporting robust targeting of Bcl-xL through different modalities. Together, these findings define a NOTCH-modulated Bcl-xL survival axis as a therapeutic vulnerability in platinum resistant OC. More broadly, this study demonstrates how translational drug profiling of physiologically relevant disease models can generate insights with clinical potential and provide precision oncology framework for identifying rational combination strategies to overcome chemoresistance.

## Introduction

Ovarian cancer (OC) remains one of the deadliest gynecological malignancies, largely due to late-stage diagnosis and limited treatment options^1,2^. While early-stage diagnosis is often highly treatable, most patients present with advanced disease due to nonspecific symptoms and the absence of effective screening strategies^2^. The current standard-of-care treatment consists of cytoreductive surgery followed by platinum and taxol-based chemotherapy^3^. Although targeted therapies such as the VEGF inhibitor Bevacizumab, and PARP inhibitors Olaparib and Niraparib, have improved progression-free survival for subset of patients^4^, relapse occurs in around 70% of the cases, often within 18 months. Recurrent disease is commonly platinum-resistant, associated with limited therapeutic options, and poor long-term survival^5,6^, highlighting the need for new treatment strategies to overcome chemoresistance.

Although epithelial OC is often treated as a single disease, it covers a heterogenous group of malignancies with different molecular and histological features. High-grade serous (HGS) OC is the most common and aggressive epithelial OC subtype, accounting for ~75% of all cases. The remaining disease subtypes include clear cell (CC), endometrioid (EN), low-grade serous (LGS), and mucinous (MUC) OCs. HGS-OC is characterized by high copy number variation (CNV) and *TP53* mutations in over 90% of all cases^7^, with *BRCA1/2* alterations present in ~12%, contributing to homologous recombination deficiency (HRD)^8^. In contrast, other disease subtypes have distinct molecular alterations but currently lack molecularly guided treatment regimens. For instance, nearly half of LGS-OC cases (47%) harbor mutations in the RAS/RAF pathway^9^. EN-OC commonly carries mutations in *KRAS*, *PTEN*, and other genes involved in the PI3K signaling pathway. CC-OC is frequently associated with alterations in *PIK3CA* and *ARID1A*, while MUC-OC often harbors *KRAS*, *HER2* or *MYC* modifications^7,10^. These divergent molecular profiles highlight the shortcomings of current OC therapies and underscore the need for tailored strategies capable of capturing patient- and subtype-specific disease vulnerabilities.

Functional drug profiling has emerged as a promising strategy to identify actionable vulnerabilities and guide therapy selection. However, traditional cell lines (CCLs) often fail to represent primary tumor biology, partly due to long-term culture adaptation, hypermutation^11^, and inconsistent classification^12,13^. Patient-derived models (PDMs), including cancer cells (PDCs), organoids (PDOs), and ex vivo cultures, provide a more physiologically relevant alternatives by preserving genomic, proteomic, and phenotypic features of primary tumors. Such models can be linked to clinical outcomes and increasingly support precision medicine efforts across both hematological and solid malignancies^14,15^.

To advance personalized therapeutic strategies in OC, we established and systemically characterized PDMs from 22 patients spanning multiple OC subtypes. These models were then profiled against a library of 528 oncology focused and clinically relevant compounds to identify patient and subtype specific therapeutic vulnerabilities. Among the most prominent findings, inhibition of the anti-apoptotic protein Bcl-xL demonstrated selective and sustained activity, in both Carboplatin-sensitive and -resistant models. Analysis of genomically similar, Carboplatin-resistant subclones revealed differential sensitivity to Bcl-xL inhibition, suggesting alternative resistance mechanisms. Further deep proteomics profiling revealed NOTCH signaling activation in Bcl-xL resistant model, which guided a rational combination strategy targeting NOTCH signaling (via γ-secretase) and Bcl-xL. This combination restored sensitivity to Carboplatin and resulted in synergistic cytotoxicity, minimal effects in the fibroblasts, and durable efficacy in long-term spheroids, as well as ex vivo patient assays. In summary, this work proposes a rational combination strategy with the potentially resensitize cancer cells and highlight the power of PDMs to guide novel therapies.

## Results

### Patient-derived models recapitulate unique patient-dependent genetic profiles

**Fig. 1:**
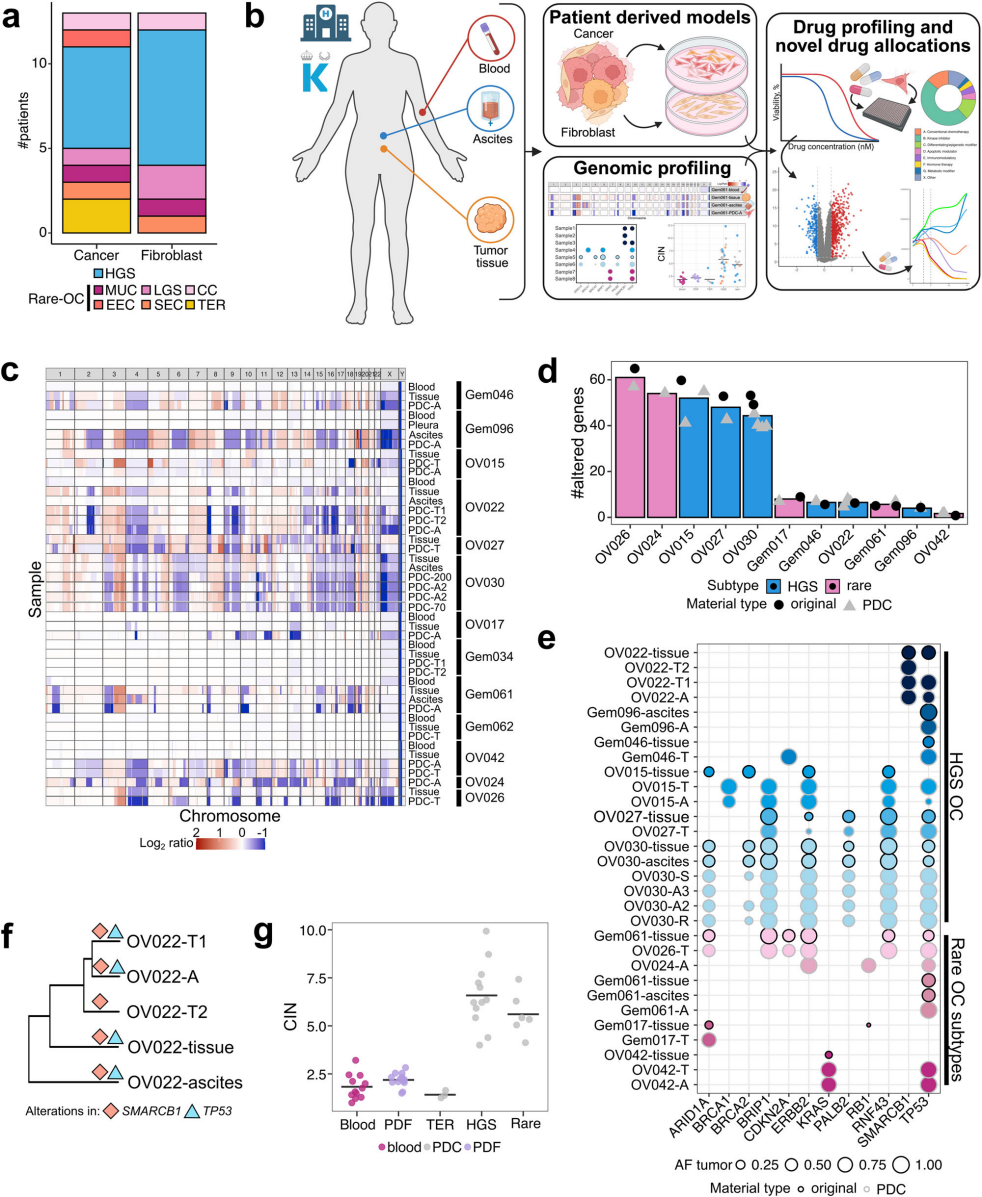
Patient-derived models (PDMs) recapitulate patient-specific genetic profiles. **a** Summary of the established PDM collection, each color represents different OC subtype. **b** Schematic overview of the patient sample collection and experimental workflow used in this study (created with Biorender.com). **c** CNV profile of original patient tumor tissue/blood/ascites material and the PDCs used in this study. **d** Comparison of the number of genes with high or moderate impact mutations between original patient material and their matched PDMs. **e** Overview of mutations in key OC-associated genes across all PDMs and their matched original material. **f** Phylogenic tree illustrating the genetic relationship between original OV022 patient samples (tissue and ascites) and three PDCs. The tree was constructed using somatic variants present at ≥0.5 allele fraction focusing on genes with high and modifier predicted impact. This representation highlights the clonal evolution and divergence between the different models and material. **g** Distribution of chromosomal instability index (CIN) in PDMs and blood samples from corresponding patients. Abbreviations used in the figures: HGS—high-grade serous OC, MUC—mucinous OC, LGS—low-grade serous OC, CC—clear cell OC, EEC—endometrioid OC, SEC—serous endometrial OC, TER—ovarian teratoma, PDC—patient-derived cancer cells, PDF—patient-derived fibroblasts, CIN—chromosomal instability index, T and A in the annotations represent tumor tissue and ascites location respectively.

To enable translational drug profiling, well-characterized disease models are critical. Yet the lack of physiologically relevant models continues to hinder progress identifying effective therapies in OC. To address this gap, we established a collection of PDMs from 22 patients, generated from freshly resected OC tumor tissue or ascites aspirates, representing both cancer (PDC) and fibroblast (PDF) models (Supplementary Table 1, Fig. 1a). All models were characterized by genomic profiling of patient-specific gene mutations and genomic instabilities. Additionally, drug sensitivity and resistance profiling was performed to determine individual therapeutic response patterns. This comprehensive approach enabled the identification of new drug combinations with potential efficacy in OC as detailed further in this paper (Fig. 1b). The most represented disease subtype in our collection is HGS-OC, where we have 11 models derived from six different patients. Our collection also includes PDCs from rare OC subtypes, such as LGS, serous endometrial (SEC), CC, EEC, and MUC. In addition, we generated two models from two patients with teratoma of the ovary (TER), representing both growing teratoma syndrome and immature teratoma. Finally, our collection contains non-cancerous models representing 14 PDFs from 13 patients. In line with observed clinical outcomes in OC, 73% of patients in this cohort, experienced disease relapse with an average time to progression of 8 months. The remaining 27% of patients in this cohort had no relapse or a favorable response to the administered treatment. Thus, our PDC and PDF model collection represents a spectrum of clinical outcomes, including both aggressive and favorable responders. The detailed clinical characteristics of each patient are presented in Supplementary Table 2.

Targeted genome sequencing was performed to first compare PDMs with their matched primary tissue or ascites samples and peripheral blood. Overall, PDCs exhibited matching CNV profiles with their corresponding original tissue or ascites samples (Fig. 1c). As expected, PDFs did not exhibit tumor-associated genomic alterations (Supplementary Fig. 1b). Mutation analysis further confirmed a great concordance between the original samples and their PDCs, based on the number of genes harboring mutations (Fig. 1d). Among these, mutations in *TP53*, whose aberrations are observed in over 90% of all HGS-OC cases^16^, were detected in 5 out of 6 patients within our HGS-OC model collection. Interestingly, the OV015 model’s mutation in *TP53* was not detected in the original material. Additionally, alterations in *BRCA1/2* genes, typically found in ~14% of HGS-OC cases^17^, were identified in two HGS-OC cancer patients. The OV030 models consistently retained a *BRCA2* mutation, whereas OV015 PDCs carried a *BRCA1* mutation, while the original tumor sample harbored a *BRCA2* alteration. This highlights the heterogeneity of advanced stage disease. Interestingly, our data also revealed recurrent mutations in *BRIP1* (3/6) and *PALB2* (2/6), both of which encode proteins interacting with BRCA1/2 and essential for homologous recombination (HR)^18,19^. While the roles of *BRIP1* and *PALB2* mutations are not as strongly linked to OC as *BRCA1/2*, cases exist where patients with these mutations were sensitive to PARP inhibitors^20^. Alterations in these genes frequently result in HR deficiency (HRD)^21^, which is a well-known predictor of response to PARP inhibitors. Interestingly, *TP53* mutations were also observed in models derived from rare OC subtypes, despite their lower prevalence in these tumor types. The CC-OC model Gem017-T did not exhibit a *TP53* mutation, but instead carried alterations in *ARID1A* gene, which are found in over 50% of CC-OC cases and are considered a subtype-specific hallmark^22^. Moreover, a known *KRAS* hotspot mutation (Gly13Asp) was identified in the MUC-OC from the OV042 patient, albeit with a higher allelic frequency in PDC, indicating potential clonal enrichment in vitro (Fig. 1e). Interestingly, the three PDC models derived from OV022 share a *SMARCB1* mutation, while only two of the models have alterations in *TP53*, OV022-T1 and OV022-A (Fig. 1f). Mutations in *SMARCB1* are more commonly associated with pediatric and rare adult cancers including such as small cell carcinoma of the ovary, hypercalcemic type (SCCOHT)^23,24^. This demonstrates the intra-patient heterogeneity in HGS-OC and suggests that *SMARCB1* may represent a key driver mutation in this patient, potentially contributing to tumor progression in the absence of *TP53* alterations.

CNV analysis of the PDMs further demonstrated that HGS-OC models exhibited significantly greater genomic instability compared to rare OC subtypes, as indicated by a higher chromosome instability index (CIN) score (Fig. 1g). However, CIN varied between individual patients (Supplementary Fig. 1c), reflecting the heterogeneity of genomic instability in OC. As expected, PDFs showed low CIN, supporting their non-cancerous genotype. Similarly, TER models exhibited minimal chromosomal alterations in line with previous reports^25^ (Fig. 1g). Among frequently altered genes, *CCNE1* and *PIK3CA* were commonly amplified across samples, while deletions in *CDKN2A* and *CDKN2B* were observed (Supplementary Fig. 1d). In addition, similarly to the retained mutational profiles in PDCs, most PDCs retained a high degree of concordance in CNV profiles compared to the corresponding original material (Supplementary Fig. 1e). Overall, our findings suggest that the generated PDMs recapitulate patient-specific genome alterations and capture the molecular diversity across a broad spectrum of OC subtypes.

### Drug sensitivity testing identifies cancer cell selective drug activity

**Fig. 2:**
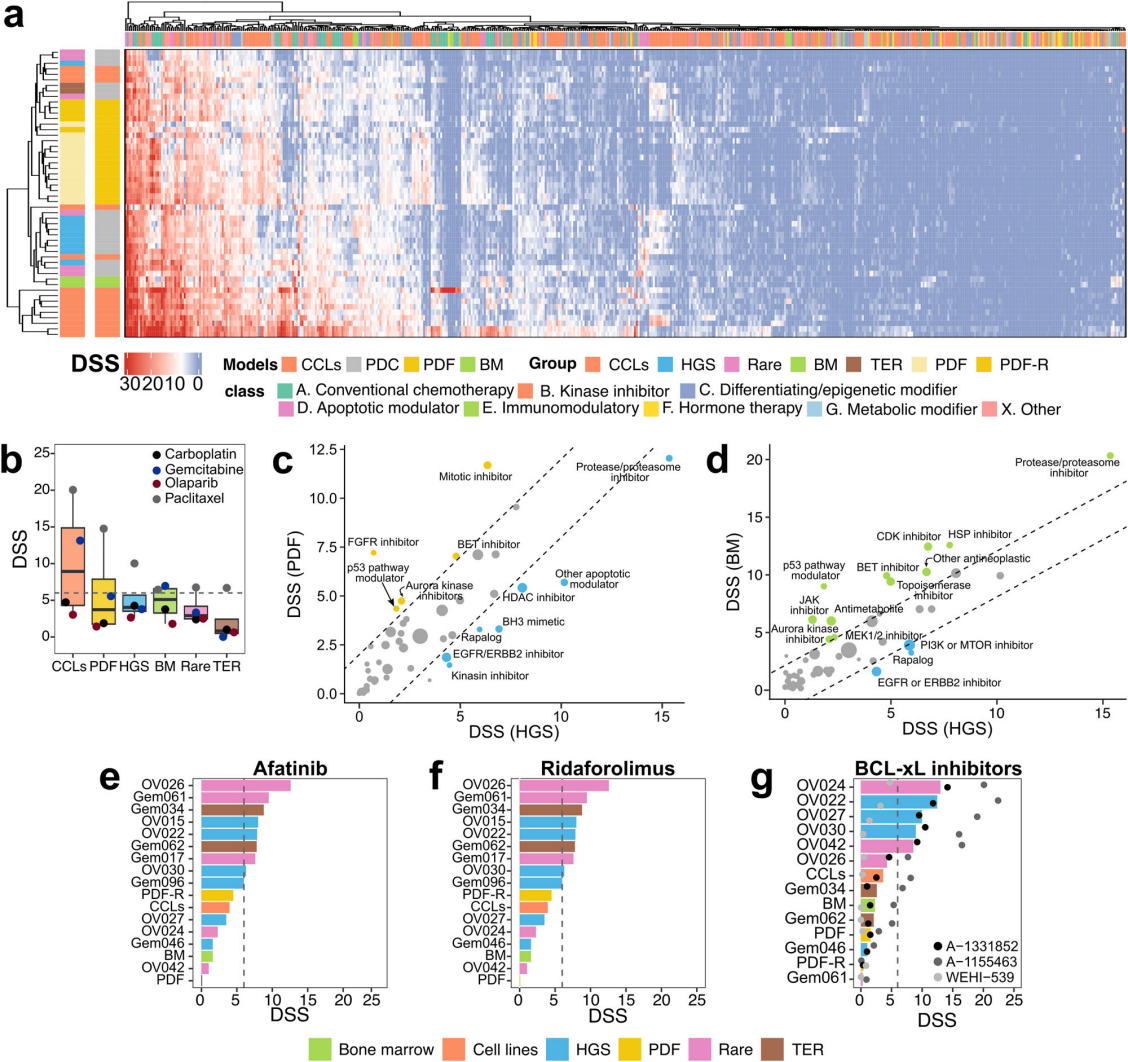
Drug sensitivity profiling uncovers selective activity in cancer cells. **a** Hierarchical clustering of all drug screened samples by DSS, drug classes are indicated with colors on the top, cell models with respective color code indicated on the left hand-side. **b** Boxplot of responses to clinically relevant drugs, with each dot representing an individual drug, colored accordingly. Dashed line marks DSS = 6. Comparison of DSS between HGS and PDF (**c**) and bone marrow (**d**). Each dot represents MoA, dot size indicated the number of compounds in the subclass. Dashed lines denote sDSS±2. DSS values for representative drugs of different subclass across patient samples: Afatinib—**e**, Ridaforolimus—**f**, Bcl-xL inhibitors—**g**. Each dot represents the average DSS per patient per drugs, dashed line indicates DSS = 6. Abbreviations used in the figures: DSS—drug sensitivity score, CCLs—cell lines, PDC—patient-derived cancer cells, PDF—patient-derived fibroblasts, HGS—high-grade serous OC, TER—ovarian teratoma, Rare—rare OC subtypes.

To further characterize the functional taxonomy of OC, we performed drug sensitivity testing (DST) with a panel of up to 528 approved and investigational drugs^26^, supporting drug repurposing efforts and providing a patient-specific drug sensitivity profile. Similar drug libraries have previously been successfully applied to study both hematological malignancies and solid tumors using primary material^27,28^. To compare the drug responses of PDCs with existing OC models, we included 15 OC CCLs. Additionally, in order to rule out general toxicity of the drugs, two healthy bone marrow samples were used as surrogate of systemic toxicity, enabling an approximation of therapeutic efficacy window for each drug (Fig. 2a). Drug sensitivity was determined using drug sensitivity score (DSS), with DSS > 6 considered indicative of moderate cellular killing (75th percentile, Supplementary Fig. 2a). Among the screened models, CCLs exhibited the highest mean sensitivity to the tested drugs with an average DSS of 5.55, in contrast, both PDCs and PDFs exhibited lower sensitivity (mean DSS = 3.53) (Supplementary Fig. 2b). Average DSS values were relatively low, largely because 66.41% of all drugs did not show any significant effect on cell viability (DSS≤6, sd>4). Among the 528 drugs tested, 304 exhibited minimal or no toxicity towards healthy bone marrow samples (DSS≤4 ± 2). However, 88.74% of these compounds also did not exhibit cytotoxic effects in PDMs or CCLs, limiting their therapeutic relevance in this disease context. In contrast, 38 out of 528 drugs displayed high toxicity in bone marrow (DSS≥15), suggesting them as poor candidates for repurposing. Minimal toxicity in bone marrow was observed in immunomodulatory and hormone therapies, whereas several classes of compounds, such as protease/proteasome inhibitors, exhibited potent cytotoxicity, with the latter showing the highest killing effects (Supplementary Fig. 2c). Global drug response analysis revealed that all PDCs clustered closely with OC CCLs, while PDFs formed a distinct cluster (Supplementary Fig. 2d, Supplementry Table 3). The partially distinct clustering pattern of CCLs, together with their overall increased sensitivity, suggests that they may represent a biologically distinct entity. Within this group, KURAMOCHI, OVSAHO, OVCAR4, OVCAR3 displayed drug response profiles most similar to the PDCs. Consistent with these observations, these cell lines have been reported as the most representative models of HGS-OC^11^. In contrast, although EFO21 had a similar profile to PDCs, its drug response profile is more likely representative of CC-OC^12^. This highlights the importance of PDMs as a validated disease model, connected to patient clinical profiles and outcomes, providing an alternative to traditional CCLs. In addition, differences in drug responses between distinct disease compartments might also arise from known biological differences between ascites as tumor tissue^29^. Comparison between the 12 PDCs derived from tumor tissue and 8 PDCs originating from ascites did not show any significant drug sensitivity differences based on DSS comparison (*p* = 0.7762, Supplementary Fig. 2e-g). However, among all drug classes tumor tissue showed higher sensitivity to the chemotherapeutic drugs (average DSS = 6.14) compared to the ones derived from ascites (average DSS = 4.24) (Supplementary Fig. 2h). Significant differences were not observed among individual drugs (Supplementary Table 4). Similar results have been observed in our previous study on short-term cultures^30^. While our data do not show compelling differences, this may also be impacted due to limited sample size. In addition, investigating differences in drug profiles between PDCs and PDFs we observed that the different media compositions used to maintain PDCs and PDFs induced systematic differences in drug responses. PDFs, cultured in Fibroblast media supplemented with fibroblast growth factor (FGF), exhibited increased sensitivity to FGFR inhibitors, whereas those (PDF-R) maintained in Rockit media containing epidermal growth factor (EGF) were more responsive to EGFR/ERBB2 inhibitors (Supplementary Fig. 2i). There was no significant difference in PDF proliferation in the different culture conditions (Supplementary Fig. 2j). Beyond media-related effects, PDMs from patient OV022 displayed unique drug sensitivity profiles. The two tissue-derived PDMs, OV022-T1 and OV022-T2, harboring mutations in both *TP53* and *SMARCB1* or only *SMARCB1*, respectively, exhibited similar overall drug sensitivity. In contrast, OV022-A, derived from ascites and carrying both the *TP53* and *SMARCB1* mutations similar OV022-T1 showed significantly greater drug sensitivity (*p* = 0.014) (Supplementary Fig. 2k). This case highlights the importance of integrating both genetic and functional profiling, as tumor site may influence therapeutic response and uncover otherwise overlooked treatment responses and therapeutic opportunities.

Standard-of-care treatments (Carboplatin, Paclitaxel, Olaparib, and Gemcitabine) were included in our drug library. In contrast to PDMs, CCLs showed a greater sensitivity to these drugs, in particular to Carboplatin and Gemcitabine (Fig. 2b). These drugs demonstrated higher toxicity in bone marrow and PDFs. Furthermore, PARP inhibitors did not show strong cytotoxic effects in our models, despite the presence of *BRCA1/2* mutations in several patients; however, it is important to mention that PARP inhibitor response is not determined by *BRCA1/2* mutations alone and that PARP inhibitors are approved in the maintenance setting for these patients^31^. The limited response observed in PDMs to standard-of-care drugs may reflect the patient clinical response, since the majority of patients experienced relapse within 8 months (11/13).

To further identify compounds with cancer cell-specific activity, we compared drug responses between the HGS-OC group and non-malignant controls (PDFs and BMs) (Fig. 2c-d). HGS-OC models were selected for this purpose since we had sufficient numbers for comparison. Certain drug subclasses, including BET, aurora kinase inhibitors, and p53 pathway modulators, displayed high toxicity across both bone marrow and PDFs, suggesting limited therapeutic selectivity for OC. Among all drugs, HGS-OC models demonstrated increased response to EGFR/ERBB2 inhibitors and rapalogs. Notably, these drugs produce minimal effects on bone marrow and PDFs. For example, the EGFR inhibitor Afatinib demonstrated low cytotoxicity towards bone marrow (DSS = 1.6) and PDFs (mean DSS = 2.3) but significant killing effect (DSS > 6) in 8/12 PDCs (Fig. 2e). Interestingly, both TER patients showed high response to this drug (Supplementary Fig. 2l). Similarly, the rapalog, Ridaforolimus, displayed selectivity towards cancer cells in 7/12 patients (Fig. 2f), however overall, the highest cytotoxic effects were detected among CCLs (Supplementary Fig. 2m). Interestingly, BH3 mimetics showed a good response in several PDMs and the Bcl-xL inhibitors (A-1331852, A-1155463, WEHI-539) exhibited higher cytotoxicity in PDCs, while maintaining low toxicity in bone marrow (DSS = 3.4) or PDFs (DSS = 1.7). These drugs had strong cytotoxic activity in HGS-OC models (DSS = 13.4) and rare epithelial OC subtypes (DSS = 10.4), while only TER showed low response (DSS = 3.5) (Fig. 2g, Supplementary Fig. 2n). Other BH3 mimetics targeting BCL-2 or MCL-1, such as Navitoclax and Venetoclax, commonly used in treatment of hematological malignancies^32^, showed higher toxicity effects in bone marrow (DSS > 6) and lower activity in PDMs or CCLs (Supplementary Fig. 2o). This suggests a specific Bcl-xL dependence in these patients. This is in line with previous studies identifying Bcl-xL as a therapeutic target in OC^33,34^. It is important to mention that PDMs enabled the identification of drugs (such as Afatinib and Bcl-xL inhibitors), which otherwise showed limited efficacy CCL models. Altogether, these findings highlight the challenges of OC treatment, revealing both the limitations of conventional chemotherapy and the challenges in the identification of targeted therapies. Nevertheless, our data identifies several promising avenues for novel targeted treatments and emphasizes the importance of patient-tailored treatment approaches. Furthermore, these results highlight the value of PDMs as a more predictive and clinically relevant platform for pre-clinical drug research compared to traditional CCLs models.

### Inter and intra-patient response differences to Bcl-xL inhibitors

**Fig. 3:**
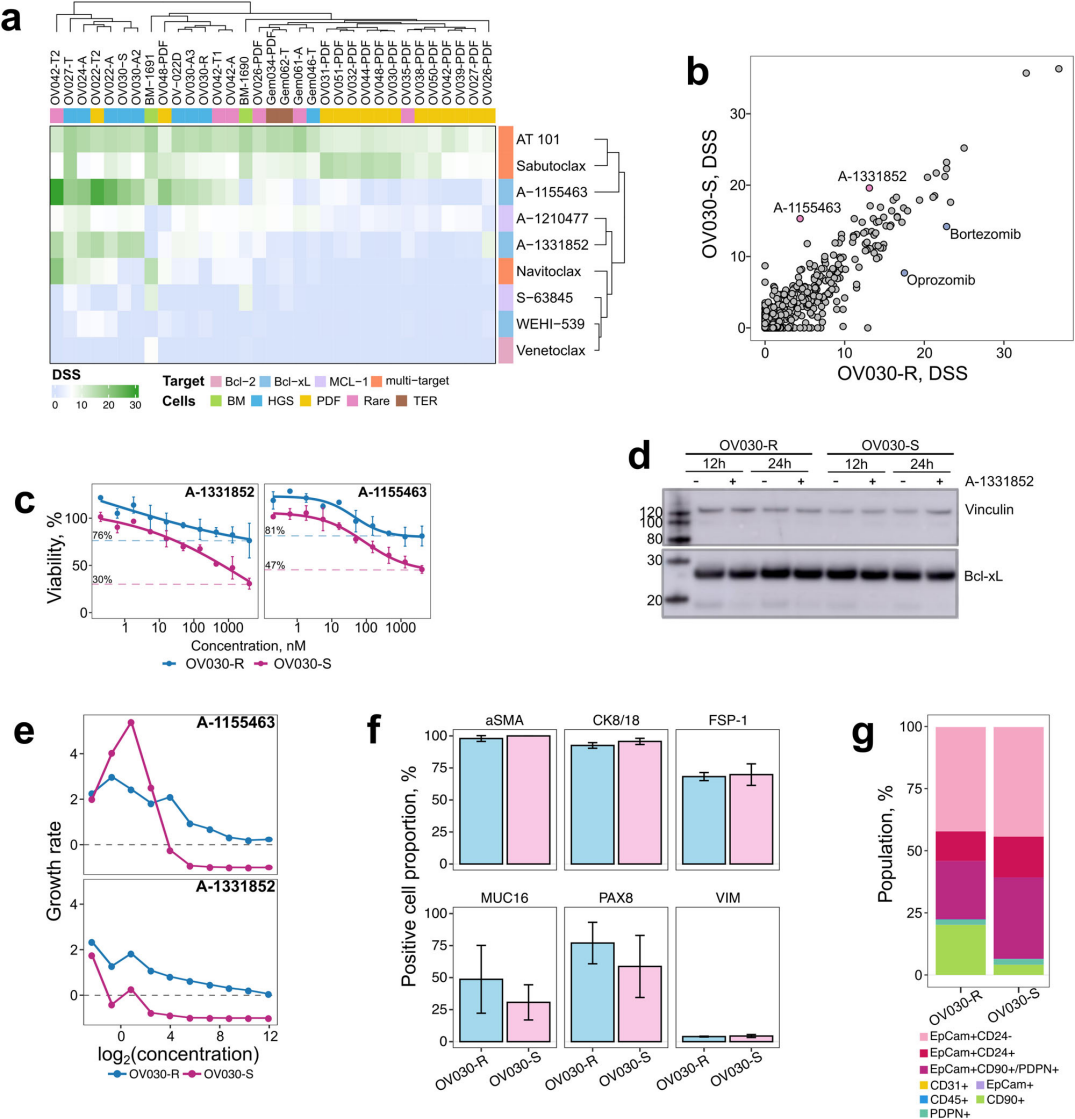
Heterogenous responses to Bcl-xL inhibitors across patients. **a** Heatmap showing DSS to BH3 mimetics across all PDMs. **b** DSS comparison between OV030-R and OV030-S clones, highlighted drugs show those with the greatest differential sensitivity. **c** Dose response curves for A-1331852 and A-1155463 in all OV030 patient clones, error bars indicate mean ± SD. **d** Representative Western Blot showing Bcl-xL protein levels in OV030-R and OV030-S clones, with and without A-1331852 treatment. On left side molecular weight marker (Magic Marker) sizes are indicated in kDa. **e** Growth rate (GR) corrected drug response of OV030-R and OV030-S clones to two Bcl-xL inhibitors. Dashed lines indicate GR = 0. **f** Bar plots showing the percentage of cells positive for selected markers in the two OV030 patient clones, error bars indicate mean ± SD. **g** Distribution of flow cytometry-based characterization of cell population in OV030-R and OV030-S clones. Abbreviations used in the figures: DSS—drug sensitivity score, T and A in the annotations represent tumor tissue and ascites location respectively.

In our DST data BH3 mimetics demonstrate a unique selective cytotoxicity towards OC cells. While some compounds, such as AT 101 and Sabutoclax showed the strongest cytotoxic effects, these drugs also exhibited high killing in non-cancerous models (average DSS > 11 in bone marrow), indicating limited selectivity. These compounds also showed stronger cytotoxic effects in our models, which is consistent with their known broader activity profiles, including induction of cell death through non-apoptotic or atypical pathways^35^. Interestingly, we observed a variable sensitivity to Bcl-xL inhibitors: A-1331852, and A-1155463 (Fig. 3a). This trend was particularly evident in the OV030 PDMs, generated from different ascites fractions of a patient who exhibited a partial treatment response followed by rapid relapse within four months (Supplementary Fig. 3a). As treatment resistance is common among OC patients, models recapitulating this phenotype are especially valuable for identifying novel therapeutic targets. Despite genetic similarity among four models (OV030-R, OV030-A3, OV030-S, and OV030-A2) (Supplementary Fig. 3b), they exhibited distinct drug responses. The OV030-R clone was resistant to both A-1331852 and A-1155463 (viability >50%), whereas OV030-S and OV030-A2 clones were consistently sensitive to both drugs (viability <50%). In contrast, OV030-A3 showed greater resistance to A-1331852, but not A-1155463 (Supplementary Fig. 3c). A comparison of response to 528 drugs between OV030-R and OV030-S, the two clones with the most divergent phenotypes, further highlighted Bcl-xL inhibitors as key class showing differential sensitivity (Fig. 3b). Interestingly, the protease inhibitors, Oprozomib and Bortezomib, have been also observed with opposing effect, showing higher killing of OV030-R, but not OV030-S, however, both of these drugs demonstrated high toxicity in bone marrow and PDFs, limiting their repurposing potential (Supplementary Fig. 3d). Validation of Bcl-xL inhibitor efficacy over an extended concentration range confirmed the initial screening results, with A-1331852 consistently showing greater potency than A-1155463 (Fig. 3c) and increasing potency over time (Supplementary Fig. 3e). Notably, Bcl-xL protein levels between the clones remained unchanged upon treatment (Fig. 3d, Supplementary Fig. 3f). Moreover, while fast proliferation is often associated with increased sensitivity to treatment^36^, particularly to Bcl-xL inhibitors^37^, the resistant OV030-R clone exhibited a higher proliferation rate (Supplementary Fig. 3g). Growth rate (GR) corrected drug response analysis (Fig. 3e) confirmed that both Bcl-xL inhibitors induced strong cytotoxic effect in the OV030-S model (GR < 0). In contrast, in the OV030-R model, proliferation was reduced, but overall cell growth was not significantly impaired (GR > 0), consistent with our previous observations. Taken together, these data suggest that resistance may be driven by alternative survival mechanisms rather than differences in target expression. To further compare the cellular phenotypes of these clones, we analyzed the expression of various cancer and tumor microenvironment (TME)-associated markers (Fig. 3f, Supplementary Fig. 3h). Both clones displayed a strong epithelial phenotype, with the majority of cells (average 95%) expressing cytokeratin 8/18 (CK8/18). In addition, alpha-smooth muscle actin (α-SMA), a marker associated with cancer aggressiveness and previously reported in serous OC subtypes^38^, was expressed in 99% of cells in both clones. Fibroblast-specific protein-1 (FSP-1) was detected in more than half of all cells (69%), indicating a potential shift towards a more invasive phenotype^39^. Furthermore, both clones expressed OC-associated markers, paired box gene 8 (PAX8) and mucin 16 (MUC16). However, OV030-S exhibited a lower proportion of PAX8 and MUC16 positive cells compared to OV030-R. Consistent with their epithelial phenotype, neither of the models showed significant expression of the mesenchymal marker vimentin (VIM), with only 4% positive cells. Finally, testing of surface markers confirmed the epithelial nature of both OV030 models (Fig. 3g). The majority of cells in each clone were EpCam+ cells with 93.3% in OV030-S and 77.4% in OV030-R. The remaining cells were mainly stromal cells (CD90 + ), comprising 20.1% in OV030-R and 4.1% in OV030-S, while the remaining ~3% of cells represent other non-epithelial populations. Together, these findings indicate that resistance and sensitivity to Bcl-xL inhibitors is not solely explained by target protein levels, proliferation rate or phenotypic differences of the models, but likely involves alternative mechanisms of survival and drug-induced apoptosis escape.

### Proteomics reveals clonal differences driving differential response to Bcl-xL inhibitor

**Fig. 4:**
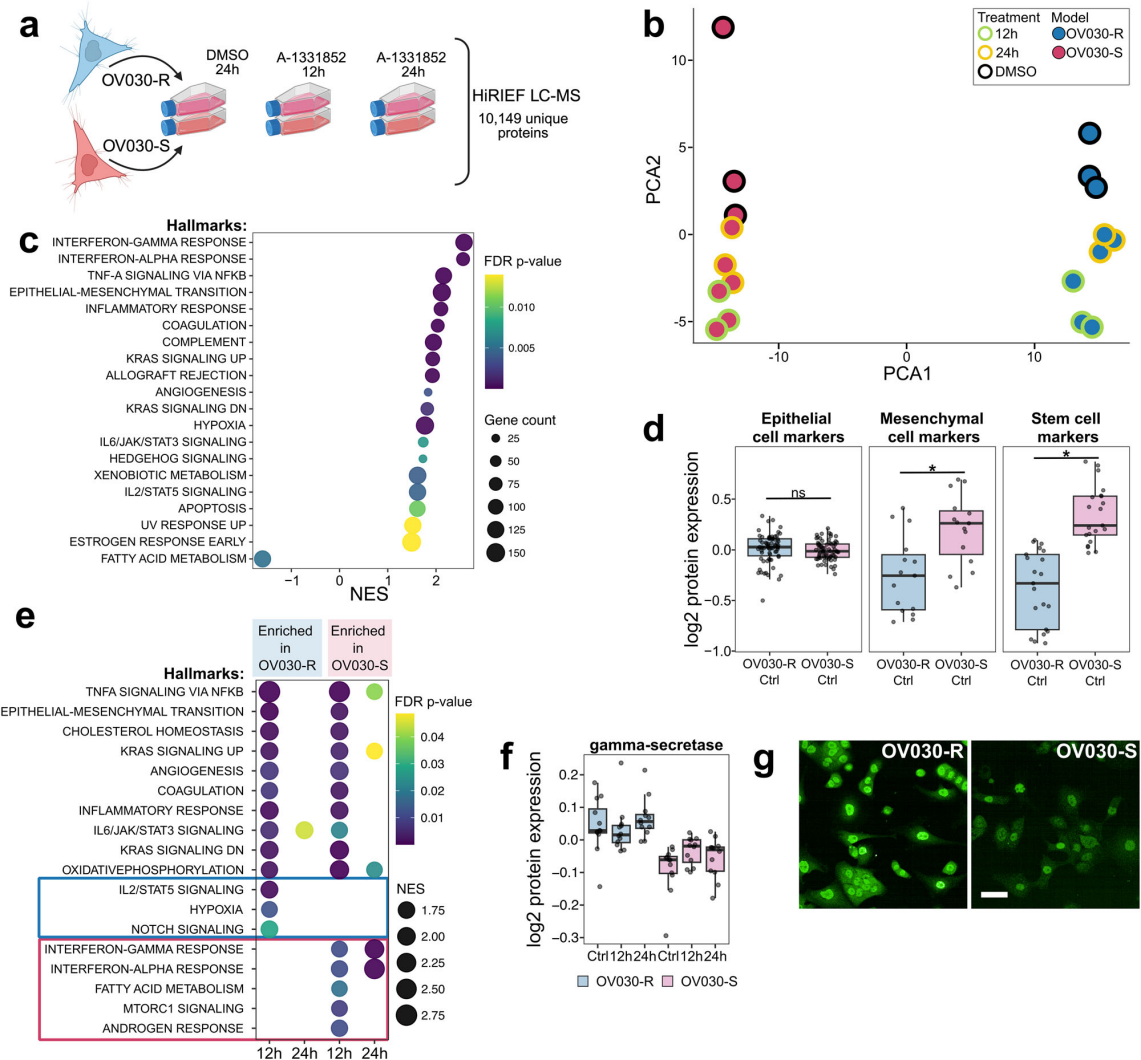
Proteomics analysis reveals pathways driving differential responses to A-1331852. **a** Schematic overview of the experimental design for HiRIEF LC-MS based proteomics analysis (created with Biorender.com). **b** PCA showing clustering of OV030-R and OV030-S clones based on protein expression profiles, with and without A-1331852 treatment. **c** Gene set enrichment analysis (GSEA) using Hallmarks gene sets comparing untreated OV030-R and OV030-S clones, shown as normalized enrichment score (NES), FDR *p*-values and gene count. **d** Boxplots comparing expression of EMT signature and CSC-associated proteins between untreated OV030-R and OV030-S, *p < 0.05 from Mann-Whitney U test. **e** GSEA results showing treatment induced changes in hallmark pathways between OV030-R and OV030-S, shown as NES with FDR *p*-values. **f** Boxplots representing expression levels of proteins comprising the γ-secretase complex in OV030-R and OV030-S clones (*n* = 3 in each condition and gene). **g** Representative IF images illustrating expression of HES1 in nucleus. Scale bar = 50 µm.

To better understand the molecular mechanism underlying the differential drug responses, we performed proteomics profiling using HiRIEF LC-MS^40^ on untreated and A-1331852 treated OV030 clones, identifying over 10,000 unique proteins (Fig. 4a). Dimensionality reduction analysis revealed clear intrinsic proteomic differences between OV030-R and OV030-S (Fig. 4b). Moreover, both models exhibit a distinct shift following the treatment (Fig. 4c), supporting the functional heterogeneity observed in the drug screening. The OV030-S showed enrichment in immune and stress response pathways, including interferon α/γ response and TNF-α signaling via NF-κB, suggesting an apoptosis-primed phenotype^41,42^. In addition, enrichment of epithelial-mesenchymal transition (EMT), KRAS signaling, and apoptosis supports increased dependency on anti-apoptotic proteins for survival^43,44^. In contrast, OV030-R exhibited enrichment in fatty acid metabolism, suggesting a metabolically altered state^45^. Comparison of EMT and cancer stem cell (CSC) associated protein (Sup. Table 5) expression profiles supported these observations (Fig. 4d). Based on an OC EMT signature^46^, both OV030-R and OV030-S exhibited similar levels of proteins representing epithelial phenotype. However, OV030-S showed significantly higher expression of proteins linked to mesenchymal phenotype compared to OV030-R (*p* = 0.002) also observed in FACS analysis. In addition, OV030-S displayed elevated expression of proteins associated with CSC characteristics (*p* = 0.002). These protein expression patterns remained largely unchanged upon treatment with A-1331852 (Supplementary Fig. 4a). However, A-1331852 treatment induced activation of several overlapping and distinct pathways in the two clones (Fig. 4e). In the A-1331852 sensitive, OV030-S clone, treatment led to suppression of interferon α/γ response and fatty acid metabolism along with upregulation of mTORC1 signaling and androgen response. In contrast, the resistant OV030-R clone exhibited enrichment in IL2-STAT5, NOTCH signaling, and hypoxia, changes which may reflect a metabolic shift supporting alternative survival pathways and reduced dependency on Bcl-xL^47–49^. Among the NOTCH pathway proteins, several proteins comprising γ-secretase, a key enzyme in NOTCH activation^50^, were significantly upregulated in OV030-R when compared to OV030-S (*p* < 0.001) (Fig. 4f, Supplementry Table 5). Given the known link between NOTCH signaling, EMT, and stemness phenotypes^49^, we hypothesized that targeting NOTCH pathway could help overcome resistance to Bcl-xL inhibition. Additionally, increased expression of HES1, a key downstream target of NOTCH signaling^51^, further supported the proteomics data. IF analysis confirmed elevated HES1 levels in the OV030-R clone compared to OV030-S after 24 h of culture, both with and without the treatment (Fig. 4g, Supplementary Fig. 4b). These findings highlight NOTCH signaling as a potential resistance mechanism to Bcl-xL inhibition and provide a strong rationale for combinatorial targeting strategies to overcome therapeutic resistance in OC.

### Targeting γ-secretase overcomes resistance to Bcl-xL inhibition

**Fig. 5:**
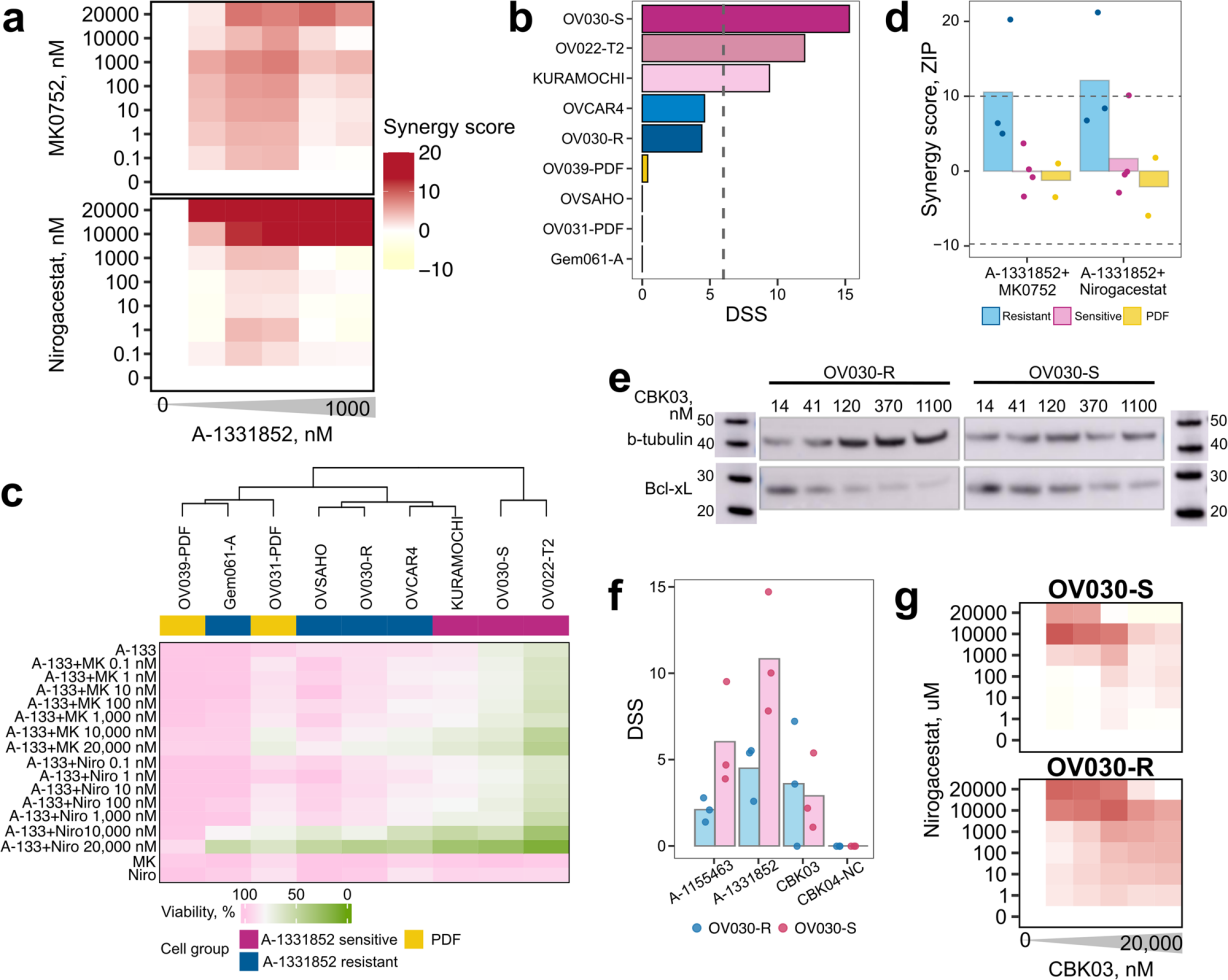
v-secretase inhibition synergizes with Bcl-xL inhibitors. **a** Synergy score heatmaps for A-1331852 and γ-secretase inhibitors (GSIs) combination treatment in the OV030-R model. **b** Bar plot displaying DSS to A-1331852 across various OC cell models. **c** Heatmap illustrating cell viability after single and combination treatment with A-1331852 and GSIs across tested models. **d** ZIP synergy scores visualized as bar plots for A-1331852 and GSIs combinations. **e** Protein bands representing the signal detected by Western blot marked for Bcl-xL and b-tubulin as reference protein. On the sides molecular weight marker (Magic Marker) sizes are indicated in kDa. **f** Bar plot displaying OV030-R and OV030-S response to Bcl-xL targeting compounds. **g** Synergy score heatmaps for CBK03 and Nirogacestat combination treatment in the OV030 models. Abbreviations used in the figures: DSS—drug sensitivity score, PDF—patient-derived fibroblast.

To evaluate the combination effects of A-1331852, we tested two γ-secretase inhibitors (GSIs) MK-0752 and Nirogacestat, across a panel of OC models. MK-0752 has previously demonstrated efficacy in phase I trial in combination with Gemcitabine^52^, while Nirogacestat is approved for the treatment of desmoid tumors^53^. A combination of A-1331852 and GSIs showed additive effects in both the resistant OV030-R and sensitive OV030-S models (Supplementry Table 6). The OV030-R model demonstrated stronger drug interactions (Fig. 5a), with overall ZIP synergy scores ranging from 5 to 6.7 for MK-0752 and Nirogacestat, respectively. Whereas the OV030-S model showed only mild additive effects (Supplementary Fig. 5a), with overall ZIP scores around 0. Among the two GSIs tested, the combination with Nirogacestat showed higher cell killing. Further based on prior results, we selected a broader panel of OC models and PDFs, both showing A-1331852-sensitivity and -resistance (Fig. 5b, Supplementary Fig. 5b) to test these drug combinations. As observed previously, the A-1331852-sensitive group (OV030-S, OV022-T2, KURAMOCHI), showed significant response to A-1331852 monotherapy (average DSS = 10.6), while the resistant group (OV030-R, OVSAHO, OVCAR4, Gem061-A) exhibited limited sensitivity (average DSS = 2.4). In contrast, neither of tested GSIs showed cytotoxicity as monotherapies (DSS < 6) (Supplementary Fig. 5c). However, the combination of A-1331852 with GSIs enhanced cancer cell killing compared to monotherapy (Supplementary Table 7). Importantly, PDFs remained highly viable (viability>90%) under all treatment conditions (Fig. 5c), indicating selective cytotoxicity towards cancer cells. To evaluate synergistic effects, ZIP synergy scores were calculated (Supplementary Table 6). The A-1331852-resistant group showed the high additivity (ZIP = 8.4 for Nirogacestat, 7.1 for MK-0752), while the A-1331852-sensitive group displayed only modest additive effects, likely due to the effectiveness of A-1331852 alone in these models (ZIP = 6.7 or 3.7, respectively) (Fig. 5d).

Although we observed enhanced killing with the drug combinations, the clinical applications of A-1331852 are limited due to on-target toxicity^54^. As a potential alternative, targeted protein degraders have been shown to offer greater selectivity, thereby potentially improving tolerability^55^. To explore this, we evaluated the Bcl-xL-targeted degrader (CBK03) along with its negative control, CBK04-NC, lacking VHL-binding activity. Consistent with previous studies^56^, CBK03 exhibited a killing of MOLT-4 cells, a Bcl-xL-dependent line^57^ while CBK04-NC showed no effect. CBK03 exhibited potent activity with DSS of 25.5 and EC_50_ of 43.5 nM. However, both Bcl-xL inhibitors (A-1331852 and A-1155463) showed greater potency with lower EC_50_ values of 1.8 and 9.3 nM, respectively (Supplementary Fig. 5d). In addition, Navitoclax, a dual Bcl-xL/Bcl-2 inhibitor, displayed minimal cytotoxicity in these models. Despite low DSS, CBK03 treatment led to dose-dependent Bcl-xL degradation in both OV030-R and OV030-S models, as confirmed by Western Blot analysis (Fig. 5e, Supplementary Fig. 5e). This finding may reflect enriched during model establishment, as the development protocol and culture conditions could support the expansion of *TP53*-mutant clones Combination treatment with CBK03 and Nirogacestat led to additive effects in OV030-R and OV030-S (ZIP = 6.4 and 2.3, respectively) (Fig. 5g) and slight antagonism in the OV039-PDF (ZIP = −1.6), showing a similar pattern to the combination with A-1331852 (Supplementary Table 6). Overall, these results suggest that the combination of GSI and Bcl-xL targeting compounds enhances cytotoxic effects without increasing the toxicity in PDFs.

### A-1331852, Nirogacestat and Carboplatin triple combination shows significant killing in long-term assay and ex vivo samples

**Fig. 6:**
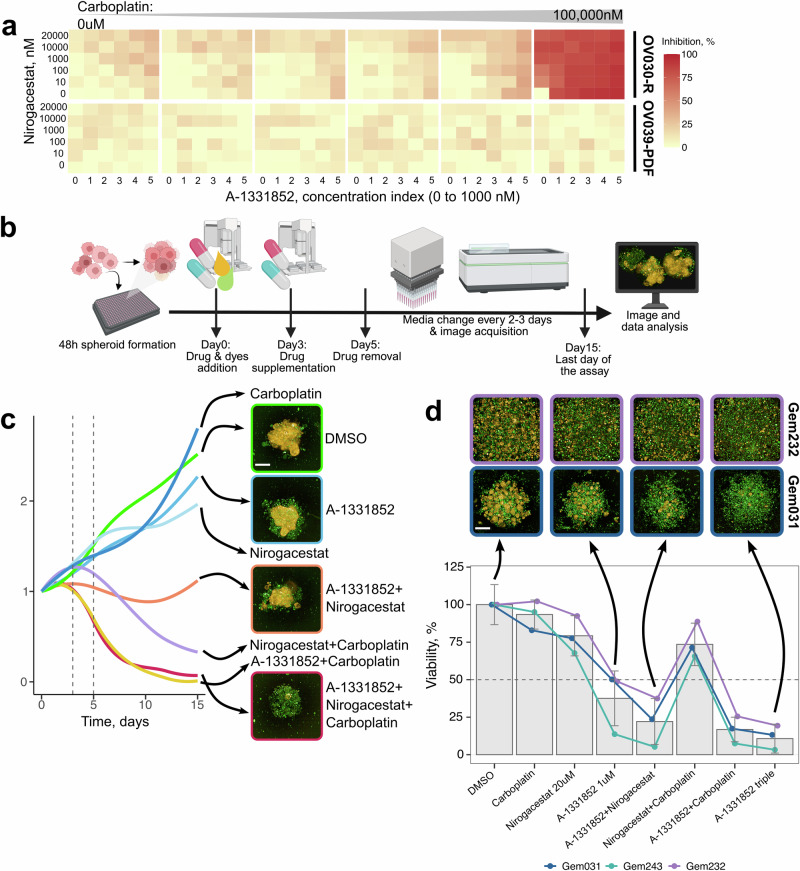
The combination of A-1331852, Nirogacestat, and Carboplatin induces strong cytotoxicity in long-term assays and ex vivo cultures. **a** OV030-R and OV039-PDF cell growth inhibition when treated with A-1331852, Nirogacestat and Carboplatin three drug combinations. **b** Schematic overview of long-term drug treatment and drug removal assay (created with Biorender.com). **c** Boxplots representing spheroid volume change at the first and last time points of the assay following treatment with A-1331852 or DT2216 in combination with Nirogacestat. Arrows indicate the representative images of spheroids at day 15 under indicated treatment conditions. Green signal marks dead, yellow indicates live cells, scale bar is 200 mm. **d** Ex vivo spheroid viability in response to A-1331852 based treatments at the highest concentrations. Error bars represent mean ± SD (*n* = 3). Arrows indicate the representative images of spheroids at day seven under indicated treatment conditions. Green signal marks dead, yellow indicates live cells. Images representing difference samples are outlined with distinct border colors, scale bar is 200 µm.

Carboplatin remains the backbone of OC treatment, while it is often combined with other chemotherapeutics, high relapse rates and the development of resistance significantly limit its long-term efficacy^58^. Considering our earlier findings showing the synergy of Bcl-xL-targeting compounds with Nirogacestat, we next evaluated whether this combination could further enhance the effect of Carboplatin in resistant cells. Our results showed that the combination of A-1331852 and Carboplatin exhibited only mild synergy (ZIP < 4.5) (Supplementary Fig. 6a). In contrast, CBK03 and Carboplatin combination demonstrated a stronger synergistic effect, reaching a ZIP score of 15 in the OV030-R model (Supplementary Fig. 6b). Based on these findings, next, we evaluated the three-drug combinations of either A-1331852 or CBK03, Nirogacestat, and Carboplatin across a range of concentrations of each compound. In both OV030-R and OV030-S models, we observed strong synergistic interactions, with ZIP > 18 for both three drug combinations. Importantly, these combinations demonstrated minimal cytotoxicity in non-cancerous OV039-PDF. In this model the combination containing A-1331852 showed a modest additive effect (ZIP = 6.5), while the CBK03-based combination exhibited a slight antagonistic profile (ZIP = −4.5), suggesting that different modes of targeting Bcl-xL may lead to distinct response profiles (Fig. 6a, Supplementary Fig. 6c). To assess the durability of these effects and better mimic clinical treatment conditions, we established a long-term treatment assay (Fig. 6b). This approach enables the assessment of long-term treatment with drug removal in 3D cultures, enabling the use of a more physiologically relevant model and better recapitulating aspects of TME within tumor-like structures^59^. Here, OV030-R, OV030-S and OV039-PDF were cultured in 3D, treated for five days, after which the drugs were removed and cells maintained for up to 15 days. Our results showed that none of the monotherapies (A-1331852, CBK03, Nirogacestat, Carboplatin) led to a significant reduction in spheroid volume over time (Supplementary Fig. 6d). In line with short-term monolayer assay results, A-1331852 or CBK03 combination with Nirogacestat suppressed cell growth over time. However, though spheroid volume remained smaller than control, this resulted in a maintenance or cytostatic effect, rather than complete cancer cell eradication during long-term culture. In contrast, the three drug combination of either A-1331852 or CBK03 with Carboplatin and Nirogacestat resulted in a robust and sustained cytotoxic response. Cell growth was markedly suppressed over time with no significant regrowth during the 15-day period (Fig. 6c, Supplementary Fig. 6e-f), suggesting a potentially durable antitumor effect. Interestingly, while no synergy was observed in short-term 2D cultures (Supplementary Fig. 6a-b) the combination of Nirogacestat and Carboplatin demonstrated strong and lasting cytotoxicity in long-term spheroids, although not to the same extent as three-drug combinations and the onset of cell killing was observed at a later time point than the three-drug combination. In addition, similar effects were observed at lower drug concentrations (Supplementary Fig. 7a). In OV039-PDF these treatments caused only moderate reduction in spheroid volume, with less pronounced effects compared to cancer models, supporting the selectivity of tested treatments to the cancer compartments (Supplementary Fig. 7b). In addition, microscopy images of the spheroids clearly illustrated the volume changes upon treatment (Fig. 6c, Supplementary Fig. 7c-d). Surprisingly, although the combination of A-1331852 or CBK03 with Carboplatin showed only moderate additive effects in our previous short-term monolayer assays, it induced a pronounced and durable cytotoxic response in the long-term spheroid assay, regardless of initial A-1331852 sensitivity status (Supplementary Fig. 8a). These findings support the A-1331852 and Carboplatin combination as a promising alternative combination therapy. None of the drug treatments significantly altered HES1 expression levels; however, our results revealed distinct kinetics of HES1 activation profiles between the two models (Supplementary Fig. 8b). OV030-R exhibited higher baseline HES1 expression, while OV030-S showed delayed activation over time. These temporal differences in NOTCH pathway activation may contribute to differential sensitivity to Bcl-xL targeting compounds.

We then evaluated the proposed drug combinations in ex vivo cultures from three patients where we have clinical outcome to standard-of-care treatment. (Fig. 6d). Two of these samples, which showed an either increased or stable spheroid size over the seven days (Gem031, Gem232) (Supplementary Fig. 8c), displayed limited sensitivity to A-1331852 or CBK03 monotherapy, with viability remaining above 50%. In contrast, the third sample (Gem243) that exhibited slower proliferation and gradual spheroid shrinkage over time showed a strong response when treated with Bcl-xL targeting compounds, with average viability reduced to 13.3% when treated with A-1331852 and 37.9% with CBK03. Monotherapy with either Carboplatin or Nirogacestat had minimal effect on cell survival, with an average viability of 87%. However, the combination treatments yielded more pronounced effects. Both A-1331852 and CBK03 combined with Nirogacestat substantially reduced spheroid viability across samples, with A-1331852 exhibiting a stronger cytotoxic effect compared to CBK03 (average viability 27.6 and 53.0%, respectively). In contrast, the combination of Nirogacestat and Carboplatin did not show strong efficacy in three samples (viability 77.1%), despite showing more potent effects in long-term treatment in OV030 models. This may reflect the later onset of efficacy observed in the long-term treatment assays. Importantly, the three drug combination of A-1331852 or CBK03 with Carboplatin and Nirogacestat led to strong and consistent cell killing across all ex vivo samples. A-1331852-based combinations were more effective, reducing viability to 21.2% compared to 37.0% when treated with CBK03-based combinations. The ex vivo patient samples were also sensitive to A-1331852 or CBK03 and Carboplatin combination, as shown in the long-term treatment assay. The trends were consistent across different tested drug concentrations (Supplementary Fig. 8d). In addition, sample characterization revealed that all three tested ex vivo cultures contained a similar proportion of EpCam+CD24- cancer cells but differed in the abundance of other cancer cell subpopulations (EpCam+CD24+ or EpCam+CD90 + /PDPN + ) (Supplementary Fig. 8e). These variations did not significantly impact sensitivity to the tested treatments. Importantly, the combination was effective in samples from both patients who experienced early relapse of 9 and 3 months for Gem031 and Gem232, respectively, and those with no disease progression to date (Gem243, 17 months since start of the treatment), highlighting its potential broad applicability.

Together, these findings support the therapeutic potential of Bcl-xL targeting compounds in combination with Carboplatin and/or Nirogacestat for the treatment of resistant OC. While our data indicate a role of NOTCH signaling in modulating response Bcl-xL inhibition, the molecular mechanism underlying the observed synergy is likely multifactorial and remains to be fully elucidated. Nevertheless, our results highlight the promise of combining GSIs with Bcl-xL inhibitors with Carboplatin to enhance therapeutic efficacy in vitro and ex vivo patient models.

## Discussion

In this study, we aimed to identify novel treatment strategies for OC using PDMs that better preserve genetic, molecular, and phenotypic features of primary tumors compared to conventional CCL models. Through systematic generation and characterization of diverse collection of PDMs representing multiple OC subtypes, followed by high-throughput drug screening, we aimed to uncover patient specific vulnerabilities and explore rational combination strategies that could improve current OC management.

Pre-clinical oncology research often heavily relies on CCLs, which, while robust, scalable and easy to handle^11^, typically harbor extensive genetic drift and exhibit profiles that are not physiologically representative of the disease or subtype. As a result, CCLs may fail to capture the genetic and phenotypic diversity found in patient tumors^11,60^. In contrast, PDMs retain patient-specific molecular features and therefore provide a more clinically relevant platform for therapeutic research, despite their technical limitations^15^. In this study, we present a comprehensive collection of PDMs from OC patients, spanning a wide range of histological subtypes and clinical outcomes. The high concordance of CNVs and mutational profiles between original material and corresponding PDCs supports the validity of these models for both functional and therapeutic research. As expected, the majority of HGS-OC models harbored *TP53* mutations, consistent with its >90% prevalence in this subtype^7^. Interestingly, we also observed aberrant *TP53* in several PDMs derived from rare OC subtypes, in which *TP53* alterations are typically less common^10^. This finding may reflect enriched during model establishment, as the development protocol and culture conditions could support the expansion of *TP53*-mutant clones. Aside from alterations in *TP53*, a substantial proportion of our models carried alterations in *BRCA1/2*, as well as in *BRIP1* and *PALB2*, genes involved in HRR pathway^61,62^, reinforcing the potential clinical significance of these less-studied alterations in OC. Our models also preserved subtype-specific alterations, such as *ARID1A*, *KRAS*, *CDKN2A* mutations, confirming their ability to maintain molecular hallmarks of diverse OC subtypes^7^. Interestingly, one HGS-OC case (OV022) harbored a loss-of-function mutation of *SMARCB1*, a key protein of the SWI/SNF chromatin remodeling complex^63^, leading to changes in gene expression^64^. Although typically associated with rhabdoid tumors^64^ and epithelial sarcomas^65^ *SMARCB1* mutant OC has been detected in rare cases of SCCOHT^66^. The rapid relapse of OV022 patient following standard platinum and taxol treatment suggests that genomics profiling could have enabled a more personalized therapeutic approach. These findings underscore the genetic heterogeneity of OC, even within a relatively small PDM collection. They further highlight the need to move beyond uniform treatment strategies toward precision medicine-guided treatment to improve patient outcomes.

While OC lacks targetable genetic alterations and patients often experience rapid relapse, drug repurposing offers an effective strategy to uncover novel therapeutic vulnerabilities^67^. In this study, a high-throughput drug screen of 528 compounds across cancerous and non-cancerous models revealed distinct tumor-specific drug sensitivities. While CCLs displayed greater sensitivity than PDCs, this exaggerated responsiveness exemplifies their limited translational use. Nevertheless, several drug subclasses demonstrated cancer-specific responses. Among these, EGFR and ERBB2 inhibitors were prominent. Although EGFR-targeted therapies have shown benefit in other solid tumors^68^, clinical response in OC has been limited^69^. Afatinib, an irreversible EGFR and HER2/4 inhibitor approved for non-small cell lung cancer (NSCLC)^70^, demonstrated the highest selectivity for cancer cells in our dataset. While underrepresented in OC, prior work has shown its synergy with Paclitaxel in advanced solid tumors, including OC^71^. Another promising subclass was rapalogs, which showed notable activity in several PDCs. Given that PI3K-AKT-mTOR pathway is frequently upregulated in OC^72^, these drugs hold considerable therapeutic potential. mTOR inhibitors, such as Everolimus, are already approved for other malignancies, including breast^73^ and renal^74^ cancers. Previous studies suggest that mTOR inhibition can reduce metastatic potential^75^ and re-sensitize platinum-resistant OC cells^76^. Although toxicity remains a consideration, phase I trial data supported the safety of combining Ridaforolimus with Paclitaxel and Carboplatin in several solid tumors^77^. BH3 mimetics also emerged as a promising subclass. Given that apoptosis dysregulation is a cancer hallmark^78^, these drugs, by antagonizing anti-apoptotic proteins, have achieved great success in hematological malignancies, where Venetoclax has markedly improved the outcomes in chronic lymphocytic (CLL) and acute myeloid (AML) leukemias^32,79^. While such malignancies typically depend on Bcl-2 for survival^80^, OC appear more reliant on Bcl-xL^81^. Our findings highlight substantial intra- and inter-patient heterogeneity in response to Bcl-xL inhibitor. However, the absence of a clear genomics-based biomarker for Bcl-xL dependency^82^ underscores the need for alternative predictive methods, such as functional profiling.

In our models, differential Bcl-xL inhibitor sensitivity occurred despite the high genomic similarity in a group of PDMs derived from the same patient, suggesting additional determinants. One such difference might be related to varying CSC proportion. Prior studies have reported elevated Bcl-xL levels in CSCs^44^, consistent with our observations. Other resistance mechanisms may involve activation of compensatory survival pathways, as described in colorectal cancer^83^, or the influence of the TME. For example, stromal cells can protect tumor cells from Bcl-xL inhibition by promoting alternative survival mechanisms^84^, which is in line with our findings. However, further mechanistic exploration using proteomics analysis revealed additional resistance-associated pathways, including NOTCH signaling, which has been linked to reduced BH3 mimetic sensitivity in hematological malignancies^85,86^. This observation provided the rationale for combining GSIs with Bcl-xL inhibitors to target both sensitive and resistant subpopulations. Indeed, we observed synergistic cytotoxicity in short-term assays, long-term 3D assays, and ex vivo samples, consistent with findings in multiple myeloma using GSI and dual Bcl-2/Bcl-xL inhibitor ABT-737^86^. Unexpectedly, we also found that A-1331852 synergized with Carboplatin in long-term spheroids and ex vivo cultures, paralleling earlier reports for ABT-737^87^. This may reflect increased Bcl-xL expression in platinum-resistant cells^88^. Finally, the triple combination of GSI, A-1331852, and Carboplatin achieved sustained cell death without regrowth, both in long-term cultures and in ex vivo patient samples, highlighting its translational potential. In addition, similar results were observed when using a targeted protein degrader of Bcl-xL (CBK03), supporting alternative clinically relevant treatment options^89^.

Our studies highlight some important learnings in reproducible drug profiling. While patient-derived drug sensitivity profiling provides a powerful platform for identifying candidate therapies, interpretation of these results requires consideration of several factors beyond in vitro response, including clinically achievable drug exposure, toxicity, treatment scheduling, assay duration, and the distinction between overall combination efficacy and formal synergy. Accordingly, functional profiling should be viewed as one component of a broader translational framework integrating pharmacology, molecular profiling, and clinical context. Assay design can substantially influence the observed drug responses. For example, the short-term viability assay used in this study may underestimate the activity of compounds with delayed or replication-dependent mechanisms of action, such as PARP inhibitors^89^, whose cytotoxic effects often require multiple rounds of DNA replication. Extending the assay duration or incorporating complementary readouts could provide a more accurate assessment of biologically relevant responses to these drugs. Likewise, we observed consistent differences in response profiles for fibroblasts cultured in different media. Our analysis suggests the main drivers of this effect are the growth factors used in each media, but other additives such as the inclusion of the Rock inhibitor, may impact drug response. Thus, it is a critical factor when comparison between PDMs and CCLs is of interest. Second, it is important to note that evaluation of multi-drug efficacy is challenging due to the lack of appropriate and reliable benchmarks that accurately represent in vivo efficacy and toxicity settings. Although ZIP synergy analysis provides insight into non-additive drug interactions, combinations producing substantial absolute cell killing through complementary mechanisms may have considerable therapeutic value even in the absence of strong formal synergy^90^. Accordingly, here, we interpret ZIP scores as a complementary measure of drug interaction rather than the primary determinant of clinical potential, and we have complemented synergy scoring with viability measurements to provide a more comprehensive assessment of the true effects of complex drug treatments. While presented results demonstrate robust and consistent ex vivo activity, these DSS measured in our assays do not capture in vivo pharmacokinetic properties of the compounds or their combinations. Therefore, further in vivo studies incorporating pharmacokinetic assessment will be required to evaluate the translational potential of these potential drug combinations. Importantly, the proposed triple drug combination may also present overlapping toxicity profiles. Hence, future translational studies should consider optimized dosing strategies, including sequential or intermittent administration, mitigate the toxicity while preserving the therapeutic efficacy. Such strategies have been previously explored for GSIs^91^ in other therapeutic contexts, and may be relevant for the clinical development of the combination proposed in this study.

Taken together, our findings highlight the value of PDMs as a powerful platform to identify actionable therapeutic vulnerabilities in OC. Although we explored in detail only one case, the dataset generated here can serve as a resource for investigating vulnerabilities across OC subtypes, including the rare ones lacking reliable CCLs models. Importantly, we identify a NOTCH-modulated Bcl-xL survival axis as a potential therapeutic target in platinum-resistant disease and provide pre-clinical evidence supporting combination strategies involving Bcl-xL inhibitors, NOTCH pathway blocking, and platinum chemotherapy. Our findings highlight the potential of physiologically relevant patient-derived models to inform precision oncology approaches and accelerate the development of rational combination therapies with treatment-resistant OC.

## Methods

### Patient sample processing and patient-derived cell model development

Patient-derived models representing cancer and fibroblast cell populations were derived from OC patient tumor tissue or ascites. Patient samples were obtained from Karolinska University Hospital (KUH) in Stockholm. The study has been performed in accordance with the Declaration of Helsinki (ethical approvals from Etikprövningsmyndigheten (Swedish Ethical Review Authority): 2016/1197–31/1, nr.2018/2642-32, nr.2018/118-32, 2020-05830). All patients have signed informed consent prior to the inclusion and material collection. Samples were processed as described by Åkerlund et al.^30^ using gentleMACS tissue dissociation kit for tumor tissue (Miltenyi) and centrifugation for ascites. After sample dissociation, cells were plated into cell culture flasks. Once the flask reached 70-80% confluency, differential trypsinization was performed. Specifically, after 3 min incubation with TrypLE (Gibco), the dissociated cell fraction was collected, resuspended in Fibroblast culture media, and spun down. The remaining cells were washed with PBS and additionally incubated with TrypLE until detached. When cancer and/or fibroblast models were proliferating consistently, cells were biobanked, collected as a 1 × 10^6^ cell pellets for further characterization and plated for DST. Overall PDC generation success rate from tissue and ascites was ~30%, while for PDFs it was ~57% with most of the PDF models derived from tissue samples rather than ascites. Clinical information describing the characteristics of the patient material is provided in Supplementary Table 2.

### Cell culture

PDCs were maintained in Rockit media as previously described by Åkerlund et al.^30^, while PDFs were cultured in Fibroblast media as stated by Gudoityte et al.^26^. A panel of CCLs representing OC was designed including the following cells and media conditions—A2780, EFO21 (DSMZ), KURAMOCHI, MH, OVCAR3, OVCAR4, OVCAR8, ONCODG1, OVSAHO, OVTOKO—were maintained in RPMI (BioWest) medium. CAOV3 (ATCC), COV362 (Sigma-Aldrich), NCI-ADR-RES, OAW28 (Merck), TYKNU were cultured in DMEM (Sigma-Aldrich). CCLs media were supplemented with 10% fetal bovine serum (FBS) (Gibco), 1X antibiotics (streptomycin/penicillin) (Gibco) and 1X L-glutamine (Gibco), unless otherwise specified. OAW28 cell media was additionally supplemented with 5 µg/mL insulin (Merck). FUOV1 cells (DSMZ) were cultured in DMEM-F12 culture media supplemented with 20% FBS and 1X antibiotics. T-lymphoblast cell line MOLT-4 was maintained in RPMI supplemented with 10% FBS and 1X antibiotics. All cells were maintained at 37 °C in a humidified incubator with 5% CO_2_ and passaged at ~70% confluency. All CCLs were obtained from the Institute Molecular Medicine Finland (FIMM) and kindly provided by Dr. Astrid Muramägi, unless stated otherwise. CCL identity was confirmed by short-tandem repeat (STR) profiling and routinely screened for mycoplasma contamination using MycoAlert (Lonza).

### Drug sensitivity testing

PDCs, PDFs, CCLs, and healthy bone marrow aspirates (*n* = 2, VWR) were profiled for DST using a drug library containing up to 528 approved and investigational compounds, as previously described^92^, custom drug plates used for follow-up validation. Black, clear bottom 384-well plates (Corning) were pre-spotted with drugs using an Echo 550 acoustic dispenser (Beckman/Labcyte). Cells from unprocessed healthy bone marrow were isolated as described by Struyf et al.^28^ and used directly in the DST assay with Rockit media. On the day of the assay, pre-spotted drugs were dissolved in respective culture media, and cell suspension was subsequently added using a Multidrop Combi Dispenser (Thermo Fisher Scientific). Plates were incubated for 72 h in a humidified chamber at 37 °C. Cell viability was assessed using the CellTiter-Glo 2.0 Cell Viability Assay (Promega), luminescence was measured using the EnSight multimode plate reader (Revvity). Models were drug tested and molecularly characterized at the same passage, when sufficient cell number was achieved to perform these assays. This information is provided in Supplementary Table 8.

### Model validation and mutation characterization

Snap-frozen tumor tissues, isolated cells from ascites, and cell pellets from PDMs were used for targeted sequencing to compare mutational profiles. Sample preparation and sequencing were performed as previously described by Gudoityte et al.^26^.

### MS-based proteomics and sample preparation

OV030 patient models: OV030-R and OV030-S, were plated into 6-well plate at a density of 0.5 × 10^6^ cells per well, two wells per condition, with three technical replicates for each. A-1331852 (1000 nM in DMSO, MedChemExpress) or DMSO (0.01%, Sigma-Aldrich) were added at the time of plating. Cells were incubated for 12 and 24 h before protein extraction. Further cells were harvested and prepared for proteomic analysis as reported earlier^93^. Specifically, 30 μg of peptides from each digested sample were labeled with TMT 18plex reagent according to the manufacturer’s protocol. After a label check, the samples were pooled and pre-fractionated on pH 3–10 strips using the HiRIEF protocol as previously described^40^. The extracted peptide fractions were separated using an Ultimate 3000 RSLCnano system coupled to a Q Exactive HF (Thermo Fisher Scientific) as previously described^93^. Peptide and protein identification was performed as published previously^93,94^. The following pipeline and software versions were used: MSGF+ v2020.03.14, Percolator v3.04.0, Nextflow v2.16, lehtiolab/ddamsproteomics v2.16. All searches were done against the human protein database Ensembl 110. Fixed modifications were TMT-18plex on lysines and peptide N-termini, and carbamidomethylation on cysteine residues. A variable modification was used for oxidation on methionine residues. Peptide spectrum matches (PSM) found at 1% false discovery rate (FDR) were used to infer gene identities. The raw files and the final output represented as log_2_ expression normalized to sample median are provided in ProteomeXchange Consortium via the PRIDE partner repository with the dataset identifier PXD065781.

### General chemistry methods used for synthesis of Bcl-xL targeted-protein degrader and inactive control compound

^1^H NMR were recorded on a Bruker DRX-400 NMR spectrometer. Analytical HPLC-MS was performed on an Agilent MSD mass spectrometer connected to an Agilent 1100 system using two different methods. The first method (acidic pH) used column ACE 3 C8 (50 × 3.0 mm), and H_2_O ( + 0.1% trifluoroacetic acid) and acetonitrile as mobile phases. The second method (basic pH) used column X-Terra MSC18 (50 × 3.0 mm), with H_2_O (containing 10 mM NH_4_HCO_3_; pH = 10) and acetonitrile as mobile phases. Both methods were performed at a flow rate of 1 mL/min, with a gradient time of 3.0 min. HPLC-MS detection was performed in the UV in the 305 + /-90 nm wavelength range and by MS (ESI + ). All final compounds were assessed to be >95% pure by HPLC-MS UV analysis and provided in Supplementary Fig. 1a.

### Navitoclax-piperazine synthesis

Navitoclax-piperazine (1) was made as described^95^ except for one reaction described below.

*2,2,2-trichloroethyl (R)-4-(3-((tert-butoxycarbonyl)amino)-4-(phenylthio)butyl)-piperazine-1-carboxylate* Sodium triacetoxyborohydride (73 mg, 0.34 mmol) was added to a solution of tert-butyl (R)-(4-oxo-1-(phenylthio)butan-2-yl)carbamate (72 mg, 0.25 mmol), 2,2,2-trichloroethyl piperazine-1-carboxylate (86 mg, 0.29 mmol) and acetic acid (21 µL, 0.37 mmol) in tetrahydrofuran (10 mL). The mixture was stirred for 5 h and then water (15 mL) was added, and the product was extracted with dichloromethane (3 × 20 mL). The combined organic phases were washed with brine (30 mL), dried (MgSO_4_), filtered, and evaporated to dryness to obtain a brown oil. Yield 133 mg. MS (ESI)+: calculated for C_22_H_33_Cl_3_N_3_O_4_S 540.12 [M + H]^+^; found 540.1. 1H NMR (400 MHz, CDCl3) δ ppm 7.39 (d, J = 7.6 Hz, 2H), 7.29 (t, J = 7.6 Hz, 2H), 7.19 (t, J = 7.2 Hz, 1H), 5.41 (br s, 1H), 4.75 (s, 2H), 3.96 –3.82 (m, 1H), 3.73 –3.56 (m, 4H), 3.22 (dd, J = 17.6, 7.4 Hz, 1H), 3.02 (s, 1H), 2.77 –2.22 (m, 6H), 2.03 –1.87 (m, 2H), 1.42 (s, 9H).

The active and inactive form of the VHL ligands were made with known methods^96,97^ and the linker molecules used for coupling described below.

### Synthesis of active VHL-linker

6-{[(2S)-1-[(2S,4 R)-4-hydroxy-2-({[4-(4-methyl-1,3-thiazol-5-yl)phenyl]methyl}-carbamoyl)pyrrolidin-1-yl]-3,3-dimethyl-1-oxobutan-2-yl]carbamoyl}hexanoic acid (2) Heptanedioic acid monoethyl ester (0.52 g, 2.8 mmol) was added to a solution of (2S,4 R)-1-[(2S)-2-amino-3,3-dimethylbutanoyl]-4-hydroxy-N-{[4-(4-methyl-1,3-thiazol-5-yl)phenyl]-methyl}pyrrolidine-2-carboxamide (1.2 g, 2.8 mmol), diisopropylethyhylamine (1.4 mL, 8.4 mmol) and TBTU (2-(1H-Benzotriazole-1-yl)-1,1,3,3-tetramethylaminium tetrafluoroborate) (0.98 g, 3.1 mmol) in dimethylformamide (10 mL) and stirred on at room temperature (RT). Sodium hydrogen carbonate (sat.) was added and the mixture was extracted with dichloromethane (×3). The organic phase was dried (Na_2_SO_4_) and evaporated. The product was treated with lithium hydroxide (0.2 g, 8.4 mmol) in tetrahydrofuran/H_2_O 1:1 (4 mL) over night, neutralized with acid resin (Dowex 50×8, H^+^ form; 20-50 mesh). The mixture was filtered and evaporated and redissolved in a small amount of dimethylformamide, then purified by chromatography on a short silica gel column in reverse phase, eluting with acetonitrile/H_2_O. Pure fractions were freeze dried to give the title compound as a solid. Yield 0.88 g. LC-MS: calculated for C_29_H_41_N_4_O_6_S 572.72; found 573.0[M + H]^+^. ^1^H NMR (400 MHz, d6-dimethyl sulfoxide) δ 8.98 (s, 1H), 8.56 (t, J = 6.0 Hz, 1H), 7.85 (d, J = 9.3 Hz, 1H), 7.40 (q, J = 8.4 Hz, 4H), 4.53 (d, J = 9.3 Hz, 1H), 4.49 –4.39 (m, 3H), 4.35 (s, 1H), 4.28 –3.53 (m, 2H), 2.44 (s, 3H), 2.15 (dd, J = 14.1, 6.8 Hz, 2H), 2.30 –1.83 (m, 2H), 2.11 –1.98 (m, 2H), 1.56 –1.38 (m, 4H), 1.21 (ddd, J = 19.8, 14.8, 7.4 Hz, 2H), 0.93 (s, 9H).

### Synthesis of inactive VHL-linker

6-{[(2S)-1-[(2S,4S)-4-hydroxy-2-({[4-(4-methyl-1,3-thiazol-5yl)phenyl]-methyl}-carbamoyl)pyrrolidin-1-yl]-3,3-dimethyl-1-oxobutan-2-yl]carbamoyl}hexanoic acid (3) The procedure was the same as for compound 2 using (2S,4S)-1-[(2S)-2-amino-3,3-dimethyl-butanoyl]-4-hydroxy-N-[[4-(4-methylthiazol-5-yl)phenyl]methyl]pyrrolidine-2-carboxamide. LC-MS: calculated for C_29_H_41_N_4_O_6_S 573.72; found 573.2 [M + H]^+^.

### Synthesis of active targeted-protein degrader CBK603803

(2S,4 R)-1-[(2S)-2-(7-{4-[(3 R)-3-{[4-({[4-(4-{[2-(4-chlorophenyl)-5,5-dimethylcyclohex-1-en-1-yl]methyl}piperazin-1-yl)phenyl]formamido}sulfonyl)-2-trifluoromethane-sulfonylphenyl]amino}-4-(phenylsulfanyl)butyl]piperazin-1-yl}-7-oxoheptanamido)-3,3-dimethylbutanoyl]-4-hydroxy-N-{[4-(4-methyl-1,3-thiazol-5-yl)phenyl]methyl}-pyrrolidine-2-carboxamide (CBK603803, further referred as CBK03).

HBTU (5 mg, 13 µmol) was added to a solution of (1) (8 mg, 8 µmol), (2) (5 mg, 10 µmol) and triethylamine (12 µL, 86 µmol) in dichloromethane (2 mL) and stirred on. The solvent was evaporated, and the residue was purified by basic preparative reversed phase HPLC to yield 4 mg (32%). Calculated for C_76_H_95_ClF_3_N_10_O_10_S_4_ 1527,567, Found 1527.2 (M + 1). ^1^H NMR (400 MHz, chloroform-*d*) d ppm 0.93 (s, 9 H) 0.97–1.02 (m, 6 H) 1.23–1.38 (m, 3 H) 1.47 (t, *J* = 6.25 Hz, 3 H) 1.51–1.64 (m, 5 H) 1.71 (br. s., 3 H) 2.02–2.31 (m, 13 H) 2.42 (br. s., 6 H) 2.50 (s, 3 H) 3.02 (dd, *J* = 13.70, 6.57 Hz, 2 H) 3.11 (dd, *J* = 13.57, 5.19 Hz, 2 H) 3.35 (br. s., 6 H) 3.56 - 3.62 (m, 1 H) 3.66 (br. s., 1 H) 3.92 (br. s., 1 H) 4.10 (d, *J* = 11.38 Hz, 1 H) 4.34 (dd, *J* = 15.01, 5.25 Hz, 1 H) 4.49 - 4.61 (m, 3 H) 4.74 (t, *J* = 7.94 Hz, 1 H) 6.25 (d, *J* = 9.01 Hz, 1 H) 6.64 (d, *J* = 9.51 Hz, 1 H) 6.77 (d, *J* = 8.88 Hz, 2 H) 6.99 (d, *J* = 8.25 Hz, 2 H) 7.11 (d, *J* = 8.25 Hz, 1 H) 7.27 - 7.40 (m, 10 H) 7.53 (br. s., 1 H) 7.68 (d, *J* = 8.75 Hz, 1 H) 8.09 (d, *J* = 9.26 Hz, 1 H) 8.36 (s, 1 H) 8.68 (s, 1 H).

### Synthesis of inactive targeted protein-degrader CBK603804

(2S,4S)-1-[(2S)-2-(7-{4-[(3 R)-3-{[4-({[4-(4-{[2-(4-chlorophenyl)-5,5-dimethylcyclohex-1-en-1-yl]methyl}piperazin-1-yl)phenyl]formamido}sulfonyl)-2-trifluoromethane-sulfonylphenyl]amino}-4-(phenylsulfanyl)butyl]piperazin-1-yl}-7-oxoheptanamido)-3,3-dimethylbutanoyl]-4-hydroxy-N-{[4-(4-methyl-1,3-thiazol-5-yl)phenyl]methyl}-pyrrolidine-2-carboxamide (CBK603804, further referred as CBK04-NC).

HBTU (Hexafluorophosphate benzotriazole tetramethyl uranium) (4 mg, 10 µmol) was added to a solution of 1 (7 mg, 7 µmol), 3 (5 mg, 9 µmol) and triethylamine (10 µL, 72 µmol) in dichloromethane (2 mL) and stirred for 2 h. The solvent was evaporated, and the residue was purified by basic preparative reversed phase HPLC to yield 4 mg (36%). Calculated for C_76_H_94_ClF_3_N_10_O_10_S_4_ 1527,57, Found 1527.2 (M + 1). ^1^H NMR (400 MHz, chloroform-*d*) d ppm 0.92 (s, 9 H) 1.01 (s, 6 H) 1.20–1.84 (m, 21 H) 2.02 (s, 1 H) 2.05–2.52 (m, 26 H) 2.92–3.18 (m, 4 H) 3.42 (br. s., 5 H) 3.57–3.74 (m, 2 H) 3.82 (d, *J* = 11.13 Hz, 1 H) 3.92 (d, *J* = 4.00 Hz, 2 H) 4.22–4.35 (m, 1 H) 4.42–4.49 (m, 1 H) 4.53 (d, *J* = 9.01 Hz, 1 H) 4.59–4.69 (m, 1 H) 4.73 (d, *J* = 8.76 Hz, 1 H) 5.47–5.57 (m, 1 H) 6.02–6.10 (m, 1 H) 6.63 (s, 1 H) 6.78 (d, *J* = 8.88 Hz, 2 H) 7.00 (d, *J* = 8.25 Hz, 2 H) 7.08–7.17 (m, 1 H) 7.28–7.41 (m, 10 H) 7.43–7.63 (m, 2 H) 7.68 (d, *J* = 8.38 Hz, 2 H) 8.07 - 8.13 (m, 1 H) 8.37 (d, *J* = 2.13 Hz, 1 H) 8.69 (s, 1 H).

### Immunoblotting

PDCs from OV030 patient were seeded in T25 flasks at a density of 1.3 × 10^6^ cells and treated with A-1331852 (1,000 nM) or a range of CBK03 concentrations (14 to 1100 nM, synthesized as described above). Cells were lysed after 12 or 24 h of treatment using 1X Pierce RIPA buffer (Thermo Fisher Scientific) supplemented with Halt Protease and Phosphatase inhibitor cocktail (Thermo Fisher Scientific). Protein concentrations were determined using the Pierce BCA Protein Assay Kit (Thermo Fisher Scientific), and lysates were stored at −80 °C until further use. For Western Blotting, equal amount of proteins were separated on NuPAGE 4-12% Bis-Tris protein gels (Invitrogen) and transferred to PDVF membranes (Thermo Fisher Scientific) using Power Blotter system (Invitrogen). SeeBlue (Invitrogen) and Magic Marker XP (Invitrogen) were used as protein standards. Membranes were then blocked in 5% nonfat dry milk (Cell Signaling Technologies) for 1 h at RT, followed by overnight incubation at 4 °C with primary antibodies against Bcl-xL (#2764, Cell Signaling Technologies) and Vinculin (700062, Invitrogen) or beta-tubulin (#86298, Cell Signaling), used as loading control. After washing, membranes were incubated for 1 h at RT with species-appropriate horseradish peroxidase (HRP) conjugated secondary antibodies (donkey anti-rabbit or anti-mouse). Protein detection was performed using SuperSignal chemiluminescent substrate (Thermo Fisher Scientific); images were acquired using the Amersham Imager 600 (GE Healthcare). Band intensities were quantified using ImageJ (v1.53) software. Details of the antibodies used and corresponding experimental conditions are provided in Supplementary Table 9, full gels are provided in Supplementary Fig. 9.

### Immunofluorescent staining

Immunofluorescent (IF) staining was used to assess target protein expression in OV030 models. Cells were seeded into 384-well plates (Revvity) containing pre-spotted drugs when applicable. Sample preparation, staining, and imaging were performed as described earlier^26^. Antibodies and their respective concentrations used in the IF assay are listed in Supplementary Table 9. In addition, cells were stained with phalloidin-647 (1:300, Invitrogen) to enable acquisition of cytoskeleton of all cells.

### Ex vivo drug testing

Biobanked patient materials were used to validate effects of chosen drugs and their combinations. Cell processing and spheroid treatment were performed as indicated before^26,30^. Clinical information describing the characteristics of the patient material is provided in Supplementary Table 2.

### Model characterization using flow cytometry

OV030 patient models: OV030-R and OV030-S, were used for surface marker characterization by flow cytometry assay. Cells were plated in 6-well plates at density of 0.62 × 10^6^ cells per well with 0.01% DMSO. After three days in culture, cells were detached and dissociated with TrypLE. Further, samples were blocked, stained, and acquired as previously described^26^.

### Drug synergy assays

For two or three drug combinations in 2D assays, a selection of PDCs and CCLs was used following DST protocol as described above. In two-drug combinations, A-1331852 was tested at five concentrations ranging from 0.1 to 1,000 nM (10-fold dilution). **γ**-secretase inhibitors: MK-0752 (MedchemExpress), Nirogacestat (MedchemExpress), and PROTAC CBK03 were tested at concentrations of 0.1, 1, 10, 100, 1000, 10,000, 20,000 nM. For three-drug combinations, A-1331852 was tested at 1000, 100 nM concentrations, Nirogacestat and CBK03 at 10,000 and 20,000 nM and Carboplatin (Sigma-Aldrich) at 10,000 nM.

### Long-term treatment recovery assay

Cells OV030-R, OV030-S and OV039-PDF were seeded into black ULA U-bottom 384-well plate (Corning) to allow spheroid formation. After two days, spheroids were treated with a single (A-1331852, CBK03, Nirogacestat, and Carboplatin) and combination drug treatment. Concentrations for each drug used in assays: for A-1331852: 1,000, 100, 10 nM, for CBK03: 20, 10, 1 mM, for Nirogacestat: 20, 10 mM, for Carboplatin: 10 mM. Drugs were pre-spotted into 384-well plates (Greiner) and dissolved in Rockit media containing live-cell dyes: TMRM (Thermo Fisher Scientific) and Sytox-Green (Invitrogen). The drug containing media was then transferred to the spheroid plates. Spheroids were treated for five days with addition of drugs after three days. At the end of the 5-day treatment period, spheroids were washed three times with the corresponding culture media. Media changes were performed every 2-3 days throughout a 15-day assay. All liquid handling steps, including drug addition, media changes, and washes were carried out using the Apricot^®^ S3 (SPT LabTech) liquid handling system.

### Imaging and image analysis

All imaging was performed using the OperaPhenix high-content screening system (Revvity). Live-dead images of long-term treatment recovery and ex vivo cultures were acquired at 10X, capturing one field of view (FOV) with up to 20 z-stack planes at 0 and 72 h after treatment. IF stained cells were imaged at 20X using 9 FOV with 3% overlap at 24 or 72 h of culture. For HES1 staining, images were acquired using a 40X water immersion objective, capturing 49 FOV with 3% overlap at both 24 and 72 h.

Image analysis was conducted using Harmony software v5.2 (Revvity). Analysis of long-term recovery treatment and ex vivo spheroids followed analysis pipeline described previously^26^, using spheroid volume as the primary output and additional parameters for morphological comparison. For IF images stained for ALDH1A1, CD44, CK8/18, FSP1, MUC16, VIM, and α-SMA, all channels were first pre-processed using Gaussian smoothing. Nuclei were identified using detection method “B”; cytoplasm and cell masks were segmented using methods “D” and “P”, respectively. Marker positivity was quantified as the proportion of marker-positive cells relative to total cells identified by phalloidin staining. For nuclear markers, PAX8, HES1 the percentage of marker-positive nuclei were calculated relative to the total number of Hoechst-stained (Invitrogen) nuclei.

### Data analysis and statistics

All data analysis and visualizations were performed using R v4.5.0. DSS from single drugs and combination screens were calculated using Breeze web-based tool (FIMM, Finland), applying DSS2 method and four-parametric (4-PL) curve fitting^98,99^. Assay quality was assessed using Z’ factor calculations for each experiment (Supplementary Table 10). Drug synergy was quantified using the “synergyfinder” v3.16.0 R package. Growth rate correction was calculated based on previously published methods^36^. Flow cytometry data was analyzed using FlowJo v10.6.0 software (BD Biosciences). Fluorescent minus one (FMO) controls were used to correct for spectral overlap, while isotype controls were included to assess nonspecific binding. Differential proteomics abundance analysis from MS-based proteomics data was conducted using the “DEqMS” v1.26.0 R package. Fold change values derived from comparison between two untreated OV030 models, or between untreated and A-1331852 treated, were used for gene set enrichment analysis (GSEA) using the “clusterProfiler” v4.16.0 R package. Visualizations of the CNV were done with “GenVisR” v.1.39.0 R package. Statistical tests used for comparison between two or more groups are detailed in the figure legends. Statistical significance was defined as p < 0.05 and is indicated in the text where applicable.

## Supplementary information

Supplementary Data

## Data Availability

The datasets generated and analyzed during the current study are not publicly available due to data protection restrictions associated with patient-derived genomic data, but are anonymized data may be available from the corresponding author on reasonable request. All drug testing data generated during this study are included in this article and its Supplementary Information.
