# Supplementary material for: Functional profiling of ovarian cancer models reveals Bcl-xL/NOTCH targeting to overcome resistance
Source: NPJ Precis Oncol. 2026 Aug 4;10:301. doi: 10.1038/s41698-026-01628-2 (PMC13438132; doi:10.1038/s41698-026-01628-2)
Supplement: Supplementary file 1 [file 41698_2026_1628_MOESM1_ESM.pdf]

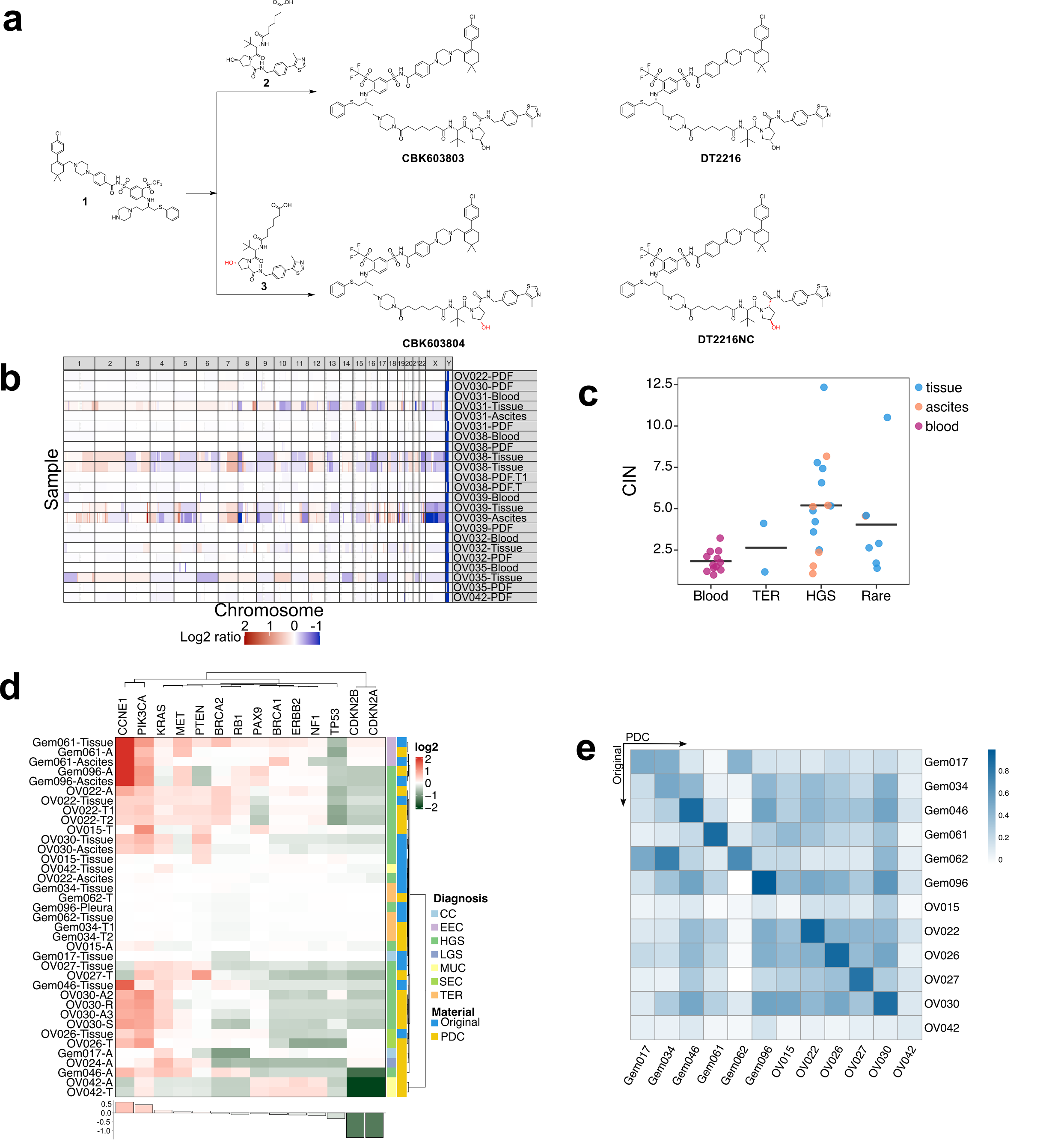

Supplementary Figure 1. a – Schematic overview of synthesis of PROTAC targeting Bcl-xL, showing key intermediate products as well as structural comparison between in-house synthesized PROTACS and the commercially available DT2216. **b** – CNV profile of original patient tumor/blood/ascites tissue and the PDFs used in this study. **c** – Distribution of CIN in tissue, ascites and blood samples from patients used for PDM development. **d** – Heatmap displaying CNVs in OC-associated genes across all original samples and their corresponding PDMs. A bar plot below the heatmap represents the average log2 CNV ratio for each gene, summarizing CNV magnitude across the cohort. **e** – Similarity matrix comparing all PDCs to their respective original tumor samples, based on CNV profiles. The matrix illustrated the degree of genomic concordance between each PDC and its source material.

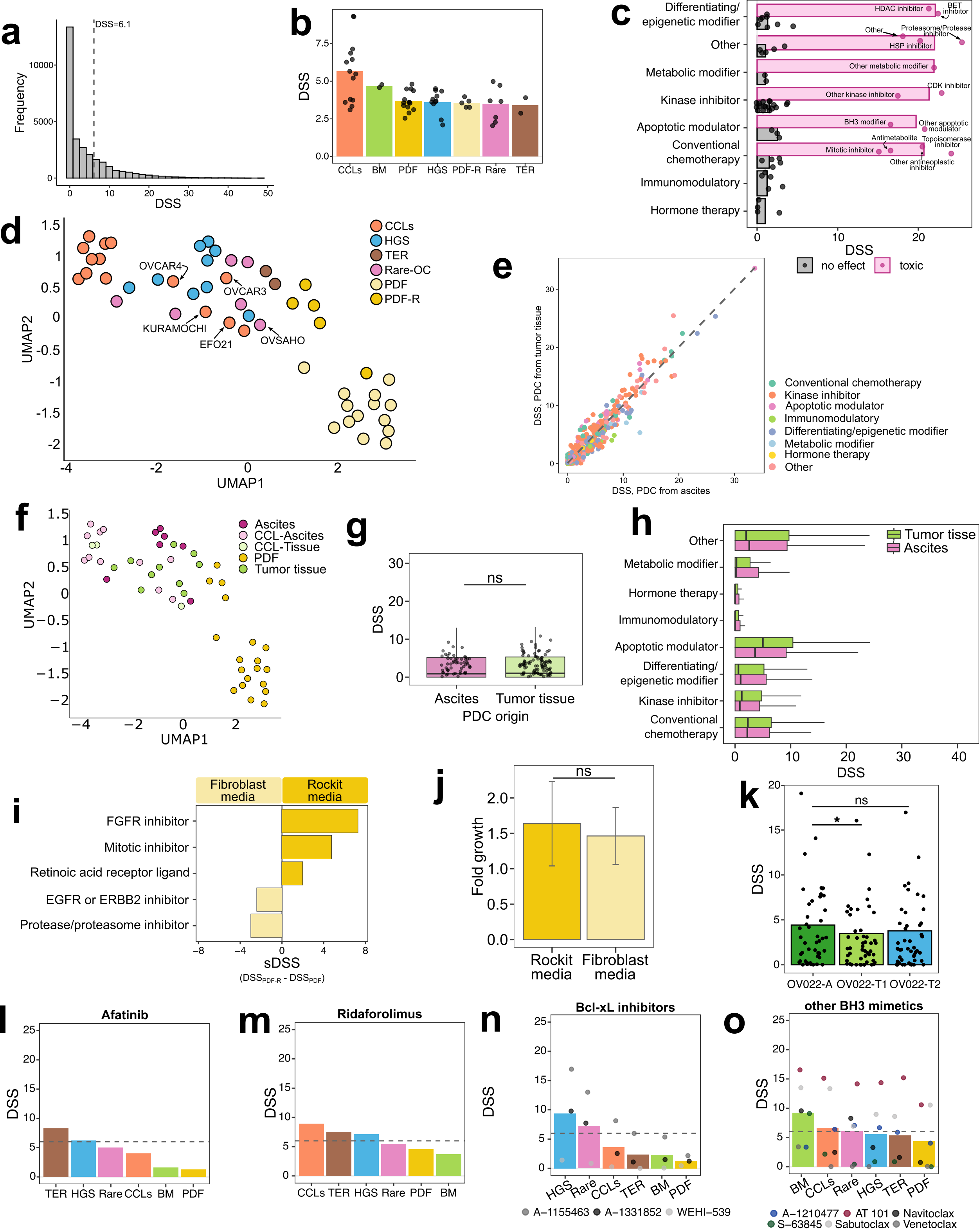

Supplementary Figure 2. a – Distribution of DSS values across screened CCLs, PDMs, BMs, the dashed line represents 75th quantile with representative DSS value. **b** – Summary of drug sensitivity across all tested samples, grouped by model type. Each dot represents the mean response of a drug subclass. **c** – Drug classes with indicated MoAs representing drugs with highest toxicity to BM or showing no effect. **d** – UMAP visualization of drug sensitivity profiles, each dot corresponding to a PDM or a CCL. Arrows indicate CCLs that cluster most closely with the PDMs. **e** – Comparison of DSS between PDCs derived from ascites and tumor tissue. Each dot represents individual drug colored by class, dashed line marks the linear trend. **f** – UMAP visualization of drug sensitivity profiles, each dot corresponding a PDM or a CCL, here unlike in Sup. Fig, 2D it is colored by the ascites or tissue origin for PDCs or CCLs. **g** – Boxplot comparing the distribution of DSS in ascites- and tumor tissue- derived PDCs. Each dot represents the DSS value of specific drug class in an individual PDC. **h** – Ascites- and tumor tissue- derived PDCs drug response comparison for each of the drug class. **i** – Effect of culture media on drug response in PDFs. **j** – Fold growth in growth of PDFs cultured under different media conditions, measured over 72h period, *p<0.05 from Mann-Whitney U test. **k** – Response of OV022 PDCs to all screened drugs, presented as DSS values, with each dot representing unique MoA, *p<0.05 from Mann-Whitney U test. DSS overview for Afatinib (**l**) and Ridaforolimus (**m**), indicating the average drug response per group. DSS overview for Bcl-xL inhibitors (**n**) and other BH3 mimetics (**o**), shown as average per group. Dots represent individual drug DSS score averaged per group. In l, m, n, o – dashed line denote DSS=6.

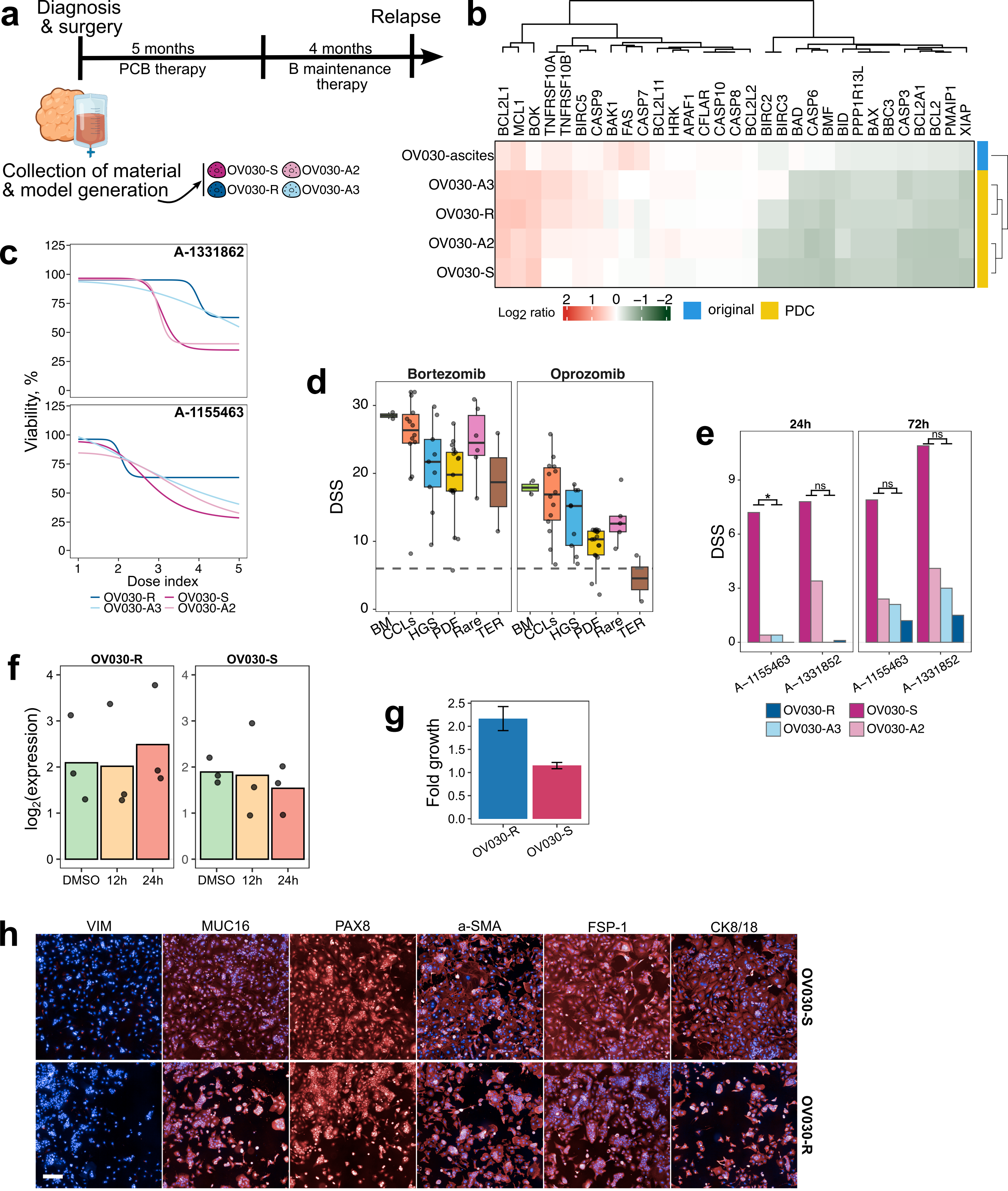

Supplementary Figure 3. a – overview of OV030 patient clinical timeline. Here P indicates Paclitaxel, C – Carboplatin and B – Bevacizumab. **b** – CNV profile of apoptosis-related genes in OV030 ascites cells and OV030 PDCs. **c** – Drug response curves of four OV030 patient PDCs with Bcl-xL inhibitors A-1331852 and A-1155463, the x-axis represents index-scaled concentrations, ranging from 0.1-1 uM for A-1331852 and 1-10 uM for A-1155463. **d** – DSS values for two protease/proteasome inhibitors across all tested cell models. Dashed lines denote DSS=6. **e** – Bar plot representing DSS values for A-1331852 across all OV030 PDCs after 24 and 72 h of treatment. *p<0.05 from Mann-Whitney U test (n=2). These results guided the selection of A-1331852 concentration for subsequent MS-proteomics analysis. **f** – Protein expression profiles in OV030-R and OV030-S models with and without A-1331852 treatment, shown as log2 fold change normalized to house-keeping proteins. Each dot represents an independent biological replicate. **g** – Comparison of cell growth between OV030-R and OV030-S after 72 h of culture, error bars indicate mean ± SD. **h** – epresentative IF images illustrating expression of OC and TME-associated markers. Scale bar = 200 um.

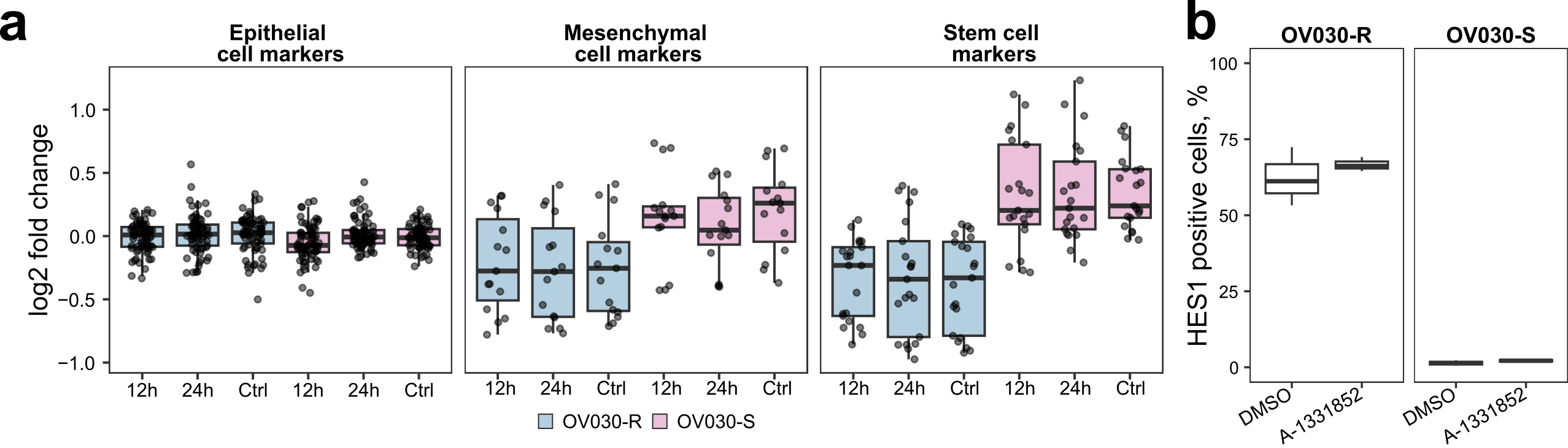

Supplementary Figure 4. a – Expression of EMT signature proteins and CSC-associated markers in OV030-R and OV03-S models, with and without A-1331852 treatment. Expression is shown as log2 fold change. **b** – IF based HES1 quantification of HES1 nuclear expression in OV030-R and OV030-S clones after 24 h in culture with and without A-1331852 treatment.

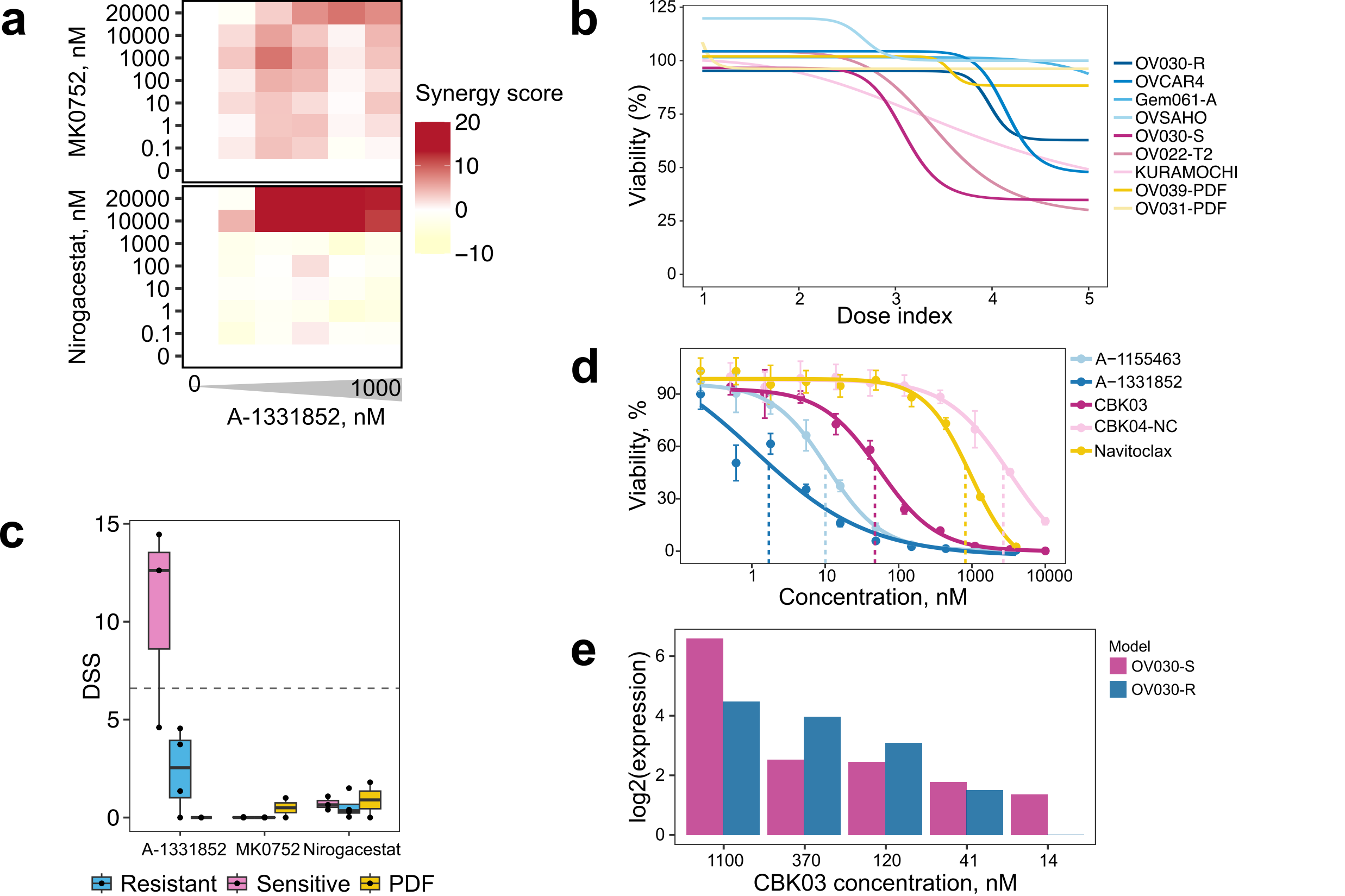

Supplementary Figure 5. a – Synergy score heatmaps for A-1331852 and GSIs combination treatment in the OV030-S model. **b** – Drug response curves following 72h of A-1331852 treatment. Blue curves indicate A-1331852 resistant cancer cell models, pink denote A-1331852 sensitive models, and yellow represent PDFs. **c** – Cell response to monotherapy of drugs used in combination treatment. Dashed lines denote DSS=6. **d** – Drug response curves of MOLT-4 cell treated with Bcl-xL targeting drugs. Dotted lines represent EC50 values, ± SD (n=3 for each condition). **e** – Protein expression profiles in OV030-R and OV030-S models at different CBK03 concentrations, shown as log2 fold change normalized to house-keeping proteins.

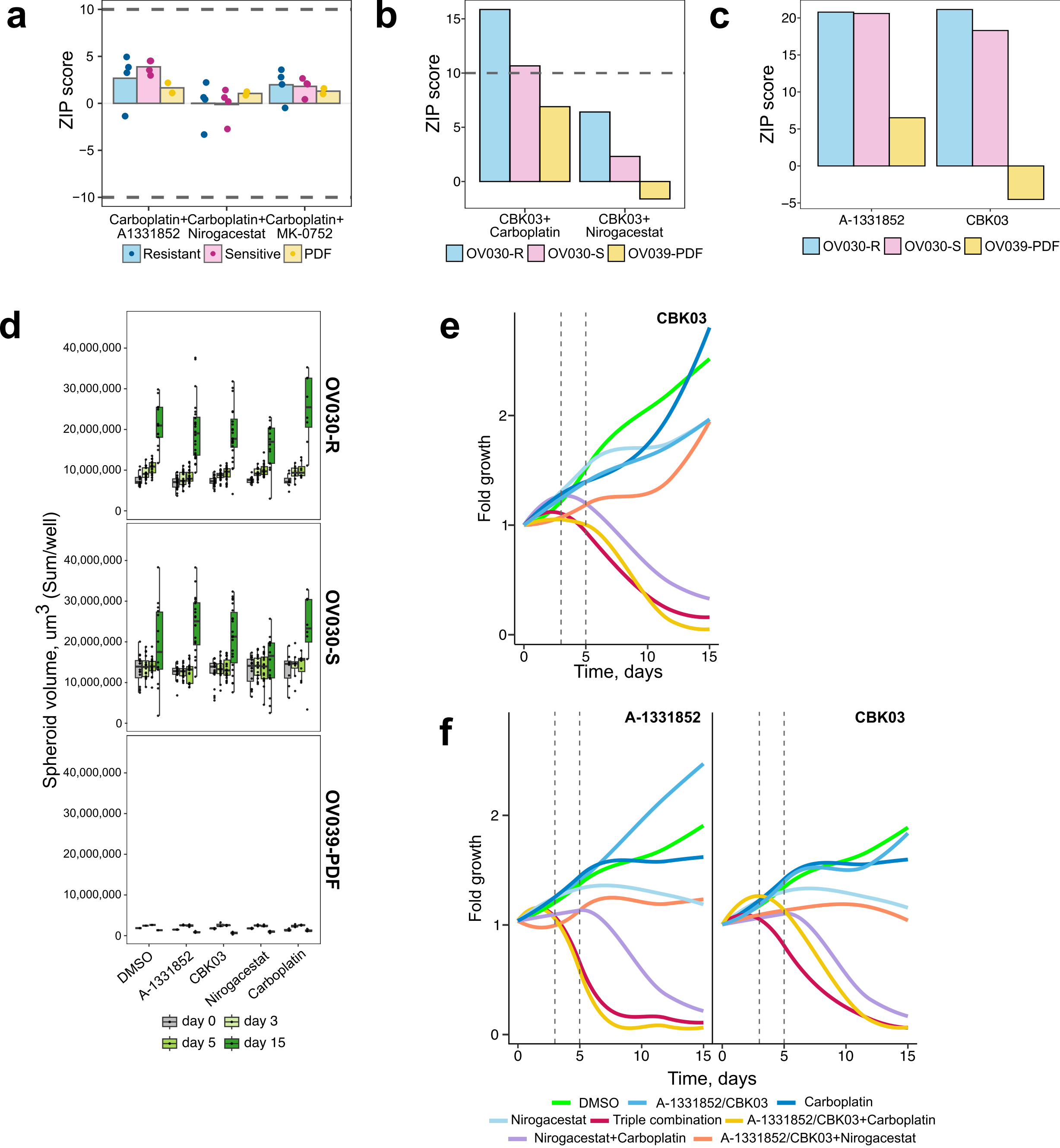

Supplementary Figure 6. a – Synergy score heatmaps for A-1331852 and GSIs combination treatment in the OV030-S model. **b** – ZIP synergy scores visualized as bar plots for CBK03 and GSIs combinations. **c** – ZIP synergy scores displayed as bar plots for A-1331852 and CBK03 based triple drug combinations for OV030-R, OV030-S and OV039-PDF. **d** – Boxplots representing spheroid volume change treated with monotherapy. Colors indicate time point: baseline (before treatment), and at 3, 5 and 15 days after treatment. Here drug exposure lasted for five days, after which treatment was removed. **e** – Longitudinal response of OV030-R to the highest tested concentrations of single and combination CBK03-based treatments across the 15-day assay. **f** – Longitudinal response of OV030-S to the highest tested concentrations of single and combination treatments across the 15-day assay.

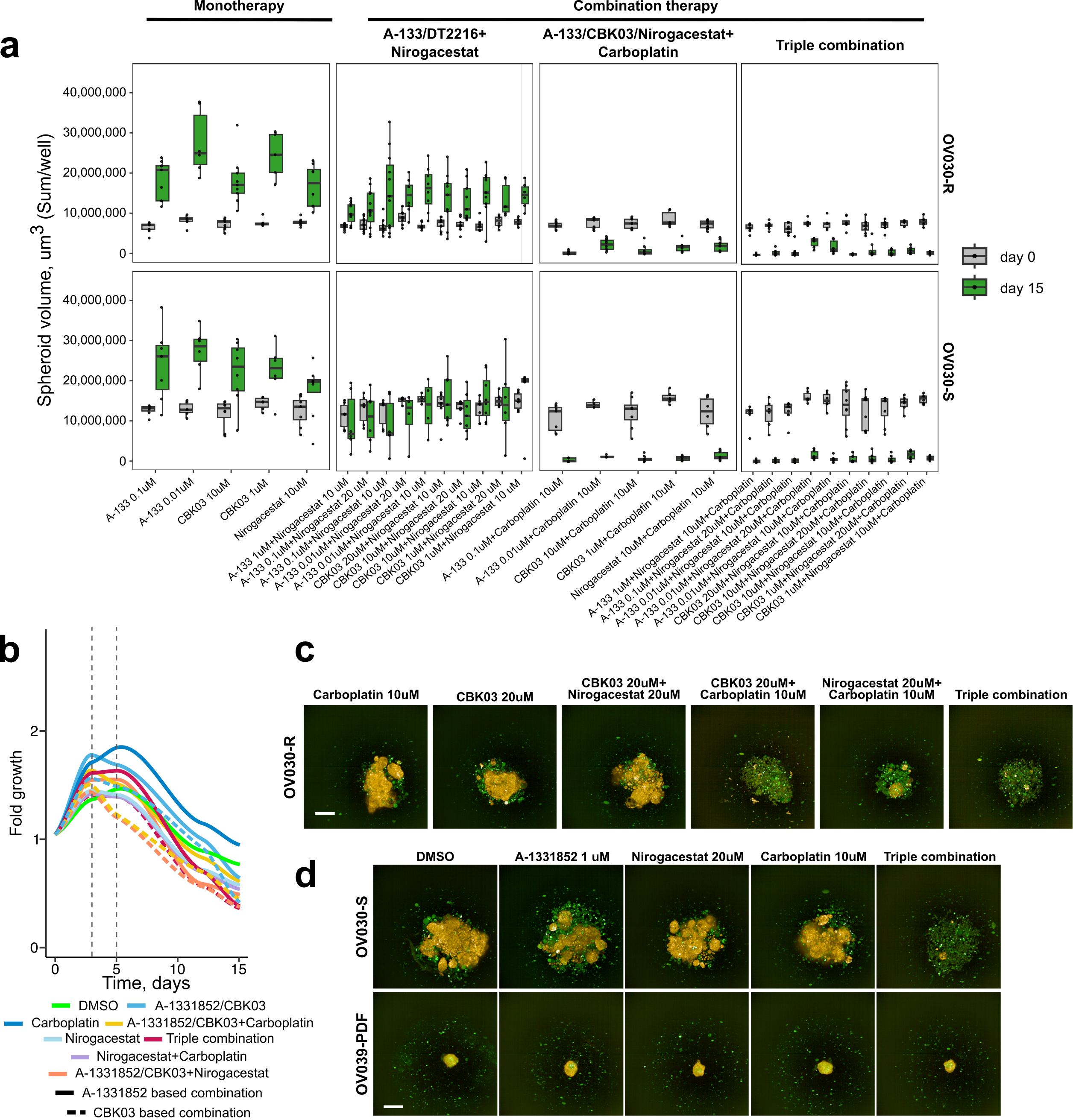

Supplementary Figure 7. a – Boxplots representing drug response of OV030-R and OV030-S models to lower tested concentrations of single and combination treatments, comparing spheroid volume at first and last time point of the assay. **b** – Longitudinal response of OV039-PDF response to the highest tested concentration of single and combination treatments across the 15-day assay. **c, d** – Representative images showing spheroid size and viability afterday 15 of the assay, with and without treatment. Green signal marks dead, yellow indicates live cells, scale bar is 200 um.

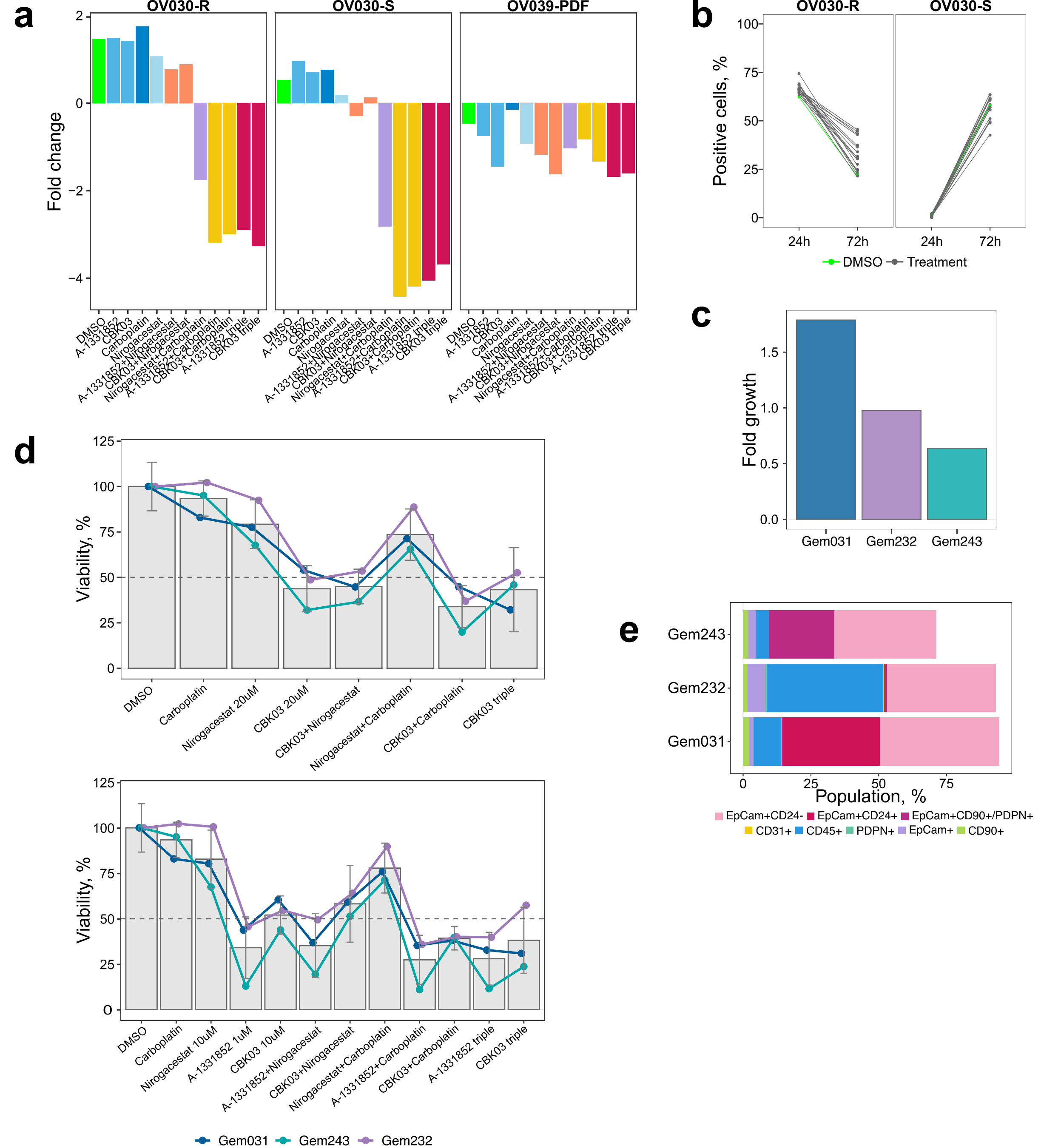

Supplementary Figure 8. a – Overview of the response of OV030-R, OV030-S, and OV039-PDF to monotherapy and combination treatments, represented as changes in spheroid volume between first and last time points of the assay. Bar plots show the average response between all tested concentrations per individual drug. **b** – Percentage of HES1 positive cells with and without treatment, 24 and 72 h in culture. **c** – *Ex vivo* culture fold growth over 7 days from the start of the imaging assay. **d** – *Ex vivo* spheroid viability in response to CBK03 based treatments at highest concentration (left hand-side) and lower tested drug concentrations (right hand-side). Error bars represent mean ± SD (n=3). **e** – Distribution of flow cytometry-based characterization of cell population in *ex vivo* cultures assessed on cell plating day.

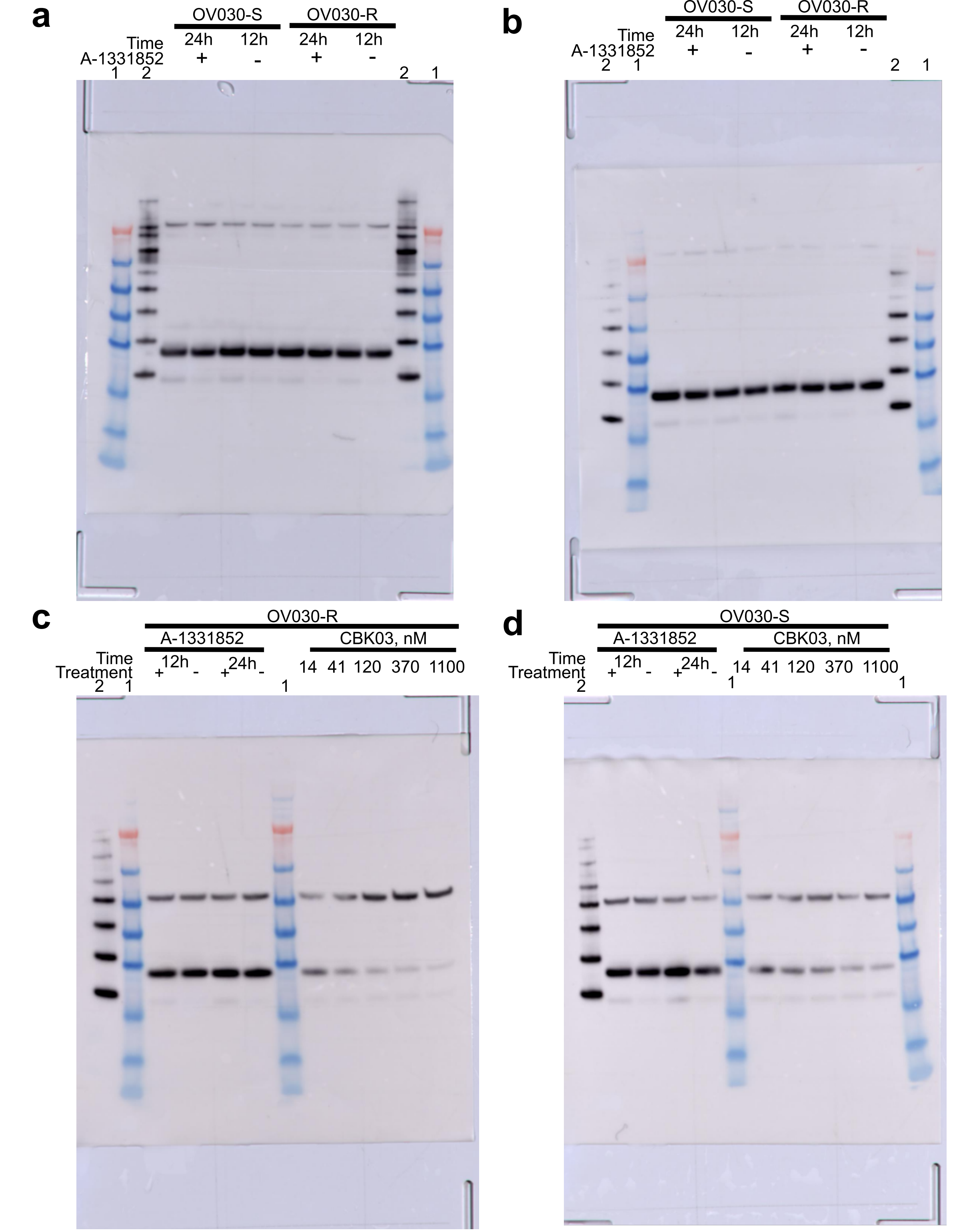

Supplementary Figure 9. Images of full Western blot gels. **a, b** – show OV030 cells treatment with A-1331852, **c, d** – gels contain samples treated with A-1331852 and CBK03. Vinculin was used as loading control for gels a and b, while b-tubulin was used for remaining gels. The images are presented as original, uncropped blots without any image adjustments to brightness, contract, or color. Exposure times were 0.5 s for gels in a and b, and 0.1 s for gels in c and d. Marker lanes are indicated as follows: 1 – SeeBlue, 2 – MagicMarker. Cropped regions from gel a were used in Fig. 3d; while cropped regions from gels c and d were used for Fig. 5e.

Table of content

#Table	Description
Supplemental Table 1	List of all PDMs and their corresponding names
Supplemental Table 2	Clinical information for the patients used to generate PDMs
Supplemental Table 3	Drug sensitivity scores for all PDMs and CCLs based on luminescence signal, represented as DSS
Supplemental Table 4	DSS comparison between ascites and tissue derived PDMs.
Supplemental Table 5	Gene names used to compare EMT and CSC associated protein expression and gamma-secretase complex containing proteins. Provided list contains markers detected in used proteomics dataset
Supplemental Table 6	ZIP synergy scores for all experiments
Supplemental Table 7	A133, Carboplatin and GSIs drug combination viability results, shown as %. Values represent average for three technical replicates
Supplemental Table 8	Passage number of PDMs at the time of drug testing and molecular characterization
Supplemental Table 9	List of antibodies used for IF and Western Blot assays
Supplemental Table 10	Drug sensitivity testing assay quality represented with Z'

Supplemental Table 1

Case ID	ID used in manuscript	PDC origin	PDF origin
Gem017	Gem017	TT	
Gem046	Gem0146	TT	
Gem096	Gem096	A	
Gem034	Gem034	TT	
Gem061	Gem061	A	
Gem062	Gem062	TT	
OV015	OV015	TT	
OV022	OV022	TT/A	
OV024	OV024	A	
OV026	OV026	TT	TT
OV027	OV027	TT	TT
OV030	OV030	A	TT
OV031	OV031		TT
OV032	OV032		TT
OV035	OV035		TT
OV038	OV038		TT
OV039	OV039		TT
OV042	OV042	TT/A	TT
OV044	OV044		TT
OC048	OV048		A
OV050	OV050		TT
OV051	OV051		TT

Abbreviations:
TT - tumor tissue
A - ascites
PDC - patient-derived cancer cells
PDF - patient-derived fibroblast cells

Supplemental Table 2

ID used in manuscript	Diagnosis-histology	Stage	BRCA-mutation	HRD	Ca-125	Operation	NACT-type	Postop treatment	Result	Date end of treatment	tion treatment	Relapse/progression	Date of progression	PFI (months)	BMI	Result2
Gem017	Clear cell OC	3C	No	Negative	85	Radical	Kar/Pak	Kar/Pak	**CR**	4/29/22		Yes	8/22/22	4	16.5	DOD
Gem046	HGS OC	4A	No	Inconclusive	1060	Non-radical	Kar/Pak	Kar/Pak	PR	9/20/22	Bev	Yes	12/1/22	2	24.30	DOD
Gem096	HGS OC	4B	No	Negative	1310	Non-radical	none	Kar/Pak/Bev	PR	5/25/23	Bev	Yes	12/11/23	6	30.33	DOD
Gem034	Growing teratoma syndrome		No	NI	NI	Radical	none	none	**CR**	9/8/22					19.57	
Gem061	Serous endometrial cancer	4B	Unknown	NI	6490	Optimal	none	Kar single	PR	1/30/23		Yes	9/14/23	7	32.88	DOD
Gem062	High grade immature teratoma	1A	NI	NI	40	Radical	none	BEP	**CR**	2/3/23					20.96	
OV015	HGS OC	3C	No	NI	243	Inoperable	none	Kar/Pak/Bev	PR	6/17/19	Bev	Yes	1/27/20	8	23.29	DOD
OV022	HGS OC	3C	No	NI	14500	Optimal	Kar/Pakx3	Kar/Pak/Bev	PR	12/27/19	Bev	Yes	6/8/20	5	21.60	DOD
OV024	LGS OC	4A	NI	NI	473	Optimal	Kar/Pakx3	Kar/Pak/Bev	PR	6/17/20	Bev	Yes	11/13/20	5	18.90	DOD
OV026	Serous endometrioid cancer	4B	NI	NI	2560	Radical	none	Kar/Pak	**CR**	11/26/20		Yes	4/6/21	4	20.03	DOD
OV027	HGS OC	4A	No	NI	2840	Inoperable	none	Kar/Pak/Bev	PR	11/11/20	Bev	Yes	12/28/20	1	28.16	DOD
OV030	HGS OC	3C	No	NI	2960	Inoperable	none	Kar/Pak/Bev	PR	3/25/21	Bev	Yes	8/19/21	4	36.98	DOD
OV031	HGSOC	4A	No	NI	3130	Optimal	Kar/Pakx3	Kar/Pak/Bev	PR	2/8/21	Bev	Yes	11/2/21	7	28.34	DOD
OV032	Clear cell endometrial cancer	4B	No	NI	172	Optimal	none	Kar/Pak	PR	3/29/21		Yes	7/30/21	4	30.27	DOD
OV035	LGS OC	4B	NI	NI	427	Radical	none	Kar/Pak/Bev	**CR**	3/24/21		Yes	11/30/23	32	26.57	
OV038	HGS OC	3C	No	NI	125	Radical	none	Kar/Pak	**CR**	7/23/21					26.60	
OV039	HGS OC	3C	No	NI	103	Inoperable	none	Kar/Pak	PR	7/29/21					27.24	
OV042	Recurrent mucinous OC from 2018	1A (2018)	NI	NI	356	Inoperable	none	Karbo	PD	6/4/21		Yes		0	43.88	DOD
OV044	LGS OC	3C	NI	NI	99	Radical	none	Kar/Lipdox	**CR**	11/5/21		Yes	10/20/22	18 & >12	29.37	
OV048	HGS OC	3C	Yes	NI	120	Radical	Kar/Pak + Kar	Kar single	**CR**	12/2/21	PARP	Yes	12/30/23	24	23.71	
OV050	HGS OC	3C	Yes	Positive	1580	Optimal	none	Kar/Pak/Bev	**CR**	2/22/22	Bev+PARP				24.58	
OV051	HGS OC	3C	No	Negative	107	Radical	Kar + Kar/Pak	Kar/Pak	**CR**	12/29/21	PARP	Yes	4/20/23	15	21.13	DOD
Gem031	HGS OC	4B	No	Negative	4160	Non-radical	none	Kar/Pak	PR	10/7/22	Bev	Yes	7/19/23	9	25.78	
Gem243	HGS OC	3C	No	Positive	7370	Optimal	none	Kar single	PR	7/11/24						
Gem232	HGS OC	4A	No	Positive	283	Non-radical	none	Kar/Pak	PR	9/25/24	Olaparib	Yes	1/3/25	3		

Abbreviations:
OC - ovarian cancer
HGS - high grade serous
LGS - low grade serous
PDF - patient-derived fibroblast cells
NACT - neoadjuvant chemotherapy
Kar - carboplatin
Pak - paclitaxel
Bev - bevacizumab
Lipdox - carboplatin+pegylated liposomal doxorubicin
BEP - bleomycin, etoposide, cisplatin
DOD - dead of disease (OC)
NI - no information

Supplemental Table 3

DRUG_NAME	class	subclass	A2780
1-methyl-D-tryptophan	X. Other	Other	0
4-hydroxytamoxifen	F. Hormone therapy	Antiestrogen	1.9
8-amino-adenosine	A. Conventional chemotherapy	Antimetabolite	23.9
8-chloro-adenosine	A. Conventional chemotherapy	Antimetabolite	21.3
A-1155463	D. Apoptotic modulator	BH3 mimetic	7
A-1210477	D. Apoptotic modulator	BH3 mimetic	8.7
A-1331852	D. Apoptotic modulator	BH3 mimetic	0
A-366	C. Differentiating/epigenetic modifier	Other differentiating/epigenetic modifier	0
A-419259	B. Kinase inhibitor	Multi kinase inhibitor	26.2
ABC294640	B. Kinase inhibitor	Other kinase inhibitor	5.8
Abemaciclib	B. Kinase inhibitor	CDK inhibitor	3.3
Abexinostat	C. Differentiating/epigenetic modifier	HDAC inhibitor	13.1
Abiraterone	F. Hormone therapy	Other hormone therapy	0
ABT-751	A. Conventional chemotherapy	Mitotic inhibitor	14.6
Acalabrutinib	B. Kinase inhibitor	BTK inhibitor	0
Acitretin	C. Differentiating/epigenetic modifier	Retinoic acid receptor ligand	7.2
Afatinib	B. Kinase inhibitor	EGFR or ERBB2 inhibitor	17.6
Afuresertib	B. Kinase inhibitor	AKT inhibitor	8
Aldoxorubicin	A. Conventional chemotherapy	Topoisomerase inhibitor	10
Alectinib	B. Kinase inhibitor	ALK inhibitor	0
Alisertib	B. Kinase inhibitor	Aurora kinase inhibitors	6.2
Allopurinol	A. Conventional chemotherapy	Other antineoplastic	0
Alpelisib	B. Kinase inhibitor	PI3K or MTOR inhibitor	9.9
Altiratinib	B. Kinase inhibitor	Multi kinase inhibitor	4.8
Alvocidib	B. Kinase inhibitor	CDK inhibitor	19.5
Amcasertib	B. Kinase inhibitor	Other kinase inhibitor	3
AMG-232	D. Apoptotic modulator	p53 pathway modulator	0
AMG-337	B. Kinase inhibitor	MET inhibitor	0
AMG-925	B. Kinase inhibitor	Multi kinase inhibitor	0
AMG319	B. Kinase inhibitor	PI3K or MTOR inhibitor	0
Aminoglutethimide	F. Hormone therapy	Aromatase inhibitor	0
Amsacrine	A. Conventional chemotherapy	Topoisomerase inhibitor	10.5
Amuvatinib	B. Kinase inhibitor	Multi kinase inhibitor	0.8
Anagrelide	X. Other	Other	0
Anastrozole	F. Hormone therapy	Aromatase inhibitor	0
Apalutamide	F. Hormone therapy	Antiandrogen	0
Apatinib	B. Kinase inhibitor	VEGFR inhibitor	0
APR-246	D. Apoptotic modulator	p53 pathway modulator	0.6
AR-42	C. Differentiating/epigenetic modifier	HDAC inhibitor	11.1
Arsenic(III) oxide	C. Differentiating/epigenetic modifier	Other differentiating/epigenetic modifier	0
ARV-825	C. Differentiating/epigenetic modifier	BET inhibitor	2.6
Asciminib	B. Kinase inhibitor	BCR-ABL inhibitor	0
ASP3026	B. Kinase inhibitor	ALK inhibitor	8
AT 101	D. Apoptotic modulator	BH3 mimetic	16.8
AT-406	D. Apoptotic modulator	Other apoptotic modulator	0
AT13148	B. Kinase inhibitor	Other kinase inhibitor	7.7
AT7519	B. Kinase inhibitor	CDK inhibitor	14.2
AT9283	B. Kinase inhibitor	Aurora kinase inhibitors	7.2
Atorvastatin	G. Metabolic modifier	HMGCR inhibitor	0.9
Auranofin	A. Conventional chemotherapy	Other antineoplastic	4.9
AVN944	G. Metabolic modifier	Other metabolic modifier	20.4
Axitinib	B. Kinase inhibitor	VEGFR inhibitor	1.1
AZ 3146	B. Kinase inhibitor	Other kinase inhibitor	4
AZ191	B. Kinase inhibitor	Other kinase inhibitor	5.1
Azacitidine	C. Differentiating/epigenetic modifier	DNMT inhibitor	6.6
AZD-1080	B. Kinase inhibitor	Other kinase inhibitor	0
AZD-5363	B. Kinase inhibitor	AKT inhibitor	11.8
AZD-5438	B. Kinase inhibitor	CDK inhibitor	13.1
AZD-6482	B. Kinase inhibitor	PI3K or MTOR inhibitor	0.8
AZD-8186	B. Kinase inhibitor	PI3K or MTOR inhibitor	2.6
AZD0156	B. Kinase inhibitor	Other kinase inhibitor	3.2
AZD1152-HQPA	B. Kinase inhibitor	Aurora kinase inhibitors	1.2
AZD1208	B. Kinase inhibitor	Other kinase inhibitor	0
AZD1480	B. Kinase inhibitor	JAK inhibitor	0.2
AZD1775	B. Kinase inhibitor	Other kinase inhibitor	18
AZD3759	B. Kinase inhibitor	EGFR or ERBB2 inhibitor	6.1
AZD3965	G. Metabolic modifier	Other metabolic modifier	0.2
AZD4547	B. Kinase inhibitor	FGFR inhibitor	0
AZD6738	B. Kinase inhibitor	Other kinase inhibitor	10.1
AZD7545	B. Kinase inhibitor	Other kinase inhibitor	0
AZD7762	B. Kinase inhibitor	Other kinase inhibitor	6.1
AZD8055	B. Kinase inhibitor	PI3K or MTOR inhibitor	29
Bafetinib	B. Kinase inhibitor	BCR-ABL inhibitor	0
Baricitinib	B. Kinase inhibitor	JAK inhibitor	0.6
BAY 87-2243	C. Differentiating/epigenetic modifier	Other differentiating/epigenetic modifier	4.2
BAY-1436032	C. Differentiating/epigenetic modifier	IDH1/IDH2 inhibitor	0.3
BCI	X. Other	Other	20.3
Belinostat	C. Differentiating/epigenetic modifier	HDAC inhibitor	14.5
Bentamapimod	B. Kinase inhibitor	Other kinase inhibitor	2.1
Bexarotene	C. Differentiating/epigenetic modifier	Retinoic acid receptor ligand	10.8
BGB-283	B. Kinase inhibitor	RAF inhibitor	7
BGB324	B. Kinase inhibitor	Other kinase inhibitor	3.4
BI 2536	B. Kinase inhibitor	Other kinase inhibitor	21.3
Bicalutamide	F. Hormone therapy	Antiandrogen	0
BIIB021	X. Other	HSP inhibitor	23.6
Bimatoprost	E. Immunomodulatory	Other immunomodulatory	0
Binimetinib	B. Kinase inhibitor	MEK1/2 inhibitor	11.1
Birabresib	C. Differentiating/epigenetic modifier	BET inhibitor	6.1
Birinapant	D. Apoptotic modulator	Other apoptotic modulator	0.4
Bleomycin	A. Conventional chemotherapy	Other antineoplastic	6.1
BMS-754807	B. Kinase inhibitor	Other kinase inhibitor	10.4
BMS-777607	B. Kinase inhibitor	MET inhibitor	0
BMS-911543	B. Kinase inhibitor	JAK inhibitor	1
BMS863233	B. Kinase inhibitor	Other kinase inhibitor	5
Bortezomib	X. Other	Protease/proteasome inhibitor	25.1
Bosutinib	B. Kinase inhibitor	BCR-ABL inhibitor	15.9
BRD7116	X. Other	Other	2.1
Brigatinib	B. Kinase inhibitor	ALK inhibitor	6.3
Brivanib	B. Kinase inhibitor	Multi kinase inhibitor	0
Bryostatin 1	B. Kinase inhibitor	Other kinase inhibitor	0.3
Buparlisib	B. Kinase inhibitor	PI3K or MTOR inhibitor	15.6
BX-912	B. Kinase inhibitor	Other kinase inhibitor	1.8
C646	C. Differentiating/epigenetic modifier	Other differentiating/epigenetic modifier	3.7
Cabazitaxel	A. Conventional chemotherapy	Mitotic inhibitor	31.8
Cabozantinib	B. Kinase inhibitor	Multi kinase inhibitor	0
Canertinib	B. Kinase inhibitor	EGFR or ERBB2 inhibitor	23.8
Capecitabine	A. Conventional chemotherapy	Antimetabolite	0
Capmatinib	B. Kinase inhibitor	MET inhibitor	0.1
Carboplatin	A. Conventional chemotherapy	Other antineoplastic	6.2
Carfilzomib	X. Other	Protease/proteasome inhibitor	26.7
CC-115	B. Kinase inhibitor	PI3K or MTOR inhibitor	14.8
CC-223	B. Kinase inhibitor	PI3K or MTOR inhibitor	8.3
CC122	E. Immunomodulatory	Other immunomodulatory	0
CCT196969	B. Kinase inhibitor	RAF inhibitor	9.2
Cediranib	B. Kinase inhibitor	Multi kinase inhibitor	0
Celecoxib	X. Other	Other	0
CEP-32496	B. Kinase inhibitor	RAF inhibitor	3.7
CEP-37440	B. Kinase inhibitor	ALK inhibitor	9.8
Cerdulatinib	B. Kinase inhibitor	JAK inhibitor	0
Ceritinib	B. Kinase inhibitor	ALK inhibitor	3.2
Chloroquine	A. Conventional chemotherapy	Other antineoplastic	8.1
Cilengitide	X. Other	Other	0
Cisplatin	A. Conventional chemotherapy	Other antineoplastic	17.7
Cladribine	A. Conventional chemotherapy	Antimetabolite	1.2
Clofarabine	A. Conventional chemotherapy	Antimetabolite	9.8
Clomifene	F. Hormone therapy	Antiestrogen	5
Cobimetinib	B. Kinase inhibitor	MEK1/2 inhibitor	16.1
Copanlisib	B. Kinase inhibitor	PI3K or MTOR inhibitor	24.6
CPI-0610	C. Differentiating/epigenetic modifier	BET inhibitor	6.1
CPI-360	C. Differentiating/epigenetic modifier	HMT inhibitor	0
CPI-613	G. Metabolic modifier	Other metabolic modifier	0
Crenolanib	B. Kinase inhibitor	FLT3 inhibitor	5.4
Crizotinib	B. Kinase inhibitor	ALK inhibitor	0
CUDC-305	X. Other	HSP inhibitor	16.3
CUDC-907	C. Differentiating/epigenetic modifier	HDAC inhibitor	34.9
Cytarabine	A. Conventional chemotherapy	Antimetabolite	5.5
Cytarabine/Idarubicin	A. Conventional chemotherapy	Other antineoplastic	9.1
Dabrafenib	B. Kinase inhibitor	RAF inhibitor	0.5
Dacomitinib	B. Kinase inhibitor	EGFR or ERBB2 inhibitor	18.8
Dactinomycin	A. Conventional chemotherapy	Other antineoplastic	34.7
Dactolisib	B. Kinase inhibitor	PI3K or MTOR inhibitor	20.3
Danusertib	B. Kinase inhibitor	Aurora kinase inhibitors	12.7
Daporinad	G. Metabolic modifier	Other metabolic modifier	30.7
Darapladib	X. Other	Other	0.8
Dasatinib	B. Kinase inhibitor	BCR-ABL inhibitor	19.1
Daunorubicin	A. Conventional chemotherapy	Topoisomerase inhibitor	11.1
Decernotinib	B. Kinase inhibitor	JAK inhibitor	0
Decitabine	C. Differentiating/epigenetic modifier	DNMT inhibitor	1.1
Deferoxamine	X. Other	Other	4.1
DEL-22379	B. Kinase inhibitor	Other kinase inhibitor	10.2
Dexamethasone	E. Immunomodulatory	Glucocorticoid	0

DRUG_NAME	BM-1690	BM-1691	EFO21	Gem017-T	Gem046-T	Gem034-T1	Gem061-A	Gem062-T	KURAMOCHI	MH	NCI-ADR-RES	OAW28	ONCODG-1	OV015-T	OV022-A	OV022-T1	OV026-T	OV026-PDF	OV027-T	OV027-PDF	OV027-PDF-R	OV030-R	OV030-S	OV030-A3	OV030-A2	OV030-PDF	OV030-PDF-R	OV031-PDF	OV031-PDF-R	OV032-PDF	OV039-PDF	OV039-PDF-R	OV042-A	OV042-T	OV042-PDF	OV042-T2	OV044-PDF	OV048-PDF	OV048-PDF-R	OV048-PDF.T	OV050-PDF	OV051-PDF	OVCAR3	OVCAR4	OVCAR8	OVSAHO	OVTOKO	Gem096	TYKNU	CAOV3	OV024-	OV035-P	OV038-P	OV022-T2	COV362
1-methyl-D-tryptophan	0	3.1	0	0	0	0	0	0	0	0.3	0	0	0		0	0	0	0	0	0	0	0	0	0	0.1	0	0	0	0	0	0	0	0	0	0	0	0	0	0	0	0.3	0	0	0	0	0	0	4.4	0	0.05	0	0.7	0.0	0	0
4-hydroxytamoxifen	5.3	4.7	0.6		0	0	2.2	0.6	2.1	6.6	4.1	3.3	4.9	3.3	4.3	5.1	5	2.8	3.3	1.1	1.6	6.3	4.9	4.1	4	1.7	4.9	0.6	5.5	2.1	3.6	5.1	0.7	0	4.4	7.4	1.9	1.1	3.5	2.4	1.5	2.2	5.5	3.3	4.1	4.2	7	6.3	3.6	2.45	3.7	2.2	3.2	5	1
8-amino-adenosine	13	11.6	21.9		20.3	13.8	19.1	20.4	20.1	24.1	23.6	22.6	22.8		18.9	16.6	19.4	21.2	20.6	15.2	13.5	21.3	18.5	18.5	19.4	19.9	18.4	17.4	17.5	20.9	16.9	18.2	16.1	17.5	18.1	21.4	13.7	17.4	15.1	10.6	15	14.6	21.2	19.6	22.2	20.5	26.2	12.8	24.1	23.2	23.95	20.4	16.9	16.1	21.6
8-chloro-adenosine	10.4	8.9	8.9		4.2	2	6.2	4.4	10	20.8	22	11.2	14.1		6.4	5.7	8.9	11.3	10.1	1.5	7.3	8.3	7.9	6.1	8.4	7.5	2.1	4.3	1.8	8.8	9.4	4.8	4	3.5	6.2	10.9	3.8	6.5	0.1	3.6	4.3	4	12.5	11.5	18.3	5.1	18.1	3.9	20.9	14	13.45	8.1	6.6	7	15.3
A-1155463	0	10.7	4.8		2.1	6.8	0.9	5.1	18.6	2.7	5.7	21.6	6.9		24.2	15.3	7.7	3.1	19	0	0	13.1	19.6	14.7	16.7	0	0	4.7	0.4	3.5	0	0	9.1	10.7	0	29.7	0	19.8	0	1	0.4	4.9	6.1	5.5	4.3	4.1	1.8		8.1	16.55	20.1	1.7	1.7	27.7	1.1
A-1210477	6.7	0	5.6		7.2	6.3	7.9	5.5	7.8	12.3	2.4	5.3	8.2		5.4	5.6	6.9	1.2	8.9	4.6	4.2	6.2	7.1	7.4	6.5	2.9	5.6	4	6.7	2.5	4.8	6.9	5.6	5.6	5.5	6.4	4.2	1.5	7.6	3.4	2.3	2.8	5.4	3.4	6.3	4.8	7.3		6.9	5.25	9.95	2.35	3.5	5.7	3.1
A-1331852	3	0	0.6		1	1	0	1.2	9.4	0	3	1.8	3.3		12	7.7	4.6	8.9	9.5	0.4	1.7	4.4	15.3	7.4	14.9	0	0	0	0	2.1	0.4	0	5.2	6.6	0	15.8	0	4.8	0	0.2	0	1.2	3.1	4.6	1.1	0	0		2.4	6.3	14.15	2.7	0.9	15.7	0.3
A-366	0.9	4.4	0.5		0	0	0	0	0.3	2.6	0.3	2.1	1.4		0	0.2	0	0.1	0	0	0.4	0	1.2	2.2	0	1.4	0	0.4	0	1.3	0	0.3	0	0	0	0.1	1	0.8	0	0.5	0	0.5	0.3	0	1.4	0	14.2	0	1.3	2.35	1.65	0.6	0.8	0	1.8
A-419259	4.5	5.2	14.5		9.1	7.6	14.5	9.9	9	9.8	9.3	22.3	22.1		11.8	9.1	7.9	13.6	6.4	13.9	8.2	8.5	7.4	3.8	8.3	16.7	9.4	17.2	10.7	15	10.5	10.8	1.6	2.8	18.7	11.9	15.8	15.2	9.3	13.8	11.4	11.9	15.5	11	9.9	15.6	9.5	5.6	13.3	16.95	8.25	18.15	15.3	8.9	9

Digoxin	X. Other	Other	13.5	[illegible]
Dinaciclib	B. Kinase inhibitor	CDK inhibitor	23.6	[illegible]
Disulfiram(+CuCl2)	G. Metabolic modifier	Other metabolic modifier	2.9	[illegible]
Docetaxel	A. Conventional chemotherapy	Mitotic inhibitor	29.2	[illegible]
Doramapimod	B. Kinase inhibitor	p38MAPK inhibitor	0	[illegible]
Dovitinib	B. Kinase inhibitor	Multi kinase inhibitor	0	[illegible]
Doxorubicin	A. Conventional chemotherapy	Topoisomerase inhibitor	10	[illegible]
Duvelisib	B. Kinase inhibitor	PI3K or MTOR inhibitor	0	[illegible]
E7820	X. Other	Other	0.4	[illegible]
Eltanexor	D. Apoptotic modulator	Other apoptotic modulator	26.3	[illegible]
Enasidenib	C. Differentiating/epigenetic modifier	IDH1/IDH2 inhibitor	0	[illegible]
Encorafenib	B. Kinase inhibitor	RAF inhibitor	0.6	[illegible]
ENMD-2076	B. Kinase inhibitor	Aurora kinase inhibitors	12.9	[illegible]
Ensartinib	B. Kinase inhibitor	ALK inhibitor	0.5	[illegible]
Entinostat	C. Differentiating/epigenetic modifier	HDAC inhibitor	3	[illegible]
Entospletinib	B. Kinase inhibitor	Other kinase inhibitor	0	[illegible]
Entrectinib	B. Kinase inhibitor	Other kinase inhibitor	0.2	[illegible]
Enzalutamide	F. Hormone therapy	Antiandrogen	0	[illegible]
Enzastaurin	B. Kinase inhibitor	Other kinase inhibitor	0.4	[illegible]
Epacadostat	E. Immunomodulatory	Other immunomodulatory	0	[illegible]
Epirubicin	A. Conventional chemotherapy	Topoisomerase inhibitor	9.4	[illegible]
EPZ-5687	C. Differentiating/epigenetic modifier	HMT inhibitor	0	[illegible]
EPZ015666	C. Differentiating/epigenetic modifier	Other differentiating/epigenetic modifier	1.7	[illegible]
EPZ031686	C. Differentiating/epigenetic modifier	Other differentiating/epigenetic modifier	0	[illegible]
Erastin	G. Metabolic modifier	Other metabolic modifier	3.1	[illegible]
Erdafitinib	B. Kinase inhibitor	FGFR inhibitor	0	[illegible]
Eribulin	A. Conventional chemotherapy	Mitotic inhibitor	23.3	[illegible]
Erlotinib	B. Kinase inhibitor	EGFR or ERBB2 inhibitor	14.7	[illegible]
Etoposide	A. Conventional chemotherapy	Topoisomerase inhibitor	8.4	[illegible]
Everolimus	E. Immunomodulatory	Rapalog	5.9	[illegible]
Exemestane	F. Hormone therapy	Aromatase inhibitor	0.4	[illegible]
Fedratinib	B. Kinase inhibitor	JAK inhibitor	7.6	[illegible]
Filanesib	X. Other	Kinesin inhibitor	26.1	[illegible]
Filgotinib	B. Kinase inhibitor	JAK inhibitor	2.4	[illegible]
Finasteride	F. Hormone therapy	Other hormone therapy	0	[illegible]
Fingolimod	E. Immunomodulatory	Other immunomodulatory	8.1	[illegible]
Floxuridine	A. Conventional chemotherapy	Antimetabolite	11.8	[illegible]
Fludarabine	A. Conventional chemotherapy	Antimetabolite	0	[illegible]
Fluorouracil	A. Conventional chemotherapy	Antimetabolite	6.2	[illegible]
Flutamide	F. Hormone therapy	Antiandrogen	0	[illegible]
Foretinib	B. Kinase inhibitor	Multi kinase inhibitor	4.8	[illegible]
Fostamatinib	B. Kinase inhibitor	Other kinase inhibitor	0.1	[illegible]
FRAX486	B. Kinase inhibitor	Other kinase inhibitor	5.5	[illegible]
Fulvestrant	F. Hormone therapy	Antiestrogen	0	[illegible]
Galiellalactone	X. Other	Other	2.7	[illegible]
Galunisertib	B. Kinase inhibitor	Other kinase inhibitor	0	[illegible]
Gandotinib	B. Kinase inhibitor	JAK inhibitor	7.8	[illegible]
Ganetespib	X. Other	HSP inhibitor	20.9	[illegible]
GDC-0084	B. Kinase inhibitor	PI3K or MTOR inhibitor	19.9	[illegible]
GDC-0623	B. Kinase inhibitor	MEK1/2 inhibitor	18.8	[illegible]
GDC-0853	B. Kinase inhibitor	BTK inhibitor	0	[illegible]
GDC-0919	E. Immunomodulatory	Other immunomodulatory	0	[illegible]
Gedatolisib	B. Kinase inhibitor	PI3K or MTOR inhibitor	12	[illegible]
Gefitinib	B. Kinase inhibitor	EGFR or ERBB2 inhibitor	18.3	[illegible]
Gemcitabine	A. Conventional chemotherapy	Antimetabolite	13.2	[illegible]
Gilteritinib	B. Kinase inhibitor	FLT3 inhibitor	3.8	[illegible]
Givinostat	C. Differentiating/epigenetic modifier	HDAC inhibitor	4.9	[illegible]
Glasdegib	X. Other	Hedgehog inhibitor	0	[illegible]
Glesatinib	B. Kinase inhibitor	Multi kinase inhibitor	0	[illegible]
GNE-0877	B. Kinase inhibitor	Other kinase inhibitor	0	[illegible]
GNE-7915	B. Kinase inhibitor	Other kinase inhibitor	0	[illegible]
Golvatinib	B. Kinase inhibitor	Multi kinase inhibitor	0	[illegible]
Goserelin	F. Hormone therapy	Other hormone therapy	0	[illegible]
GSK-1070916	B. Kinase inhibitor	Aurora kinase inhibitors	1.7	[illegible]
GSK-2334470	B. Kinase inhibitor	Other kinase inhibitor	0	[illegible]
GSK-461364	B. Kinase inhibitor	Other kinase inhibitor	30.2	[illegible]
GSK-690693	B. Kinase inhibitor	AKT inhibitor	11.1	[illegible]
GSK-J4	C. Differentiating/epigenetic modifier	Other differentiating/epigenetic modifier	4.7	[illegible]
GSK2256098	B. Kinase inhibitor	Other kinase inhibitor	1.2	[illegible]
GSK2636771	B. Kinase inhibitor	PI3K or MTOR inhibitor	0	[illegible]
GSK2656157	B. Kinase inhibitor	Other kinase inhibitor	0	[illegible]
GSK269962	B. Kinase inhibitor	Other kinase inhibitor	10.2	[illegible]
GSK2801	C. Differentiating/epigenetic modifier	Other differentiating/epigenetic modifier	0	[illegible]
GSK2830371	X. Other	Other	0	[illegible]
GSK2879552	C. Differentiating/epigenetic modifier	Other differentiating/epigenetic modifier	0	[illegible]
GSK343	C. Differentiating/epigenetic modifier	HMT inhibitor	0	[illegible]
GSK650394	B. Kinase inhibitor	Other kinase inhibitor	3.9	[illegible]
GSK923295	X. Other	Kinesin inhibitor	20.1	[illegible]
Hydroxyfasudil	B. Kinase inhibitor	Other kinase inhibitor	1	[illegible]
Hydroxyurea	A. Conventional chemotherapy	Other antineoplastic	3	[illegible]
I-BET151	C. Differentiating/epigenetic modifier	BET inhibitor	6.2	[illegible]
Ibrutinib	B. Kinase inhibitor	BTK inhibitor	11.2	[illegible]
Icotinib	B. Kinase inhibitor	EGFR or ERBB2 inhibitor	10.5	[illegible]
Idarubicin	A. Conventional chemotherapy	Topoisomerase inhibitor	16	[illegible]
Idasanutlin	D. Apoptotic modulator	p53 pathway modulator	2.9	[illegible]
Idelalisib	B. Kinase inhibitor	PI3K or MTOR inhibitor	0.5	[illegible]
Imatinib	B. Kinase inhibitor	BCR-ABL inhibitor	0	[illegible]
Imiquimod	E. Immunomodulatory	TLRs ligand	0.1	[illegible]
Indibulin	A. Conventional chemotherapy	Mitotic inhibitor	6.8	[illegible]
Infigratinib	B. Kinase inhibitor	FGFR inhibitor	0	[illegible]
IOX-1	C. Differentiating/epigenetic modifier	Other differentiating/epigenetic modifier	3	[illegible]
IOX-2	C. Differentiating/epigenetic modifier	Other differentiating/epigenetic modifier	0.2	[illegible]
Ipatasertib	B. Kinase inhibitor	AKT inhibitor	11.1	[illegible]
Itraconazole	X. Other	Other	3.9	[illegible]
Ivosidenib	C. Differentiating/epigenetic modifier	IDH1/IDH2 inhibitor	0.2	[illegible]
Ixabepilone	A. Conventional chemotherapy	Mitotic inhibitor	5.9	[illegible]
Ixazomib	X. Other	Protease/proteasome inhibitor	8.8	[illegible]
JPH203	X. Other	Other	0	[illegible]
JQ1	C. Differentiating/epigenetic modifier	BET inhibitor	9.7	[illegible]
KD025	B. Kinase inhibitor	Other kinase inhibitor	2.6	[illegible]
KU-60019	B. Kinase inhibitor	Other kinase inhibitor	1.9	[illegible]
Lapatinib	B. Kinase inhibitor	EGFR or ERBB2 inhibitor	9.8	[illegible]
Larotrectinib	B. Kinase inhibitor	Other kinase inhibitor	0	[illegible]
Lasofoxifene	F. Hormone therapy	Antiestrogen	0	[illegible]
Lenalidomide	E. Immunomodulatory	Other immunomodulatory	0	[illegible]
Lenvatinib	B. Kinase inhibitor	Multi kinase inhibitor	0	[illegible]
Letrozole	F. Hormone therapy	Aromatase inhibitor	0	[illegible]
Linifanib	B. Kinase inhibitor	Multi kinase inhibitor	0	[illegible]
Linsitinib	B. Kinase inhibitor	Other kinase inhibitor	0	[illegible]
Litronesib	X. Other	Kinesin inhibitor	25.9	[illegible]
Lomeguatrib	C. Differentiating/epigenetic modifier	Other differentiating/epigenetic modifier	0	[illegible]
Lonafarnib	C. Differentiating/epigenetic modifier	Other differentiating/epigenetic modifier	14	[illegible]
Losmapimod	B. Kinase inhibitor	p38MAPK inhibitor	0	[illegible]
Lovastatin	G. Metabolic modifier	HMGCR inhibitor	0	[illegible]
Lucitanib	B. Kinase inhibitor	Multi kinase inhibitor	2	[illegible]
Luminespib	X. Other	HSP inhibitor	23.2	[illegible]
LY-2584702	B. Kinase inhibitor	Other kinase inhibitor	1.2	[illegible]
LY-2874455	B. Kinase inhibitor	FGFR inhibitor	7.6	[illegible]
LY3009120	B. Kinase inhibitor	RAF inhibitor	15.5	[illegible]
LY3023414	B. Kinase inhibitor	PI3K or MTOR inhibitor	14.5	[illegible]
Marimastat	X. Other	Other	1.7	[illegible]
Masitinib	B. Kinase inhibitor	Multi kinase inhibitor	0.7	[illegible]
Megestrol acetate	F. Hormone therapy	Other hormone therapy	0	[illegible]
Mepacrine	X. Other	Other	18.1	[illegible]
Mercaptopurine	A. Conventional chemotherapy	Antimetabolite	7.1	[illegible]
Merestinib	B. Kinase inhibitor	MET inhibitor	0.1	[illegible]
Metformin	G. Metabolic modifier	Other metabolic modifier	0	[illegible]
Methotrexate	A. Conventional chemotherapy	Antimetabolite	27.6	[illegible]
Methylprednisolone	E. Immunomodulatory	Glucocorticoid	0	[illegible]
Midostaurin	B. Kinase inhibitor	Multi kinase inhibitor	6.4	[illegible]
Milciclib	B. Kinase inhibitor	CDK inhibitor	15.4	[illegible]
Miltefosine	X. Other	Other	6.2	[illegible]
Mitomycin C	A. Conventional chemotherapy	Alkylating agent	6.5	[illegible]
Mitotane	A. Conventional chemotherapy	Other antineoplastic	0	[illegible]
Mitoxantrone	A. Conventional chemotherapy	Topoisomerase inhibitor	10.7	[illegible]
Mivebresib	C. Differentiating/epigenetic modifier	BET inhibitor	17.7	[illegible]
MK-0752	X. Other	Other	0.2	[illegible]
MK-2206	B. Kinase inhibitor	AKT inhibitor	2.9	[illegible]
MK-8745	B. Kinase inhibitor	Aurora kinase inhibitors	6.4	[illegible]
MK-8776	B. Kinase inhibitor	Other kinase inhibitor	0	[illegible]
ML323	X. Other	Other	0	[illegible]
ML390	C. Differentiating/epigenetic modifier	Other differentiating/epigenetic modifier	7	[illegible]
Mocetinostat	C. Differentiating/epigenetic modifier	HDAC inhibitor	9.8	[illegible]
Molibresib	C. Differentiating/epigenetic modifier	BET inhibitor	3.9	[illegible]
Momelotinib	B. Kinase inhibitor	JAK inhibitor	5.5	[illegible]
Motesanib	B. Kinase inhibitor	Multi kinase inhibitor	0	[illegible]
Motolimod	E. Immunomodulatory	TLRs ligand	0	[illegible]
MST-312	X. Other	Other	14	[illegible]
Mubritinib	B. Kinase inhibitor	EGFR or ERBB2 inhibitor	2.7	[illegible]
Napabucasin	X. Other	Other	18	[illegible]
Navitoclax	D. Apoptotic modulator	BH3 mimetic	0	[illegible]
Necrostatin 2	D. Apoptotic modulator	Other apoptotic modulator	0	[illegible]
Neflamapimod	B. Kinase inhibitor	p38MAPK inhibitor	1.2	[illegible]
Nelarabine	A. Conventional chemotherapy	Antimetabolite	0	[illegible]

Neratinib	B. Kinase inhibitor	EGFR or ERBB2 inhibitor
Nilotinib	B. Kinase inhibitor	BCR-ABL inhibitor
Nilutamide	F. Hormone therapy	Antiandrogen
Nintedanib	B. Kinase inhibitor	Multi kinase inhibitor
Niraparib	C. Differentiating/epigenetic modifier	PARP inhibitor
NMS-873	X. Other	Other
NVP-AEW541	B. Kinase inhibitor	Other kinase inhibitor
NVP-BGT226	B. Kinase inhibitor	PI3K or MTOR inhibitor
NVP-BHG712	B. Kinase inhibitor	Other kinase inhibitor
NVP-CGM097	D. Apoptotic modulator	p53 pathway modulator
NVP-LCL161	D. Apoptotic modulator	Other apoptotic modulator
NVP-LGK974	X. Other	Other
NVP-RAF265	B. Kinase inhibitor	RAF inhibitor
NVP-SHP099	X. Other	Other
ODM-201	F. Hormone therapy	Antiandrogen
Olaparib	C. Differentiating/epigenetic modifier	PARP inhibitor
Olmutinib	B. Kinase inhibitor	EGFR or ERBB2 inhibitor
Omacetaxine	A. Conventional chemotherapy	Other antineoplastic
Omaveloxolone	X. Other	Other
Omipalisib	B. Kinase inhibitor	PI3K or MTOR inhibitor
Onalespib	X. Other	HSP inhibitor
ONX-0914	X. Other	Protease/proteasome inhibitor
Oprozomib	X. Other	Protease/proteasome inhibitor
Orteronel	F. Hormone therapy	Antiandrogen
Osimertinib	B. Kinase inhibitor	EGFR or ERBB2 inhibitor
OSU-03012	B. Kinase inhibitor	Other kinase inhibitor
OTS-964	B. Kinase inhibitor	Other kinase inhibitor
OTS167	B. Kinase inhibitor	Other kinase inhibitor
Oxaliplatin	A. Conventional chemotherapy	Other antineoplastic
PAC-1	D. Apoptotic modulator	Other apoptotic modulator
Paclitaxel	A. Conventional chemotherapy	Mitotic inhibitor
Pacritinib	B. Kinase inhibitor	JAK inhibitor
Palbociclib	B. Kinase inhibitor	CDK inhibitor
Palomid-529	B. Kinase inhibitor	PI3K or MTOR inhibitor
Panobinostat	C. Differentiating/epigenetic modifier	HDAC inhibitor
Pazopanib	B. Kinase inhibitor	Multi kinase inhibitor
PCI-34051	C. Differentiating/epigenetic modifier	HDAC inhibitor
PD0325901	B. Kinase inhibitor	MEK1/2 inhibitor
Peficitinb	B. Kinase inhibitor	JAK inhibitor
Pemetrexed	A. Conventional chemotherapy	Antimetabolite
Pentostatin	A. Conventional chemotherapy	Antimetabolite
Perifosine	B. Kinase inhibitor	AKT inhibitor
Pevonedistat	G. Metabolic modifier	Other metabolic modifier
Pexidartinib	B. Kinase inhibitor	Multi kinase inhibitor
PF-00477736	B. Kinase inhibitor	Other kinase inhibitor
PF-00562271	B. Kinase inhibitor	Other kinase inhibitor
PF-03758309	B. Kinase inhibitor	Other kinase inhibitor
PF-04708671	B. Kinase inhibitor	Other kinase inhibitor
PF-06463922	B. Kinase inhibitor	ALK inhibitor
PF-3845	X. Other	Other
PF-4800567	B. Kinase inhibitor	Other kinase inhibitor
PF-670462	B. Kinase inhibitor	Other kinase inhibitor
PF06650833	B. Kinase inhibitor	Other kinase inhibitor
PFI-1	C. Differentiating/epigenetic modifier	BET inhibitor
PH-797804	B. Kinase inhibitor	p38MAPK inhibitor
PHA 408	B. Kinase inhibitor	Other kinase inhibitor
Pictilisib	B. Kinase inhibitor	PI3K or MTOR inhibitor
Pilocarpine	X. Other	Other
PIM-447	B. Kinase inhibitor	Other kinase inhibitor
Pinometostat	C. Differentiating/epigenetic modifier	Other differentiating/epigenetic modifier
Pirfenidone	E. Immunomodulatory	Other immunomodulatory
Pixantrone	A. Conventional chemotherapy	Topoisomerase inhibitor
Plerixafor	E. Immunomodulatory	Other immunomodulatory
Plicamycin	A. Conventional chemotherapy	Other antineoplastic
Pomalidomide	E. Immunomodulatory	Other immunomodulatory
Ponatinib	B. Kinase inhibitor	Multi kinase inhibitor
Poziotinib	B. Kinase inhibitor	EGFR or ERBB2 inhibitor
Pracinostat	C. Differentiating/epigenetic modifier	HDAC inhibitor
Pravastatin	G. Metabolic modifier	HMGCR inhibitor
Prednisolone	E. Immunomodulatory	Glucocorticoid
Prexasertib	B. Kinase inhibitor	Other kinase inhibitor
PS-1145	B. Kinase inhibitor	Other kinase inhibitor
PTC-209	C. Differentiating/epigenetic modifier	Other differentiating/epigenetic modifier
Quisinostat	C. Differentiating/epigenetic modifier	HDAC inhibitor
Quizartinib	B. Kinase inhibitor	FLT3 inhibitor
Rabusertib	B. Kinase inhibitor	Other kinase inhibitor
Radotinib	B. Kinase inhibitor	BCR-ABL inhibitor
Ralimetinib	B. Kinase inhibitor	p38MAPK inhibitor
Raloxifene	F. Hormone therapy	Antiestrogen
Raltitrexed	A. Conventional chemotherapy	Antimetabolite
Ravoxertinib	B. Kinase inhibitor	Other kinase inhibitor
Regorafenib	B. Kinase inhibitor	Multi kinase inhibitor
Resatorvid	E. Immunomodulatory	TLRs ligand
Resiquimod	E. Immunomodulatory	TLRs ligand
Resminostat	C. Differentiating/epigenetic modifier	HDAC inhibitor
RGFP966	C. Differentiating/epigenetic modifier	HDAC inhibitor
Ribociclib	B. Kinase inhibitor	CDK inhibitor
Ridaforolimus	E. Immunomodulatory	Rapalog
Rigosertib	X. Other	Other
Ripasudil	B. Kinase inhibitor	Other kinase inhibitor
RO5126766	B. Kinase inhibitor	Other kinase inhibitor
Rociletinib	B. Kinase inhibitor	EGFR or ERBB2 inhibitor
Rocilinostat	C. Differentiating/epigenetic modifier	HDAC inhibitor
Romidepsin	C. Differentiating/epigenetic modifier	HDAC inhibitor
Roxadustat	C. Differentiating/epigenetic modifier	Other differentiating/epigenetic modifier
RSL3	G. Metabolic modifier	Other metabolic modifier
Ruboxistaurin	B. Kinase inhibitor	Other kinase inhibitor
Rucaparib	C. Differentiating/epigenetic modifier	PARP inhibitor
Ruxolitinib	B. Kinase inhibitor	JAK inhibitor
S-63845	D. Apoptotic modulator	BH3 mimetic
Sabutoclax	D. Apoptotic modulator	BH3 mimetic
Salinomycin	X. Other	Other
Sapanisertib	B. Kinase inhibitor	PI3K or MTOR inhibitor
Sapitinib	B. Kinase inhibitor	EGFR or ERBB2 inhibitor
SAR405838	D. Apoptotic modulator	p53 pathway modulator
Saracatinib	B. Kinase inhibitor	Other kinase inhibitor
Saridegib	X. Other	Hedgehog inhibitor
SB 743921	X. Other	Kinesin inhibitor
SCH772984	B. Kinase inhibitor	Other kinase inhibitor
Seliciclib	B. Kinase inhibitor	CDK inhibitor
Selinexor	D. Apoptotic modulator	Other apoptotic modulator
Selonsertib	B. Kinase inhibitor	Other kinase inhibitor
Selumetinib	B. Kinase inhibitor	MEK1/2 inhibitor
Senexin B	B. Kinase inhibitor	CDK inhibitor
Sepantronium bromide	D. Apoptotic modulator	Other apoptotic modulator
Serabelisib	B. Kinase inhibitor	PI3K or MTOR inhibitor
SGC-CBP30	C. Differentiating/epigenetic modifier	Other differentiating/epigenetic modifier
SGC0946	C. Differentiating/epigenetic modifier	HMT inhibitor
SGI-1776	B. Kinase inhibitor	Other kinase inhibitor
SH-4-54	X. Other	Other
Silmitasertib	B. Kinase inhibitor	Other kinase inhibitor
Simvastatin	G. Metabolic modifier	HMGCR inhibitor
Sirolimus	E. Immunomodulatory	Rapalog
Sitravatinib	B. Kinase inhibitor	Multi kinase inhibitor
SN-38	A. Conventional chemotherapy	Topoisomerase inhibitor
SNS-032	B. Kinase inhibitor	CDK inhibitor
Sonidegib	X. Other	Hedgehog inhibitor
Sonolisib	B. Kinase inhibitor	PI3K or MTOR inhibitor
Sorafenib	B. Kinase inhibitor	Multi kinase inhibitor
Sotrastaurin	B. Kinase inhibitor	Other kinase inhibitor
Spebrutinib	B. Kinase inhibitor	BTK inhibitor
StemRegenin 1	C. Differentiating/epigenetic modifier	Other differentiating/epigenetic modifier
Sunitinib	B. Kinase inhibitor	Multi kinase inhibitor
Tacedinaline	C. Differentiating/epigenetic modifier	HDAC inhibitor
Tacrolimus	E. Immunomodulatory	Other
TAK-285	B. Kinase inhibitor	EGFR or ERBB2 inhibitor
TAK-580	B. Kinase inhibitor	RAF inhibitor
TAK-901	B. Kinase inhibitor	Aurora kinase inhibitor
Taladegib	X. Other	Hedgehog inhibitor
Talazoparib	C. Differentiating/epigenetic modifier	PARP inhibitor
Talmapimod	B. Kinase inhibitor	p38MAPK inhibitor
Tamatinib	B. Kinase inhibitor	Other kinase inhibitor
Tamoxifen	F. Hormone therapy	Antiestrogen
Tandutinib	B. Kinase inhibitor	FLT3 inhibitor
Tanzisertib	B. Kinase inhibitor	Other kinase inhibitor
Tarenflurbil	X. Other	Other
Taselisib	B. Kinase inhibitor	PI3K or MTOR inhibitor
Tasquinimod	E. Immunomodulatory	Other immunomodulatory
Tazemetostat	C. Differentiating/epigenetic modifier	HMT inhibitor
Telatinib	B. Kinase inhibitor	Multi kinase inhibitor
Temozolomide	A. Conventional chemotherapy	Alkylating agent
Temsirolimus	E. Immunomodulatory	Rapalog
Teniposide	A. Conventional chemotherapy	Topoisomerase inhibitor
Tepotinib	B. Kinase inhibitor	MET inhibitor

Tesevatinib	B. Kinase inhibitor	EGFR or ERBB2 inhibitor	19.8	1.6	1.5	1.6		3.3	8	9.6	10.8	0.7	1.5	1.7	5.1	17		7.6	6.7	12.8	0.9	1.4	0	1.3	8.7	7	4.5	6.9	0	9.8	0	5.9	0.6	0	9	0	0	0	3.6	0	0	3.7	0	0.8	0	6.3	0	2.1	1.2	1.2	7.7	1	3.95	3.35	0.35	0.4	7.9	0
TEW-7197	B. Kinase inhibitor	Other kinase inhibitor	0	0	0	0		0.1	0.8	0	0.6	0	0	0	1.8	0		0	0	0	0	2.9	0	0	0	0	0	0	0	0	0	3.3	0	0	0	0	0	0	6.4	0	0	0	0	0.8	0	0	1.3	0.3	1.5	0	0	3	0.65	0	0	0.0	0	1.5
TG100-115	B. Kinase inhibitor	PI3K or MTOR inhibitor	0	0.8	1.2	0		0	0.1	0	0.1	0	0.3	0	1.5	3.7		0.1	0.1	0	0	0	0.3	0.8	0	0	0	0	0	0.1	0	0.1	1.1	0.2	0	0	0	0	0	0.2	0.3	0	0	0.8	0.3	0.3	0.2	0	0	1.2	0	0	1.45	0	1.5	1.6	0	0
TGR-1202	B. Kinase inhibitor	PI3K or MTOR inhibitor	0	0.7	1.4	0		0	0.1	0	0	0	0	0	0.8	0		0	0	0.2	0	0	0	16.8	0	0	0	0	0.4	0	0	0	0.2	0	0	0	0.1	0	0	0	0	0	0	0.8	0	1.1	0	0	0	1	0.1	0	0	0.35	0	0.1	0	0
TGX-221	B. Kinase inhibitor	PI3K or MTOR inhibitor	1.6	0.8	0.1	4.6		1.2	1.2	0.3	2.7	3.7	4.6	2.8	6.6	10.8		0.7	2.3	0	0.2	0.7	0	2.2	0	0	0	0.2	1.5	1.2	0.2	2.5	2.7	0	0.9	0	0	0.5	1.9	0	2.4	0.3	0	0.8	3	2.9	2.6	3.4	5.1	4.9	0.4	0.8	5.15	1.2	0.45	0.0	0.5	1.3
TH588	G. Metabolic modifier	Other metabolic modifier	4.7	0	0	1.3		0.3	0	0	0.6	0.5	7.7	9.4	0	6.5		2.9	1.9	2.2	2.6	2.7	0.4	4.9	1	0.5	0.1	2.5	1	0	3.3	2.7	3.7	3.5	0.2	0	0.3	4.9	1.7	0	1.6	1	0	0.8	1.4	6.4	1	3	0.3	7.5		0	3.15	4.2	1.85	4.4	0.2	0.1
Thalidomide	E. Immunomodulatory	Other immunomodulatory	0	0	0	0	0	0	0	0	0	0	0	0	0	0.6		0	0	0	0	0.8	0	0	0	0	0.7	0.6	0.2	0	0	0	0	0	0	0	0	0	0	0	0	0.2	0	0.8	0	23.1	0	0.5	0	2.3	0	0	0.5	0.85	0.25	0.0	0	0
Thioguanine	A. Conventional chemotherapy	Antimetabolite	12	4.2	3.7	2.8	0	0	0	0	0	4	13.4	14.3	5.7	9.4		0	0	0	1.4	0	0.8	0.5	0	0	0	0	3.6	0	3.3	0	3	3	0	0	0	7.1	11.6	2.5	0.9	0	3.6	0.8	2.5	28.2	6.4	12.2	3.2	13.5	0	7.8	4.65	0	2.95	3.7	0	6
THZ2	B. Kinase inhibitor	CDK inhibitor	22.6	28.5	27.5	17.1		15.7	19	16.1	22.6	21.2	29.4	1.6	22.5	25.4		18.6	15.4	13	0	20.5	0	11.6	16.5	18.3	14.9	16.1	21.5	17.3	23.4	20.2	19.3	14.9	18.3	9.2	10.9	23.3	19.7	16.5	17.9	14.9	15	0.8	14.8	26.9	13.4	23.2	13.9	27.1	0	22.3	18.05	24.85	13.95	11.7	18.7	25.5
TIC10	X. Other	Other	6	10.7	12.4	8.1		0.2	2.4	4.1	2.1	0.4	12.3	11.2	2	8.9		2.1	0.3	0.8	0	3.8	4.4	0	0.4	3.6	0.6	2.7	1.6	0.6	2.4	0.5	1	0	0.9	2.2	1.3	1.6	2.3	0.1	0.8	1.3	1.7	0.8	0	8.3	7	10.2	0.9	10.4		8.4	2.35	4.4	2	0.5	0.9	0.1
Tideglusib	B. Kinase inhibitor	Other kinase inhibitor	0	0	0	0		0	0	0	0	0	0	0.9	0	1.1		0.1	0	0.2	0.6	0	0	0	0	0	0	0	0	0	0	0	0.1	0	0	0	0	0	0	0	0.2	0	0	0.8	0	0	0	0	0	0	0	0.2	0	0	0	0.0	0	0.1
Tipifarnib	C. Differentiating/epigenetic modifier	Other differentiating/epigenetic modifier	10.5	8.2	6.9	4.2		1.5	1.9	3	2.7	4	22.8	3.5	4.4	0.8		3.9	2	6	3.5	8.7	6.6	3.6	5.3	11.8	6.5	4.9	7.5	3.7	2.3	8.5	3.1	1.9	7	1.2	0.9	0	6	6.5	1.8	2.7	4.9	0.8	2.7	7.8	6.9	3.9	2.4	23.1	3.8	8.5	6.05	7.8	8.3	3.3	4.9	1.6
Tirabrutinib	B. Kinase inhibitor	BTK inhibitor	4.6	0	0	0		0	0	0	0.1	0	0	0.4	0.8	4.4		0.8	1.8	0.5	0	0.2	0	0.2	1.8	0.9	0	0.1	0	0	0	0	0	0	0.2	0	0	0	0.6	0	0	0	0	0.8	0	3.4	0	0	0.3	0	0	0	0.15	0	0	0.0	0.1	0
Tivantinib	B. Kinase inhibitor	MET inhibitor	3.3	2.4	0.3	0.9		0.3	0.4	0.2	0.2	0.7	4	3.5	4.5	4.9		3.1	1.2	3.5	4.6	4.8	2.4	0	0.7	0	0.6	1	1.9	1.7	3.7	4	3.7	1.9	2.3	0	0	2.5	0.9	1.2	3.4	1.5	2.2	0.8	2.2	6.9	2.5	2.3	0.6	3.8	5.6	3.5	4	3.45	4.05	3.6	0.6	3.6
Tivozanib	B. Kinase inhibitor	VEGFR inhibitor	3.7	1	4.2	0.8	4.9	1	0.7	1.9	3.2	1.3	7.7	5.9	4.1	6.2		0.8	1.4	0.9	6.2	1.9	3.3	4	1.2	5.2	0	2	4	2.2	5.9	4.8	5.6	6.8	3	0.5	0.4	4.3	5.5	4.5	3	3.3	4	0.8	4	3	3.1	4.6	2.4	7.7	2.3	1.1	5.5	2.5	5.55	5.8	0.6	2.2
Tofacitinib	B. Kinase inhibitor	JAK inhibitor	0	7.4	3.1	0	0	0	0	0	0.8	0	0	0	0	0		0	0	0	0	0	0	0	0.6	0	4.9	0	0	0	0	0.1	0	0	0	0.5	0	0	0.1	0	1.4	0	0	0.8	0	0	0.3	0	0	2.6	0.3	0	0	0	0	0.0	0.5	0.3
Topotecan	A. Conventional chemotherapy	Topoisomerase inhibitor	16.4	9.5	5.3	7.7	6.6	2	6.2	6.9	6.3	8.7	26.2	13.2	7.7	11.4	7.9	11.1	9.1	5	2.5	12.8	3.7	3.1	8.3	6.3	6.1	7.8	8.3	8.4	7.2	7.6	5.1	3	12.6	9.3	11.9	3.3	12.8	6.5	4.8	7	4.2	0.8	3.7	16.1	6.1	14.7	3.8	26	8.2	18	20.45	13	4.9	4.6	8.6	6.7
Toremifene	F. Hormone therapy	Antiestrogen	4.1	4.4	2.8	2.2	2	1.5	3.1	6.9	5	3.7	8	5.5	4.1	5.5	2.1	4.9	4.7	5.5	1	3.6	0	1.6	4.6	4.3	6.6	4.4	1.3	5.7	0	3.2	0.5	2.3	5.8	0.6	0.9	3.1	8.1	0.3	1.8	4.9	1.4	0.8	1.6	3.5	0.1	5.2	4.6	6.9	3.9	3.5	2.95	5.2	0.1	1.3	5.9	0
Tosedostat	X. Other	Protease/proteasome inhibitor	3.7	1.6	2.6	1.9		0	2.8	0	6	2.6	8.4	0	1.1	6.5		10.2	6	10.1	2.9	13.3	0	12.3	4.2	7.1	3.7	13.6	2.3	1.5	2.8	3.5	0	0	5.5	2.8	1.3	1.1	17.4	0	0.1	3.6	0	0.8	0.1	11.7	7.1	6.4	9.3	7.6		6.4	10.55	14.2	0.1	0.0	4.2	4.1
Tozasertib	B. Kinase inhibitor	Aurora kinase inhibitors	7	8.7	4.8	2.5		0.5	0	2.1	1.7	0.3	17.5	0	5.6	7.1		3.2	1.2	0	3.9	1.3	3	5.5	0.7	0	0.3	0.6	7.9	2.7	7.2	5.1	7.2	6.8	2.5	3	2.3	8.7	2.6	6.2	5.1	1.6	5.1	0.8	3.9	9.4	5.7	4.3	2.3	18.4	0	6.9	18.6	3.45	8.5	6.8	1.4	3.4
TRAM-34	X. Other	Other	0	0	0	0		0	0	0	0	0	0	0	0	0		0	0	0	0	0	0	0	0	0	0	0	0	0	0	0	0	0	0	0	0	0	0	0	0	0	0	0.8	0	0	0	0	0	0		0	0	0	0	0.0	0	0
Trametinib	B. Kinase inhibitor	MEK1/2 inhibitor	19.8	8.9	8.1	7.4	7	0.9	9.2	8.5	8.5	6.3	17	4.2	10.5	12.7	5.4	2.7	3.9	6.2	1.7	4.7	2.6	2.5	9	2.6	9.2	7.3	8.3	6.9	5.2	0.7	8.4	9.1	5.1	10.7	10.3	4.6	16.6	11.4	6.4	7.7	7.4	0.8	5.8	6	3.5	9	9	18.5	0	19.2	9.6	5.05	9.15	6.3	4	2.8
Tretinoin	C. Differentiating/epigenetic modifier	Retinoic acid receptor ligand	6.7	0	0	0	0	0	0	0	0	0	0	0	0	0		0.6	0	0	1.4	0	2.5	0	0	0	0	1.4	5.1	0	5.2	0	1.9	0	1.4	0	0	0	0	3.6	0.4	0	0	0.8	0	0	0	0	0	0.1	0	0	0	0.85	1.5	1.0	0	0
Triapine	G. Metabolic modifier	Other metabolic modifier	3	2.3	2.3	0.5		0	0.8	0.1	0	1.4	7.5	3.7	0.6	1.6		2.8	0.6	0	0.1	2.3	0	5.8	1.5	0.2	1.3	2.6	0.2	0.8	3.1	1.3	0.5	0.4	0.6	1.9	2.8	1.9	1.1	1.1	0	1.6	0.1	0.8	0	2.3	0	2.6	0	5.9		8.8	3.6	1.7	1.45	0.0	0	1.5
Triciribine	B. Kinase inhibitor	AKT inhibitor	25.5	6.3	13.6	7.6		8.2	12.6	11.8	10	6.3	19.1	19.4	12.5	9.6		14.6	12.3	12.8	16.6	11.7	10	5.1	14.5	11.2	9.6	16.4	12.1	16.5	2.7	11.9	10.9	13.1	14.2	3	5	9.8	17.9	7.2	6.3	11.3	7.2	0.8	5.6	8	16.4	20.1	6.8	23.5	13.8	12.7	12.75	15.1	12.45	10.8	12.3	10.6
Trifluridine	A. Conventional chemotherapy	Antimetabolite	2.4	0	0	0	0	0	0	0	0	1.2	8.2	2.2	0	1.6		0	0	0	0	0.9	0	0.1	0.1	0	0	0.2	0	0	0	0	0	0	0	0	0	0	0	0	0	0	0	0.8	0.1	40.8	0	2.9	0.6	12.8	0	5.4	3.05	0	0	0.0	0	0.6
Tubacin	C. Differentiating/epigenetic modifier	HDAC inhibitor	3	3	0	0		0.3	0	0	0	4	12.9	0	6.3	7		1	2.4	0.9	0.4	3.7	1.5	0	3.4	4.8	1.4	5.5	2.3	0	1.2	0	0.7	0.3	0	0	0	1	0	0	1	0	3.4	0.8	0.5	2.4	3.2	3.7	1.6	13.4	3.8	4.8	3	2.4	1.25	1.8	2.4	0.2
Tubastatin A	C. Differentiating/epigenetic modifier	HDAC inhibitor	0	0	0.5	0.2		0.1	0	0	0.9	1	1.4	0.2	2.5	0		0	0	0	0.3	0.4	3.6	0	4.3	6.3	2.1	0.8	3	1.4	0.2	0	3.5	1.7	2.7	0	0	0.7	0.2	2.6	0	0.9	2.6	0.8	0	0	1.9	2.5	0.3	2.1	0.1	4.1	0.5	0	2.25	2.0	0	0
Tucatinib	B. Kinase inhibitor	EGFR or ERBB2 inhibitor	0	0	0	0		0	0	0	0	0	0	0	0	0		0.2	0	0	0	0	0	0	0	0	0	0	0	0	0	0	0	0	0	0	0	0	0	0	0	0	0	0.8	0	0.2	0	0	0	0	0	0	0	0	0	0.0	0.2	0
Tucidinostat	C. Differentiating/epigenetic modifier	HDAC inhibitor	3	8.1	9.6	1.2		0	1.3	2.4	4.6	5.6	8.8	1.5	3.4	8		8.7	6.6	2.5	2.6	4.4	6.9	1.3	5.6	9	4.6	6.6	6.2	5.8	4.8	6.1	3.8	4.4	8	0.9	0.3	5.7	3.3	3.2	2.2	6.4	5.3	0.8	3.1	6.2	2.4	6.7	0.3	9.2	7.6	4.1	3.25	6.1	4.4	3.5	8.2	0
UCN-01	B. Kinase inhibitor	Other kinase inhibitor	16.1	17.5	17.3	21.8		18.8	13.5	14.4	21.9	13.2	15.3	12.3	18.8	20.5		18.7	14.4	19.4	16.8	12.7	18.2	16.3	19.4	17.9	15.4	19.2	24.6	20.6	26	27.2	23.6	22.2	25.6	14.3	16.9	26.5	23.7	19.6	23.5	25.5	20.8	0.8	20	16.7	14.3	15.2	11	12.8	16.9	24.2	24.3	21.35	22.2	19.9	16.8	18.5
Ulixertinib	B. Kinase inhibitor	Other kinase inhibitor	15.1	5.3	1.9	3.3		1.6	5.3	6.3	5.3	4.7	17.4	4.4	9.2	11.5	3.7	3.7	3.7	4.7	0	5.9	3	2.6	9.8	0.2	4.1	5.6	5.7	6.1	5.9	3.4	7.6	7.4	7	8	4.6	0.9	16.7	9.1	4.1	6	6.6	0.8	4.4	9.6	1.8	6.2	8	17.4	0	13.2	6.25	6.4	6.25	3.9	4.6	2.2
UM729	C. Differentiating/epigenetic modifier	Other differentiating/epigenetic modifier	1.5	0.1	0	0.8		0	0	0.7	0	0	3.5	2.1	0.2	0.6		1.3	0.9	0.2	6.8	0	0	4	0.9	2.1	2.8	0.6	1.3	0	1.4	0	3.5	3.9	0.2	0	0	1.2	0	0.7	0.2	0	0	0.8	0	0.7	0	2.6	0	4.8	0	2.5	0.95	1.2	2.45	2.1	1.2	0.7
UNC0638	C. Differentiating/epigenetic modifier	Other differentiating/epigenetic modifier	4.5	5.4	7.5	4.3		0	0	2.3	0.4	2.8	5.7	0.8	5.1	4.4		0.8	5.4	0.4	4.6	0.5	0	1.6	2.4	4.7	3.2	2.4	6	0.8	5.7	0	6.6	6.7	1.4	0	0	0	2.2	6	5.1	0.1	1.4	0.8	0	2.9	2.7	6.1	1.4	5.5	0.2	6.5	5.9	1.55	6.75	5.9	5.1	2
UNC0642	C. Differentiating/epigenetic modifier	Other differentiating/epigenetic modifier	1.3	5	5.3	1.6	0	0	0	0.1	0	1.4	4.5	0.6	3.2	2.5		1.3	1.4	0	5.3	0	0	2.1	2	1.8	2.2	0.7	5.1	0	4.5	0	4.7	3.3	1.5	0	0	4.2	0.2	0.3	0.6	0	0	0.8	0.1	2.2	0.2	1.5	0.8	3	0	4.5	6.05	1.05	5.95	4.5	3.8	4.4
UNC1215	C. Differentiating/epigenetic modifier	Other differentiating/epigenetic modifier	0	0	0	0		0	0	0	0	0	0	0.1	0	0		0	0	0	1.5	0	0	4	0	0	0	0	0	0	0	0	0	0	0	0	0	0	0	0	0	0	0	0.8	0	0	0	0	0	0	0	0	0	0	0.35	0.0	0	0
UNC2881	B. Kinase inhibitor	Other kinase inhibitor	1.7	0	0	0.3		0	0	0	0	0.3	4.6	0	1.8	2.4		0.1	0	0.1	0.9	0	1.8	1.2	0	0	0	0	2.7	0	2.7	0	1.6	3.8	0	0	0	2.7	0.5	1.3	0	0	1.3	0.8	1.3	0	0	0.1	1.2	2	0	1.2	1.5	0.15	2.7	1.3	0.5	0.7
Upadacitinib	B. Kinase inhibitor	JAK inhibitor	0	6.2	1.3	0	0	0	0	0	0	0	0	0	0	0		0	0	0	0	0	0.8	0	0.2	0	0	0.1	1.4	1.8	0.6	5.2	0.2	0	0	0.2	0	1.9	0	0	0	0.1	0	0.8	0.6	0	0	0	0	2.3	0	0	0	0	0	0.0	0	0
Uprosertib	B. Kinase inhibitor	AKT inhibitor	5	1.1	0	1.2		1	8.2	13.6	5.4	0.3	7.1	0	0.9	3.9		2.5	1.9	2.5	1.8	1.4	0	4.8	6.8	4.4	10.2	8.5	3	1.7	7.7	2	4.5	2.1	0.5	0	0	0.8	8	0	2.9	0	0	0.8	2.7	3.3	0	0.1	2.1	11	1.5	2.9	0.6	6.25	3.3	1.0	2.7	0
URB597	G. Metabolic modifier	Other metabolic modifier	0.1	0.1	0	0		0	0	0.3	0.1	0	0.8	0.7	2.5	0		0	0	0	0	0	0	0.1	0	0	0	0	0	0	0	0	0	0	0	0	0	0	0	0	0	0	0	0.8	0	0	0	0	0	1.2		0	1.6	0	0	0.0	0	0
Valproic acid	C. Differentiating/epigenetic modifier	HDAC inhibitor	0	0	0	0	0	0	0	0	0	0	0	0	0	0		0	0	0	0	0	1.6	0	0	0	0	0	0	0	0	0	0	0	0	0	0	0	0	0	0	0	0	0.8	0	0	0	0	0	0	0	0	0	0	0	0.0	0	0
Valrubicin	A. Conventional chemotherapy	Topoisomerase inhibitor	11.6	13.4	15.4	2.4	0.1	2.4	7.3	5.4	6	4.6	22.1	0.5	5.3	6		7	6.6	4.9	8.1	7.7	7.1	4.1	5.5	3.3	4.8	0.1	9.9	6.6	9.7	7.4	7.5	8.7	6.2	4.1	3.4	11.2	3.6	9.6	8.1	5.1	6.6	0.8	3.9	8.7	3.8	8.8	2.4	20.1	9.1	12.2	11.85	10.15	8.65	7.7	7.3	8.7
Vandetanib	B. Kinase inhibitor	Multi kinase inhibitor	6.5	0	0	0.1	0.4	0.1	1.4	3.4	4.3	0	0.2	0	3.6	5.7		2.2	1.1	0.2	0.9	0	2.3	0	1.3	1.7	1.4	0.6	0	4.1	3.8	5.5	0.1	0.2	3.4	0	0	0	1.6	0.2	0	2.8	0.1	0.8	0	2.3	0	0	0.8	0	3.3	0	2.95	1.65	0.6	1.0	0.3	0
Varespladib	X. Other	Other	0.8	0	0	0		0	0	0.3	0	1	2.1	0	0	0	0	0	0	0	0	0	0	0	0	0	0	0	0	0	0.4	0	0.7	0	0	0	0	0	0	0	0	0	0	0.8	0	0.7	0	0	4.1	2.9		0	0	0	0	0.0	0	0
Varlitinib	B. Kinase inhibitor	EGFR or ERBB2 inhibitor	10.6	0	0.9	1.4		0.1	0.6	1.1	3.6	0	3.8	0.7	2.8	9.5		5.7	4.7	3.5	2.3	0.1	0	1.3	3.6	3.7	0.5	2.6	0	3.2	1.3	1.7	2.5	0	4.1	0	0	2.9	2.5	0	0	2.5	0	0.8	0	4.1	0.2	0.2	1	1.5	0	0.3	2.5	2.35	0.9	0.2	2.8	0
Vatalanib	B. Kinase inhibitor	VEGFR inhibitor	0	0	0	0.5	0.1	0	0	0	0	0	0.3	0	0	0.2		0	0	0	0	0.1	0	0	0	0	0	0	0	0	0	0	0	0	0	0	0	0	0	0	0	0.5	0	0.8	0	0	0	0	0	0	0	0	0	0	0	0.0	0.1	0.1
VE-821	B. Kinase inhibitor	Other kinase inhibitor	0	0	0	0		0.9	0	0.3	0	0	5.2	0	0	0.8		1.9	2.3	1	0	0.1	0	0	3.2	1.3	0.3	0.8	0	0	0	0	0	0	0	0	0	0	0.8	0	0	0	0	0.8	0	0	1.1	0	0.2	4.4	0.6	2.6	0.7	0.8	0.05	0.0	0	1.6
Veliparib	C. Differentiating/epigenetic modifier	PARP inhibitor	0	0.3	2.4	0.1		0	0.2	0	0.5	0.6	4.3	0	2.8	0.5		0	0	9.2	0	1.3	0	3.7	0.8	2.8	0	0	0	0	3.9	0	1	0.1	0	0	0	0	6.1	0	0	0.4	0	0.8	0	0	0	1.4	0.4	5.1	0.1	2.6	1.05	1.5	1.25	0.0	1.8	0
Vemurafenib	B. Kinase inhibitor	RAF inhibitor	0	0	0.1	0	0.4	1	0	0.1	1.3	0	1	0.4	0.2	1.1	3.8	2.7	1.3	3	0	0.4	0.1	4.3	1.1	2.9	3.5	2.9	3	1.6	2.4	4	1.7	0.2	3.3	0	0	2.5	3.5	0.8	2.9	1.8	0	0.8	0	0.2	0	0.7	0	0	0	1.7	0.85	2.9	1.2	0.6	1	0.2
Venetoclax	D. Apoptotic modulator	BH3 mimetic	0	0.5	6.3	0	0.5	0	0	0	0	0	0	0	0	0	0	0	0	0	0.1	0.3	0	1.2	0	0	0	0	0	0	0	0	0	0	0	0	0	0	0	0	0	0	0	0.8	0	0	0	0	0	0.4	0	0	0	0	0	0.0	0	0
VER 155008	X. Other	HSP inhibitor	9.6	1	0.2	6.4		1.4	5.2	3.2	5.1	5.8	11.1	0.9	2.3	6.2		3.4	4	2.8	0	3.3	1.1	5.1	2.2	3.1	3.2	1.2	0	3.8	0	4.7	2	0.7	3.8	1.4	2.2	0	5.1	0	0.6	4.2	1	0.8	0	4.1	1	7.7	2.7	8.6		5.5	4.65	4.35	2.55	1.0	2.4	0.3
Verdinexor	D. Apoptotic modulator	Other apoptotic modulator	14.8	7.8	10.5	2.4		5.1	8.1	7.7	8.5	6.7	13.9	11	9	11.7		7.9	7.6	8.6	0	17.2	5.6	11.2	4.4	8.1	7.2	5.8	3.2	0	5.1	4.6	6.5	6.8	6.5	4.8	6	1.7	8.8	2.6	3.6	5.9	5.1	0.8	0.6	8.7	6.7	10.9	0.1	13.9		14	8.9	11	4.75	1.9	7.3	4
Vesatolimod	E. Immunomodulatory	TLRs ligand	0	0	0	0		0	0	0	0	0	0	0.5	0	1		0	0	0	0	0	0	0	0	0	0	0	0	0	0	0	0	0	0	0	0	0	0	0	0	0	0	0.8	0	0	0	0	4.1	1	0	0	0	0	0	0.0	0	0
VGX-1027	E. Immunomodulatory	Other immunomodulatory	0	0	0	0		0	0	0	0	0	0	0	0	0		0.2	0	0	0	0	0	0	0	0	0	0.5	0	0	0	0	0	0	0	0	0	0	0	0	0.2	0	0	0.8	0	0	0.3	0	0	0	0	0	0	0	0	0.0	0	0
Vidofludimus	C. Differentiating/epigenetic modifier	Other differentiating/epigenetic modifier	1.5	0	0	0		0	0	0	0.1	1.3	1.6	1.6	0	0		0.2	0.3	0	0	0.6	0	3.4	0	0.1	0.1	0	0	0	0.1	0	0	0	0	0	0.3	0	0	0	0	0	0	0.8	0	0.1	0	1.1	0	1.8	0	0	0	0	0	0.1	0	0
Vinblastine	A. Conventional chemotherapy	Mitotic inhibitor	21.6	10.7	7.2	5.3	0	6.8	5.3	6.6	7.2	8.7	26.5	1.7	10.5	18.2	9.9	10.4	4.7	1.5	7.4	13.2	4.8	6.8	0	0.6	0.4	0.6	18.9	5.1	22.2	5.6	8	3.5	4.8	0	0	0	9.2	13.5	15.7	4.4	20.2	0.8	10.9	15.7	6.7	12.7	4.8	25.7	2	10.8	12.05	5.95	7.55	2.2	1.3	13.6
Vincristine	A. Conventional chemotherapy	Mitotic inhibitor	19.7	14.7	10	1.8	0.5	0	1.7	4.5	1.6	6.1	27.8	0	12.6	17.9	10.9	11.4	5.1	1.9	9.5	15.7	5.5	2.2	3.3	1.1	0	3	14.6	3.9	19.3	4.5	9.5	3.4	4.2	0	0.6	3	6.7	6.7	12	3.8	17.8	0.8	11.9	27.7	7.2	8.9	3.3	26	1.6	11.4	9.65	5.65	9.3	6.1	1	12.6
Vinflunine	A. Conventional chemotherapy	Mitotic inhibitor	4.3	1	0.2	0	0	0	0	0.7	0.2	0	4	0	3.7	2.9	2	2.6	2.3	1.7	4.2	4	0	0.8	1.1	0	0	1.7	2.5	1.4	4.9	1.1	4.3	3.4	0.6	0	0.6	0	0.5	1	4.1	0	2.9	0.8	3.3	16.6	2.4	1.9	0.1	3.8	0	3.5	5.25	4.9	3.4	4.6	0.1	2.7
Vinorelbine	A. Conventional chemotherapy	Mitotic inhibitor	28.6	18.2	12	6.2	7.8	5.7	7.9	6.6	5.4	13.9	38	10.2	26.4	29.9	15.1	20.9	12.3	8.9	31.4	26.9	21.1	19.4	9.8	2.6	8.5	7.8	21.3	13.4	26.2	14.6	28.4	21.9	16.7	1.2	0.4	4.1	11.5	26.9	31	14.7	25.2	0.8	25.8	41.9	21.4	26.7	7.8	38	4.8	30.3	24.15	24	32.55	32.9	7	18.3
Vismodegib	X. Other	Hedgehog inhibitor	0	0	0	0	0	0	0	0	0	0	0.4	0	0	0		1.2	0	0	0	0	0	0.5	0.2	2.4	2	0	0	0	0	0	0	0	0	0	0	0	2.6	0	0	0	0	0.8	0	0	0	0	0	0.3	0.9	0	0	0	0.35	0.0	0.5	0.6
Vistusertib	B. Kinase inhibitor	PI3K or MTOR inhibitor	21.1	7.3	5	16.2		11.5	13.5	15.7	17.6	12.5	24.1	9.8	12.7	23.4		12.8	9.7	12.8	14.6	9.1	8.1	16.7	13.9	13.3	16.2	10.7	16.1	9.1	17.2	14.3	15.8	13.8	11.5	7.6	10	16.9	16.7	12.7	14	10	12.3	0.8	13.9	17.3	13.4	12.5	10.5	26.1	7.1	17	11.8	13.3	14.35	14.8	14.7	11.5
VLX1570	X. Other	Protease/proteasome inhibitor	22.9	26.4	27	11.4		16.7	10.6	20.3	10.1	22.1	28.7	25.3	23.3	24.1		19.3	17.9	24.1	17.5	20.9	0.6	19.2	22.6	21.2	21.9	21.5	17.6	26.1	17.3	22.7	8.8	4.1	24.1	6.9	5.2	11.4	12.7	5.3	5.9	19.9	10.9	0.8	9.2	29	17.1	24.2	20.1	24.7		23.1	21.65	23	12.9	4.5	14.4	5.5
Volasertib	B. Kinase inhibitor	Other kinase inhibitor	15.5	7.2	2.2	1	0.6	2	1.3	1.9	2.9	2.7	18	5.9	18.2	11.9	0	5.9	1.3	3.5	1	9.3	0	0.1	2.9	0	0	2.7	4.8	2.3	2.8	1.6	3	0.1	1.1	0.2	0	0	1.9	0	1.2	2.1	2.7	0.8	0.3	16.7	5.1	13.1	4	17.9	0	12.8	11.85	7.75	2.4	0.8	1.9	7.5
Vorinostat	C. Differentiating/epigenetic modifier	HDAC inhibitor	8.3	5.9	7.8	1.1	5.7	2.6	4.2	3.9	9	7	11.3	16.5	8.5	10.9	9.1	7.1	7.2	3.6	0	6.4	4.4	2.1	14.2	11.5	12.4	10.3	6.4	4.4	4.3	3.6	3.4	5.8	5.8	2.9	3.3	2.9	7.2	2.1	2.8	4.2	4.5	0.8	4.4	3.8	4.8	11.9	5.8	12.6	1.7	8.3	8.6	6.45	4.4	2.7	8.3	3.3
VS-4718	B. Kinase inhibitor	Other kinase inhibitor	12.9	2.3	3.2	6.8		0.1	3.5	7.2	3.7	5.6	14.4	6.5	10.1	7		5.6	5.6	4.6	10.6	7.7	8.6	8.6	4.4	0	2.3	1	12	8.8	9.7	10.4	8.9	9	10.6	6.9	7	9.3	13.1	9.9	9.6	8.6	9.3	0.8	9.6	7.4	7	4.4	13.2	9.9	7.1	7.6	5.55	6.55	12.3	7.7	9.4	5.4
WEHI-539	D. Apoptotic modulator	BH3 mimetic	0	0	0.1	0		0	0	0	0	0	0	0.1	0.2	0.5		3.6	0.8	0.5	0.9	1.4	0	4	1	0	0.3	0.3	0	0	0.1	0.1	0	0.1	0	0	0	0	0	0	2.2	0	0	0.8	0.2	1.1	0	0	0	0		1	0.6	4.75	0.45	0.0	5.2	0
XAV-939	C. Differentiating/epigenetic modifier	Other differentiating/epigenetic modifier	0	0	0	2.1		0.8	0.5	0.4	0	0.1	0	1.5	2.3	9.1		3.7	0.7	0.4	2.3	0	0.2	6.3	1	2	0	0	2.3	0	3.3	0.6	0.2	1.5	0	0	0	1.1	3.3	2.5	5.4	0	0.2	0.8	2.3	7.1	0	0	2.4	0	0	0.5	0.7	0	0.15	1.7	1.5	0
ZSTK474	B. Kinase inhibitor	PI3K or MTOR inhibitor	19	6.4	6.9	12.6		5.7	7.1	10.3	9	8.2	15.3	10.8	14.3	22.6		14	11.9	10.2	15.6	3.2	8.6	11	9.5	9.7	8.5	11.1	14.1	9.4	16.7	12.4	14.6	12.4	10.8	5.1	5.6	15.6	10.5	9.4	11.9	8.5	9.3	0.8	11.5	15	7.8	8.1	9.5	19.4	6.4	7.7	10.5	8.8	14.95	14.0	12.2	4.4

Supplemental Table 4

DSS comparison between all PDCs derived from ascites or tissue, based on individual drugs

DRUG_NAME	p.value	mean_ascites	mean_tissue	logFC	p.adj
1-methyl-D-tryptophan	0.39	0.27	0.00	0.26	0.7563527
4-hydroxytamoxifen	0.25	4.09	3.04	1.05	0.6384922
8-amino-adenosine	0.12	21.07	19.43	1.64	0.5429712
8-chloro-adenosine	0.03	12.60	7.96	4.64	0.4960204
A-1155463	0.26	9.52	13.53	-4.01	0.6502002
A-1210477	0.63	6.57	6.33	0.24	0.8454114
A-1331852	0.46	4.88	6.26	-1.38	0.7629368
A-366	0.01	1.59	0.20	1.38	0.4828655
A-419259	0.97	10.83	11.09	-0.26	0.9905677
ABC294640	0.10	4.74	3.48	1.26	0.5429712
ABT-751	0.10	8.46	5.56	2.90	0.5429712
AMG-232	0.16	2.10	0.37	1.73	0.5663914
AMG-337	NaN	0.00	0.00	0.00	NaN
AMG-925	0.29	0.87	0.16	0.71	0.6739784
AMG319	0.13	0.68	0.09	0.59	0.5429712
APR-246	1.00	0.18	0.13	0.05	1.0000000
AR-42	0.05	10.21	8.07	2.14	0.4989832
ARV-825	0.94	10.05	9.01	1.04	0.9778587
ASP3026	0.78	5.21	5.48	-0.27	0.9096508
AT 101	0.48	14.98	14.20	0.78	0.7629368
AT-406	0.35	2.09	4.24	-2.15	0.7421939
AT13148	0.62	1.32	1.90	-0.57	0.8454114
AT7519	0.16	12.54	11.60	0.93	0.5663914
AT9283	0.89	3.00	2.92	0.08	0.9595549
AVN944	0.05	12.75	8.49	4.26	0.4989832
AZ 3146	0.65	3.14	2.53	0.61	0.8455843
AZ191	0.32	2.07	1.37	0.70	0.6961959
AZD-1080	0.39	0.03	0.10	-0.07	0.7563527
AZD-5363	0.87	4.56	4.01	0.54	0.9595549
AZD-5438	0.95	6.81	6.74	0.07	0.9865162
AZD-6482	0.48	1.76	1.79	-0.03	0.7629368
AZD-8186	0.84	2.05	1.46	0.59	0.9402133
AZD0156	0.69	1.63	1.00	0.63	0.8665021
AZD1152-HQPA	0.09	3.59	1.80	1.79	0.5244741
AZD1208	0.46	1.16	0.80	0.36	0.7629368
AZD1480	0.40	0.72	0.13	0.60	0.7563527
AZD1775	0.09	14.33	10.92	3.41	0.5244741
AZD3759	0.07	1.08	2.17	-1.09	0.5181296
AZD3965	0.92	0.66	0.03	0.63	0.9774233
AZD4547	0.14	1.15	0.00	1.15	0.5429712
AZD6738	0.12	9.04	6.91	2.13	0.5429712
AZD7545	0.11	0.33	0.06	0.27	0.5429712
AZD7762	0.01	6.12	3.54	2.58	0.4713480
AZD8055	0.08	20.49	18.12	2.37	0.5182156
Abemaciclib	0.82	4.21	2.21	2.00	0.9314794
Abexinostat	0.03	12.65	9.16	3.49	0.4960204
Abiraterone	0.46	0.28	0.50	-0.22	0.7629368
Acalabrutinib	0.14	0.13	0.00	0.13	0.5429712
Acitretin	0.52	1.39	0.66	0.73	0.7905405

Afatinib	0.07	3.98	6.63	-2.65	0.5181296
Afuresertib	0.98	3.75	3.33	0.41	0.9944730
Aldoxorubicin	0.25	7.24	5.53	1.71	0.6417486
Alectinib	0.14	0.79	0.29	0.51	0.5429712
Alisertib	0.18	6.53	3.93	2.60	0.5990458
Allopurinol	0.77	2.19	0.08	2.11	0.9096508
Alpelisib	0.06	2.56	3.64	-1.08	0.5109537
Altiratinib	0.94	4.64	4.37	0.26	0.9778587
Alvocidib	0.41	15.59	15.33	0.25	0.7563527
Amcasertib	0.12	3.78	1.67	2.11	0.5429712
Aminoglutethimide	0.74	0.04	0.15	-0.11	0.8973379
Amsacrine	0.66	6.65	4.19	2.47	0.8455843
Amuvatinib	0.06	2.67	0.91	1.76	0.5027989
Anagrelide	0.93	1.25	1.62	-0.37	0.9778587
Anastrozole	0.30	0.00	0.02	-0.02	0.6869234
Apalutamide	0.73	0.06	0.01	0.05	0.8829666
Apatinib	0.71	0.56	0.16	0.40	0.8696719
Arsenic(III) oxide	0.41	0.01	0.00	0.01	0.7563527
Asciminib	0.80	0.63	0.65	-0.02	0.9264843
Atorvastatin	0.19	1.85	2.06	-0.21	0.6012754
Auranofin	0.12	7.68	4.98	2.70	0.5429712
Axitinib	0.12	2.07	0.67	1.39	0.5429712
Azacitidine	0.01	4.93	2.64	2.29	0.4828655
BAY 87-2243	0.09	8.38	2.79	5.58	0.5244741
BAY-1436032	0.22	0.34	0.05	0.29	0.6384922
BCI	0.13	16.37	14.28	2.09	0.5429712
BGB-283	0.07	3.85	2.63	1.22	0.5181296
BGB324	0.25	4.05	2.88	1.16	0.6384922
BI 2536	0.29	10.30	5.53	4.76	0.6739784
BIIB021	0.25	12.38	10.17	2.22	0.6400935
BMS-754807	0.07	9.50	11.83	-2.33	0.5181296
BMS-777607	0.43	0.34	0.02	0.32	0.7563527
BMS-911543	0.58	0.26	0.37	-0.11	0.8347758
BMS863233	0.46	1.23	0.66	0.57	0.7629368
BRD7116	0.02	1.99	0.37	1.62	0.4828655
BX-912	0.45	3.71	3.04	0.67	0.7629368
Bafetinib	0.14	0.09	0.00	0.09	0.5429712
Baricitinib	0.27	0.00	0.05	-0.05	0.6641408
Belinostat	0.01	13.21	9.36	3.84	0.4713480
Bentamapimod	0.66	1.86	1.42	0.44	0.8455843
Bexarotene	0.59	1.03	1.23	-0.20	0.8347758
Bicalutamide	0.25	0.85	0.38	0.47	0.6405503
Bimatoprost	0.97	0.17	0.08	0.09	0.9905677
Binimetinib	0.63	3.99	3.12	0.88	0.8454114
Birabresib	0.67	9.13	7.14	1.99	0.8589130
Birinapant	0.70	3.24	3.77	-0.52	0.8686009
Bleomycin	0.70	3.69	1.72	1.97	0.8686009
Bortezomib	0.65	23.83	24.60	-0.77	0.8455843
Bosutinib	0.33	7.86	6.93	0.92	0.7064197
Brigatinib	0.09	3.18	4.37	-1.19	0.5244741
Brivanib	0.30	0.00	0.03	-0.03	0.6869234
Bryostatin 1	0.76	1.39	1.69	-0.30	0.9039728
Buparlisib	0.79	7.66	7.51	0.14	0.9162302
C646	0.18	1.17	1.56	-0.39	0.5990458

CC-115	0.97	10.64	10.25	0.39	0.9905677
CC-223	0.14	5.31	4.09	1.23	0.5429712
CC122	0.05	0.41	0.00	0.41	0.4989832
CCT196969	0.86	4.58	3.91	0.67	0.9530552
CEP-32496	0.92	1.74	1.23	0.51	0.9774233
CEP-37440	0.90	7.94	7.86	0.08	0.9700926
CPI-0610	0.17	6.20	3.50	2.70	0.5752278
CPI-360	0.24	0.04	0.00	0.04	0.6384922
CPI-613	0.95	0.09	0.08	0.00	0.9873546
CUDC-305	1.00	8.83	9.02	-0.18	1.0000000
CUDC-907	0.02	30.51	26.02	4.50	0.4828655
Cabazitaxel	0.23	15.71	10.34	5.37	0.6384922
Cabozantinib	0.60	1.49	1.41	0.08	0.8378599
Canertinib	0.96	10.55	11.18	-0.63	0.9873546
Capecitabine	0.43	0.03	0.07	-0.04	0.7563527
Capmatinib	0.21	0.07	0.20	-0.13	0.6384922
Carboplatin	0.10	4.78	3.20	1.58	0.5429712
Carfilzomib	0.46	22.43	20.49	1.94	0.7629368
Cediranib	0.06	0.52	0.05	0.47	0.5027989
Celecoxib	0.46	0.06	0.08	-0.02	0.7629368
Cerdulatinib	0.03	1.96	0.64	1.32	0.4960204
Ceritinib	0.31	2.11	2.61	-0.51	0.6961959
Chloroquine	0.53	6.06	6.36	-0.30	0.7980697
Cilengitide	0.60	1.74	1.03	0.71	0.8378599
Cisplatin	0.13	11.68	8.50	3.18	0.5429712
Cladribine	0.25	4.00	1.19	2.81	0.6384922
Clofarabine	0.12	11.82	5.38	6.43	0.5429712
Clomifene	1.00	3.94	4.07	-0.13	1.0000000
Cobimetinib	0.29	5.81	4.74	1.07	0.6739784
Copanlisib	0.73	8.99	9.93	-0.94	0.8855636
Crenolanib	0.83	4.02	4.09	-0.06	0.9358950
Crizotinib	0.82	0.08	0.10	-0.01	0.9314794
Cytarabine	0.34	5.72	2.78	2.94	0.7208606
Cytarabine/Idarubicin	0.14	9.51	4.90	4.61	0.5429712
DEL-22379	0.95	7.59	7.58	0.02	0.9865162
Dabrafenib	0.62	0.59	0.33	0.27	0.8454114
Dacomitinib	0.48	5.18	6.53	-1.35	0.7629368
Dactinomycin	0.38	24.31	22.76	1.54	0.7563527
Dactolisib	0.15	14.78	12.79	1.99	0.5607478
Danusertib	0.75	8.15	6.60	1.55	0.9003286
Daporinad	0.11	21.35	14.07	7.28	0.5429712
Darapladib	0.13	0.89	0.14	0.75	0.5429712
Dasatinib	0.25	6.01	7.68	-1.67	0.6384922
Daunorubicin	0.23	7.71	5.17	2.54	0.6384922
Decernotinib	0.36	0.23	0.07	0.16	0.7482851
Decitabine	0.27	2.07	1.26	0.81	0.6646899
Deferoxamine	0.13	2.73	1.46	1.27	0.5429712
Dexamethasone	0.28	0.37	0.74	-0.37	0.6727284
Digoxin	0.11	13.92	12.00	1.92	0.5429712
Dinaciclib	0.65	17.37	18.54	-1.17	0.8455843
Disulfiram(+CuCl2)	0.88	4.26	4.18	0.08	0.9595549
Docetaxel	0.30	15.43	11.11	4.32	0.6878734
Doramapimod	0.04	1.81	0.64	1.17	0.4960204
Dovitinib	0.14	0.30	0.04	0.26	0.5525617

Doxorubicin	0.44	6.32	4.74	1.58	0.7563527
Duvelisib	0.60	0.59	0.37	0.22	0.8378599
E7820	0.57	5.69	4.06	1.63	0.8333432
ENMD-2076	0.38	8.64	7.37	1.27	0.7563527
EPZ-5687	0.03	0.43	0.09	0.34	0.4960204
EPZ015666	0.52	1.18	0.45	0.73	0.7905405
EPZ031686	0.14	0.52	0.00	0.52	0.5429712
Eltanexor	0.69	18.60	17.39	1.21	0.8665021
Enasidenib	0.42	0.19	0.23	-0.04	0.7563527
Encorafenib	0.79	0.54	0.40	0.14	0.9170828
Ensartinib	0.66	0.98	0.87	0.11	0.8455843
Entinostat	0.11	5.82	4.38	1.44	0.5429712
Entospletinib	0.66	0.03	0.32	-0.29	0.8455843
Entrectinib	0.21	0.96	0.43	0.54	0.6384922
Enzalutamide	0.45	0.64	0.41	0.23	0.7629368
Enzastaurin	0.82	0.78	0.43	0.35	0.9314794
Epacadostat	0.40	0.29	0.08	0.22	0.7563527
Epirubicin	0.18	7.98	4.42	3.56	0.5990458
Erastin	0.00	15.21	4.88	10.33	0.4713480
Erdafitinib	0.52	1.83	0.07	1.76	0.7905405
Eribulin	0.05	17.07	8.72	8.35	0.4989832
Erlotinib	0.22	6.00	7.61	-1.61	0.6384922
Etoposide	0.22	5.23	2.86	2.38	0.6384922
Everolimus	0.04	6.75	4.00	2.75	0.4960204
Exemestane	0.34	0.38	0.11	0.28	0.7292744
FRAX486	0.33	4.27	3.38	0.89	0.7046038
Fedratinib	0.45	5.96	4.63	1.33	0.7629368
Filanesib	0.07	13.10	7.10	6.00	0.5181296
Filgotinib	0.11	1.88	0.88	1.00	0.5429712
Finasteride	0.47	0.01	0.01	-0.01	0.7629368
Fingolimod	0.02	7.38	5.95	1.43	0.4828655
Floxuridine	0.01	7.82	2.53	5.29	0.4713480
Fludarabine	0.31	1.09	0.36	0.73	0.6961959
Fluorouracil	0.01	4.06	0.78	3.27	0.4713480
Flutamide	0.08	0.33	0.04	0.29	0.5181296
Foretinib	0.61	3.25	2.31	0.94	0.8389581
Fostamatinib	0.17	2.56	2.86	-0.30	0.5829313
Fulvestrant	0.93	0.01	0.01	0.00	0.9776876
GDC-0084	0.78	10.47	10.04	0.43	0.9096508
GDC-0623	0.95	3.61	4.07	-0.46	0.9865162
GDC-0853	0.43	0.02	0.00	0.02	0.7563527
GDC-0919	0.56	0.42	0.37	0.05	0.8236341
GNE-0877	NaN	0.00	0.00	0.00	NaN
GNE-7915	0.24	0.19	0.00	0.19	0.6384922
GSK-1070916	0.19	2.55	1.58	0.96	0.6052212
GSK-2334470	0.57	1.50	1.10	0.40	0.8333432
GSK-461364	0.16	13.46	8.30	5.16	0.5694928
GSK-690693	0.59	5.19	3.60	1.58	0.8347758
GSK-J4	0.70	4.17	2.35	1.82	0.8686009
GSK2256098	0.02	0.60	1.08	-0.48	0.4828655
GSK2636771	0.75	0.77	0.43	0.34	0.9039728
GSK2656157	0.15	1.06	0.42	0.64	0.5663914
GSK269962	0.63	3.23	2.43	0.80	0.8454114
GSK2801	0.62	0.15	0.06	0.09	0.8454114

GSK2830371	0.02	1.27	0.17	1.10	0.4828655
GSK2879552	0.43	0.10	0.01	0.10	0.7563527
GSK343	NaN	0.00	0.00	0.00	NaN
GSK650394	0.88	0.28	0.43	-0.15	0.9595549
GSK923295	0.09	10.66	5.88	4.78	0.5220806
Galiellalactone	0.05	4.53	2.08	2.45	0.4989832
Galunisertib	NaN	0.00	0.00	0.00	NaN
Gandotinib	0.57	5.23	4.83	0.41	0.8333432
Ganetespib	0.60	13.11	11.84	1.27	0.8378599
Gedatolisib	0.08	12.48	10.10	2.37	0.5220806
Gefitinib	0.34	5.95	7.56	-1.62	0.7261571
Gemcitabine	0.05	10.29	4.83	5.46	0.4989832
Gilteritinib	0.65	2.11	1.68	0.43	0.8455843
Givinostat	0.08	5.72	4.01	1.71	0.5220806
Glasdegib	0.27	0.15	0.03	0.12	0.6671774
Glesatinib	0.62	0.94	0.47	0.47	0.8454114
Golvatinib	0.42	0.48	0.95	-0.47	0.7563527
Goserelin	0.77	2.66	0.02	2.64	0.9096508
Hydroxyfasudil	0.47	0.19	0.07	0.12	0.7629368
Hydroxyurea	0.08	5.08	1.61	3.48	0.5181296
I-BET151	0.70	7.04	6.18	0.86	0.8686009
IOX-1	0.32	1.85	0.85	1.00	0.7046038
IOX-2	0.17	0.27	0.35	-0.09	0.5752278
Ibrutinib	0.58	2.39	3.02	-0.63	0.8347758
Icotinib	0.12	2.91	4.24	-1.33	0.5429712
Idarubicin	0.37	9.31	6.55	2.76	0.7497097
Idasanutlin	0.44	3.68	1.19	2.49	0.7563527
Idelalisib	0.06	2.00	0.88	1.12	0.5027989
Imatinib	0.43	0.18	0.43	-0.24	0.7563527
Imiquimod	0.97	2.54	0.01	2.54	0.9905677
Indibulin	0.39	7.86	5.90	1.96	0.7563527
Infigratinib	NaN	0.00	0.00	0.00	NaN
Ipatasertib	0.70	4.58	3.69	0.89	0.8686009
Itraconazole	0.66	1.82	1.39	0.43	0.8455843
Ivosidenib	0.25	0.13	0.02	0.11	0.6384922
Ixabepilone	0.31	3.49	2.48	1.02	0.6961959
Ixazomib	0.85	11.58	10.95	0.62	0.9486213
JPH203	0.42	0.02	0.00	0.02	0.7563527
JQ1	0.22	11.61	8.64	2.97	0.6384922
KD025	0.28	3.55	2.24	1.31	0.6727284
KU-60019	0.76	1.76	1.45	0.31	0.9093617
LY-2584702	0.05	1.80	0.25	1.55	0.4989832
LY-2874455	0.12	5.33	4.85	0.48	0.5429712
LY3009120	0.30	10.11	8.68	1.43	0.6861317
LY3023414	0.25	11.00	9.65	1.35	0.6384922
Lapatinib	0.61	0.83	1.42	-0.59	0.8389581
Larotrectinib	0.14	0.18	0.00	0.18	0.5429712
Lasofoxifene	0.28	0.36	0.17	0.19	0.6727284
Lenalidomide	0.73	0.28	0.10	0.18	0.8840056
Lenvatinib	0.37	0.82	0.20	0.62	0.7497097
Letrozole	0.39	0.21	0.00	0.21	0.7563527
Linifanib	0.49	0.12	0.16	-0.04	0.7675784
Linsitinib	0.42	2.14	2.59	-0.45	0.7563527
Litronesib	0.04	13.46	6.88	6.58	0.4960204

Lomeguatrib	0.19	0.02	0.05	-0.03	0.6052212
Lonafarnib	0.12	12.36	9.53	2.84	0.5429712
Losmapimod	0.04	1.58	0.63	0.96	0.4960204
Lovastatin	0.79	0.29	0.40	-0.11	0.9156139
Lucitanib	0.09	2.19	0.72	1.47	0.5244741
Luminespib	0.63	15.31	13.88	1.43	0.8454114
MK-0752	0.28	0.00	0.02	-0.02	0.6727284
MK-2206	0.64	2.03	1.94	0.09	0.8455843
MK-8745	0.18	4.42	2.03	2.39	0.5942115
MK-8776	0.15	1.26	0.64	0.62	0.5663914
ML323	0.16	1.31	0.25	1.07	0.5663914
ML390	0.23	4.11	2.18	1.93	0.6384922
MST-312	0.05	9.79	6.55	3.25	0.4989832
Marimastat	0.70	0.36	0.34	0.02	0.8686009
Masitinib	0.48	2.03	1.64	0.39	0.7629368
Megestrol acetate	0.32	0.27	0.53	-0.27	0.6961959
Mepacrine	0.05	13.07	10.54	2.53	0.4989832
Mercaptopurine	0.22	4.25	1.42	2.83	0.6384922
Merestinib	0.66	0.48	0.68	-0.21	0.8455843
Metformin	NaN	0.00	0.00	0.00	NaN
Methotrexate	0.55	4.88	3.45	1.42	0.8211493
Methylprednisolone	0.42	0.19	0.78	-0.59	0.7563527
Midostaurin	0.30	6.48	7.98	-1.50	0.6852521
Milciclib	0.98	10.39	9.06	1.33	0.9944730
Miltefosine	0.30	6.83	8.05	-1.22	0.6890909
Mitomycin C	0.66	1.39	0.81	0.58	0.8455843
Mitotane	0.39	1.67	0.17	1.50	0.7563527
Mitoxantrone	0.07	6.50	3.69	2.82	0.5181296
Mivebresib	0.48	19.12	16.37	2.75	0.7629368
Mocetinostat	0.81	9.69	9.31	0.38	0.9289648
Molibresib	0.13	5.91	3.62	2.29	0.5429712
Momelotinib	0.61	3.49	3.25	0.24	0.8427575
Motesanib	0.83	0.85	1.17	-0.32	0.9358950
Motolimod	0.08	0.64	0.34	0.30	0.5220806
Mubritinib	0.63	3.91	1.86	2.05	0.8454114
NMS-873	0.48	9.82	8.11	1.71	0.7629368
NVP-AEW541	0.43	6.93	7.69	-0.76	0.7563527
NVP-BGT226	0.52	16.57	15.27	1.30	0.7929268
NVP-BHG712	0.30	2.31	1.46	0.85	0.6852521
NVP-CGM097	0.87	8.06	7.14	0.92	0.9595549
NVP-LCL161	0.98	4.96	7.04	-2.08	0.9944730
NVP-LGK974	0.08	0.72	0.05	0.67	0.5220806
NVP-RAF265	0.89	2.36	1.76	0.59	0.9595549
NVP-SHP099	0.59	1.47	1.27	0.19	0.8347758
Napabucasin	0.01	18.98	15.16	3.82	0.4713480
Navitoclax	0.53	2.73	5.43	-2.69	0.7944291
Necrostatin 2	0.80	0.05	0.10	-0.05	0.9264843
Neflamapimod	0.05	0.61	0.12	0.48	0.4989832
Nelarabine	0.90	0.48	0.18	0.30	0.9647279
Neratinib	0.57	4.54	5.10	-0.56	0.8333432
Nilotinib	0.01	1.80	0.21	1.58	0.4713480
Nilutamide	0.41	0.02	0.06	-0.04	0.7563527
Nintedanib	0.07	5.68	3.45	2.23	0.5181296
Niraparib	0.55	2.49	1.71	0.78	0.8172852

ODM-201	0.43	0.95	0.39	0.56	0.7563527
ONX-0914	0.24	15.18	14.27	0.91	0.6384922
OSU-03012	0.66	0.28	0.50	-0.21	0.8455843
OTS-964	0.19	13.35	12.22	1.13	0.6012754
OTS167	0.87	13.80	13.39	0.41	0.9595549
Olaparib	0.97	3.20	2.06	1.14	0.9905677
Olmutinib	0.88	0.19	0.32	-0.12	0.9595549
Omacetaxine	0.16	23.53	19.47	4.06	0.5749379
Omaveloxolone	0.46	15.28	13.98	1.29	0.7629368
Omipalisib	0.39	16.97	18.20	-1.23	0.7563527
Onalespib	0.65	9.64	8.78	0.85	0.8455843
Oprozomib	0.37	15.86	13.47	2.39	0.7497097
Orteronel	NaN	0.00	0.00	0.00	NaN
Osimertinib	0.79	4.97	5.27	-0.30	0.9156139
Oxaliplatin	0.11	10.10	7.22	2.88	0.5429712
PAC-1	0.21	2.99	1.98	1.00	0.6307466
PCI-34051	0.01	1.92	0.39	1.53	0.4713480
PD0325901	0.89	6.96	6.81	0.15	0.9595549
PF-00477736	0.03	11.12	7.55	3.57	0.4960204
PF-00562271	0.60	5.53	6.06	-0.53	0.8382060
PF-03758309	0.42	16.64	13.09	3.55	0.7563527
PF-04708671	0.73	1.46	1.05	0.41	0.8855636
PF-06463922	0.95	1.23	1.29	-0.06	0.9865162
PF-3845	0.23	1.56	0.54	1.03	0.6384922
PF-4800567	1.00	0.66	0.59	0.06	1.0000000
PF-670462	0.64	1.75	1.29	0.46	0.8455843
PF06650833	0.32	1.99	1.05	0.94	0.7046038
PFI-1	0.50	5.47	4.48	0.98	0.7684873
PH-797804	0.08	0.24	0.00	0.24	0.5220806
PHA 408	0.01	4.48	2.31	2.17	0.4828655
PIM-447	0.27	3.09	1.80	1.29	0.6646899
PS-1145	0.88	0.13	0.14	-0.01	0.9595549
PTC-209	0.78	4.89	4.89	0.00	0.9096508
Paclitaxel	0.08	16.26	10.44	5.81	0.5181296
Pacritinib	0.23	8.48	7.25	1.23	0.6384922
Palbociclib	0.10	2.70	1.01	1.69	0.5429712
Palomid-529	0.49	0.95	0.42	0.53	0.7675784
Panobinostat	0.01	13.82	10.07	3.75	0.4713480
Pazopanib	0.69	1.04	0.63	0.42	0.8665021
Peficitinb	0.92	0.14	0.01	0.13	0.9774233
Pemetrexed	0.37	3.34	1.76	1.58	0.7497097
Pentostatin	0.17	0.35	0.08	0.27	0.5829313
Perifosine	0.68	0.20	0.40	-0.20	0.8608913
Pevonedistat	0.59	9.31	8.06	1.25	0.8347758
Pexidartinib	0.18	1.18	0.60	0.58	0.5990458
Pictilisib	0.90	10.54	9.64	0.90	0.9667140
Pilocarpine	0.25	0.42	0.16	0.25	0.6400935
Pinometostat	NaN	0.00	0.00	0.00	NaN
Pirfenidone	0.39	2.39	0.00	2.39	0.7563527
Pixantrone	0.06	0.49	0.09	0.40	0.5109537
Plerixafor	0.51	0.03	0.02	0.01	0.7905405
Plicamycin	0.47	8.91	7.91	1.00	0.7629368
Pomalidomide	0.93	0.13	0.02	0.11	0.9774233
Ponatinib	0.04	4.84	2.83	2.01	0.4960204

Poziotinib	0.24	7.10	10.07	-2.97	0.6384922
Pracinostat	0.04	12.21	9.15	3.06	0.4960204
Pravastatin	0.68	0.20	0.02	0.18	0.8615785
Prednisolone	0.11	0.94	1.00	-0.06	0.5429712
Prexasertib	0.02	22.85	16.67	6.18	0.4828655
Quisinostat	0.02	10.97	7.55	3.42	0.4828655
Quizartinib	0.56	0.92	0.43	0.49	0.8266202
RGFP966	0.69	2.47	1.83	0.64	0.8665021
RO5126766	0.47	1.73	1.95	-0.23	0.7629368
RSL3	0.28	0.00	0.13	-0.13	0.6727284
Rabusertib	0.43	0.98	0.82	0.16	0.7563527
Radotinib	0.87	0.96	1.22	-0.27	0.9595549
Ralimetinib	0.13	3.40	2.64	0.76	0.5429712
Raloxifene	0.14	3.31	1.71	1.60	0.5429712
Raltitrexed	0.37	3.84	2.11	1.73	0.7497097
Ravoxertinib	0.26	5.12	3.60	1.52	0.6502002
Regorafenib	0.03	4.31	2.53	1.78	0.4960204
Resatorvid	0.24	0.12	0.00	0.12	0.6384922
Resiquimod	0.13	0.54	0.18	0.37	0.5429712
Resminostat	0.03	8.24	5.67	2.57	0.4960204
Ribociclib	0.13	1.58	0.09	1.49	0.5429712
Ridaforolimus	0.04	9.00	5.07	3.93	0.4960204
Rigosertib	0.06	18.33	11.84	6.49	0.5027989
Ripasudil	0.93	0.12	0.06	0.05	0.9774233
Rociletinib	0.57	6.13	6.81	-0.68	0.8333432
Rocilinostat	0.07	5.02	3.60	1.42	0.5181296
Romidepsin	0.20	23.83	22.67	1.16	0.6254250
Roxadustat	0.21	0.28	0.12	0.15	0.6384922
Ruboxistaurin	0.04	6.64	4.40	2.24	0.4960204
Rucaparib	0.20	0.88	0.10	0.78	0.6254250
Ruxolitinib	0.34	0.48	1.13	-0.65	0.7173344
S-63845	0.40	1.79	0.90	0.90	0.7563527
SAR405838	0.83	3.42	2.15	1.27	0.9358950
SB 743921	0.02	4.78	2.03	2.74	0.4828655
SCH772984	0.86	9.23	8.92	0.30	0.9530552
SGC-CBP30	0.12	5.05	3.30	1.75	0.5429712
SGC0946	0.54	0.69	0.39	0.30	0.8131087
SGI-1776	0.27	6.71	5.72	0.99	0.6646899
SH-4-54	0.71	7.21	7.34	-0.13	0.8693846
SN-38	0.48	24.07	20.76	3.31	0.7629368
SNS-032	0.92	17.78	17.89	-0.11	0.9774233
Sabutoclax	0.77	10.47	10.40	0.07	0.9096508
Salinomycin	0.38	15.86	13.18	2.68	0.7563527
Sapanisertib	0.35	13.72	12.22	1.50	0.7295473
Sapitinib	0.02	2.47	5.40	-2.94	0.4828655
Saracatinib	0.17	6.01	7.61	-1.60	0.5752278
Saridegib	0.15	0.87	0.28	0.59	0.5607478
Seliciclib	0.57	1.68	1.05	0.62	0.8333432
Selinexor	0.78	16.54	15.99	0.54	0.9096508
Selonsertib	0.93	0.13	0.01	0.12	0.9774233
Selumetinib	0.40	5.77	5.22	0.56	0.7563527
Senexin B	0.04	0.80	1.68	-0.88	0.4960204
Sepantronium bromide	0.13	35.34	33.23	2.11	0.5429712
Serabelisib	0.16	1.88	2.96	-1.08	0.5663914

Silmitasertib	0.49	6.00	6.39	-0.39	0.7684873
Simvastatin	0.61	0.82	0.61	0.21	0.8389581
Sirolimus	0.19	4.59	3.05	1.54	0.6012754
Sitravatinib	0.82	1.70	1.18	0.52	0.9338992
Sonidegib	0.82	1.43	0.85	0.58	0.9338992
Sonolisib	0.31	0.46	0.11	0.35	0.6961959
Sorafenib	0.59	0.06	0.09	-0.03	0.8347758
Sotrastaurin	0.24	0.35	0.20	0.15	0.6384922
Spebrutinib	0.27	1.06	0.48	0.57	0.6671774
StemRegenin 1	0.92	2.47	2.26	0.21	0.9774233
Sunitinib	0.49	0.79	0.27	0.52	0.7675784
TAK-285	0.46	0.81	1.51	-0.70	0.7629368
TAK-530	0.23	0.86	0.63	0.23	0.6384922
TAK-901	0.50	4.54	3.40	1.14	0.7684873
TEW-7197	0.22	0.44	1.00	-0.56	0.6384922
TG100-115	0.83	0.40	0.14	0.26	0.9358950
TGR-1202	0.58	0.19	0.03	0.16	0.8347758
TGX-221	0.64	2.58	1.77	0.81	0.8455843
TH588	0.64	2.96	1.61	1.35	0.8455843
THZ2	0.24	18.86	17.59	1.28	0.6384922
TIC10	0.02	5.68	1.96	3.72	0.4828655
TRAM-34	NaN	0.00	0.00	0.00	NaN
Tacedinaline	0.98	0.46	0.28	0.18	0.9944730
Tacrolimus	0.05	0.94	0.24	0.70	0.4989832
Taladegib	0.15	0.63	0.20	0.43	0.5607478
Talazoparib	0.45	5.12	2.99	2.13	0.7629368
Talmapimod	0.03	0.56	0.00	0.56	0.4960204
Tamatinib	0.06	2.59	3.53	-0.94	0.5027989
Tamoxifen	0.42	2.82	2.47	0.34	0.7563527
Tandutinib	NaN	0.00	0.00	0.00	NaN
Tanzisertib	0.76	0.77	0.65	0.12	0.9093617
Tarenflurbil	0.87	0.54	1.12	-0.58	0.9595549
Taselisib	0.36	5.32	6.55	-1.23	0.7497097
Tasquinimod	0.52	1.09	0.72	0.38	0.7929268
Tazemetostat	0.43	0.04	0.01	0.03	0.7563527
Telatinib	0.43	0.03	0.00	0.03	0.7563527
Temozolomide	0.41	0.29	0.00	0.29	0.7563527
Temsirolimus	0.04	8.22	4.10	4.12	0.4960204
Teniposide	0.28	0.00	0.01	-0.01	0.6727284
Tepotinib	0.48	0.14	0.49	-0.35	0.7629368
Tesevatinib	0.19	4.44	6.70	-2.26	0.6052212
Thalidomide	0.19	1.55	0.09	1.46	0.6052212
Thioguanine	0.04	6.72	2.25	4.47	0.4960204
Tideglusib	0.58	0.13	0.02	0.10	0.8347758
Tipifarnib	0.37	6.95	4.42	2.53	0.7497097
Tirabrutinib	0.19	0.66	0.70	-0.05	0.6052212
Tivantinib	0.15	2.84	1.76	1.08	0.5663914
Tivozanib	0.12	3.47	2.35	1.12	0.5429712
Tofacitinib	0.47	0.26	0.10	0.16	0.7629368
Topotecan	0.23	11.92	9.40	2.53	0.6384922
Toremifene	0.63	4.22	3.93	0.29	0.8454114
Tosedostat	0.64	6.11	7.30	-1.19	0.8455843
Tozasertib	0.33	4.91	3.24	1.68	0.7046038
Trametinib	0.77	8.49	7.85	0.64	0.9096508

Tretinoin	0.93	0.13	0.52	-0.39	0.9774233
Triapine	0.16	2.58	1.31	1.27	0.5663914
Triciribine	0.48	13.56	12.50	1.06	0.7629368
Trifluridine	0.07	4.22	0.50	3.73	0.5181296
Tubacin	0.01	4.32	1.41	2.91	0.4713480
Tubastatin A	0.04	1.51	0.18	1.33	0.4989832
Tucatinib	0.39	0.01	0.03	-0.02	0.7563527
Tucidinostat	0.14	5.23	3.57	1.65	0.5536097
UCN-01	0.70	16.96	17.55	-0.60	0.8686009
UM729	0.09	1.29	0.47	0.82	0.5264730
UNC0638	0.08	3.23	2.05	1.18	0.5220806
UNC0642	0.04	1.87	1.06	0.81	0.4960204
UNC1215	0.43	0.01	0.00	0.01	0.7563527
UNC2881	0.83	0.74	0.43	0.31	0.9358950
URB597	0.59	0.32	0.14	0.19	0.8347758
Ulixertinib	0.31	7.44	6.37	1.07	0.6961959
Upadacitinib	0.07	0.16	0.00	0.16	0.5181296
Uprosertib	0.98	3.92	3.18	0.74	0.9944730
VE-821	0.37	1.26	0.61	0.65	0.7497097
VER 155008	0.77	4.06	3.99	0.07	0.9096508
VGX-1027	0.73	0.04	0.02	0.03	0.8829666
VLX1570	0.01	21.33	16.66	4.67	0.4713480
VS-4718	0.98	6.58	7.07	-0.49	0.9944730
Valproic acid	NaN	0.00	0.00	0.00	NaN
Valrubicin	0.46	7.69	5.87	1.82	0.7629368
Vandetanib	0.44	1.32	1.56	-0.24	0.7599729
Varespladib	0.40	0.41	0.35	0.06	0.7563527
Varlitinib	0.41	2.03	2.90	-0.88	0.7563527
Vatalanib	0.89	0.03	0.02	0.01	0.9595549
Veliparib	0.89	1.25	1.58	-0.33	0.9595549
Vemurafenib	0.37	0.86	1.28	-0.43	0.7497097
Venetoclax	0.47	0.02	0.05	-0.03	0.7629368
Verdinexor	0.72	8.96	8.38	0.58	0.8827228
Vesatolimod	0.56	0.14	0.32	-0.18	0.8266202
Vidofludimus	1.00	0.42	0.23	0.19	1.0000000
Vinblastine	0.53	9.25	7.20	2.06	0.7944291
Vincristine	0.25	9.85	5.98	3.87	0.6384922
Vinflunine	0.51	2.77	1.58	1.20	0.7905405
Vinorelbine	0.17	19.54	12.69	6.85	0.5901206
Vismodegib	0.65	0.27	0.31	-0.04	0.8455843
Vistusertib	0.55	14.55	13.22	1.33	0.8172852
Volasertib	0.09	8.07	4.13	3.94	0.5220806
Vorinostat	0.22	8.33	6.43	1.90	0.6384922
WEHI-539	0.72	0.53	0.93	-0.40	0.8778278
XAV-939	0.41	1.33	1.08	0.26	0.7563527
ZSTK474	0.81	10.81	9.88	0.93	0.9289648

Supplemental Data 4

DSS comparison between all PDCs derived from ascites (A) or tissue (TT), based on drug subclasses

subclass	p.value	mean_ascites	mean_tissue	logFC	p.adj
AKT inhibitor	0.39	4.72	3.79	0.93	0.6872510
ALK inhibitor	0.87	2.69	2.58	0.11	0.8888971
Alkylating agent	0.43	0.84	0.41	0.44	0.7020970
Antiandrogen	0.02	0.41	0.19	0.22	0.2200095
Antiestrogen	0.12	2.68	2.18	0.49	0.3479416
Antimetabolite	0.02	5.92	2.95	2.97	0.2200095
Aromatase inhibitor	0.47	0.16	0.07	0.09	0.7020970
Aurora kinase inhibitors	0.26	5.15	3.45	1.69	0.4946228
BCR-ABL inhibitor	0.59	2.50	2.76	-0.26	0.7020970
BET inhibitor	0.28	9.29	6.87	2.42	0.5062874
BH3 mimetic	0.58	5.68	6.17	-0.50	0.7020970
BTK inhibitor	0.76	0.85	0.89	-0.04	0.8286396
CDK inhibitor	0.54	9.19	8.39	0.80	0.7020970
DNMT inhibitor	0.02	3.50	1.86	1.64	0.2200095
EGFR or ERBB2 inhibitor	0.16	3.85	5.14	-1.29	0.4016318
FGFR inhibitor	0.18	2.08	1.23	0.85	0.4190417
FLT3 inhibitor	0.96	1.76	1.66	0.10	0.9567173
Glucocorticoid	0.06	0.50	0.84	-0.34	0.2438848
HDAC inhibitor	0.04	8.86	7.08	1.78	0.2304531
HMGCR inhibitor	0.26	0.79	0.77	0.02	0.4946228
HMT inhibitor	0.06	0.24	0.10	0.14	0.2438848
HSP inhibitor	0.59	10.55	9.61	0.94	0.7020970
Hedgehog inhibitor	0.51	0.67	0.42	0.24	0.7020970
IDH1/IDH2 inhibitor	0.12	0.22	0.10	0.12	0.3479416
JAK inhibitor	0.48	2.09	1.66	0.43	0.7020970
Kinesin inhibitor	0.03	10.50	5.47	5.03	0.2200095
MEK1/2 inhibitor	0.79	5.77	5.28	0.50	0.8375438
MET inhibitor	0.51	0.65	0.50	0.14	0.7020970
Mitotic inhibitor	0.14	11.43	7.44	3.99	0.3690920
Multi kinase inhibitor	0.13	2.20	1.64	0.56	0.3507668
Other	0.04	5.14	4.11	1.03	0.2438651
Other antineoplastic	0.21	9.17	7.20	1.97	0.4522256
Other apoptotic modulator	0.69	10.43	10.22	0.21	0.7862732
Other differentiating/epigenetic modifier	0.10	2.45	1.97	0.48	0.3143516
Other hormone therapy	0.57	0.80	0.27	0.54	0.7020970
Other immunomodulatory	0.08	1.18	0.66	0.52	0.2673560
Other kinase inhibitor	0.20	4.12	3.28	0.84	0.4439292
Other metabolic modifier	0.03	5.47	3.58	1.89	0.2200095
PARP inhibitor	0.53	2.59	1.59	1.00	0.7020970
PI3K or MTOR inhibitor	0.76	7.14	6.62	0.53	0.8286396
Protease/proteasome inhibitor	0.69	16.47	15.65	0.82	0.7862732
RAF inhibitor	0.49	2.84	2.27	0.57	0.7020970
Rapalog	0.03	7.14	3.97	3.17	0.2200095
Retinoic acid receptor ligand	0.46	0.85	0.80	0.05	0.7020970
TLRs ligand	0.02	0.80	0.22	0.58	0.2200095
Topoisomerase inhibitor	0.24	7.78	5.54	2.24	0.4897961
VEGFR inhibitor	0.07	1.53	0.76	0.78	0.2556658
p38MAPK inhibitor	0.05	1.36	0.71	0.66	0.2438848
p53 pathway modulator	0.81	3.36	2.20	1.16	0.8446375

Supplemental Table 5

Epithelial differentiation genes	Mesenchyal differentiation genes	Cancer stem cells markers	gamma-secretase complex containing proteins
CDH1	ITGA5	SOX2	PSEN1
ST14	SYDE1	CD44	APH1A
GRHL2	S100A14	KLF4	APH1B
GRHL1	VIM	ALDH1A1	NCSTN
CDH3	CD99L2	PROM1	
LLGL2		ALDH1A3	
EPCAM			
DDR1			
PRSS8			
MISP			
MAL2			
AP1M2			
LSR			
PKP2			
MAP7			
SLC44A2			
RAB25			
MYO5B			
ARHGEF5			
JUP			
TC2N			
SPINT1			
HOOK1			

Supplemental Table 6

ZIP scores A133, Carboplatin, GSI

Model	drug1	drug2	ZIP_synergy	A-1331852 response group
OVSAHO	A-1331852	MK-0752	20.246	resistant
OVSAHO	A-1331852	Nirogacestat	21.195	resistant
OVSAHO	Carboplatin	MK-0752	-0.483	resistant
OVSAHO	Carboplatin	Nirogacestat	-3.320	resistant
OVSAHO	Carboplatin	A-1331852	-1.359	resistant
Gem061-A	A-1331852	MK-0752	6.405	resistant
Gem061-A	A-1331852	Nirogacestat	8.377	resistant
Gem061-A	Carboplatin	MK-0752	2.023	resistant
Gem061-A	Carboplatin	Nirogacestat	2.222	resistant
Gem061-A	Carboplatin	A-1331852	3.258	resistant
OV039-PDF	A-1331852	MK-0752	1.011	PDF
OV039-PDF	A-1331852	Nirogacestat	1.785	PDF
OV039-PDF	Carboplatin	MK-0752	1.004	PDF
OV039-PDF	Carboplatin	Nirogacestat	0.860	PDF
OV039-PDF	Carboplatin	A-1331852	1.111	PDF
OV031-PDF	A-1331852	MK-0752	-3.482	PDF
OV031-PDF	A-1331852	Nirogacestat	-5.961	PDF
OV031-PDF	Carboplatin	MK-0752	1.591	PDF
OV031-PDF	Carboplatin	Nirogacestat	1.248	PDF
OV031-PDF	Carboplatin	A-1331852	2.187	PDF
OvcaR4	A-1331852	MK-0752	-3.399	resistant
OvcaR4	A-1331852	Nirogacestat	-2.878	resistant
OvcaR4	Carboplatin	MK-0752	3.578	resistant
OvcaR4	Carboplatin	Nirogacestat	0.637	resistant
OvcaR4	Carboplatin	A-1331852	3.850	resistant
KURAMOCHI	A-1331852	MK-0752	3.700	sensitive
KURAMOCHI	A-1331852	Nirogacestat	10.113	sensitive
KURAMOCHI	Carboplatin	MK-0752	2.041	sensitive
KURAMOCHI	Carboplatin	Nirogacestat	1.418	sensitive
KURAMOCHI	Carboplatin	A-1331852	4.521	sensitive
OV022-T2	A-1331852	MK-0752	7.1614322	sensitive
OV022-T2	A-1331852	Nirogacestat	9.9306245	sensitive
OV022-T2	Carboplatin	MK-0752	0.4290571	sensitive
OV022-T2	Carboplatin	Nirogacestat	0.6221957	sensitive
OV022-T2	Carboplatin	A-1331852	2.9736026	sensitive
OV030-R	A-1331852	MK-0752	5.006	resistat
OV030-R	A-1331852	Nirogacestat	6.763	resistat
OV030-R	Carboplatin	MK0752	2.791	resistat
OV030-R	Carboplatin	Nirogacestat	0.427	resistat
OV030-R	Carboplatin	A-1331852	4.941	resistat
OV030-S	A-1331852	MK-0752	0.236	sensitive
OV030-S	A-1331852	Nirogacestat	-0.076	sensitive
OV030-S	Carboplatin	MK-0752	2.104	sensitive
OV030-S	Carboplatin	Nirogacestat	-2.728	sensitive
OV030-S	Carboplatin	A-1331852	3.541	sensitive

ZIP scores CBK03, Carboplatin, Nirogacestat

Model	drug1	drug2	ZIP_synergy
OV030-S	DT2216	Nirogacestat	2.313527824
OV030-S	DT2216	Carboplatin	10.66365933
OV030-R	DT2216	Nirogacestat	6.411799386
OV030-R	DT2216	Carboplatin	15.86327844
OV039-PDF	DT2216	Nirogacestat	-1.608453191
OV039-PDF	DT2216	Carboplatin	6.900148433

Supplemental Table 7

DRUG_NAME	Gem061-A	KURAMOCHI	OV022-T2	OV024-A	OV0031-PDF	OV039-PDF	OVCAR4	OVSAHO
A-1331852	100.80	86.52	64.88	97.57	95.04	99.53	90.26	90.52
A-1331852+MK-0752 0.1 nM	99.74	85.25	61.13	92.36	87.21	103.59	85.16	97.90
A-1331852+MK-0752 1 nM	99.03	82.17	61.48	93.86	90.00	102.44	83.62	95.72
A-1331852+MK-0752 10 nM	102.40	83.88	60.12	95.84	88.86	102.31	86.83	101.47
A-1331852+MK-0752 100 nM	97.84	81.98	57.96	94.65	87.69	103.73	81.51	92.43
A-1331852+MK-0752 1000 nM	96.85	80.89	62.98	94.08	85.94	99.76	84.65	96.31
A-1331852+MK-0752 10000 nM	96.48	70.51	45.51	91.62	75.04	96.37	76.45	90.00
A-1331852+MK-0752 20000 nM	95.41	67.73	41.88	89.20	72.18	95.02	74.09	84.30
A-1331852+Nirogacestat 0.1 nM	100.10	82.56	64.52	92.86	90.49	103.32	84.70	94.85
A-1331852+Nirogacestat 1 nM	103.08	81.46	63.73	95.30	95.45	106.13	88.07	101.40
A-1331852+Nirogacestat 10 nM	99.89	83.14	60.96	92.78	87.59	105.80	84.40	94.99
A-1331852+Nirogacestat 100 nM	96.66	79.65	59.03	95.60	86.05	101.99	82.09	95.25
A-1331852+Nirogacestat 1000 nM	99.12	74.05	52.68	93.87	84.18	102.98	82.84	92.14
A-1331852+Nirogacestat 10000 nM	81.27	50.10	30.30	81.34	75.75	99.21	58.23	67.16
A-1331852+Nirogacestat 20000 nM	51.46	29.78	10.92	59.80	62.10	91.91	44.81	48.29
Carboplatin	98.28	86.67	88.05	89.71	96.49	94.60	85.17	82.56
Carboplatin+A-1331852 0.1 nM	97.99	88.76	85.02	91.05	98.01	92.31	83.42	85.00
Carboplatin+A-1331852 1 nM	97.46	85.85	82.93	88.67	96.55	92.05	80.43	78.47
Carboplatin+A-1331852 10 nM	93.63	78.00	64.48	86.95	96.40	92.47	75.48	74.28
Carboplatin+A-1331852 100 nM	92.19	64.56	28.36	83.72	90.84	91.05	73.80	62.63
Carboplatin+A-1331852 1000 nM	85.59	37.72	10.30	79.57	85.37	86.72	65.61	46.11
Carboplatin+MK-0752 0.1 nM	100.16	86.72	88.04	89.62	96.74	93.33	81.73	81.73
Carboplatin+MK-0752 1 nM	96.12	83.86	81.65	89.03	97.22	91.15	79.73	80.12
Carboplatin+MK-0752 10 nM	99.68	87.72	86.03	89.79	97.61	92.98	84.26	86.70
Carboplatin+MK-0752 100 nM	96.44	84.54	88.39	90.60	95.00	90.91	78.84	81.47
Carboplatin+MK-0752 1000 nM	94.05	84.04	84.28	92.10	91.96	90.86	80.70	83.36
Carboplatin+MK-0752 10000 nM	95.71	82.07	81.96	87.89	78.81	86.36	77.84	81.26
Carboplatin+MK-0752 20000 nM	94.31	79.00	81.19	85.43	78.82	83.80	76.01	78.83
Carboplatin+Nirogacestat 0.1 nM	96.75	84.90	86.04	91.05	97.86	94.08	82.30	83.75
Carboplatin+Nirogacestat 1 nM	99.35	87.63	85.47	91.93	98.13	95.79	83.90	87.28
Carboplatin+Nirogacestat 10 nM	98.27	86.96	89.55	90.36	91.95	93.15	81.92	80.53
Carboplatin+Nirogacestat 100 nM	95.07	84.20	84.78	93.41	89.78	91.39	80.33	83.90
Carboplatin+Nirogacestat 1000 nM	99.58	83.84	84.72	92.83	87.72	93.15	78.46	84.49
Carboplatin+Nirogacestat 10000 nM	86.13	79.09	78.56	84.07	82.69	89.74	67.20	78.82
Carboplatin+Nirogacestat 20000 nM	64.32	64.76	59.96	68.36	73.60	87.12	59.60	70.43
MK-0752	101.65	97.82	97.98	100.80	91.95	97.86	95.08	108.55
Nirogacestat	97.37	94.21	93.30	95.10	88.70	99.92	87.70	102.40
DMSO	98.20	97.09	94.79	97.01	97.74	95.16	94.06	100.70

Supplemental Table 8

Model_name	Passage at drug testing & innitial characterization
**Gem017-T**	P7
**Gem034-T1**	P6
**Gem046-T**	P5
**Gem061-A**	P8
**Gem062-T**	P8
**Gem096-A**	P4
**OV015-T**	NA
**OV022-A**	NA
**OV022-T1**	NA
**OV022-T2**	NA
**OV024-A**	P7
**OV026-PDF**	P4
**OV026-T**	P9
**OV027-PDF**	P9
**OV027-PDF-R**	P9
**OV027-T**	P8
**OV030-A2**	P8
**OV030-A3**	P8
**OV030-PDF**	P7
**OV030-PDF-R**	P10
**OV030-R**	P8
**OV030-S**	P8
**OV031-PDF**	P8
**OV031-PDF-R**	P12
**OV032-PDF**	P6
**OV035-PDF**	P5
**OV038-PDF**	P5
**OV039-PDF**	P5
**OV039-PDF-R**	P9
**OV042-A**	P8
**OV042-PDF**	P5
**OV042-T**	P8
**OV042-T2**	P8
**OV044-PDF**	P5
**OV048-PDF**	P9
**OV048-PDF-R**	P8
**OV048-PDF.T**	P9
**OV050-PDF**	P5
**OV051-PDF**	P5

Supplemental Table 9

Assay	Antibody	Manufacturer	Cat. No	Dilution
Immunofluorescent staining	aSMA-Alexa647	Abcam	ab32575	1:100
	ALDH1A1	Abcam	ab52492	1:125
	CD44	Invitrogen	MA5-13890	1:100
	CK8/18	Agilent/Dako	M3652	1:300
	FSP1	Agilent/Dako	A5114	1:100
	HES1	Abcam	ab108937	1:250
	MUC16	Invitrogen	MA5-11202	1:100
	PAX8	Proteintech	10336-1-AP	1:100
	Vimentin	Invitrogen	MA5-11883	1:300
	Donkey anti-Mouse Secondary Antibody, Alexa Fluor™ 488	Invitrogen	A21202	1:2000
	Donkey anti-Mouse Secondary Antibody, Alexa Fluor™ 568	Invitrogen	A10037	1:2000
	Donkey anti-Rabbit Secondary Antibody, Alexa Fluor™ 488	Invitrogen	A21206	1:2000
	Donkey anti-Rabbit Secondary Antibody, Alexa Fluor™ 568	Invitrogen	A10042	1:2000
Western Blot	Bcl-xL	Cell Signaling	#2764	1:1000
	Vinculin	Invitrogen	700062	1:1000
	beta-tubulin	Cell Signaling	#86298	1:1000
	Donkey anti-Rabbit IgG (H+L) Highly Cross-Adsorbed Secondary Antibody, HRP	Invitrogen	A16035	1:10000
	Donkey anti-Mouse IgG (H+L) Highly Cross-Adsorbed Secondary Antibody, HRP	Invitrogen	A16017	1:10000

Supplemental Table 10
Drug screen against 528 drugs

Type	PatientID	Model	#plate	Z-prime
	OV015	Tissue PDC	1	0.59
		Tissue PDC	2	0.71
	OV022	Ascites PDC	1	0.81
		Ascites PDC	2	0.8
		Ascites PDC	3	0.8
		Ascites PDC	4	0.88
		Ascites PDC	5	0.85
		Ascites PDC	6	0.79
		Ascites PDC	7	0.82
		Ascites PDC	8	0.82
	OV022	Tissue DMEM PDC	1	0.83
		Tissue DMEM PDC	2	0.81
		Tissue DMEM PDC	3	0.87
		Tissue DMEM PDC	4	0.84
		Tissue DMEM PDC	5	0.82
		Tissue DMEM PDC	6	0.84
		Tissue DMEM PDC	7	0.84
		Tissue DMEM PDC	8	0.71
	OV022	Tissue Rockit PDC	1	0.79
		Tissue Rockit PDC	2	0.8
		Tissue Rockit PDC	3	0.18
		Tissue Rockit PDC	4	0.85
		Tissue Rockit PDC	5	0.85
		Tissue Rockit PDC	6	0.88
		Tissue Rockit PDC	7	0.88
		Tissue Rockit PDC	8	0.77
	OV024	Ascites PDC	1	0.78
		Ascites PDC	2	0.81
		Ascites PDC	3	0.86
		Ascites PDC	4	0.78
		Ascites PDC	5	0.84
		Ascites PDC	6	0.83
		Ascites PDC	7	0.83
		Ascites PDC	8	0.8
	OvCa026	Tissue PDC	1	0.85
		Tissue PDC	2	0.81
		Tissue PDC	3	0.7
		Tissue PDC	4	0.6
		Tissue PDC	5	0.81
		Tissue PDC	6	0.83
		Tissue PDC	7	0.78
		Tissue PDC	8	0.78
	OvCa026	Tissue PDF in Fibroblasts	1	0.72
		Tissue PDF in Fibroblasts	2	0.73
		Tissue PDF in Fibroblasts	3	0.74
		Tissue PDF in Fibroblasts	4	0.79
		Tissue PDF in Fibroblasts	5	0.72
		Tissue PDF in Fibroblasts	6	0.75
		Tissue PDF in Fibroblasts	7	0.52
		Tissue PDF in Fibroblasts	8	0.7
	OV027	Tissue PDC	1	0.77
		Tissue PDC	2	0.84
		Tissue PDC	3	0.84
		Tissue PDC	4	0.85
		Tissue PDC	5	0.76

	Tissue PDC	6	0.81
	Tissue PDC	7	0.83
	Tissue PDC	8	0.85
OV027	Tissue PDF in Fibroblasts	1	0.73
	Tissue PDF in Fibroblasts	2	0.73
	Tissue PDF in Fibroblasts	3	0.79
	Tissue PDF in Fibroblasts	4	0.8
	Tissue PDF in Fibroblasts	5	0.73
	Tissue PDF in Fibroblasts	6	0.77
	Tissue PDF in Fibroblasts	7	0.73
	Tissue PDF in Fibroblasts	8	0.68
OV030	Ascites resistant to Bcl-xL inhibitors	1	0.7
	Ascites resistant to Bcl-xL inhibitors	2	0.79
	Ascites resistant to Bcl-xL inhibitors	3	0.81
	Ascites resistant to Bcl-xL inhibitors	4	0.7
	Ascites resistant to Bcl-xL inhibitors	5	0.69
	Ascites resistant to Bcl-xL inhibitors	6	0.64
	Ascites resistant to Bcl-xL inhibitors	7	0.74
	Ascites resistant to Bcl-xL inhibitors	8	0.74
OV030	Ascites model A2	1	0.82
	Ascites model A2	2	0.79
	Ascites model A2	3	0.76
	Ascites model A2	4	0.79
	Ascites model A2	5	0.79
	Ascites model A2	6	0.77
	Ascites model A2	7	0.79
	Ascites model A2	8	0.79
OV030	Ascites model A3	1	0.77
	Ascites model A3	2	0.79
	Ascites model A3	3	0.76
	Ascites model A3	4	0.56
	Ascites model A3	5	0.68
	Ascites model A3	6	0.74
	Ascites model A3	7	0.82
	Ascites model A3	8	0.62
OV030	Ascites sensitive to Bcl-xL inhibitors	1	0.7
	Ascites sensitive to Bcl-xL inhibitors	2	0.8
	Ascites sensitive to Bcl-xL inhibitors	3	0.63
	Ascites sensitive to Bcl-xL inhibitors	4	0.79
	Ascites sensitive to Bcl-xL inhibitors	5	0.82
	Ascites sensitive to Bcl-xL inhibitors	6	0.78
	Ascites sensitive to Bcl-xL inhibitors	7	0.66
	Ascites sensitive to Bcl-xL inhibitors	8	0.74
OV030	Tissue PDF in Fibroblasts	1	0.79
	Tissue PDF in Fibroblasts	2	0.87
	Tissue PDF in Fibroblasts	3	0.82
	Tissue PDF in Fibroblasts	4	0.77
	Tissue PDF in Fibroblasts	5	0.83
	Tissue PDF in Fibroblasts	6	0.78
	Tissue PDF in Fibroblasts	7	0.78
	Tissue PDF in Fibroblasts	8	0.81
OV031	Tissue PDF in Fibroblasts	1	0.81
	Tissue PDF in Fibroblasts	2	0.8
	Tissue PDF in Fibroblasts	3	0.72
	Tissue PDF in Fibroblasts	4	0.82
	Tissue PDF in Fibroblasts	5	0.79
	Tissue PDF in Fibroblasts	6	0.81
	Tissue PDF in Fibroblasts	7	0.7
	Tissue PDF in Fibroblasts	8	0.74

PDM

OV032	Tissue PDF in Fibroblasts	1	0.79
	Tissue PDF in Fibroblasts	2	0.84
	Tissue PDF in Fibroblasts	3	0.79
	Tissue PDF in Fibroblasts	4	0.75
	Tissue PDF in Fibroblasts	5	0.84
	Tissue PDF in Fibroblasts	6	0.82
	Tissue PDF in Fibroblasts	7	0.83
	Tissue PDF in Fibroblasts	8	0.82
OV035	Tissue PDF (from Bpart) in Fibroblasts	1	0.83
	Tissue PDF (from Bpart) in Fibroblasts	2	0.87
	Tissue PDF (from Bpart) in Fibroblasts	3	0.83
	Tissue PDF (from Bpart) in Fibroblasts	4	0.87
	Tissue PDF (from Bpart) in Fibroblasts	5	0.87
	Tissue PDF (from Bpart) in Fibroblasts	6	0.84
	Tissue PDF (from Bpart) in Fibroblasts	7	0.8
	Tissue PDF (from Bpart) in Fibroblasts	8	0.85
OV035	Tissue PDF (from Apart) in Fibroblasts	1	0.82
	Tissue PDF (from Apart) in Fibroblasts	2	0.78
	Tissue PDF (from Apart) in Fibroblasts	3	0.72
	Tissue PDF (from Apart) in Fibroblasts	4	0.71
	Tissue PDF (from Apart) in Fibroblasts	5	0.77
	Tissue PDF (from Apart) in Fibroblasts	6	0.82
	Tissue PDF (from Apart) in Fibroblasts	7	0.75
	Tissue PDF (from Apart) in Fibroblasts	8	0.75
OV038	Tissue PDF (from No2) in Fibroblasts	1	0.74
	Tissue PDF (from No2) in Fibroblasts	2	0.89
	Tissue PDF (from No2) in Fibroblasts	3	0.84
	Tissue PDF (from No2) in Fibroblasts	4	0.86
	Tissue PDF (from No2) in Fibroblasts	5	0.74
	Tissue PDF (from No2) in Fibroblasts	6	0.79
	Tissue PDF (from No2) in Fibroblasts	7	0.79
	Tissue PDF (from No2) in Fibroblasts	8	0.82
OV038	Tissue PDF (from No1) in Fibroblasts	1	0.84
	Tissue PDF (from No1) in Fibroblasts	2	0.8
	Tissue PDF (from No1) in Fibroblasts	3	0.71
	Tissue PDF (from No1) in Fibroblasts	4	-0.15
	Tissue PDF (from No1) in Fibroblasts	5	0.75
	Tissue PDF (from No1) in Fibroblasts	6	0.79
	Tissue PDF (from No1) in Fibroblasts	7	0.8
	Tissue PDF (from No1) in Fibroblasts	8	0.78
OV038	Tissue PDF (from Adex) in Fibroblasts	1	0.82
	Tissue PDF (from Adex) in Fibroblasts	2	0.85
	Tissue PDF (from Adex) in Fibroblasts	3	0.78
	Tissue PDF (from Adex) in Fibroblasts	4	0.83
	Tissue PDF (from Adex) in Fibroblasts	5	0.84
	Tissue PDF (from Adex) in Fibroblasts	6	0.81
	Tissue PDF (from Adex) in Fibroblasts	7	0.8
	Tissue PDF (from Adex) in Fibroblasts	8	0.83
OV039	Tissue PDF in Fibroblasts	1	0.86
	Tissue PDF in Fibroblasts	2	0.87
	Tissue PDF in Fibroblasts	3	0.84
	Tissue PDF in Fibroblasts	4	0.85
	Tissue PDF in Fibroblasts	5	0.85
	Tissue PDF in Fibroblasts	6	0.85
	Tissue PDF in Fibroblasts	7	0.88
	Tissue PDF in Fibroblasts	8	0.79
OV042	Tissue PDC	1	0.82
	Tissue PDC	2	0.87
	Tissue PDC	3	0.88

	Tissue PDC	4	0.8
	Tissue PDC	5	0.78
	Tissue PDC	6	0.87
	Tissue PDC	7	0.83
	Tissue PDC	8	0.64
OV042	Ascites PDC	1	0.84
	Ascites PDC	2	0.82
	Ascites PDC	3	0.81
	Ascites PDC	4	0.84
	Ascites PDC	5	0.85
	Ascites PDC	6	0.86
	Ascites PDC	7	0.78
	Ascites PDC	8	0.82
OV042	Tissue (SM) PDC	1	0.79
	Tissue (SM) PDC	2	0.79
	Tissue (SM) PDC	3	0.81
	Tissue (SM) PDC	4	0.81
	Tissue (SM) PDC	5	0.81
	Tissue (SM) PDC	6	0.79
	Tissue (SM) PDC	7	0.79
	Tissue (SM) PDC	8	0.77
OV042	Tissue PDF in Fibroblasts	1	0.9
	Tissue PDF in Fibroblasts	2	0.83
	Tissue PDF in Fibroblasts	3	0.85
	Tissue PDF in Fibroblasts	4	-0.15
	Tissue PDF in Fibroblasts	5	0.81
	Tissue PDF in Fibroblasts	6	0.86
	Tissue PDF in Fibroblasts	7	0.87
	Tissue PDF in Fibroblasts	8	0.86
OV044	Tissue PDF (from pelvis) in Fibroblasts	1	0.84
	Tissue PDF (from pelvis) in Fibroblasts	2	0.77
	Tissue PDF (from pelvis) in Fibroblasts	3	0.82
	Tissue PDF (from pelvis) in Fibroblasts	4	0.81
	Tissue PDF (from pelvis) in Fibroblasts	5	0.82
	Tissue PDF (from pelvis) in Fibroblasts	6	0.78
	Tissue PDF (from pelvis) in Fibroblasts	7	0.8
	Tissue PDF (from pelvis) in Fibroblasts	8	0.77
OV048	Tissue PDF in Fibroblasts	1	0.84
	Tissue PDF in Fibroblasts	2	0.79
	Tissue PDF in Fibroblasts	3	0.88
	Tissue PDF in Fibroblasts	4	0.89
	Tissue PDF in Fibroblasts	5	0.84
	Tissue PDF in Fibroblasts	6	0.82
	Tissue PDF in Fibroblasts	7	0.83
	Tissue PDF in Fibroblasts	8	0.84
OV048	Ascites PDF in Fibroblasts	1	0.74
	Ascites PDF in Fibroblasts	2	0.82
	Ascites PDF in Fibroblasts	3	0.78
	Ascites PDF in Fibroblasts	4	0.82
	Ascites PDF in Fibroblasts	5	0.8
	Ascites PDF in Fibroblasts	6	0.81
	Ascites PDF in Fibroblasts	7	0.74
	Ascites PDF in Fibroblasts	8	0.8
OV051	Tissue PDF in Fibroblasts	1	0.86
	Tissue PDF in Fibroblasts	2	0.87
	Tissue PDF in Fibroblasts	3	0.77
	Tissue PDF in Fibroblasts	4	0.84
	Tissue PDF in Fibroblasts	5	0.83
	Tissue PDF in Fibroblasts	6	0.85

	Tissue PDF in Fibroblasts	7	0.87
	Tissue PDF in Fibroblasts	8	0.8
OV050	Tissue PDF in Fibroblasts	1	0.76
	Tissue PDF in Fibroblasts	2	0.83
	Tissue PDF in Fibroblasts	3	0.82
	Tissue PDF in Fibroblasts	4	0.84
	Tissue PDF in Fibroblasts	5	0.72
	Tissue PDF in Fibroblasts	6	0.84
	Tissue PDF in Fibroblasts	7	0.81
	Tissue PDF in Fibroblasts	8	0.8
Gem017	Ascites PDC	1	0.85
	Ascites PDC	2	0.82
	Ascites PDC	3	0.83
Gem034	Tissue PDC	1	0.47
	Tissue PDC	2	0.89
	Tissue PDC	3	0.75
	Tissue PDC	4	0.91
	Tissue PDC	5	0.86
	Tissue PDC	6	0.36
	Tissue PDC	7	0.84
	Tissue PDC	8	0.83
Gem046	Ascites PDC	1	0.63
	Ascites PDC	2	0.78
	Ascites PDC	3	0.9
	Ascites PDC	4	0.88
	Ascites PDC	5	0.82
	Ascites PDC	6	0.81
	Ascites PDC	7	0.91
	Ascites PDC	8	0.79
Gem061	Ascites PDC	1	0.41
	Ascites PDC	2	0.86
	Ascites PDC	3	0.88
	Ascites PDC	4	0.91
	Ascites PDC	5	0.81
	Ascites PDC	6	0.92
	Ascites PDC	7	0.88
	Ascites PDC	8	0.88
Gem062	Tissue PDC	1	0.52
	Tissue PDC	2	0.87
	Tissue PDC	3	0.71
	Tissue PDC	4	0.86
	Tissue PDC	5	0.84
	Tissue PDC	6	0.84
	Tissue PDC	7	0.81
	Tissue PDC	8	0.79
Gem096	Ascites PDC	1	0.74
	Ascites PDC	2	0.71
	Ascites PDC	3	0.7
	Ascites PDC	4	0.71
	Ascites PDC	5	0.65
	Ascites PDC	6	0.65
	Ascites PDC	7	0.61
OV026	Tissue PDF in Rockit	1	0.78
	Tissue PDF in Rockit	2	0.61
	Tissue PDF in Rockit	3	0.71
	Tissue PDF in Rockit	4	0.71
	Tissue PDF in Rockit	5	0.71
	Tissue PDF in Rockit	6	0.64
	Tissue PDF in Rockit	7	0.7

		Tissue PDF in Rockit	8	0.79
	OV030	Tissue PDF in Rockit	1	0.87
		Tissue PDF in Rockit	2	0.9
		Tissue PDF in Rockit	3	0.88
		Tissue PDF in Rockit	4	0.88
		Tissue PDF in Rockit	5	0.89
		Tissue PDF in Rockit	6	0.9
		Tissue PDF in Rockit	7	0.9
		Tissue PDF in Rockit	8	0.96
	OV031	Tissue PDF in Rockit	1	0.85
		Tissue PDF in Rockit	2	0.67
		Tissue PDF in Rockit	3	0.81
		Tissue PDF in Rockit	4	0.85
		Tissue PDF in Rockit	5	0.76
		Tissue PDF in Rockit	6	0.85
		Tissue PDF in Rockit	7	0.52
		Tissue PDF in Rockit	8	0.69
	OV039	Tissue PDF in Rockit	1	0.86
		Tissue PDF in Rockit	2	0.92
		Tissue PDF in Rockit	3	0.78
		Tissue PDF in Rockit	4	0.9
		Tissue PDF in Rockit	5	0.87
		Tissue PDF in Rockit	6	0.84
		Tissue PDF in Rockit	7	0.84
		Tissue PDF in Rockit	8	0.88
	OV048	Tissue PDF in Rockit	1	0.84
		Tissue PDF in Rockit	2	0.91
		Tissue PDF in Rockit	3	0.88
		Tissue PDF in Rockit	4	0.9
		Tissue PDF in Rockit	5	0.89
		Tissue PDF in Rockit	6	0.86
		Tissue PDF in Rockit	7	0.83
		Tissue PDF in Rockit	8	0.88
BM	BM-1690	Bone marrow	1	0.75
		Bone marrow	2	0.79
		Bone marrow	3	0.81
		Bone marrow	4	0.82
		Bone marrow	5	0.82
		Bone marrow	6	0.83
		Bone marrow	7	0.81
		Bone marrow	8	0.76
	BM-1691	Bone marrow	1	0.78
		Bone marrow	2	0.84
		Bone marrow	3	0.73
		Bone marrow	4	0.84
		Bone marrow	5	0.67
		Bone marrow	6	0.8
		Bone marrow	7	0.72
		Bone marrow	8	0.84
		Bone marrow	1	0.86
	A-2780	CCLs	2	0.85
		CCLs	3	0.87
		CCLs	4	0.79
		CCLs	5	0.83
		CCLs	6	0.83
		CCLs	7	0.87
		CCLs	8	0.64
	EFO-21	CCLs	1	0.81
		CCLs	2	0.81

CCLs

	CCLs	3	0.77
	CCLs	4	0.83
	CCLs	5	0.82
	CCLs	6	0.84
	CCLs	7	0.88
	CCLs	8	0.88
KURAMOCHI	CCLs	1	0.86
	CCLs	2	0.89
	CCLs	3	0.82
	CCLs	4	0.84
	CCLs	5	0.88
	CCLs	6	0.85
	CCLs	7	0.81
	CCLs	8	0.93
MH	CCLs	1	0.78
	CCLs	2	0.78
	CCLs	3	0.79
	CCLs	4	0.76
	CCLs	5	0.69
	CCLs	6	0.77
	CCLs	7	0.81
	CCLs	8	0.81
NCI-ADR-RES	CCLs	1	0.53
	CCLs	2	0.54
	CCLs	3	0.63
	CCLs	4	0.31
	CCLs	5	0.42
	CCLs	6	0.62
	CCLs	7	0.57
	CCLs	8	0.5
OAW-28	CCLs	1	0.85
	CCLs	2	0.8
	CCLs	3	0.8
	CCLs	4	0.81
	CCLs	5	0.81
	CCLs	6	0.85
	CCLs	7	0.85
	CCLs	8	0.79
ONCODG-1	CCLs	1	0.84
	CCLs	2	0.74
	CCLs	3	0.83
	CCLs	4	0.82
	CCLs	5	0.81
	CCLs	6	0.82
	CCLs	7	0.88
	CCLs	8	0.84
OVCAR3	CCLs	1	0.84
	CCLs	2	0.88
	CCLs	3	0.81
	CCLs	4	0.76
	CCLs	5	0.74
	CCLs	6	0.72
	CCLs	7	0.61
	CCLs	8	0.7
OVCAR4	CCLs	1	0.77
	CCLs	2	0.8
	CCLs	3	0.8
	CCLs	4	0.81
	CCLs	5	0.79

		CCLs	6	0.82
		CCLs	7	0.8
		CCLs	8	0.65
	OVCAR8	CCLs	1	0.85
		CCLs	2	0.85
		CCLs	3	0.8
		CCLs	4	0.8
		CCLs	5	0.82
		CCLs	6	0.83
		CCLs	7	0.85
		CCLs	8	0.77
		CCLs	1	0.81
	OVSAHO	CCLs	2	0.81
		CCLs	3	0.84
		CCLs	4	0.82
		CCLs	5	0.86
		CCLs	6	0.8
		CCLs	7	0.77
		CCLs	8	0.79
	OVTOKO	CCLs	1	0.59
		CCLs	2	0.59
		CCLs	3	0.61
		CCLs	4	0.59
		CCLs	5	0.61
		CCLs	6	0.59
		CCLs	7	0.67
		CCLs	8	0.64
	TYKNU	CCLs	1	0.83
		CCLs	2	0.79
		CCLs	3	0.85
		CCLs	4	0.77
		CCLs	5	0.8
		CCLs	6	0.81
		CCLs	7	0.77
		CCLs	8	0.83
	CAOV3	CCLs	1	0.81
		CCLs	2	0.85
		CCLs	3	0.82
		CCLs	4	0.8
		CCLs	5	0.85
		CCLs	6	0.86
		CCLs	7	0.87
		CCLs	8	0.56
	COV362	CCLs	1	0.79
		CCLs	2	0.82
		CCLs	3	0.83
		CCLs	4	0.8
		CCLs	5	0.86
		CCLs	6	0.83
		CCLs	7	0.77
		CCLs	8	0.82

Supplemental Data 10

Validation drug screening

Model	corresponding figures	plate	Z-prime
OV030-200	Fig. 3C, Sup. Fig. 3C	24h	0.8
OV030-200	Fig. 3C, Sup. Fig. 3C	72h	0.75
OV030-70	Fig. 3C, Sup. Fig. 3C	24h	0.54
OV030-70	Fig. 3C, Sup. Fig. 3C	72h	0.66
OV030.A3	Fig. 3C, Sup. Fig. 3C	24h	0.61
OV030.A3	Fig. 3C, Sup. Fig. 3C	72h	0.6
OV030.A2	Fig. 3C, Sup. Fig. 3C	24h	0.85
OV030.A2	Fig. 3C, Sup. Fig. 3C	72h	0.58
OV030-200	Fig.5B-D & Sup. Fig. 5B-C	A133	0.78
OV030-200	Fig.5B-D & Sup. Fig. 5B-C	Carboplatin	0.83
OV030-70	Fig.5B-D & Sup. Fig. 5B-C	A133	0.70
OV030-70	Fig.5B-D & Sup. Fig. 5B-C	Carboplatin	0.69
OV039-PDF	Fig.5B-D & Sup. Fig. 5B-C	A133	NA
OV039-PDF	Fig.5B-D & Sup. Fig. 5B-C	Carboplatin	NA
OV031-PDF	Fig.5B-D & Sup. Fig. 5B-C	A133	NA
OV031-PDF	Fig.5B-D & Sup. Fig. 5B-C	Carboplatin	
Gem061.A	Fig.5B-D & Sup. Fig. 5B-C	A133	0.79
Gem061.A	Fig.5B-D & Sup. Fig. 5B-C	Carboplatin	0.78
OV024.A	Fig.5B-D & Sup. Fig. 5B-C	A133	NA
OV024.A	Fig.5B-D & Sup. Fig. 5B-C	Carboplatin	NA
OVSAHO	Fig.5B-D & Sup. Fig. 5B-C	A133	0.79
OVSAHO	Fig.5B-D & Sup. Fig. 5B-C	Carboplatin	0.79
OVCAR4	Fig.5B-D & Sup. Fig. 5B-C	A133	NA
OVCAR4	Fig.5B-D & Sup. Fig. 5B-C	Carboplatin	NA
KURAMOCHI	Fig.5B-D & Sup. Fig. 5B-C	A133	NA
KURAMOCHI	Fig.5B-D & Sup. Fig. 5B-C	Carboplatin	NA
OV022.T2	Fig.5B-D & Sup. Fig. 5B-C	A133	0.79
OV022.T2	Fig.5B-D & Sup. Fig. 5B-C	Carboplatin	0.77
OV030-200	Fig. 5F,G	DT2216	0.54
OV030-70	Fig. 5F,G	DT2216	0.79
OV039-PDF	Fig. 5F,G	DT2216	0.60
MOLT4	Sup. Fig. 5D	DT2216	0.80

ex vivo cultures

PatientID	day	Z-prime
Gem031	3	0.6623
Gem0243	3	0.3232
Gem0232	3	0.8736

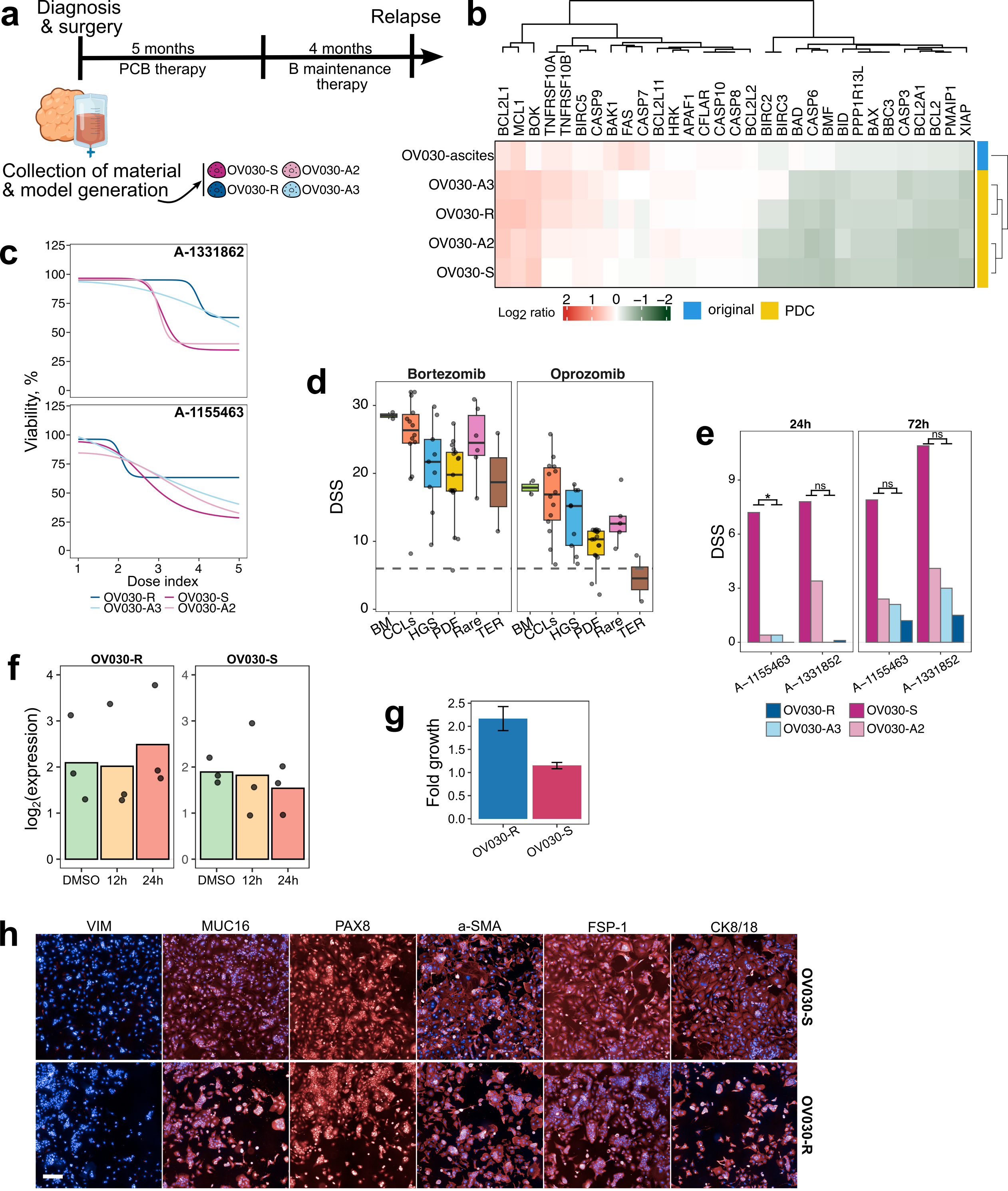

Supplementary Figure 3. a – overview of OV030 patient clinical timeline. Here P indicates Paclitaxel, C – Carboplatin and B – Bevacizumab. **b** – CNV profile of apoptosis-related genes in OV030 ascites cells and OV030 PDCs. **c** – Drug response curves of four OV030 patient PDCs with Bcl-xL inhibitors A-1331852 and A-1155463, the x-axis represents index-scaled concentrations, ranging from 0.1-1 uM for A-1331852 and 1-10 uM for A-1155463. **d** – DSS values for two protease/proteasome inhibitors across all tested cell models. Dashed lines denote DSS=6. **e** – Bar plot representing DSS values for A-1331852 across all OV030 PDCs after 24 and 72 h of treatment. *p<0.05 from Mann-Whitney U test (n=2). These results guided the selection of A-1331852 concentration for subsequent MS-proteomics analysis. **f** – Protein expression profiles in OV030-R and OV030-S models with and without A-1331852 treatment, shown as log2 fold change normalized to house-keeping proteins. Each dot represents an independent biological replicate. **g** – Comparison of cell growth between OV030-R and OV030-S after 72 h of culture, error bars indicate mean ± SD. **h** – epresentative IF images illustrating expression of OC and TME-associated markers. Scale bar = 200 um.

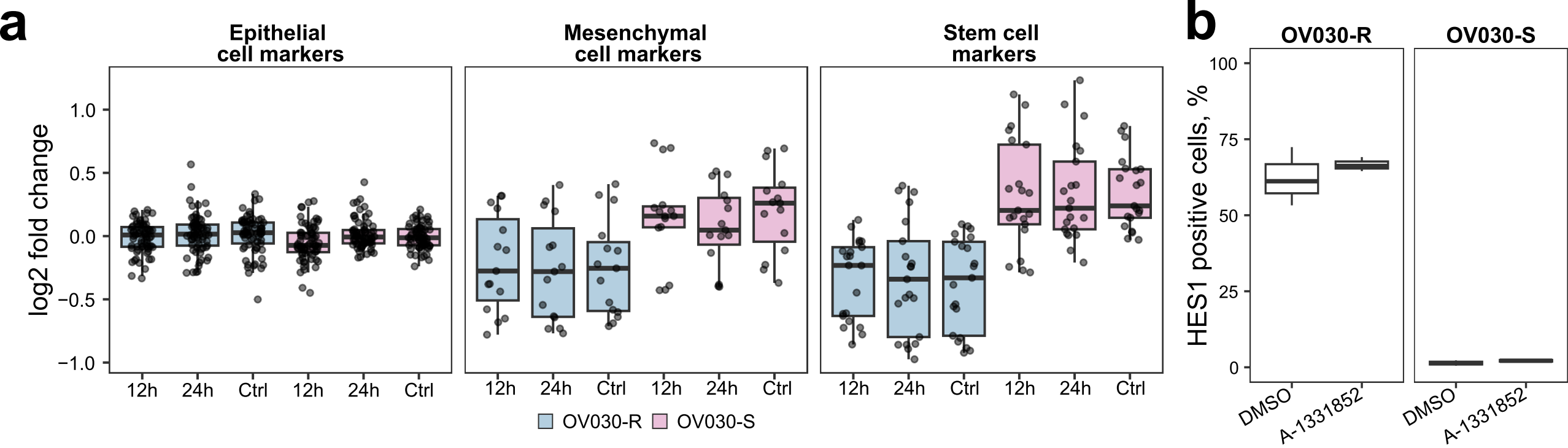

Supplementary Figure 4. a – Expression of EMT signature proteins and CSC-associated markers in OV030-R and OV03-S models, with and without A-1331852 treatment. Expression is shown as log2 fold change. **b** – IF based HES1 quantification of HES1 nuclear expression in OV030-R and OV030-S clones after 24 h in culture with and without A-1331852 treatment.

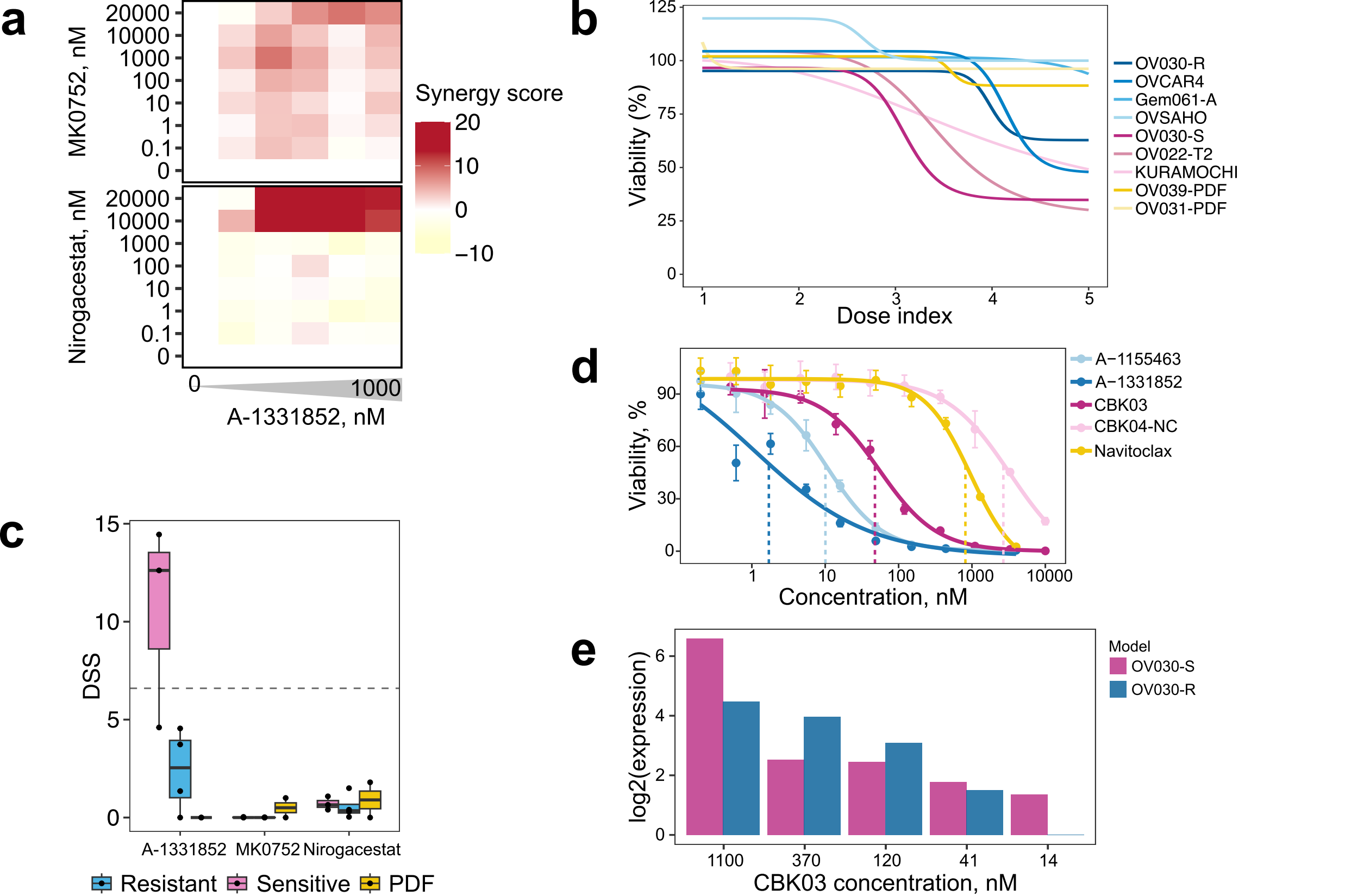

Supplementary Figure 5. a – Synergy score heatmaps for A-1331852 and GSIs combination treatment in the OV030-S model. **b** – Drug response curves following 72h of A-1331852 treatment. Blue curves indicate A-1331852 resistant cancer cell models, pink denote A-1331852 sensitive models, and yellow represent PDFs. **c** – Cell response to monotherapy of drugs used in combination treatment. Dashed lines denote DSS=6. **d** – Drug response curves of MOLT-4 cell treated with Bcl-xL targeting drugs. Dotted lines represent EC50 values, ± SD (n=3 for each condition). **e** – Protein expression profiles in OV030-R and OV030-S models at different CBK03 concentrations, shown as log2 fold change normalized to house-keeping proteins.

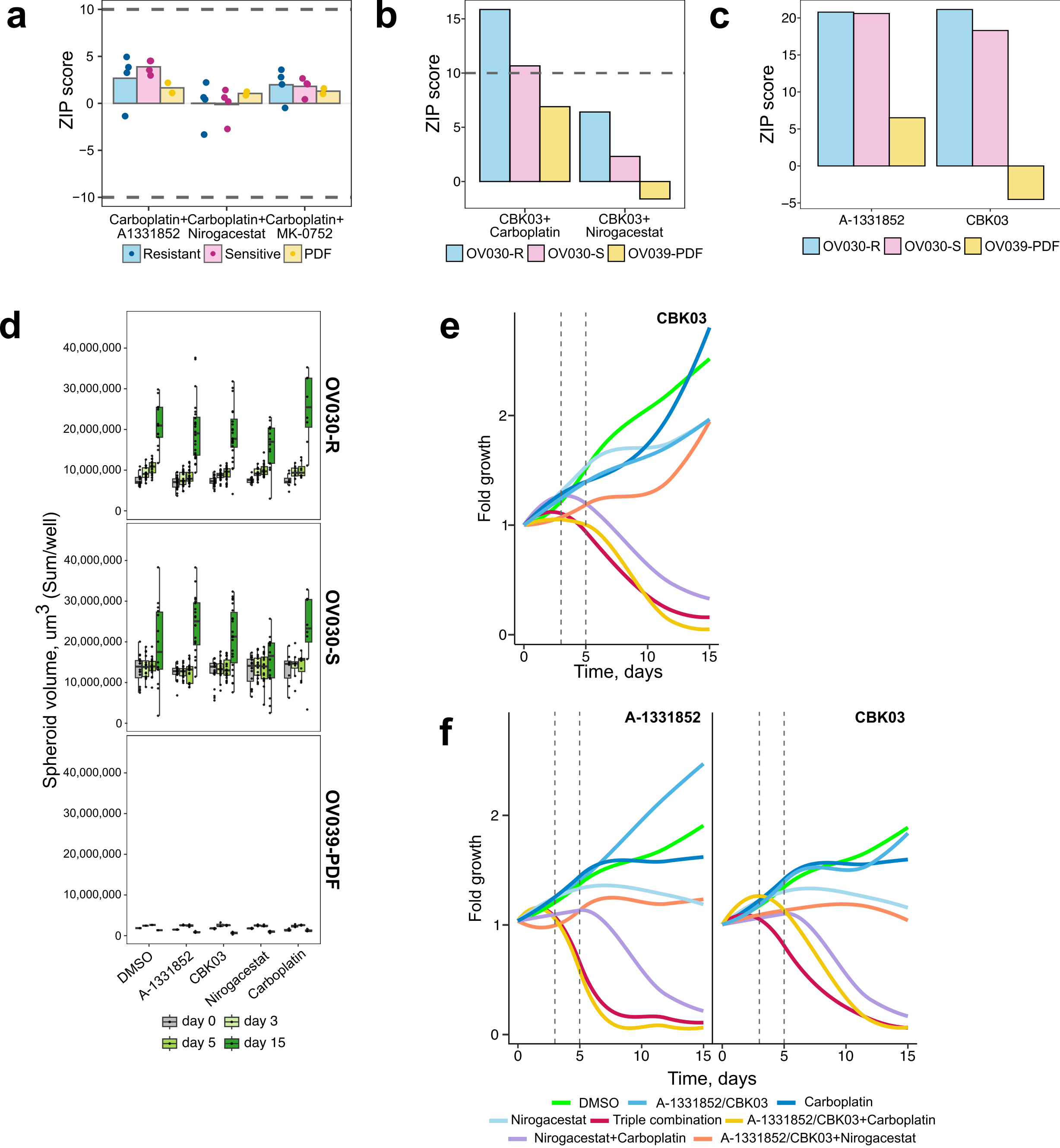

Supplementary Figure 6. a – Synergy score heatmaps for A-1331852 and GSIs combination treatment in the OV030-S model. **b** – ZIP synergy scores visualized as bar plots for CBK03 and GSIs combinations. **c** – ZIP synergy scores displayed as bar plots for A-1331852 and CBK03 based triple drug combinations for OV030-R, OV030-S and OV039-PDF. **d** – Boxplots representing spheroid volume change treated with monotherapy. Colors indicate time point: baseline (before treatment), and at 3, 5 and 15 days after treatment. Here drug exposure lasted for five days, after which treatment was removed. **e** – Longitudinal response of OV030-R to the highest tested concentrations of single and combination CBK03-based treatments across the 15-day assay. f – Longitudinal response of OV030-S to the highest tested concentrations of single and combination treatments across the 15-day assay.

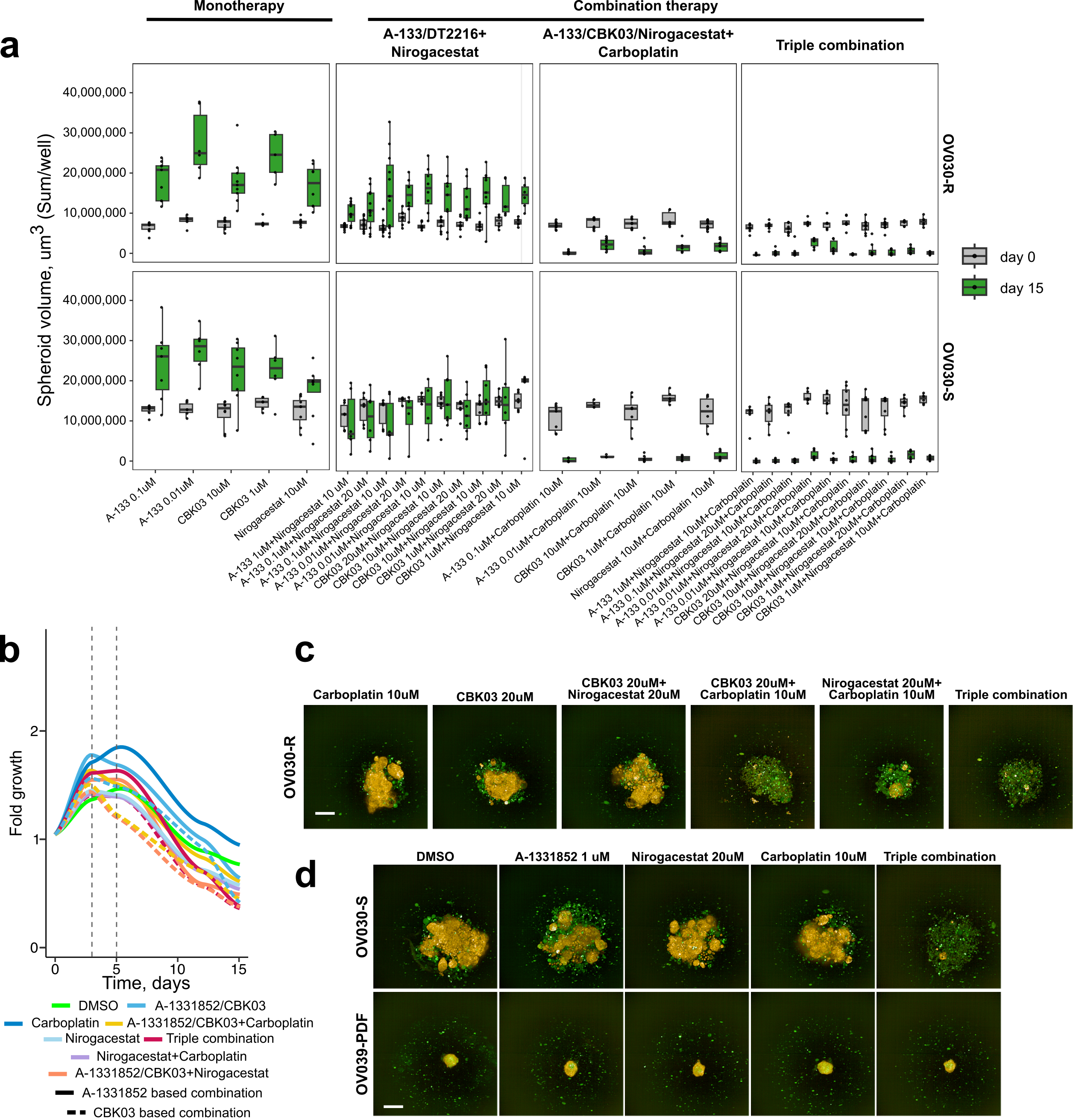

Supplementary Figure 7. a – Boxplots representing drug response of OV030-R and OV030-S models to lower tested concentrations of single and combination treatments, comparing spheroid volume at first and last time point of the assay. **b** – Longitudinal response of OV039-PDF response to the highest tested concentration of single and combination treatments across the 15-day assay. **c, d** – Representative images showing spheroid size and viability afterday 15 of the assay, with and without treatment. Green signal marks dead, yellow indicates live cells, scale bar is 200 um.

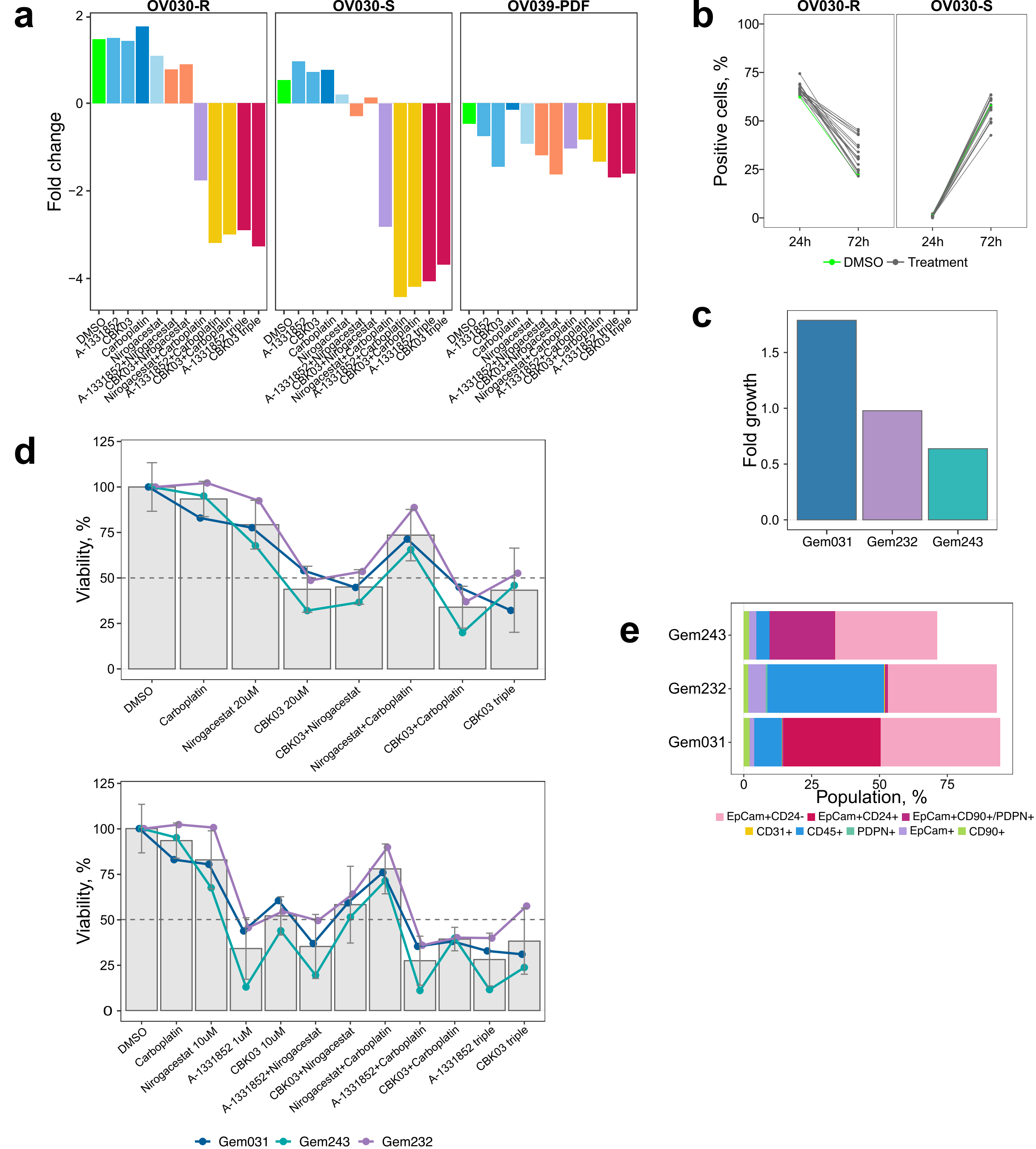

Supplementary Figure 8. a – Overview of the response of OV030-R, OV030-S, and OV039-PDF to monotherapy and combination treatments, represented as changes in spheroid volume between first and last time points of the assay. Bar plots show the average response between all tested concentrations per individual drug. **b** – Percentage of HES1 positive cells with and without treatment, 24 and 72 h in culture. **c** – *Ex vivo* culture fold growth over 7 days from the start of the imaging assay. **d** – *Ex vivo* spheroid viability in response to CBK03 based treatments at highest concentration (left hand-side) and lower tested drug concentrations (right hand-side). Error bars represent mean ± SD (n=3). **e** – Distribution of flow cytometry-based characterization of cell population in ex vivo cultures assessed on cell plating day.

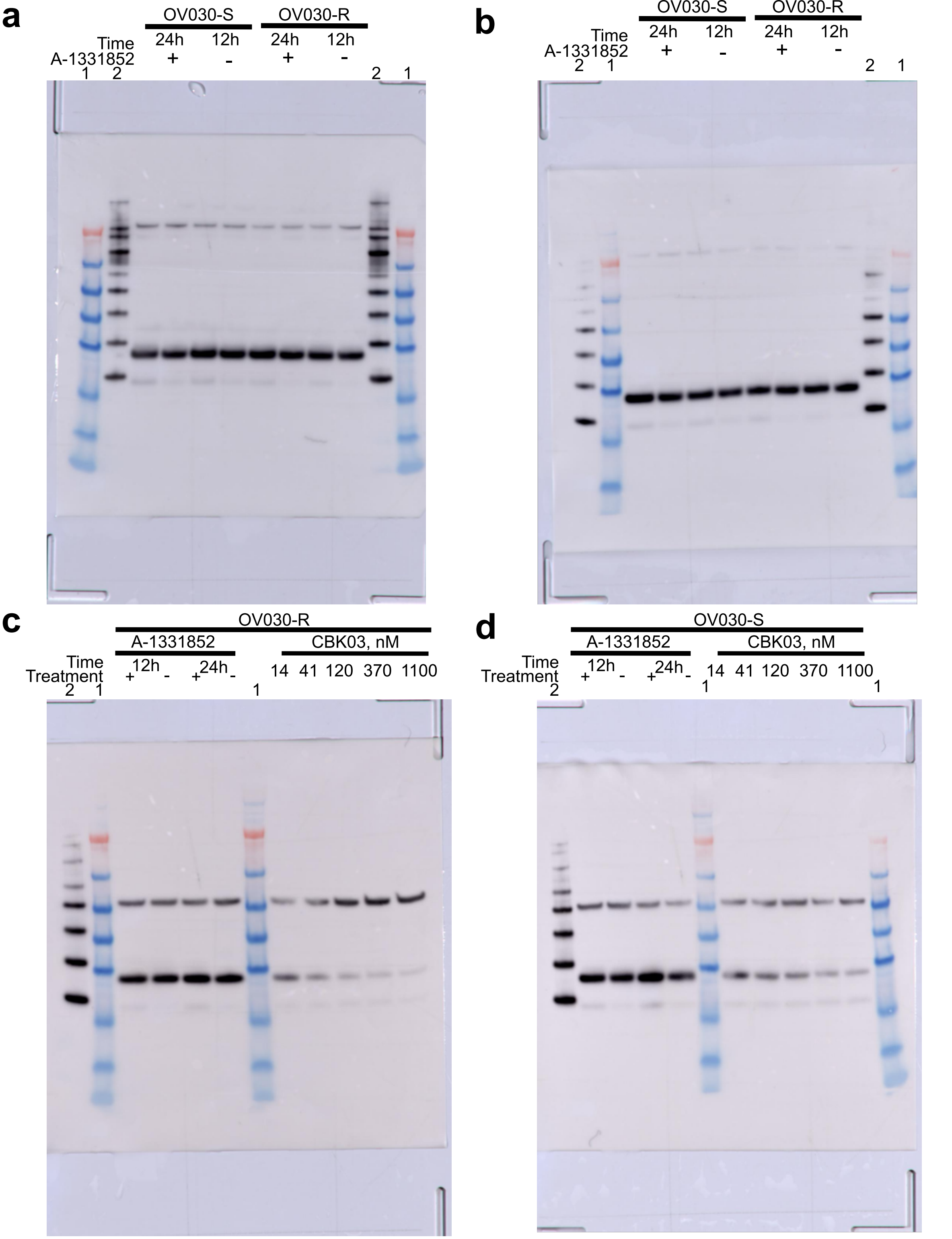

Supplementary Figure 9. Images of full Western blot gels. **a, b** – show OV030 cells treatment with A-1331852, **c, d** – gels contain samples treated with A-1331852 and CBK03. Vinculin was used as loading control for gels a and b, while b-tubulin was used for remaining gels. The images are presented as original, uncropped blots without any image adjustments to brightness, contract, or color. Exposure times were 0.5 s for gels in a and b, and 0.1 s for gels in c and d. Marker lanes are indicated as follows: 1 – SeeBlue, 2 – MagicMarker. Cropped regions from gel a were used in Fig. 3d; while cropped regions from gels c and d were used for Fig. 5e.

Table of content

#Table	Description
Supplemental Table 1	List of all PDMs and their corresponding names
Supplemental Table 2	Clinical information for the patients used to generate PDMs
Supplemental Table 3	Drug sensitivity scores for all PDMs and CCLs based on luminescence signal, represented as DSS
Supplemental Table 4	DSS comparison between ascites and tissue derived PDMs.
Supplemental Table 5	Gene names used to compare EMT and CSC associated protein expression and gamma-secretase complex containing proteins. Provided list contains markers detected in used proteomics dataset
Supplemental Table 6	ZIP synergy scores for all experiments
Supplemental Table 7	A133, Carboplatin and GSIs drug combination viability results, shown as %. Values represent average for three technical replicates
Supplemental Table 8	Passage number of PDMs at the time of drug testing and molecular characterization
Supplemental Table 9	List of antibodies used for IF and Western Blot assays
Supplemental Table 10	Drug sensitivity testing assay quality represented with Z'

Supplemental Table 1

Case ID	ID used in manuscript	PDC origin	PDF origin
Gem017	Gem017	TT	
Gem046	Gem0146	TT	
Gem096	Gem096	A	
Gem034	Gem034	TT	
Gem061	Gem061	A	
Gem062	Gem062	TT	
OV015	OV015	TT	
OV022	OV022	TT/A	
OV024	OV024	A	
OV026	OV026	TT	TT
OV027	OV027	TT	TT
OV030	OV030	A	TT
OV031	OV031		TT
OV032	OV032		TT
OV035	OV035		TT
OV038	OV038		TT
OV039	OV039		TT
OV042	OV042	TT/A	TT
OV044	OV044		TT
OC048	OV048		A
OV050	OV050		TT
OV051	OV051		TT

Abbreviations:

TT - tumor tissue
A - ascites
PDC - patient-derived cancer cells
PDF - patient-derived fibroblast cells

Supplemental Table 2

ID used in manuscript	Diagnosis-histology	Stage	BRCA-mutation	HRD	Ca-125	Operation	NACT-type	Postop treatment	Result	Date end of treatment	tion treatment	Relapse/progression	Date of progression	PFI (months)	BMI	Result2
Gem017	Clear cell OC	3C	No	Negative	85	Radical	Kar/Pak	Kar/Pak	**CR**	4/29/22		Yes	8/22/22	4	16.5	DOD
Gem046	HGS OC	4A	No	Inconclusive	1060	Non-radical	Kar/Pak	Kar/Pak	PR	9/20/22	Bev	Yes	12/1/22	2	24.30	DOD
Gem096	HGS OC	4B	No	Negative	1310	Non-radical	none	Kar/Pak/Bev	PR	5/25/23	Bev	Yes	12/11/23	6	30.33	DOD
Gem034	Growing teratoma syndrome		No	NI	NI	Radical	none	none	**CR**	9/8/22					19.57	
Gem061	Serous endometrial cancer	4B	Unknown	NI	6490	Optimal	none	Kar single	PR	1/30/23		Yes	9/14/23	7	32.88	DOD
Gem062	High grade immature teratoma	1A	NI	NI	40	Radical	none	BEP	**CR**	2/3/23					20.96	
OV015	HGS OC	3C	No	NI	243	Inoperable	none	Kar/Pak/Bev	PR	6/17/19	Bev	Yes	1/27/20	8	23.29	DOD
OV022	HGS OC	3C	No	NI	14500	Optimal	Kar/Pakx3	Kar/Pak/Bev	PR	12/27/19	Bev	Yes	6/8/20	5	21.60	DOD
OV024	LGS OC	4A	NI	NI	473	Optimal	Kar/Pakx3	Kar/Pak/Bev	PR	6/17/20	Bev	Yes	11/13/20	5	18.90	DOD
OV026	Serous endometrioid cancer	4B	NI	NI	2560	Radical	none	Kar/Pak	**CR**	11/26/20		Yes	4/6/21	4	20.03	DOD
OV027	HGS OC	4A	No	NI	2840	Inoperable	none	Kar/Pak/Bev	PR	11/11/20	Bev	Yes	12/28/20	1	28.16	DOD
OV030	HGS OC	3C	No	NI	2960	Inoperable	none	Kar/Pak/Bev	PR	3/25/21	Bev	Yes	8/19/21	4	36.98	DOD
OV031	HGSOC	4A	No	NI	3130	Optimal	Kar/Pakx3	Kar/Pak/Bev	PR	2/8/21	Bev	Yes	11/2/21	7	28.34	DOD
OV032	Clear cell endometrial cancer	4B	No	NI	172	Optimal	none	Kar/Pak	PR	3/29/21		Yes	7/30/21	4	30.27	DOD
OV035	LGS OC	4B	NI	NI	427	Radical	none	Kar/Pak/Bev	**CR**	3/24/21		Yes	11/30/23	32	26.57	
OV038	HGS OC	3C	No	NI	125	Radical	none	Kar/Pak	**CR**	7/23/21					26.60	
OV039	HGS OC	3C	No	NI	103	Inoperable	none	Kar/Pak	PR	7/29/21					27.24	
OV042	Recurrent mucinous OC from 2018	1A (2018)	NI	NI	356	Inoperable	none	Karbo	PD	6/4/21		Yes		0	43.88	DOD
OV044	LGS OC	3C	NI	NI	99	Radical	none	Kar/Lipdox	**CR**	11/5/21		Yes	10/20/22	18 & >12	29.37	
OV048	HGS OC	3C	Yes	NI	120	Radical	Kar/Pak + Kar	Kar single	**CR**	12/2/21	PARP	Yes	12/30/23	24	23.71	
OV050	HGS OC	3C	Yes	Positive	1580	Optimal	none	Kar/Pak/Bev	**CR**	2/22/22	Bev+PARP				24.58	
OV051	HGS OC	3C	No	Negative	107	Radical	Kar + Kar/Pak	Kar/Pak	**CR**	12/29/21	PARP	Yes	4/20/23	15	21.13	DOD
Gem031	HGS OC	4B	No	Negative	4160	Non-radical	none	Kar/Pak	PR	10/7/22	Bev	Yes	7/19/23	9	25.78	
Gem243	HGS OC	3C	No	Positive	7370	Optimal	none	Kar single	PR	7/11/24						
Gem232	HGS OC	4A	No	Positive	283	Non-radical	none	Kar/Pak	PR	9/25/24	Olaparib	Yes	1/3/25	3		

Abbreviations:
OC - ovarian cancer
HGS - high grade serous
LGS - low grade serous
PDF - patient-derived fibroblast cells
NACT - neoadjuvant chemotherapy
Kar - carboplatin
Pak - paclitaxel
Bev - bevacizumab
Lipdox - carboplatin+pegylated liposomal doxorubicin
BEP - bleomycin, etoposide, cisplatin
DOD - dead of disease (OC)
NI - no information

Supplemental Table 3

DRUG_NAME	class	subclass	A2780	BM-1690	BM-1691	EFO21	Gem017-T	Gem046-T	Gem034-T1	Gem061-A	Gem062-T	KURAMOCHI	MH	NCI-ADR-RES	OAW28	ONCODG-1	OV015-T	OV022-A	OV022-T1	OV026-T	OV026-PDF	OV027-T	OV027-PDF	OV027-PDF-R	OV030-R	OV030-S	OV030-A3	OV030-A2	OV030-PDF	OV030-PDF-R	OV031-PDF	OV031-PDF-R	OV032-PDF	OV039-PDF	OV039-PDF-R	OV042-A	OV042-T	OV042-PDF	OV042-T2	OV044-PDF	OV048-PDF	OV048-PDF-R	OV048-PDF.T	OV050-PDF	OV051-PDF	OVCAR3	OVCAR4	OVCAR8	OVSAHO	OVTOKO	Gem096	TYKNU	CAOV3	OV024-	OV035-P	OV038-P	OV022-T2	COV362
1-methyl-D-tryptophan	X. Other	Other	0	0	3.1	0	0	0	0	0	0	0	0.3	0	0	0		0	0	0	0	0	0	0	0	0	0	0.1	0	0	0	0	0	0	0	0	0	0	0	0	0	0	0	0.3	0	0	0	0	0	0	4.4	0	0.05	0	0.7	0.0	0	0
4-hydroxytamoxifen	F. Hormone therapy	Antiestrogen	1.9	5.3	4.7	0.6		0	0	2.2	0.6	2.1	6.6	4.1	3.3	4.9	3.3	4.3	5.1	5	2.8	3.3	1.1	1.6	6.3	4.9	4.1	4	1.7	4.9	0.6	5.5	2.1	3.6	5.1	0.7	0	4.4	7.4	1.9	1.1	3.5	2.4	1.5	2.2	5.5	3.3	4.1	4.2	7	6.3	3.6	2.45	3.7	2.2	3.2	5	1
8-amino-adenosine	A. Conventional chemotherapy	Antimetabolite	23.9	13	11.6	21.9		20.3	13.8	19.1	20.4	20.1	24.1	23.6	22.6	22.8		18.9	16.6	19.4	21.2	20.6	15.2	13.5	21.3	18.5	18.5	19.4	19.9	18.4	17.4	17.5	20.9	16.9	18.2	16.1	17.5	18.1	21.4	13.7	17.4	15.1	10.6	15	14.6	21.2	19.6	22.2	20.5	26.2	12.8	24.1	23.2	23.95	20.4	16.9	16.1	21.6
8-chloro-adenosine	A. Conventional chemotherapy	Antimetabolite	21.3	10.4	8.9	8.9		4.2	2	6.2	4.4	10	20.8	22	11.2	14.1		6.4	5.7	8.9	11.3	10.1	1.5	7.3	8.3	7.9	6.1	8.4	7.5	2.1	4.3	1.8	8.8	9.4	4.8	4	3.5	6.2	10.9	3.8	6.5	0.1	3.6	4.3	4	12.5	11.5	18.3	5.1	18.1	3.9	20.9	14	13.45	8.1	6.6	7	15.3
A-1155463	D. Apoptotic modulator	BH3 mimetic	7	0	10.7	4.8		2.1	6.8	0.9	5.1	18.6	2.7	5.7	21.6	6.9		24.2	15.3	7.7	3.1	19	0	0	13.1	19.6	14.7	16.7	0	0	4.7	0.4	3.5	0	0	9.1	10.7	0	29.7	0	19.8	0	1	0.4	4.9	6.1	5.5	4.3	4.1	1.8		8.1	16.55	20.1	1.7	1.7	27.7	1.1
A-1210477	D. Apoptotic modulator	BH3 mimetic	8.7	6.7	0	5.6		7.2	6.3	7.9	5.5	7.8	12.3	2.4	5.3	8.2		5.4	5.6	6.9	1.2	8.9	4.6	4.2	6.2	7.1	7.4	6.5	2.9	5.6	4	6.7	2.5	4.8	6.9	5.6	5.6	5.5	6.4	4.2	1.5	7.6	3.4	2.3	2.8	5.4	3.4	6.3	4.8	7.3		6.9	5.25	9.95	2.35	3.5	5.7	3.1
A-1331852	D. Apoptotic modulator	BH3 mimetic	0	3	0	0.6		1	1	0	1.2	9.4	0	3	1.8	3.3		12	7.7	4.6	8.9	9.5	0.4	1.7	4.4	15.3	7.4	14.9	0	0	0	0	2.1	0.4	0	5.2	6.6	0	15.8	0	4.8	0	0.2	0	1.2	3.1	4.6	1.1	0	0		2.4	6.3	14.15	2.7	0.9	15.7	0.3
A-366	C. Differentiating/epigenetic modifier	Other differentiating/epigenetic modifier	0	0.9	4.4	0.5		0	0	0	0	0.3	2.6	0.3	2.1	1.4		0	0.2	0	0.1	0	0	0.4	0	1.2	2.2	0	1.4	0	0.4	0	1.3	0	0.3	0	0	0	0.1	1	0.8	0	0.5	0	0.5	0.3	0	1.4	0	14.2	0	1.3	2.35	1.65	0.6	0.8	0	1.8
A-419259	B. Kinase inhibitor	Multi kinase inhibitor	26.2	4.5	5.2	14.5		9.1	7.6	14.5	9.9	9	9.8	9.3	22.3	22.1		11.8	9.1	7.9	13.6	6.4	13.9	8.2	8.5	7.4	3.8	8.3	16.7	9.4	17.2	10.7	15	10.5	10.8	1.6	2.8	18.7	11.9	15.8	15.2	9.3	13.8	11.4	11.9	15.5	11	9.9	15.6	9.5	5.6	13.3	16.95	8.25	18.15	15.3	8.9	9
ABC294640	B. Kinase inhibitor	Other kinase inhibitor	5.8	5.1	7.9	1.2		1.8	1.9	3.3	2.5	3	7.2	7.3	5.1	5.2	3.6	2.1	4.2	3.8	1.9	4.2	3.5	1.5	6.5	5.4	4.9	5.4	3.6	4.4	4.5	2.9	1.4	2.4	4.3	1.2	1.2	5.5	5.6	2.4	2	4.2	3.7	2.4	1.5	5.1	2.9	3.9	2.7	8.3	2.4	5.1	2.95	7	2.9	3.0	6.4	1
Abemaciclib	B. Kinase inhibitor	CDK inhibitor	3.3	4.9	7.3	1.7	0	0	0	0	1.4	4.7	16.9	0	7.8	5.7		2.8	2.7	4.8	1.7	1.7	0.2	4.7	0.6	0	2.4	0	4	0	3.8	1.4	4.9	1.4	2.5	0.8	1	1.3	4.4	1.5	2.7	1.1	2.2	1.5	2.3	2.5	0	0.6	5.7	19.6	0.2	10	3.15	0.6	2.6	0.2	0	5.8
Abexinostat	C. Differentiating/epigenetic modifier	HDAC inhibitor	13.1	16.4	16.4	6.8		6	5.3	7.6	10.5	13.2	18.1	8.2	18	15.1		11	8.6	8.1	6.7	7.7	8.8	3.2	13.2	17.1	12.3	14.5	10	6.6	9.3	4	8.6	8.4	10.9	6.2	5.9	10.2	10	5.2	7.6	6.3	8.9	5.9	7.6	12.9	9.7	18.3	7.8	16.9	9.3	16.8	13.65	9.3	9.7	6.1	11.4	3.3
Abiraterone	F. Hormone therapy	Other hormone therapy	0	0	0.4	0	0.5	0	0	0	0	0	0.2	0	0	0	2.2	0.7	0	2	0	0	0	1.7	1.1	0	0	0	0	0	0	0	0.7	0.5	0	0	0	0	2.1	0	0	0	0	0	0	0	0	0	0	1.6	0	0	0	0	0.05	0.0	0	2.2
ABT-751	A. Conventional chemotherapy	Mitotic inhibitor	14.6	5.3	2.6	2.3		0	2	3.4	2.9	4.3	15.2	12.6	12.3	13.5		8.6	4.9	3.5	11.5	13.6	10.4	8.3	7.2	2	0	4.2	10.2	7.5	11.9	12	10.6	8.8	11.2	0.2	0.7	10.9	5.5	9.7	12.8	8.2	12.5	13.2	12.8	7.4	7.1	11.9	3	14.5	7.2	11.7	10.35	9.5	13.15	14.3	2.6	8.1
Acalabrutinib	B. Kinase inhibitor	BTK inhibitor	0	0	0.1	0		0	0	0	0	0	0	0	0	0.1		0	0	0	0	0	0	10.4	0	2.2	0	0	0	0	0	0	1.6	0	0	0	0	0	0	0	0	0	0	0.8	0	0	0	0	0	0	0	0	0	0.1	0	0.0	0	0
Acitretin	C. Differentiating/epigenetic modifier	Retinoic acid receptor ligand	7.2	0	0	0	0	0	0	0.7	0	0	0.7	0	0	0		0	0	1.6	0	0.4	3.7	0	0	0	0	0	5.5	1	2	2.7	0.3	0.7	0	0	0	0	0	5.8	2.9	0.3	0	0	0.7	20.5	0.8	0	0	1.9	0	0	0	0.35	1.2	1.7	0	0
Afatinib	B. Kinase inhibitor	EGFR or ERBB2 inhibitor	17.6	0.1	3.1	1.2	7.6	1.6	8.8	9.5	7.8	0.2	0.2	0.4	6.8	13.9	8	10.1	6.9	12.6	1.1	3.5	0	1.5	7.2	7	3	8	0	7.2	0	5.2	0	0	8.6	0	0	0	3.1	0	0	0	0	0	0	6.6	0.5	2	1.5	0.4	6	0.6	3.7	2.3	0	0.0	6.6	0
Afuresertib	B. Kinase inhibitor	AKT inhibitor	8	1	0.1	3.2		1.6	4.6	11.7	5.7	0.9	8	1.1	1.7	5.8		3.5	2.9	3.7	1.8	1.7	0	0.4	6.6	1.5	4.9	3.8	0.7	2.2	3.7	2.6	2.5	1.1	0.8	0	0	1.9	5.5	0	1.4	0.6	0	0	0.7	4.2	0.3	2	2.9	8.9	2.1	2.4	0.35	6.45	2.4	0.2	2.9	0
Aldoxorubicin	A. Conventional chemotherapy	Topoisomerase inhibitor	10	8.5	8.5	1.9		2.2	5.7	4.6	5.9	6.7	18.9	0.1	5	5.1		6.2	5.8	3.9	6	5.9	8.1	5.1	7.4	4	5	6.1	10.2	7.7	9.2	8.5	8.5	6.7	7.1	3.1	3.6	0	4.1	7.3	6.5	5.7	7.2	3.5	6.6	6.4	5.6	8.9	3.8	16.5	5.9	11.2	8.05	7.3	7.3	4.8	6.8	7.6
Alectinib	B. Kinase inhibitor	ALK inhibitor	0	0	0	0.7	0.3	0	0	0	0	0	0.9	2	1.6	0.3		0.6	0.3	0.1	0	0	0	0.8	0.7	2.4	1	0.8	0	0	0	0.3	1.2	0.1	0.2	0	0	0	2.3	0	0	0.2	0.1	0	0	1.6	0	0.3	0	2.3	0	1.4	0.1	0	0	0.0	0.3	0
Alisertib	B. Kinase inhibitor	Aurora kinase inhibitors	6.2	5.9	0	3.7	0.6	3.8	0	0.7	3.1	4.2	18.7	8.2	3.1	9.4		3.4	3.1	2.6	2.6	10.4	0.5	3.5	3.7	4.7	4.5	0.3	2.6	2.5	3.8	5.7	3.4	4.6	1.6	0.3	0.4	7.2	1.1	1.5	1.7	2.6	0.5	0.4	1.3	14.4	4.4	5.8	4.5	21.7	0.8	8.5	15.35	6.2	3.2	2.5	0.5	2.4
Allopurinol	A. Conventional chemotherapy	Other antineoplastic	0	0	0	0	0	0	0	0	0	0	0.3	0	0	0		0	0	0	1.4	0	0	0	0	0	0	0	0	0	0	0	0	0	0	0	0	0	0.1	0	0	0	0	0	0	36.5	0	0.9	0.2	1.7	0	0	0.85	0	0.1	0.0	0	0
Alpelisib	B. Kinase inhibitor	PI3K or MTOR inhibitor	9.9	0	0	3.2		1.5	3.3	3.6	3.8	0.5	9.2	0	4.3	5.2		5.1	4.9	1.1	0.2	1.1	1	1.8	1.5	0.1	1.7	0.7	2.9	1.6	3.1	1.8	3.3	1.6	0.8	1.5	2	3.2	3.5	1.8	3.6	0.6	2.2	2.8	3.6	1.7	0.2	1.1	3.6	11.3	0.8	2	2.5	2.15	2.95	3.5	5	0.2
Altiratinib	B. Kinase inhibitor	Multi kinase inhibitor	4.8	3.7	4.9	1.6		4.8	2.5	2.4	5.4	1.7	10.1	6.6	0	2.7		5.8	5.7	6.5	7.4	0	3.8	4	5.2	4.3	3.3	3.7	6.9	1.5	7.7	4.7	6.8	5.7	2.3	1.4	2.5	7.2	6.2	2.7	3.9	2.8	4.9	4.9	4.8	5.5	1.9	5.6	3.2	10	2.1	6.4	4.45	7.05	6.3	6.2	5	6.8
Alvocidib	B. Kinase inhibitor	CDK inhibitor	19.5	19.5	19.2	14		11.8	16.8	13.2	19.6	15	22.4	17.9	17.8	18.5	15.5	14.4	9.6	16.1	9.6	18.2	6.4	5.7	12.6	11.8	14.3	8.5	12.7	11.7	19.1	18.8	15.2	11.1	11.3	11.1	11.3	18.1	17.4	10.6	14.4	10.4	13.4	13	12.8	19	11.9	19.9	10.9	20.1	2.7	21	15.75	18.65	12.4	11.8	17.8	18.5
Amcasertib	B. Kinase inhibitor	Other kinase inhibitor	3	3.2	9	6		0	0.9	0	0	5.6	4.1	7.7	1.8	8.3		0.9	0.7	6	0	3.5	0	6	0.1	2.8	0.2	5.7	0	0	0	0	0	2.4	0	0	0	0	0	0	0	0	0	0	0	4.8	0	8	5.9	7.5	0	4.5	0	7.15	3.45	2.2	0.8	0
AMG-232	D. Apoptotic modulator	p53 pathway modulator	0	8.7	10.6	1		0	4.5	0	0	0	17.3	0	0.1	0.6		0	0	0.1	6.4	0.1	2.2	8.5	0	0	0.9	0	2.7	2	7.8	8.6	5.1	5.4	7.4	0	0.1	6.3	0	5.9	5.2	4.3	1.9	0.6	1.2	0	0.3	0.6	0	16.4	0.2	0.3	0	2.05	4.75	4.4	0	0
AMG-337	B. Kinase inhibitor	MET inhibitor	0	0	0.2	0		0	0	0	0	0	0	0	0	0		0	0	0	0	0	0.1	0	0	0	0	0	0	0	0.2	0	0	0	0	0	0	0	0	0	0	0	0	1	0	0	0	0	0	0	0	0	0	0	0.1	0.0	0	0
AMG-925	B. Kinase inhibitor	Multi kinase inhibitor	0	0	0	0		0	0	0	0.8	0.4	5.3	0.5	0	0		0	0	0	0	0	0	0	0	0	0	0	0	0	0	0	0	0	0	0	0	0	0	0	0	0	0	0	0	0	0	0	1.3	5.8	0	3.6	0	0	0	0.0	0	0.1
AMG319	B. Kinase inhibitor	PI3K or MTOR inhibitor	0	0	0	0.8		0	0	0	0	0.8	3.1	0	0	2.6		0.1	0	0	0	0	0	0.6	0	1.6	0	0	0	0	0.1	0	0.7	0	0	0	0	0	0	0	0	0	0	0	0.1	0.3	0	1	0.3	2.7	0	0.1	0.8	0	0.2	0.1	0	0
Aminoglutethimide	F. Hormone therapy	Aromatase inhibitor	0	0	0	0	0	0	0	0	0	0	0.6	0	0	0	0	0	0	0	0	0	0	0	0	0	0	0	0	0	0	0	0	0	0	0	0	0	2.3	0	0	0.1	0	0	0	0	0.1	0	0	0	0	0	0	0	0	0.0	0	0
Amsacrine	A. Conventional chemotherapy	Topoisomerase inhibitor	10.5	11.2	11.1	2.8	1.2	0.1	1.2	1.1	2.6	3.7	22.4	1.5	4.6	6		7.2	5.4	3.6	2.1	4.2	0.1	2.4	1.2	0.9	2.8	3.3	6.8	0.9	4.5	1.8	1.3	2	3.2	2.1	4	6	4.4	2.1	1.8	2	0.7	0	0.7	15.8	0	9	0.2	20.9	0	15.4	10.4	6.15	1.5	0.9	3.6	5.7
Amuvatinib	B. Kinase inhibitor	Multi kinase inhibitor	0.8	2.2	1.7	0.1		2.4	0	0.3	0.3	0.3	9.1	6.8	0.2	2.9		0.4	0.3	0	0	0.2	0	2	1.1	5.8	2.4	0	0	0	0	2.3	0	0	0.9	1.2	4.2	0	0.7	0	0	1.1	0	0	0	0.5	1	4.1	1	7.2	5	0.6	0.05	1.2	0	0.0	1.5	0.7
Anagrelide	X. Other	Other	0	0	0	0	0	2.6	0	0	0	0	0	0	0.9	2.2	11.8	7	2.4	0	7.6	0	0	1.9	3.6	2.4	9.6	6.9	0	10.6	0	12.1	5.7	2.2	11.8	0	0	0	0	0	0	7	0	0	0	0	0	0	0	0	0	0	0	6.25	2.05	0.8	0.5	0.2
Anastrozole	F. Hormone therapy	Aromatase inhibitor	0	0	0	0	0	0	0	0	0	0	0	0	0	0	0.3	0	0	0	0	0	0	0	0	0	0	0	0	0	0	0	0	0	0	0	0	0	0	0	0	0	0	0	0	0	0	0	0	0	0	0	0	0	0	0.0	0	0
Apalutamide	F. Hormone therapy	Antiandrogen	0	0	0	0		0	0	0	0	0	0	0.8	0	0		0	0	0	0	0	0	0.3	0	0.2	0	0	0	0	0.3	0	0	0	0	0	0	0	0	0	4.6	0	0	0	0	0	0	0	0	0	0	0	0	0	0.45	0.1	0.1	0
Apatinib	B. Kinase inhibitor	VEGFR inhibitor	0	0	0	0	0	0	0	0	0	0	8.8	0	0	0		0.3	0.4	0	0	0.7	0	0	0.3	0	0.7	0	0	0	0	0.1	0	0	0	0	0	0	0.8	0	0	0	0	0	0	0	0	0	0	0.4	0	0	0	0	0	0.0	0	0.6
APR-246	D. Apoptotic modulator	p53 pathway modulator	0.6	0.8	2.8	0		0	0	0	0	0	1.2	0	0	0		0.7	0	0	0	0	0	0	0	0	0	0.3	0	0	0	0	0	0	0	0	0	0	0	0	0	0	0	0	0	0	0	0	0	1.3		0	0.35	0.2	0	0.0	0	0
AR-42	C. Differentiating/epigenetic modifier	HDAC inhibitor	11.1	12	11.6	4.5		4.9	5.6	6.8	9.7	9.8	13.7	16.1	11.8	14.4		10.5	7.9	7.9	1.8	8.2	6.5	5.1	9.6	13.1	9	10.9	7.5	5.7	7.1	5.1	5.9	4.5	7.2	5.1	4.8	8.2	8.5	3.6	5.2	6.3	5.6	5.1	5.1	10.8	8.2	14.4	6.3	13.1	4.8	11.5	9.75	8.4	6.1	5.6	9.7	1.2
Arsenic(III) oxide	C. Differentiating/epigenetic modifier	Other differentiating/epigenetic modifier	0	0	0.1	0	0	0	0	0	0	0	0	0	0	0		0	0	0	0	0	0	0	0	0	0	0	0	0	0	0	0	0	0	0	0	0	0	0	0	0	0	0	0	0	0	0	0	0	0	0	0	0.1	0	0.0	0	0
ARV-825	C. Differentiating/epigenetic modifier	BET inhibitor	2.6	23.2	24.4	8.8		3.2	12.4	9.3	15.3	7.7	24.9	0	11.7	13.3		13.9	10.5	10.4	7.5	8.1	13.2	5.8	3.5	5.9	6.1	4.3	15.3	17.8	15.5	15.3	14.1	6.6	14.9	8.1	6.9	13.8	7.9	13.7	15.4	13.1	12.8	11	12.1	7.2	10.8	17.9	6.1	22.8	7.7	15.5	8.45	10.35	11.9	7.7	11.4	0
Asciminib	B. Kinase inhibitor	BCR-ABL inhibitor	0	0	0	0		0.9	0.3	0	0	0.2	2.8	0	0	0		1.2	1.2	0	0	0.1	0	0	0.2	0.6	0	0	0	0	0	0	0	0	0	0	0	0	3.1	0	0	0	0	0	0.2	0.2	0	0.6	1.1	2.7	2.2	1.4	0	0.05	0	0.1	0.6	0.4
ASP3026	B. Kinase inhibitor	ALK inhibitor	8	2.8	1.2	9.2		6.2	2.6	7.6	7.2	4	6.9	5.5	8.6	9.6		6.2	5.5	3.8	8.5	2.2	3.2	1	1.8	2.3	3.2	3.1	8.2	5.3	10.5	7.5	8.1	9.1	5.5	3.8	2.5	11.2	7.2	6.8	8.8	2.6	7	7.9	8.6	5.8	3.6	6.5	6.5	7.2	1.1	5.8	8.25	4.05	8.45	9.2	5.1	6.6
AT 101	D. Apoptotic modulator	BH3 mimetic	16.8	16.5	16.6	13.9		13.1	15.4	17.4	15	13.7	18.8	18.3	12.2	16.5		14.9	15.4	13.1	8.2	17.5	7.2	10.2	15.2	14.3	14.4	13.4	10.1	12.8	10.2	14.9	9.7	9.6	12.7	13.4	13.3	11.4	12	11.4	9.2	15.2	10.6	8.2	9.7	12.8	12.6	17.1	11.6	20.1	11.4	14.8	12.45	15.75	9.8	9.9	14	11.9
AT-406	D. Apoptotic modulator	Other apoptotic modulator	0	2.3	3.6	5.1		0	0	0	0	0	0	0	0.2	0		11.7	7.8	1.8	0	8.6	0	0	0	2.6	0	5.4	0	0	0	0	0	0	0	0.2	0	0	8	0	0.2	0	0	0	0	4	11.2	7.5	0	0		1.6	9.6	2.85	0	0.0	7.6	0
AT13148	B. Kinase inhibitor	Other kinase inhibitor	7.7	1.8	0.9	0.2		2.1	4.7	3.2	2.9	0	4.1	0	0.1	0		1.5	0.7	3	0	0	0	0.1	2.1	4.7	2.2	1.3	3.5	4	2.7	4.3	1.1	0.7	3.7	0	0	2.8	0	1.8	2.7	2.7	0.5	0.2	1.2	0	0	0.1	0	2.1	2.3	0.9	1.55	2.6	0.75	0.3	0.5	0.3
AT7519	B. Kinase inhibitor	CDK inhibitor	14.2	19.5	19.9	8.9		8	14.4	10.6	15.5	10	19.3	1.1	16.1	15.8		8.9	8.3	11.1	10.8	15.6	7.8	7.9	9.6	12.5	10.3	11.3	15.9	9.4	19.8	13.3	14.4	13.5	10.7	5.9	6.2	0	15.3	12.2	11.5	8.5	13.1	9.7	11.9	15.4	12.5	16.1	9.1	18.8	1.3	16.3	14.35	16.75	13.05	8.0	9.9	16.3
AT9283	B. Kinase inhibitor	Aurora kinase inhibitors	7.2	5.7	3.4	0		1.8	0	0	0	2.1	11	0	0.1	6.5		3.1	1.1	1.4	3.6	6.1	5.4	0.1	0.4	0	0	0.5	4.4	0.4	3.2	4.3	5.8	6.3	1.8	0.2	0	8.2	1.6	5.1	3.8	2.2	3.6	3.5	2	9.4	3.6	1.4	0.2	4.5	0	7.7	13.65	2.85	5.9	2.7	1.8	3.7
Atorvastatin	G. Metabolic modifier	HMGCR inhibitor	0.9	4.6	1.6	0	4.6	4.4	0.5	3.9	5.5	4.4	7.7	1.4	0	0		1.6	1	1.8	0	1	1.8	3	0	2.8	1.2	0.6	1.9	6	3	5.9	0.9	1.3	3.1	0.5	0.8	2.2	3.5	1	1.9	4.9	1.3	0.6	2.2	0	0	3.7	1.1	5	0.1	0	0.4	3.2	1	0.5	1.7	0
Auranofin	A. Conventional chemotherapy	Other antineoplastic	4.9	7.7	12.4	0	3.3	6.2	5.5	4	3.7	9.6	15.1	8.7	5.8	8.9		5.8	5	9.2	0	7.2	0	1.9	8.1	4.7	6.6	3.7	2.6	4.8	0	3	0.7	0	2.9	0	0	0	2.2	2.3	2.3	4.4	1.5	0	0	15.5	3.5	4.7	2.8	19.5	5.7	5.9	9.15	8.9	0.2	0.0	4.7	5.9
AVN944	G. Metabolic modifier	Other metabolic modifier	20.4	11	4.5	10.6		2.7	1.6	11.4	5.3	10.7	26.1	5.2	11.3	19.6		11.7	10	5.6	1.3	8.8	8.3	3.4	11.5	13.1	10.2	14.8	10.8	5.5	10.7	11.8	10.3	7.1	9.1	5.1	4.5	2.2	5.6	7.7	3.9	7.9	12	5.7	7.6	17	9.7	12.5	9.6	25.3		11.4	10.4	7.4	9.5	0.8	14.2	4.7
Axitinib	B. Kinase inhibitor	VEGFR inhibitor	1.1	0.6	2.6	0.7	0	0	0.2	0	1.8	0	11.5	2.4	0	1.3	0	0	0	0	5.7	0.1	7.9	2.8	0	0	1.3	0.1	6.6	0.9	10.2	1.4	6.7	7.8	1	0	0	2.5	0.8	7.6	3.8	2.1	7.7	5.5	5.5	1.4	0.9	1	0	11.3	2.5	3.1	6.1	1.7	7.7	6.5	0	0
AZ 3146	B. Kinase inhibitor	Other kinase inhibitor	4	3.4	2.7	2.7	1.3	0.9	0.7	2.1	2.8	2.1	7.4	4.4	5.9	3.2		1.6	1.3	0.9	5	2.2	0.6	2.1	0.7	1.2	2.6	0.1	3.7	1.2	3	0.8	2.6	2.1	0.5	0.1	0.3	5.8	6.5	2.6	3	1.2	3.3	1.5	3	2.9	0.9	5.9	4.1	8.2	0.4	6.2	7.5	1.7	3.6	3.8	1.3	3.1
AZ191	B. Kinase inhibitor	Other kinase inhibitor	5.1	4.4	7.9	0.1		0	0.1	2.6	0.1	1.2	6.8	0	1	0.8		0.6	0.5	2.5	4.2	1.1	6.3	1.4	1.3	0.8	2.1	0.6	4.5	2.3	2.1	1.4	4.9	1.8	3.5	0.1	0.3	2.4	0	3.4	1.1	1.5	3.8	1	0.7	3.6	0	0.7	0.9	8.8	0.4	3.9	4.65	1.35	3.4	1.2	2	3.3
Azacitidine	C. Differentiating/epigenetic modifier	DNMT inhibitor	6.6	4.3	1.1	2.6	0	0.4	0	1.2	0.3	6.7	9.2	3.7	0.1	4.7		6.3	2.9	5.1	0	5.3	0	0.9	7.6	6.6	4.9	6.9	0.5	1.4	1.2	6.2	0	0	2.6	1.3	1.3	3.3	2	1.4	0.1	3.6	0.1	0	0	2.1	3.9	4.4	1.8	6.6	7.3	6.3	1	7.65	0.25	0.0	3.9	2.5
AZD-1080	B. Kinase inhibitor	Other kinase inhibitor	0	0	0	0		0	0	0	0	0	0	0	0	0		1.2	0	0	0	0.1	0	1.9	0	0	0	0	0	0	0	0	0	0	0	0	0	0	0	0	0	0	0	0	0	0	0	0	0	0	0	0	0	0	0	0.0	0	0.5
AZD-5363	B. Kinase inhibitor	AKT inhibitor	11.8	3.2	3.7	2.5		1.1	6.7	14.2	6.8	0.1	9.1	0	1.5	5.2		3.6	3.6	5.6	0.2	2.1	3.2	3.7	6.9	7.3	5.5	6.4	4.6	3.8	4.6	6.3	3	1.9	2.1	0.6	0	5.8	3.2	3.7	0.6	3	0.9	1.3	2.7	4.7	0	1.9	1.7	10.9	3.6	2.3	1.15	6.7	2.85	0.4	4.8	0.6
AZD-5438	B. Kinase inhibitor	CDK inhibitor	13.1	10.2	9.8	7.3		3.1	3.6	3.8	5.8	6.4	12.8	5.9	5.5	9.5		7.1	5.3	8.5	1.6	10.2	0.1	0.9	5.2	4.2	2.3	5.5	2.6	2.5	8	4.3	8.5	1.8	3	3.8	3.4	7.9	6.6	1.9	8.3	2.3	3.5	2.1	6.5	8.7	2.7	9.5	5.6	10.5	0	13.7	7.3	6.1	3	3.0	8	8.8
AZD-6482	B. Kinase inhibitor	PI3K or MTOR inhibitor	0.8	0.1	0	1		1.8	2.7	0.6	3.3	2	2.6	0	2.3	9.2		1.6	3.3	0.5	5.4	0	0	0	0.6	0.7	0	0	1.6	1.2	3	4.3	1.3	2	1.5	0	0	0.4	0.4	0	3	1.5	0.1	1.3	1.8	2.2	2.3	0.6	3.4	4.9	0.3	3.2	3.65	0.15	1.2	2.7	1.8	0
AZD-8186	B. Kinase inhibitor	PI3K or MTOR inhibitor	2.6	0.7	0	0.9		0	0.1	0	1.6	1.8	2.7	0	4.9	12		1.7	0.8	1.4	3.4	0.4	1.5	3.7	0.7	0	0	0.9	4.3	0.9	6	3.8	5.8	2.6	3.2	0	0	2	2.3	3.4	3.7	0.6	2.1	4.9	2.5	4.3	1.7	0.5	3.6	5.3	0	1.9	3	0.2	3.4	3.5	1.5	0
AZD0156	B. Kinase inhibitor	Other kinase inhibitor	3.2	0	0	1.4		0	0.6	0.5	0.3	3.1	5	0.5	2	6		0.7	0.6	0	0.7	0	0.7	3.4	0	1.9	0	0	2.3	0	2.4	0	2.1	1.1	0	0	0.1	0	1.7	0.4	2.5	0	0.3	0.8	1.2	2.3	0	0.5	0.6	3.2	0	2.7	2.45	0.15	1.45	0.6	2.7	1.5
AZD1152-HQPA	B. Kinase inhibitor	Aurora kinase inhibitors	1.2	2.4	0	0	0	0	0	0	0	2.6	14.5	0	2.8	3.3		1.1	0	0	0.4	5	0	0	1.2	1.3	5.4	0.8	1.2	0	0	0	1.8	0.6	0	0	0.2	0.4	0	0.7	0	0.7	0.2	0	0	9.6	4.5	1.8	3.6	15.7	0	5.8	14.1	0.8	0.6	0.1	0	0
AZD1208	B. Kinase inhibitor	Other kinase inhibitor	0	4	1.4	0		0	1.9	0	0.5	0	8.6	0	3.6	0.3		2.5	0	0	0	1.2	0.1	0	0	0	0.8	0	0.3	0.9	1.5	3.6	0	0	0.7	0	1	1.6	0.3	0.4	0	0	0	0	0	0	0	0	0	7.1	0.4	0.6	0	0.25	0.65	0.0	3	0
AZD1480	B. Kinase inhibitor	JAK inhibitor	0.2	4.2	2.1	0.7	0	0	0	0	0	0	4	0.4	1.5	2.3		0	0	0.3	6.1	0.4	2.5	4.7	0	0	0.2	0	4.3	0.1	5.1	0	4.7	7	0	0	0	7.5	0.5	4.3	3.8	0	3.5	5.1	4.1	0	0	0	0	3.5	0	1	0.35	0.2	5.1	5.1	0	0.1
AZD1775	B. Kinase inhibitor	Other kinase inhibitor	18	4	2.6	12.7		9.2	0	10.8	7.6	10	20.6	9.2	17.6	20.4		19.9	14.4	7.1	0	9.7	0.5	0	12.2	14.8	11.3	14.9	7.3	3.8	8.4	4.1	6.7	5.7	3.1	4.2	6.5	10.3	8	2.8	7.4	3.7	4.7	3	3.5	18.9	11.7	15.5	8.9	20.5	5	23.6	17.1	13.15	8.3	6.1	15.5	14.8
AZD3759	B. Kinase inhibitor	EGFR or ERBB2 inhibitor	6.1	0	0	1.4		1	1.4	3.2	4.7	0	0.2	0.7	1	5.2		2.6	3	3.5	0	0	0	0	2.6	2.6	0.6	1.1	0	2.7	0	0.1	0	0	2.6	0	0	0	0.8	0	0	0.5	0	0	0	2	0	0	0.5	0	0	0	0.7	0.75	0	0.0	3.9	0
AZD3965	G. Metabolic modifier	Other metabolic modifier	0.2	0	0	0		0	0	0	0	10.4	0	0	0	0		0	0	0	0	0	0	0	0	0	0	0	0	0	0	3.4	0	0	0	0	0	0	0	0	0	0	0	0	0	0.8	0	0	0	0		0	0.2	0	0	0.0	0	0
AZD4547	B. Kinase inhibitor	FGFR inhibitor	0	0.1	0	0		0	0	0	0	0	8.8	0	0	0		0	0	0	8.7	0	6.6	3.2	0	0	0	0	10.5	0	9.6	0	11.3	9	0	0	0	5.9	0	8.3	9.1	0	7.9	6.8	7.2	0	0	0	0	11.8	0	0	0	0	9.7	8.6	0	0.1
AZD6738	B. Kinase inhibitor	Other kinase inhibitor	10.1	5.6	5.8	5.5		3.4	3.2	8	4.2	4.7	15.4	1.8	8.5	11.8		10.6	6.6	8	4.2	5.3	3.7	4.4	9.4	8.1	6.3	11.7	8.7	3.5	7.3	3.3	5.6	4.4	4.5	3.7	5.5	7.9	6.6	6	7.2	3.7	4.4	4.2	4.2	11	7.9	8.5	5.1	13	5.5	18	11.75	8.8	6.25	4.8	9.5	6.9
AZD7545	B. Kinase inhibitor	Other kinase inhibitor	0	0	0	0		0	0	0	0	0	2.9	0.3	0	0		0	0	0	0	0	0	0	0	0	0	0	0	0	0	0.2	0	0	0	0	0.8	0	0	0	0	0	0	0	0	0	0	0.8	0	1.6	0.1	0	0	0.3	0	0.0	0	0
AZD7762	B. Kinase inhibitor	Other kinase inhibitor	6.1	4.8	5.4	3.8	0.7	3	0	4.5	0	4.2	4.5	0	8.6	7.3	1.8	6	4	6.2	3.9	1.5	0.8	0	7.5	9.3	3.9	7.2	6.9	1.9	10.5	3.1	4.7	4.7	0.1	2.1	2.8	0	4.5	5	0.8	1.1	1.4	4.3	4	8	7.6	4.4	1.3	4.1	6.1	10.2	11.95	6.65	6.35	4.1	3.3	7.9
AZD8055	B. Kinase inhibitor	PI3K or MTOR inhibitor	29	7.1	7.4	22		15.9	16	18.4	18.5	16.8	29.1	21	20.3	30	19.5	17.1	14.3	18.7	21.4	14.8	10.7	19.6	21.1	18.3	21.3	19.3	22.8	15.8	23.2	19.5	24.7	21.8	17.2	11.9	13.3	24.5	22.7	19.2	23.8	14.8	17.3	16.8	22.6	21.9	19.9	20.1	15.6	31	9.8	24.2	18.05	19.05	21.2	22.4	20.2	16.6
Bafetinib	B. Kinase inhibitor	BCR-ABL inhibitor	0	0	0	0		0	0	0	0	0	0.2	0	0	0		0	0	0	0	0	1.1	0.3	0	0	0	0	0	0	0	0	0	0	0	0	0	0	0	0	3.9	0	0	0	0	0	0	0	0	0.3	0	1.1	0	0	0.15	0.0	0	0
Baricitinib	B. Kinase inhibitor	JAK inhibitor	0.6	3.4	1.1	0		0	0	0	0	0	0	0	0	0		0	0	0	0	0	0	0	0	0	0	0	0	0	0	0	0	0	0	0	0	0	0	0	0	0	0	0	0	0	0	0	0	0	0	0	0	0	0	0.0	0	0
BAY 87-2243	C. Differentiating/epigenetic modifier	Other differentiating/epigenetic modifier	4.2	12.7	13.8	3.2		0	0.2	4	4.5	0.9	28.7	25.3	3	4.8		0	1.3	0	0	1.6	0	0	0	3.5	0.9	0.5	0	0	0	0	0	0	0	9.4	13	0	2.2	0	0	2.2	0	0	0	3.2	8.6	19.9	3.1	25.3	8.6	0	1.8	5.05	0	0.0	4.4	0
BAY-1436032	C. Differentiating/epigenetic modifier	IDH1/IDH2 inhibitor	0.3	0	0	0		0	0	0	0	0.1	2.5	0	0	0.8		0	0	0	0	0.3	0	0	0	0	0	0	0	0	0	0	0	0	0	0	0	0	0	0	0	0	0	0	0	0	0	1.2	0	0	0.3	0	0	1.15	0	0.0	0	0
BCI	X. Other	Other	20.3	19.9	23.6	9.6		15.5	11.6	14.5	10.7	18.5	22.8	17.8	19.2	22.3		17	15	16.1	9.9	14.7	6.9	14.6	15.8	14.4	14.7	13.2	16.1	16.2	15	15.5	11.7	9.5	15.9	5.9	5.2	10.4	12.2	7.2	12.9	14.6	10.4	5.9	5.6	21	12.8	16	14.1	22.2		19.8	18.8	17.2	11.6	9.5	14.4	4.9
Belinostat	C. Differentiating/epigenetic modifier	HDAC inhibitor	14.5	13	14.6	5.9	9	6.1	4.3	7.7	11.7	12.6	16	14.9	15.2	18.6		13.6	10.6	9.2	6.8	8.7	5.6	3.4	14.9	15.5	14.9	14.3	7.2	7.5	6.2	8.3	5.1	6.7	5.7	4.9	4.7	0	8.8	3.7	6.1	5.9	4.3	3.7	4	17	8	16.4	7.6	17.3	9.3	16.3	11.05	12.3	6.35	5.0	11.2	6.5
Bentamapimod	B. Kinase inhibitor	Other kinase inhibitor	2.1	0.7	0.8	0.1		0.1	0.4	0	0.1	1.1	5.6	1	4	2.6		4.6	2.1	0.4	0.7	2.3	0	0	0.7	0.1	0	0.5	0.4	0	0	0	0.3	0	0	0.2	0.5	0.2	2.7	0.5	0.6	1.2	1.2	0.1	0	1.2	0	1.7	0.2	6.2	1.3	4.4	0.1	2.55	1.55	0.3	2.9	0.4
Bexarotene	C. Differentiating/epigenetic modifier	Retinoic acid receptor ligand	10.8	0.4	2.2	0	4.8	0	0	0.6	0	0	2	0	0.5	0		0.6	0	0.1	0	0.4	4.3	0	0	0	0	0.6	4.6	0.5	5	8.3	0	0	0	0	0	0	0	4.8	2.4	0	0	0.6	0	3.3	0	0	0	2.9	7.5	0.3	0.5	0.9	0.25	2.3	0	0
BGB-283	B. Kinase inhibitor	RAF inhibitor	7	0.8	0.9	0.1		0	0.3	0.3	1.2	1.2	8.6	2.5	1.3	2.4		1.1	0.5	1.5	0.3	2	6.9	1.6	3.3	4.4	1.4	3.2	3.9	1.8	1.5	3	2.5	1.8	1.7	5.9	5.7	3	8.4	6.8	0.1	2.3	5.8	1.2	2.6	3.1	2.2	2.5	1.6	8.1	6	6.5	3.35	4	4.25	2.8	1.6	3.8
BGB324	B. Kinase inhibitor	Other kinase inhibitor	3.4	3.2	5.6	3.1		1.3	0.7	1.1	1.9	1.2	8.1	6.2	6.2	5.6		6	3	2.7	4.5	1.4	0	1.6	4.4	5.6	3.8	1.4	3.9	1.2	4.5	1.4	6.5	6.1	1.8	0	0	0	4.7	2.3	3.1	1.4	0.8	0	0.9	5.6	2.3	4.2	0.8	6.4	0	7.7	8.8	4.65	6.05	2.4	2.8	2.2
BI 2536	B. Kinase inhibitor	Other kinase inhibitor	21.3	9.7	4.6	1		2.3	2.5	1.4	4.2	2	23.3	4.3	19.3	18.1		7.6	0.3	5.4	1.3	8.7	1.2	7.5	4.6	1.7	2	0.6	4.1	3.6	3.7	3.2	3.9	0	3	0.7	1.8	8.4	1.5	0	1	5.3	3	0.5	0.7	24.5	7	20.8	3.5	21.4	0.6	15.3	11.35	13.65	4.35	1.4	1.5	6.1
Bicalutamide	F. Hormone therapy	Antiandrogen	0	0	0	0	0	0.2	0	0	0	1.3	2.5	0	0	0	0	0	0	0.5	0.1	0		0	0.7	0	3.8	0	0	0	0	0	0	0	0.3	0	0	0	2.7	0	0	0	0	0	0	3.6	1.3	1.2	0.5	2.9	0	1.7	1.8	0.1	0	0.0	0	0
BIIB021	X. Other	HSP inhibitor	23.6	16.7	12.9	6.8		7.3	13.2	9.3	12.3	1.1	25.1	17.2	8.1	12.5		8.5	7.8	7.1	8.2	9.5	9.8	10	8.6	5.9	7.8	9.8	13.5	12.8	9.9	14.8	14.2	9.6	11.7	7.4	5.1	0	10.5	13.5	10.2	14.1	11.8	9.6	6.1	16.7	10.1	20	6.9	24.3		14	14.15	13.5	13.2	12.6	6.2	6.9
Bimatoprost	E. Immunomodulatory	Other immunomodulatory	0	0	0	0	1.2	0	0	0	0	0	0	0	0	0	0	0	0	0	0	0	3.9	6.7	0	0	0	0	0	0	0	0	0	0.1	0	0	0	0	0	0	0	0	0	0	0	3.1	0	0	0	0	0	0	0	0	0	0.2	0	0
Binimetinib	B. Kinase inhibitor	MEK1/2 inhibitor	11.1	2.4	1.2	2.3		0	3.2	4.1	4.8	3.6	8.5	2.2	5.6	8.4		0	0.2	1.5	2.4	0	0.5	1.1	1.7	0	1.4	0	2	1.5	0	0	3.1	2.1	2.4	5.8	5.3	1.5	5.3	2.3	0	1.7	2.5	0.7	2.3	7.9	0	2.5	4	10.1	0	10.4	4.75	1.1	2.55	1.7	0.4	0
Birabresib	C. Differentiating/epigenetic modifier	BET inhibitor	6.1	6.2	8.3	7.8		1.4	8.3	5.3	11.6	5.4	15.2	5.2	11.2	19.2		4.1	2	8.3	2.2	10.1	8.2	8.8	5.7	3.4	2.2	1.6	10.5	5.5	9.2	16.8	4.4	2.3	9.6	5.7	5.9	9.6	8.1	1.6	2.5	9.8	3.6	0	2.4	11.5	8.7	16.8	8.9	17.1	0	14.3	12.15	16.4	7.85	6.7	5.9	1.7
Birinapant	D. Apoptotic modulator	Other apoptotic modulator	0.4	5.5	3.7	5.8		0.5	0	0	0	2.3	8.2	0	0	0		10.3	7.1	0	0	8.5	0	0	1.7	0	0.1	5.7	0	0	0	0	0	0	0	0	0	0	4.9	0	0	0	0	0	0	8.8	11.7	6.5	0.1	2.1	0	4.1	10.25	5.5	0	0.0	6.9	1.8
Bleomycin	A. Conventional chemotherapy	Other antineoplastic	6.1	3.6	2.7	0	2.7	0	0.8	0.2	2.4	0.7	17.6	8.3	0	0.2		2.7	3.2	0.7	0	1.1	1.1	0.2	0.9	0	0	0.9	4.3	1.9	5.7	5.2	1	1.2	3.8	0.5	1	4.4	1.4	4.3	0.1	1.5	1.3	1.1	1.6	0	0.3	6.9	0	20.8	1.2	4.6	1.6	3.05	1.5	2.4	0.4	0.3
BMS-754807	B. Kinase inhibitor	Other kinase inhibitor	10.4	4.9	3.5	13.2		8.1	12.8	22.5	10.1	6.2	16.4	7.6	9.4	10.1	8.6	19.8	17.1	4.9	15.3	11.1	12.1	5.2	8.3	5.3	9.3	0.1	14.1	10.9	15.1	19.6	13.9	14.4	11.8	9.7	11.4	15.6	15.4	9.6	11.6	9.6	10.1	15.9	13.5	8.1	4.1	6.4	6.9	17.1	3.5	7.8	10.15	19.25	15.85	15.5	18.9	9.1
BMS-777607	B. Kinase inhibitor	MET inhibitor	0	0	0	0		0	0	0	0	0	1.9	0	1.8	0		0	0	0	0.8	0	0.1	1	0	0	0	0	0	0	0.3	1	0	0	0	0	0	0	0	0	0.2	0	0	0.1	0.2	0	0	0	0	2.5	0	0	0.3	0	0	0.0	0	0
BMS-911543	B. Kinase inhibitor	JAK inhibitor	1	3.8	2.2	0		0	0	0	0	0	0	0	0.9	2		0.2	0	0	0	0	0	1	0	0	0	0	1.3	0.7	1.1	0.9	0	0	0	0	0	1	1.5	0.3	0.1	0	0	0	0	0	0.7	0	2.1	0	0	0	0	1.15	0.05	0.0	0	0
BMS863233	B. Kinase inhibitor	Other kinase inhibitor	5	2.8	1.2	0	0.2	0.4	0	0	0.4	0	5.4	0	0	3.8		0.9	0	0	0	1	0	1.5	0	0	0	0	0	0.3	0	1.3	0	0	1.3	1.3	0.3	0	0	0	0	0.4	0	0	0	0.5	0	0	0	6	0	5.1	0.55	0	0	0.0	0.5	0
Bortezomib	X. Other	Protease/proteasome inhibitor	25.1	29	28	24.6	29.5	21.7	11.5	23.4	25.9	25.6	31.5	19.2	28.9	27.1		28.6	25	25.6	10.5	29.8	10.3	17.4	22.8	14.2	20.1	18	22.3	17.5	24.2	17.2	22.2	27.3	17.5	16.3	22.4	5.7	30.9	22.9	19.8	24.7	23.5	17.5	23.1	28	27.6	24.4	8.2	32	9.5	32	31.2	28.95	23.25	24.1	29	19.5
Bosutinib	B. Kinase inhibitor	BCR-ABL inhibitor	15.9	7.7	8.8	6.7	8.6	3.3	3.8	9.2	6.2	8	6.9	4.4	14.7	14.7	4.4	8	7.5	3.8	9	2.7	4.6	4.5	9.1	7.8	7.8	7.7	9.6	7.5	11.3	5.2	10.4	8.5	8.9	3.2	2.9	6	9.8	9.5	8.2	4.4	7	6.2	6.3	9	7.2	8.3	7.2	4.4	4	10.2	12.1	5.7	11.25	10.7	7.8	6.9
BRD7116	X. Other	Other	2.1	1.2	0	0		0	0	0	0	2.3	3.3	0.1	3.9	5.1		0	0	0	0	0	0	0	0	0	0	0	0.6	0	0	0.4	0	0	1.7	0	0	0	0	0.1	0	0.2	0	0.3	0	3.1	9.8	0	0	1.3		4	2.75	1	0.5	0.8	0	0
Brigatinib	B. Kinase inhibitor	ALK inhibitor	6.3	0.2	0.7	4.9		3.6	3.4	4.5	4.4	3.7	5.6	0	4.2	6.1		3.7	3.2	2.8	7.8	4.2	3.5	6.6	1.7	3.5	3.6	0	6.9	3.6	7.9	3.6	9.8	8	3.1	1.9	6.1	0	6	3.4	5.5	2.6	5.1	7.6	5.7	3.5	1.8	0.6	4.1	6.2	2.4	3.5	4.75	3.3	8.35	6.7	4.2	4.7
Brivanib	B. Kinase inhibitor	Multi kinase inhibitor	0	0	0	0	0	0	0	0	0	0	0	0	0	0	0	0	0	0	0	0	0	0.8	0	0	0	0	0	0	0	0	0	0	0	0	0	0	0.4	0	0	0	0	0	0	0	0	0	0	0	0	0	0	0	0	0.0	0	0
Bryostatin 1	B. Kinase inhibitor	Other kinase inhibitor	0.3	0	3.3	0		3.6	5.7	0.2	8.1	0	0	0	3.1	0.6		1	1.1	0	0	1.2	6.4	9.1	0	6.4	0.5	0	5.1	6.7	6.4	0.9	6.5	0	7.6	0	0	0	0	7.8	6.7	6.8	3.3	4	9.9	2.5	0.3	1.1	0	1.6	0	6.8	0.95	2.35	5.95	0.5	0	0
Buparlisib	B. Kinase inhibitor	PI3K or MTOR inhibitor	15.6	3.5	1.1	9.2		5.7	6.4	7.1	7.9	5.5	12.3	9.6	9.3	13	6.2	9.4	6.9	5.4	11	6.5	7.1	9.5	6.7	6.4	6.3	4.5	10.2	6.5	10	8.5	9.4	9.2	6.4	4.3	5	10.8	6.9	7.6	12.1	5.7	6.3	9.3	9.4	8.7	4.9	8	6.9	12.6	1.8	9.6	8	8.55	10.1	10.8	8.4	5
BX-912	B. Kinase inhibitor	PI3K or MTOR inhibitor	1.8	7.2	4.4	6		3.2	3.1	0.9	3.3	3.4	11.4	0.3	3.9	3.5		2.8	2.1	1.5	5.7	2.6	6.8	2.7	2.2	2	1.2	2.2	7.6	3.5	8.3	7.4	7.6	9	4.1	1.4	2.2	10.6	3.8	8.4	4.1	3.7	6.8	6.9	7.1	3.9	3.6	3.2	1.7	11.9	0.3	6	9.15	3.85	7.55	4.8	2.3	2.9
C646	C. Differentiating/epigenetic modifier	Other differentiating/epigenetic modifier	3.7	4.9	0	0		1.2	3.2	2.5	2.6	1.6	6.8	0	0	0		0	0.5	2.4	0.4	0	0	0	0	0	0	0	0.2	0	0.4	2.5	0.1	0	0	2.8	2.7	0	2.4	0.2	0	0	0	0.5	0	1.1	0	0.5	1	4.2	0	1.5	0.55	0	0.45	0.0	0	0
Cabazitaxel	A. Conventional chemotherapy	Mitotic inhibitor	31.8	13	2.7	6.2	0	5.2	3.2	4.1	8	3	31.9	12	25.5	21.4	9.7	17.3	9.3	9.9	17.4	23.5	14.5	9.6	8.1	5.1	7.5	4.2	16.4	7.9	18.3	14.3	19.7	18	9.3	0	0	2.9	7.8	13.9	25.6	13.7	15.5	23	25	34.2	17.2	24.6	0	31.5	0	25.6	24.7	20.3	24.4	26.9	4.7	14.1
Cabozantinib	B. Kinase inhibitor	Multi kinase inhibitor	0	0.4	0	0.9	4.2	1.1	0.8	0	5	0	7.4	0	3.7	0	0	0.9	0	3.1	7.2	0	0	5.4	0.2	0	4.7	2.3	2.1	0	0	1.2	1.7	2.6	0	0	0	0	0	2.2	1	2.1	1.4	1.8	6.4	0	0	0	2.3	9.6	1.7	0	3.75	1.05	1.9	1.0	0	0.8
Canertinib	B. Kinase inhibitor	EGFR or ERBB2 inhibitor	23.8	7.5	8.3	4.2	7.5	6.3	6.9	15.4	10.6	6.1	9.7	9.6	12.2	23.6	12.7	12.9	13.7	21.3	8.3	8.4	5.3	5.9	14.9	11.7	10.3	14	7.2	10.7	8.8	8.3	4.9	7.1	10	2.5	4.2	0	9.1	6.8	7.9	9.2	5.8	4.4	3.2	13.3	7.7	9.1	7.7	10.1	2.5	8.6	11.05	11.95	8.5	5.4	11.6	7
Capecitabine	A. Conventional chemotherapy	Antimetabolite	0	0	0.3	0		0	0	0	0	0	0	0	0	0		0.6	0	0	0	0	0	0	0	0	0	0.2	0	0	0	0	0	0	0	0	0	0	0	0	0	0	0	0	0	0	0	0	0	0	0	0.3	0.3	0	0	0.0	0.1	0
Capmatinib	B. Kinase inhibitor	MET inhibitor	0.1	0	0	0		0	0	0	0	0.1	0	0	0	0.2	0.2	0	1.4	0	0	0	0	0	0	0	0	0	0	0	0	0	0	0	0	0	0	0	0	0	0	0	0	0	0	0	0	0	0.3	0.9	0	0	0.8	0	0	0.0	0	0
Carboplatin	A. Conventional chemotherapy	Other antineoplastic	6.2	4.4	3.1	0	0	2.9	1.3	0	0.7	5.2	10.4	4.5	0.9	4.7	6.8	6	3.1	5.2	0.3	4.2	0.6	0	3.6	3.3	2.2	6.7	1.1	3.2	3	4.3	2.6	1.1	3.3	0.7	1.3	5.6	3	2	0.8	3.2	1	0.8	0.7	4.6	5.9	2.7	0	9.9	5.2	6.7	4.35	6.6	1.4	0.4	2.9	4.4
Carfilzomib	X. Other	Protease/proteasome inhibitor	26.7	29.1	29.9	20.8	15.5	14.4	12.2	15.8	13.5	25.7	34.8	7.8	29.2	27.6		26.8	21.9	25.4	19	24.3	6.3	14.4	23.3	17.6	21.2	20.1	23.4	19.1	23.7	17.2	20.8	21.9	23.7	8.5	12.4	6.5	23.4	20.4	16.8	21.5	16.1	0.8	14.5	27.9	24	22	12.6	31.3	11.1	30	30.6	25.8	21.85	21.9	27.2	21.3
CC-115	B. Kinase inhibitor	PI3K or MTOR inhibitor	14.8	7.7	8.2	9.6		9.3	11.8	11.4	13.4	11.8	16.8	9	8	16.7		8.9	7.6	6.9	9.3	8.1	5.4	10.5	9.3	8.6	9.3	6.2	12.9	6.9	11.4	8.2	9.3	8	7	7.1	8.1	8.8	14.1	11.8	12.4	6.8	9.2	0.8	11.4	12.4	8.1	10.2	9.4	19.6	5.7	11	9.45	10.95	8.5	7.1	11.4	8.6
CC-223	B. Kinase inhibitor	PI3K or MTOR inhibitor	8.3	6.7	4.6	6		2.7	5.7	5.8	6.1	4.9	7.1	5	5.9	9.1		1.8	1.8	4.3	4.8	2.8	3.4	6.2	5.8	3.8	5.3	3.2	6.9	3.3	7.5	4.8	4.3	3.7	4.7	2.7	1.7	3.4	6.5	3.4	4.6	2.5	3.6	0.8	4.1	5.8	4.7	4.8	3.9	10.4	2.3	6.4	3.85	5.55	4.05	3.2	3.7	2.4
CC122	E. Immunomodulatory	Other immunomodulatory	0	0	0	0		0	0	0	0	0	0	0	0	0		0	0	0	0	0	2.8	0	0.7	0	0	0.1	0	0	0	0	0.9	0	0	0	0	0	0	1.4	0	0	0	0.8	0.1	0	0	0	0	1.7	4.8	0	0	0.1	0.05	0.0	0	0
CCT196969	B. Kinase inhibitor	RAF inhibitor	9.2	10.2	5.7	6.3		1.9	0	0.8	0.4	7.9	14	4.4	1.3	9		4.6	5.3	2	0.7	4.5	0.2	3.6	0.1	0	0	1.3	7.5	0	7.5	0.4	2.7	3.6	0	2.8	2.9	1.5	4.6	6	3.8	0.1	5.6	0.8	4.8	2.6	0	8.4	7.6	13.6	0	2.2	3.85	6.5	3.45	2.3	4	7.6
Cediranib	B. Kinase inhibitor	Multi kinase inhibitor	0	0	0	0	0	0	0	0	0	0	0.9	0	0.1	0.1		0	0	0	0.5	0	0.5	0	0	0	0	0	3.9	0	3.9	7.2	1.7	2.7	0	0	0	4.2	0.7	2.5	0.6	0	3.2	0.8	0.2	0.4	0	0	0	0.4	7.4	0	0	0.05	4.6	2.8	0	0
Celecoxib	X. Other	Other	0	0	0.3	0	0	0	0	0	0	0	0.2	0	0	0	1.2	0	0	0	0	0	0	0	0	0.5	1.7	0	0	0	0	0	0	0	0	0	0	0	0	0	0	0	0	0.8	0	0.3	0	0	0	0	0	0	0	0	0	0.0	0	0
CEP-32496	B. Kinase inhibitor	RAF inhibitor	3.7	0	0	0.5		1.2	0	0.8	3.1	0	9.6	0	0	4.1		2.9	4.7	0	2.4	0	1.7	2.9	2.4	0	0	1.4	0.1	0	0	3.6	3.9	0	0.1	0	0	0	0	0	2.9	0	0.7	0.8	0	2.5	0	0.9	0.3	9.1	0	0.1	0.05	0.4	3.4	2.5	0	0
CEP-37440	B. Kinase inhibitor	ALK inhibitor	9.8	3.5	3.8	8.6		6.7	7	10	8.9	7	9.7	7.6	11.4	8.7		9.4	7.6	7.2	12.8	5.8	1	6	5.7	8.5	7.2	6.7	10.3	7.4	11.7	9.6	11.2	9.7	7.9	4.3	4.8	3.3	9.1	6.4	9.3	6.1	7.1	0.8	7.5	8	6.7	7.6	8.3	12.8	3.3	8.1	9.25	8.1	10.7	9.2	8.3	8.7
Cerdulatinib	B. Kinase inhibitor	JAK inhibitor	0	9.6	9.2	0		0.3	0	0	0	0	5.2	3.7	0	2.2		0	0	0.2	0	0.7	0	0	5.6	0	3.6	2.7	0	2.5	0	3.6	0	0	0.2	1.4	0.4	0.9	4.7	0	0	1.4	1.8	0.8	0	0.8	2.4	1.1	0	6.1	0.5	0	2	0.4	2.5	0.0	0	3.2
Ceritinib	B. Kinase inhibitor	ALK inhibitor	3.2	3.4	3.8	2.9	1.8	2	2.7	6.4	2.5	3.6	3.2	0	3.1	1.9	1.3	2.6	3	3	3.5	1.5	0.8	0.2	0	1.9	1.4	0	3.1	4	3.8	2.9	2	3.6	3.8	1.8	1.2	0	4	1.4	2.6	3	2.5	0.8	2.5	0.1	1.9	0.8	3.3	3.2	2	2.7	4	2.05	2.3	1.5	3.1	3.3
Chloroquine	A. Conventional chemotherapy	Other antineoplastic	8.1	9.7	10.5	6.7	5.5	4.2	6.6	5.7	6	5.2	7.7	5.7	7.9	6		8.4	7.5	3.8	8.8	5.6	9	6.4	4.2	3.4	5.7	5.7	9.3	7.2	8.4	6.8	9.4	5.6	5.3	4.7	3.9	6.6	9.3	8.9	10.3	2.3	8.3	0.8	8.5	7	6.1	5.9	4.4	8	3.5	11.1	7.75	3.9	8.6	7.8	8	7.4
Cilengitide	X. Other	Other	0	0	0	5.5		0.4	0	0	0	5.7	0	0	0	9.3		0	0	0.2	13	0	7.1	9	0	2	1.7	0	10.2	5.6	12	9.7	14.7	7.8	7.4	0	0	3.8	0	6.2	11.5	7.6	7.1	0.8	6.7	4.6	4.7	0	12.3	0		3.2	0.45	0	13.15	9.5	0	0
Cisplatin	A. Conventional chemotherapy	Other antineoplastic	17.7	8.7	8	10.1	7.2	10.8	1.5	10.6	4.1	16.5	16.3	15.9	5.5	16.3	5.3	15	10.3	5.7	4.1	8.8	2.8	5.4	12.8	12	13.2	13	7.2	7.4	6.7	7.7	5.8	5.5	8.6	6.9	8.1	11.1	6.1	5.7	4.3	7.9	3.9	0.8	6.6	5.8	4.8	13.8	7.8	25.2	7.1	6.3	6.65	15.55	6.05	5.2	12.5	5.9
Cladribine	A. Conventional chemotherapy	Antimetabolite	1.2	14.8	16.4	0.8		0	0	0	0	0.2	13.4	12	0	0		0.7	0	1.1	0	0	0	2.5	0	0	0	0	1.2	1.4	0	0	0	0.9	1.7	0.6	2.7	0	2.2	0	0	0	0	0.8	0	9.1	0	11.3	0	12.3	0	7.5	8.7	1.45	0.6	0.0	0	4.1
Clofarabine	A. Conventional chemotherapy	Antimetabolite	9.8	23.7	27.1	5.5	0	0	3.1	2.7	3.4	3	25.9	22.5	4.4	3.8		6.6	3	1.3	3	6.3	1	5	6.3	5.6	5.1	6	6.9	8.3	4.8	6.1	4.6	3.8	8.1	4	7.9	10.1	11.2	4.2	2	7	3	0.8	1.3	48.3	0	18	0	24.8	1.9	16.4	19.35	8.2	5.55	3.0	3.4	10.9
Clomifene	F. Hormone therapy	Antiestrogen	5	4.8	6.1	1.1	0.7	2.3	2.8	5.5	4.4	3.4	4.4	3.1	4.3	4	4.2	5.5	4.1	5.4	3.9	4.8	0	1.8	6.2	5.9	8	5	2	4	4.7	4	2.6	3.7	4	1.1	1.1	5.4	9.3	2.9	2.7	2.8	3.4	0.8	0.4	4.8	2.4	2.4	2.5	3.3	3.6	5.7	3.5	3.95	3.95	4.8	5.4	1.8
Cobimetinib	B. Kinase inhibitor	MEK1/2 inhibitor	16.1	3.7	3.2	3.7	3	0	5.6	6.2	5	4.4	10.3	3	7.8	10.9		2.5	1.5	3.2	3.9	1.9	0.7	0.5	4.9	3.9	4.8	3.6	5.7	4.1	6.1	1.5	7.4	5.3	4.3	6.4	5.2	3.4	9.5	6.6	5.7	4.1	5.9	0.8	4.5	6	2.2	4.4	4.7	10	0.3	15.1	5.4	3.05	7.3	5.5	2.8	2.2
Copanlisib	B. Kinase inhibitor	PI3K or MTOR inhibitor	24.6	6.8	5.2	11.1		5.6	10.3	11.7	10.8	6	15.3	3.5	10.6	16.5	8.1	12.6	12.3	7.4	13.8	4.4	7.2	7.5	9.4	7	5.3	6.8	11.3	7.4	10.3	11.9	8	8.1	6.1	4.7	4.9	11.2	8.6	6.3	5.6	4.8	6.3	0.8	8.4	12	1.4	6	6.8	16.8	8.1	11.2	8.5	9.2	10.55	9.9	14.1	5.6
CPI-0610	C. Differentiating/epigenetic modifier	BET inhibitor	6.1	3.9	5.8	4.8		0	4.2	1.8	5.2	4.3	12.6	9	9.2	13.2		3	0	0	0.3	6.4	4.9	0	1.9	2.9	2.9	2.3	5.7	7.9	4.6	9.1	4.8	0	7.6	4.5	3.6	3	5.5	4.3	4.2	7.6	4.3	0.8	1	0.1	8.4	9.9	5	12.7		12.2	6.55	0	6.4	5.3	0	0.4
CPI-360	C. Differentiating/epigenetic modifier	HMT inhibitor	0	0	0	0		0	0	0	0	0	0	0	0	0		0	0	0	0	0	2.1	0	0	0	0	0	0	0	0	0	0	0	0	0	0	0	0	0.3	0	0	0	0.8	0	0	0	0	0	0.3	0.4	0	0	0	0.1	0.0	0	0
CPI-613	G. Metabolic modifier	Other metabolic modifier	0	0	0	0		0	0	0	0	0	0	0.4	0.2	0.5		0.7	0	0	0.3	0.3	0	5.8	0	0	0	0	0	0	3.8	0	0	0	0	0	0	0	0	0	0	0	0	0.8	0.4	0	0	0	0	0		0	0	0.35	0	0.0	0.1	0
Crenolanib	B. Kinase inhibitor	FLT3 inhibitor	5.4	9.5	9.1	5.3		1.8	3.8	2.6	4	3.5	4.2	1.4	4	3.8	3.7	2.1	3.8	5.9	4.2	4.6	6.2	5.6	3.3	3.8	3	2.7	4	6.1	4.1	6.2	5.4	4.6	3.3	0.7	1.6	5.3	5	6.9	4.6	1.5	4.1	0.8	2.4	5.6	5.4	4.8	2.8	3.9	0	9.6	8.4	5.2	4.2	4.3	4.3	7.9
Crizotinib	B. Kinase inhibitor	ALK inhibitor	0	1.1	0.7	0	0	0	0	0	0	0	0.4	0	0.4	0	0	0	0	0	0	0	0	1.8	0	0	0	0	0.3	0	2.2	0	0.6	0.1	0	0	0	0.1	0.2	0	0.5	0	0	0.8	0	0	0	0	0	0.7	0	0	1.25	0	1.85	0.5	0	0
CUDC-305	X. Other	HSP inhibitor	16.3	12.2	9.7	4.6		7.5	15	8.9	11.2	0	16.2	10.3	7.5	8.5		7.2	7.7	5.6	6.4	9.6	4.4	4.8	5	4.2	6.4	5.8	9.2	9.8	9.3	10.8	9.2	8.3	8.3	6.5	5.3	0	10.9	8.8	7.5	7.8	10.8	0.8	4.8	12.2	9.9	14.6	3.4	14.8		11.9	12.2	8.45	9.8	9.4	5.3	5.4
CUDC-907	C. Differentiating/epigenetic modifier	HDAC inhibitor	34.9	35.3	36	25.4		22.7	19.6	28.6	27	32.3	41.5	32.1	34.4	35		30.2	25.7	31.3	15.5	28.6	19.2	18.1	32.8	35.7	31.2	27.7	23	24.9	24.5	28.1	19.9	22.7	23	16.1	16.9	27.3	23.5	15.8	20.3	22.6	18.2	0.8	20.4	35.5	28.5	35.6	22	39.1	15.6	35.7	27.9	29.6	24.85	19.8	27.9	13.4
Cytarabine	A. Conventional chemotherapy	Antimetabolite	5.5	10.2	9.8	0.1	0	0	0	0.9	0.1	0	23.1	9.3	0	0	0	1.5	0.3	3.4	0	7.7	3.8	0.1	2.3	0	0.4	5.6	7.1	3.7	4.9	4.5	3.6	3.1	8	1.4	2.5	4	4.7	5.9	0.6	6.2	4	0.8	0.2	0	0	9.6	0	23.7	7	9.2	15.95	7.5	6.45	0.3	0.1	3.4
Cytarabine/Idarubicin	A. Conventional chemotherapy	Other antineoplastic	9.1	11.7	10.5	3.6	0	0	1.5	4.6	2.1	7.2	24.2	6.2	4.2	4.6		6.6	5.6	5	4.4	7.1	5.6	1.6	5.9	1.8	3.5	6	6.3	3.5	6.6	5.3	4.9	6	6.1	3.7	4.6	0	6.8	5	2.8	3.5	2.9	0.8	2.8	37.3	5.3	8.1	2.8	25.9	0	11.3	13.45	7.8	6.8	3.0	4	7.1
Dabrafenib	B. Kinase inhibitor	RAF inhibitor	0.5	0.6	0	1.2	0.2	0	0	0	1.4	0.1	0.1	0	0.6	2.9		0	0	0	2	1.4	0	3	0	0	4	0	0.6	0	3.4	0.8	0.2	0	0	0	0	0.3	1	0	4.4	0	1.4	0.8	0	1.4	0	1	0	0.7	0	3.1	0.1	0.8	1.45	0.1	0	0
Dacomitinib	B. Kinase inhibitor	EGFR or ERBB2 inhibitor	18.8	2.4	2.8	0	8.3	2.5	9.3	12.4	10.6	0.7	2.6	4.8	5.6	16	6.1	8.3	8.8	12.6	2.2	1.8	0	1.8	9.6	8.3	7.8	9.5	0.2	6.3	0.5	3.2	0	0	3.1	0	0	0	2.4	0	0.2	4.4	0	0.8	0	10.9	2.3	1.9	3.1	1.3	0	3.1	5.2	4.1	1.2	0.0	0.2	0.2
Dactinomycin	A. Conventional chemotherapy	Other antineoplastic	34.7	24	23	19.3	17.9	22.6	25.8	23.8	29.3	21.4	39.7	6	20.5	28.6		21.4	18	19.2	6.8	28.1	12.1	12.9	22.8	23.2	17.8	20.1	22.6	14.4	23.6	26.1	19.7	26.6	15.4	15.9	19.1	5.6	22.2	20	19.7	16.9	18.3	0.8	15.2	34.5	22.9	22.7	11.5	39.3	9.4	28.4	26.1	29.3	21.1	21.5	22.8	29
Dactolisib	B. Kinase inhibitor	PI3K or MTOR inhibitor	20.3	8.8	3.8	10.6		11.4	11.6	11.2	14.7	10.9	20.5	12.4	14.7	18.3	12.5	8.3	8	16.6	14	11.3	10.3	16.9	15.7	14.2	17	13.4	17.7	11.4	17.4	13.1	17.1	16.4	13.2	9.1	10.4	17.2	17.2	14.5	15.6	10.9	12.8	0.8	13	20.2	17	15.4	8.6	21.2	7.2	17.6	13.85	14.8	16.25	10.9	14.3	12.2
Danusertib	B. Kinase inhibitor	Aurora kinase inhibitors	12.7	9.1	6.7	7.6		5.1	0	4.9	6.9	5.9	24.5	0	10.7	16		5.2	2.3	3.4	14.2	5.6	12.5	14.5	0	2	1.5	0.3	13.4	6	16.1	9.7	15.5	15	6.6	1.7	3.6	0	7.1	14	11.9	5.4	12.1	0.8	13.5	17.6	9.4	6.2	8.1	23.6	0	10.6	21.55	7.8	14.8	15.3	4.3	5.5
Daporinad	G. Metabolic modifier	Other metabolic modifier	30.7	22	21.9	17.2		18	0	17.4	0	24.6	37	27.1	17.6	33.7		20.3	18.7	0.5	0	17.6	0	3.1	16.6	15.6	13.4	16.7	0.4	0	7.2	0	0.7	0	6.4	8.4	11.6	3.5	15.5	7.2	0	0	7.1	0.8	0	22.5	13.7	30.6	19.9	35.8		19.4	9.6	5.1	2.65	2.4	20.5	21.1
Darapladib	X. Other	Other	0.8	3.1	3.4	0		0	0	0.4	0	1.5	0	0	1.7	0		0	0	0	0.7	0	0	0	0	8.7	2.9	0	0.3	0	0	0.3	0	0	0	0	0	0	0.5	0	0	0	0	0.8	0	0.9	0.1	0	0	0		1.3	0.5	0.55	0.6	0.0	0	0
Dasatinib	B. Kinase inhibitor	BCR-ABL inhibitor	19.1	0	0	13.7	5	8.6	0	12.4	11.3	4.7	0	3.3	19	18.5	6.5	9.6	6.8	1.4	15.6	5.4	9.6	8.9	0	0.9	1.7	0.5	9.5	6.9	16.7	9	14.2	15.6	8.4	0	0	4.5	9.5	7.6	11.5	6.8	9.1	0.8	11.3	16	8.2	1.1	13.5	0	0.6	11.4	12.85	5.4	14.75	15.8	5.7	6.2
Daunorubicin	A. Conventional chemotherapy	Topoisomerase inhibitor	11.1	10.3	9.9	4.6	0	3.1	3.9	4.1	5.9	8	20.7	1.5	5.5	8.6		8.4	5.7	2	5.6	5.3	3.7	4.6	3.3	4.3	4.3	3.6	8.9	4.2	9.9	5.9	7.2	6.7	6	3.8	3.5	0	3.8	6.7	5.5	4.9	7.3	0.8	2.9	8.1	4.5	10.3	3.9	22	1.2	10.6	7.7	9.25	6.5	4.6	8.1	9.5
Decernotinib	B. Kinase inhibitor	JAK inhibitor	0	3.1	2.5	0	0	0	0	0	0	0	1	0	0.5	0		0	0	0	0	0	2.4	0	0	0	0	0	0.1	0.7	0.3	2.4	0.3	0	0	0.6	0.8	2.7	0	1.1	0	0.2	0	0.8	0.9	0	0	0	0	1.9	0	0	0.2	0.1	0	0.0	0	0
Decitabine	C. Differentiating/epigenetic modifier	DNMT inhibitor	1.1	1.5	3.2	0	0	0	0	0	0	0.1	6.2	1.6	0	0.8	0	7.2	1.9	0.4	0	0.4	0	1.8	3.5	0	1.4	0	0	0	0	0	0	0	0	0	0.1	0	3.5	0	2.5	0	0	0.8	0	3.6	2.2	3	0	9.1	0.8	4.1	0.2	0.65	0.15	0.1	4.1	1.6
Deferoxamine	X. Other	Other	4.1	2.9	1.6	4.3	2	1.1	0.1	0.8	2.3	1.9	6.1	1.8	2.1	4.7	0	0.4	0.5	2.9	0.1	1.7	0.1	2.1	0.9	0	0.9	0	2.9	0	1.3	0.5	2.3	1	1.5	0.7	0	1.4	1.1	3.1	0.3	1.1	3.3	0.8	0.8	3.2	1.9	5.7	2.7	5	0.3	6.8	3	2.45	2.55	0.9	0	4.8
DEL-22379	B. Kinase inhibitor	Other kinase inhibitor	10.2	5	13.9	8.3		6.6	6.7	8.8	8.1	7.8	6.9	7.2	11.6	10		10.2	8.2	5.7	5.7	6.1	4.8	8.2	6.2	8.7	6.2	5.6	10.6	7.8	9.4	8.2	9.5	5.3	7.8	5.2	6.8	10.8	5.9	6	8.4	6.6	5.4	0.8	4.6	9.2	4.7	9.6	7.4	5.4	1.7	10.1	9.9	8	6	5.4	6.7	10
Dexamethasone	E. Immunomodulatory	Glucocorticoid	0	0	3.1	0	0	0	0	0	0	0.5	0	0	0.4	0	0	0	0	0	1.1	0		0	0	0	0	0	0	0	0	0	0	0	0	0	0	0	0	0	0	4.3	0	0.8	0	3.3	0	2.4	0	0	0	0	11.05	0	0	0.0	0	0

Digoxin	X. Other	Other	13.5	11.9	9.6	8.3	8.4	11.8
Dinaciclib	B. Kinase inhibitor	CDK inhibitor	23.6	27.3	23.7	13.1	26.4	15.7
Disulfiram(+CuCl2)	G. Metabolic modifier	Other metabolic modifier	2.9	3	1.1	2.3		1
Docetaxel	A. Conventional chemotherapy	Mitotic inhibitor	29.2	11.7	2.1	5.6	5.1	7.4
Doramapimod	B. Kinase inhibitor	p38MAPK inhibitor	0	0	0	0		0
Dovitinib	B. Kinase inhibitor	Multi kinase inhibitor	0	0.2	0.5	0	0	0
Doxorubicin	A. Conventional chemotherapy	Topoisomerase inhibitor	10	9.1	6.9	2.9	0	4.1
Duvelisib	B. Kinase inhibitor	PI3K or MTOR inhibitor	0	0.6	0.6	1.6	0	0
E7820	X. Other	Other	0.4	11.2	6.4	1.2		0
Eltanexor	D. Apoptotic modulator	Other apoptotic modulator	26.3	19.6	21.6	11.2		11
Enasidenib	C. Differentiating/epigenetic modifier	IDH1/IDH2 inhibitor	0	0	0.6	1.4		0
Encorafenib	B. Kinase inhibitor	RAF inhibitor	0.6	0	0	0		0
ENMD-2076	B. Kinase inhibitor	Aurora kinase inhibitors	12.9	10.2	9.1	10.1		5.3
Ensartinib	B. Kinase inhibitor	ALK inhibitor	0.5	2.9	1.5	0.7	0.1	1.1
Entinostat	C. Differentiating/epigenetic modifier	HDAC inhibitor	3	8.9	8.9	1.2		0.9
Entospletinib	B. Kinase inhibitor	Other kinase inhibitor	0	0	1	0		0
Entrectinib	B. Kinase inhibitor	Other kinase inhibitor	0.2	0	1.1	0.7		0.2
Enzalutamide	F. Hormone therapy	Antiandrogen	0	0	0	0.3	0	0.1
Enzastaurin	B. Kinase inhibitor	Other kinase inhibitor	0.4	0	0	0.2	0	0
Epacadostat	E. Immunomodulatory	Other immunomodulatory	0	0	0.1	0		0
Epirubicin	A. Conventional chemotherapy	Topoisomerase inhibitor	9.4	9	9.1	4.3	0	1.2
EPZ-5687	C. Differentiating/epigenetic modifier	HMT inhibitor	0	0	1.7	0		0
EPZ015666	C. Differentiating/epigenetic modifier	Other differentiating/epigenetic modifier	1.7	0	0	0.7		0
EPZ031686	C. Differentiating/epigenetic modifier	Other differentiating/epigenetic modifier	0	0	0	0		0
Erastin	G. Metabolic modifier	Other metabolic modifier	3.1	9.2	12.2	8.2		14.4
Erdafitinib	B. Kinase inhibitor	FGFR inhibitor	0	0.6	0.3	0		0
Eribulin	A. Conventional chemotherapy	Mitotic inhibitor	23.3	12.6	10.8	5.3	2.8	3.4
Erlotinib	B. Kinase inhibitor	EGFR or ERBB2 inhibitor	14.7	0	0	5	9.3	4.6
Etoposide	A. Conventional chemotherapy	Topoisomerase inhibitor	8.4	6	2.9	2.5	0	0
Everolimus	E. Immunomodulatory	Rapalog	5.9	3.6	0	4.9	0	1.8
Exemestane	F. Hormone therapy	Aromatase inhibitor	0.4	0.4	0.3	1.3	0.7	0
Fedratinib	B. Kinase inhibitor	JAK inhibitor	7.6	9	9.6	9.4		2.1
Filanesib	X. Other	Kinesin inhibitor	26.1	9.9	3.1	1		3.6
Filgotinib	B. Kinase inhibitor	JAK inhibitor	2.4	5.6	4.4	0.5		0
Finasteride	F. Hormone therapy	Other hormone therapy	0	0	0	0	0	0
Fingolimod	E. Immunomodulatory	Other immunomodulatory	8.1	6	5.7	7.3	0	6.7
Floxuridine	A. Conventional chemotherapy	Antimetabolite	11.8	1.5	1	1.6	0	0.5
Fludarabine	A. Conventional chemotherapy	Antimetabolite	0	11.6	13.2	0.8	0	0
Fluorouracil	A. Conventional chemotherapy	Antimetabolite	6.2	0.2	0	0	0	0
Flutamide	F. Hormone therapy	Antiandrogen	0	1.1	0.9	0	0.1	0
Foretinib	B. Kinase inhibitor	Multi kinase inhibitor	4.8	4.4	2.3	3.5	1.3	2.1
Fostamatinib	B. Kinase inhibitor	Other kinase inhibitor	0.1	2.4	1.1	0.4	4.9	3.6
FRAX486	B. Kinase inhibitor	Other kinase inhibitor	5.5	3.9	7.2	6		2.2
Fulvestrant	F. Hormone therapy	Antiestrogen	0	0	0	0.7	0	0
Galiellalactone	X. Other	Other	2.7	4.6	8.5	0.1		0
Galunisertib	B. Kinase inhibitor	Other kinase inhibitor	0	0	0	0		0
Gandotinib	B. Kinase inhibitor	JAK inhibitor	7.8	5.8	5.7	4.1		5.2
Ganetespib	X. Other	HSP inhibitor	20.9	18.4	15.4	7.9		9.2
GDC-0084	B. Kinase inhibitor	PI3K or MTOR inhibitor	19.9	6.5	5.3	11.4		6.4
GDC-0623	B. Kinase inhibitor	MEK1/2 inhibitor	18.8	5.7	3.3	1.8		0.1
GDC-0853	B. Kinase inhibitor	BTK inhibitor	0	0	0	0		0
GDC-0919	E. Immunomodulatory	Other immunomodulatory	0	0	0	0		0
Gedatolisib	B. Kinase inhibitor	PI3K or MTOR inhibitor	12	8.4	7.2	11.3		8.6
Gefitinib	B. Kinase inhibitor	EGFR or ERBB2 inhibitor	18.3	1.4	2.1	3.8	10.1	3.4
Gemcitabine	A. Conventional chemotherapy	Antimetabolite	13.2	10.2	3.7	1.7	0	0
Gilteritinib	B. Kinase inhibitor	FLT3 inhibitor	3.8	0	0	5.1	0.5	0
Givinostat	C. Differentiating/epigenetic modifier	HDAC inhibitor	4.9	6.1	7.3	2.6		2
Glasdegib	X. Other	Hedgehog inhibitor	0	0	0	0		0
Glesatinib	B. Kinase inhibitor	Multi kinase inhibitor	0	0.9	2.8	1.7		0.4
GNE-0877	B. Kinase inhibitor	Other kinase inhibitor	0	0	0	0		0
GNE-7915	B. Kinase inhibitor	Other kinase inhibitor	0	0	0.3	0		0
Golvatinib	B. Kinase inhibitor	Multi kinase inhibitor	0	0	0	0.5		0
Goserelin	F. Hormone therapy	Other hormone therapy	0	0	0	0	0	0
GSK-1070916	B. Kinase inhibitor	Aurora kinase inhibitors	1.7	0	0	0		0
GSK-2334470	B. Kinase inhibitor	Other kinase inhibitor	0	0	0	0		0.1
GSK-461364	B. Kinase inhibitor	Other kinase inhibitor	30.2	12.4	8.4	2.4		2.6
GSK-690693	B. Kinase inhibitor	AKT inhibitor	11.1	0.5	0.4	5		2.5
GSK-J4	C. Differentiating/epigenetic modifier	Other differentiating/epigenetic modifier	4.7	9.9	14.1	0.7		1
GSK2256098	B. Kinase inhibitor	Other kinase inhibitor	1.2	0	0	1.3		1.5
GSK2636771	B. Kinase inhibitor	PI3K or MTOR inhibitor	0	0	0	1.9		0
GSK2656157	B. Kinase inhibitor	Other kinase inhibitor	0	0	0	1.4		0
GSK269962	B. Kinase inhibitor	Other kinase inhibitor	10.2	9	1.8	0		2
GSK2801	C. Differentiating/epigenetic modifier	Other differentiating/epigenetic modifier	0	0	0.3	1		0
GSK2830371	X. Other	Other	0	0	0	0.6		0
GSK2879552	C. Differentiating/epigenetic modifier	Other differentiating/epigenetic modifier	0	2.1	0.5	0		0
GSK343	C. Differentiating/epigenetic modifier	HMT inhibitor	0	0	0	0		0
GSK650394	B. Kinase inhibitor	Other kinase inhibitor	3.9	2.5	1.8	0		0
GSK923295	X. Other	Kinesin inhibitor	20.1	11.5	8.3	3.1		0
Hydroxyfasudil	B. Kinase inhibitor	Other kinase inhibitor	1	1.4	0.8	0	0	0
Hydroxyurea	A. Conventional chemotherapy	Other antineoplastic	3	2.7	3	0.3	0	0
I-BET151	C. Differentiating/epigenetic modifier	BET inhibitor	6.2	6.1	6.8	6.4		1.7
Ibrutinib	B. Kinase inhibitor	BTK inhibitor	11.2	0	0	2.2	1.1	1.1
Icotinib	B. Kinase inhibitor	EGFR or ERBB2 inhibitor	10.5	0	0	0		0.7
Idarubicin	A. Conventional chemotherapy	Topoisomerase inhibitor	16	15.2	11	2.9	0.5	2.3
Idasanutlin	D. Apoptotic modulator	p53 pathway modulator	2.9	10.4	13.4	0		0
Idelalisib	B. Kinase inhibitor	PI3K or MTOR inhibitor	0.5	4.2	2.7	2.5	0	0
Imatinib	B. Kinase inhibitor	BCR-ABL inhibitor	0	0	1.5	0.1	0	0
Imiquimod	E. Immunomodulatory	TLRs ligand	0.1	0	0	0	0	0
Indibulin	A. Conventional chemotherapy	Mitotic inhibitor	6.8	1.9	0	2.4		8.1
Infigratinib	B. Kinase inhibitor	FGFR inhibitor	0	0	0	0		0
IOX-1	C. Differentiating/epigenetic modifier	Other differentiating/epigenetic modifier	3	0.9	1.6	2.5		0.2
IOX-2	C. Differentiating/epigenetic modifier	Other differentiating/epigenetic modifier	0.2	3.2	0.8	0		0.5
Ipatasertib	B. Kinase inhibitor	AKT inhibitor	11.1	2.1	1.3	5.7		0.6
Itraconazole	X. Other	Other	3.9	3	3.5	1.3	0.7	0
Ivosidenib	C. Differentiating/epigenetic modifier	IDH1/IDH2 inhibitor	0.2	0	0	0		0
Ixabepilone	A. Conventional chemotherapy	Mitotic inhibitor	5.9	2.1	0.5	4.1	0	1.3
Ixazomib	X. Other	Protease/proteasome inhibitor	8.8	14	12.5	7.6	13.1	9
JPH203	X. Other	Other	0	0	0	0		0
JQ1	C. Differentiating/epigenetic modifier	BET inhibitor	9.7	7.8	10.3	7		2.3
KD025	B. Kinase inhibitor	Other kinase inhibitor	2.6	1.3	2.1	1.1		0
KU-60019	B. Kinase inhibitor	Other kinase inhibitor	1.9	1.1	2.8	0.7		2.3
Lapatinib	B. Kinase inhibitor	EGFR or ERBB2 inhibitor	9.8	0	0	0	1.5	0
Larotrectinib	B. Kinase inhibitor	Other kinase inhibitor	0	0	0	0		0
Lasofoxifene	F. Hormone therapy	Antiestrogen	0	0	0	0	1.3	0
Lenalidomide	E. Immunomodulatory	Other immunomodulatory	0	1.6	1.8	0	0	0
Lenvatinib	B. Kinase inhibitor	Multi kinase inhibitor	0	0	0	0	1	0
Letrozole	F. Hormone therapy	Aromatase inhibitor	0	0	0.2	0	0	0
Linifanib	B. Kinase inhibitor	Multi kinase inhibitor	0	0	0	0	0	0.3
Linsitinib	B. Kinase inhibitor	Other kinase inhibitor	0	3	2.8	4.6		1.4
Litronesib	X. Other	Kinesin inhibitor	25.9	10.6	2	0		3.4
Lomeguatrib	C. Differentiating/epigenetic modifier	Other differentiating/epigenetic modifier	0	0	0	0		0
Lonafarnib	C. Differentiating/epigenetic modifier	Other differentiating/epigenetic modifier	14	12.3	11.9	10.1		6.4
Losmapimod	B. Kinase inhibitor	p38MAPK inhibitor	0	0	0	0	1.3	0
Lovastatin	G. Metabolic modifier	HMGCR inhibitor	0	0.5	0	0	2.1	0
Lucitanib	B. Kinase inhibitor	Multi kinase inhibitor	2	2.6	0.2	0.2		0
Luminespib	X. Other	HSP inhibitor	23.2	20.8	17.7	11		11.7
LY-2584702	B. Kinase inhibitor	Other kinase inhibitor	1.2	0	0	0		0
LY-2874455	B. Kinase inhibitor	FGFR inhibitor	7.6	6.3	4.5	4.2		4.9
LY3009120	B. Kinase inhibitor	RAF inhibitor	15.5	4.5	5.1	9.2		4.7
LY3023414	B. Kinase inhibitor	PI3K or MTOR inhibitor	14.5	6.1	4.7	10.1		8.5
Marimastat	X. Other	Other	1.7	0	0.1	0		0
Masitinib	B. Kinase inhibitor	Multi kinase inhibitor	0.7	3.7	7.1	0	0	0
Megestrol acetate	F. Hormone therapy	Other hormone therapy	0	0.7	1.1	0	3.7	0
Mepacrine	X. Other	Other	18.1	18.3	19.5	9.8	7.5	8.7
Mercaptopurine	A. Conventional chemotherapy	Antimetabolite	7.1	0	0	0	1.4	0
Merestinib	B. Kinase inhibitor	MET inhibitor	0.1	0	0.3	0		1
Metformin	G. Metabolic modifier	Other metabolic modifier	0	0	0.1	0	0	0
Methotrexate	A. Conventional chemotherapy	Antimetabolite	27.6	0	0.2	0	0	0
Methylprednisolone	E. Immunomodulatory	Glucocorticoid	0	0	3.8	0	0	0
Midostaurin	B. Kinase inhibitor	Multi kinase inhibitor	6.4	4.8	3.2	13.5	6.3	8.9
Milciclib	B. Kinase inhibitor	CDK inhibitor	15.4	9.4	9	13.2		5.1
Miltefosine	X. Other	Other	6.2	6.8	4.4	2.9	10.9	6.2
Mitomycin C	A. Conventional chemotherapy	Alkylating agent	6.5	6.4	4.7	0.8	0	0
Mitotane	A. Conventional chemotherapy	Other antineoplastic	0	0	0	0	0	0
Mitoxantrone	A. Conventional chemotherapy	Topoisomerase inhibitor	10.7	14.4	14.8	3.8	0	1.2
Mivebresib	C. Differentiating/epigenetic modifier	BET inhibitor	17.7	17.4	20.2	15.4		9.4
MK-0752	X. Other	Other	0.2	0	0	0		0
MK-2206	B. Kinase inhibitor	AKT inhibitor	2.9	3	1.4	3		0.1
MK-8745	B. Kinase inhibitor	Aurora kinase inhibitors	6.4	0	0	2.1		1
MK-8776	B. Kinase inhibitor	Other kinase inhibitor	0	1.4	0.2	0		0
ML323	X. Other	Other	0	0	0.1	0.1		0
ML390	C. Differentiating/epigenetic modifier	Other differentiating/epigenetic modifier	7	0	0	0		0
Mocetinostat	C. Differentiating/epigenetic modifier	HDAC inhibitor	9.8	10.8	10.5	5.8		4.6
Molibresib	C. Differentiating/epigenetic modifier	BET inhibitor	3.9	4.3	5.7	4.4		0
Momelotinib	B. Kinase inhibitor	JAK inhibitor	5.5	9.6	8.9	2.1	3.3	1.6
Motesanib	B. Kinase inhibitor	Multi kinase inhibitor	0	0	0	1.3		1.4
Motolimod	E. Immunomodulatory	TLRs ligand	0	2.6	3.4	0		0
MST-312	X. Other	Other	14	7.3	8.2	6		1.8
Mubritinib	B. Kinase inhibitor	EGFR or ERBB2 inhibitor	2.7	5.1	0	1.9		0.5
Napabucasin	X. Other	Other	18	18.4	20	16.2		15.1
Navitoclax	D. Apoptotic modulator	BH3 mimetic	0	4.5	14.6	0.1	19	0.8
Necrostatin 2	D. Apoptotic modulator	Other apoptotic modulator	0	0	0	0		0
Neflamapimod	B. Kinase inhibitor	p38MAPK inhibitor	1.2	0	0	0		0
Nelarabine	A. Conventional chemotherapy	Antimetabolite	0	4.1	12.3	0	1.6	0

Neratinib	B. Kinase inhibitor	EGFR or ERBB2 inhibitor	14.9	0.3	0	4.1	6	2.9	5.4	4.8	7	0	5.1	3.2	5.7	13.2		6.8	5.8	6.5	3.9	2.2	0	3.2	7.3	4.3	3.5	6.1	3.4	5.6	2.3	3.9	2.8	1.9	6.1	0.3	0.7	0	4.8	1.4	4	5	1.1	0.8	0	7.4	1.1	4.9	1.6	4.4	1.5	4.9	4.85	4.3	4.4	1.0	2	3.3
Nilotinib	B. Kinase inhibitor	BCR-ABL inhibitor	0.3	0	0	0.1	0	0	0	0	0	2.8	2.2	1.6	0	0		1.1	0	0	2.9	0	0.8	1.9	1.7	3.3	3	2.7	0	0	0	0	1.8	0	0	0	0	0	0	1.4	0	0	3.4	0.8	0	4.6	0	3	1.6	1.4	5.1	0	0	3.95	0.9	2.1	0	0
Nilutamide	F. Hormone therapy	Antiandrogen	0	0	0	0	0.3	0	0	0	0	0	0	0	0	0		0	0	0.5	0	0	0	0.1	0	0	0	0	0	0	0.2	0.2	0	0	0	0	0	0	0	0	0	0	0	0.8	0	0	0.3	0	0	0	0	0	0	0	0	0.1	0	0
Nintedanib	B. Kinase inhibitor	Multi kinase inhibitor	9.9	2.6	6	8.6	1.5	1.8	2.6	9.1	4.9	7.4	8.5	0.3	12.2	9.7		0	0	2.8	10.1	0.5	2.9	7	5.1	3.9	6.2	2.1	10.3	2.3	11.7	4.2	13.9	11.1	3.2	2	1.4	12.8	3.7	10	6.6	1	9.6	0.8	7.4	8.4	2.4	8.7	7.4	8.2	1.2	2.3	5.5	6.6	11.4	10.6	6.3	4.1
Niraparib	C. Differentiating/epigenetic modifier	PARP inhibitor	1.9	1.4	1.2	0		0	0	0	0	0.1	12.1	1.7	1	0.1	0	1.3	1.3	0	0.3	3.5	0	5.7	0.4	0	0.7	0	2.9	0	1.7	0	0.9	0.1	0	0.8	0.6	0.3	9.5	0	0	0	0	0.8	0	2.2	0	4.2	0	12.7	0	6	5.3	2.65	3.7	0.4	0.5	0.9
NMS-873	X. Other	Other	4.2	9.5	9.6	6.4		5.8	8.5	5.9	6.7	6.8	20.6	15.5	8.8	8.6		7.9	8.3	9.5	6.1	9	4	8.5	8.3	7.3	8.3	6.9	5	7.2	6.2	7.9	7.5	6.5	7.6	7.5	13.2	5.7	8.9	6.1	10	8.5	5.4	0.8	6.8	9.4	8.1	10.4	5.8	19.1		8.9	8.65	10.15	6.2	7.0	9	4.7
NVP-AEW541	B. Kinase inhibitor	Other kinase inhibitor	3.5	6.5	6.2	8.6		7.8	7.4	16.6	9.2	5.6	8.8	8.4	7.1	6.4		12.4	10.8	5.2	8.7	3.8	4.4	1.8	8	6.7	7.6	6.2	6.9	10.2	7.1	7.8	7.9	6.4	7	6.1	6.4	8.5	10.6	5.9	8.7	3.9	1.9	0.8	5.2	3.8	0	7.7	6.9	11.6	3.2	3.3	4.55	10.25	7.25	7.7	11.4	5
NVP-BGT226	B. Kinase inhibitor	PI3K or MTOR inhibitor	25.2	11	9.1	13.7		14.7	16	16.3	18.4	14.7	23.2	10.5	19.9	23.7		14.8	10.7	15.1	15.4	11.9	8.7	18	15.9	16.4	17.3	13.2	21	13.4	22.7	11.9	21.5	15.9	13.5	9.6	10.6	21.7	16.4	14.1	21.8	11.2	14	0.8	14.9	21.9	15.7	16.2	11.6	23.5	4.7	22.2	16.85	15.75	20.05	16.7	16.3	14.9
NVP-BHG712	B. Kinase inhibitor	Other kinase inhibitor	1.3	2.3	2.4	0.1		0.2	0.8	0.4	0.5	1.6	3.9	0.7	0	2.8		3.6	1.9	2.8	4.4	0	5.2	3.6	4.1	2.1	0.7	6	5.3	1.3	5.2	7.3	4.7	1.3	1.4	0	0	2.4	0	1	3.5	1.5	0.3	0.8	5.5	5.9	0.4	4	3.4	3.2	2.6	0	3.1	3.95	4.8	2.9	1.4	0
NVP-CGM097	D. Apoptotic modulator	p53 pathway modulator	8.1	10.6	12	4.4		6.7	7.4	6.1	8.6	7.1	18.9	8.4	9.6	7.3		7.4	6	7.6	4.7	6.8	0.9	7.7	5.5	5.6	6.1	4	6.3	9.2	8.1	9.3	8	9.4	8.9	5.9	6.7	6.5	7.6	5.2	6.4	9.1	4.9	0.8	3.4	10.1	6.9	7.3	6.5	17.7		8.5	7.7	6.65	8.25	8.9	5.7	1.4
NVP-LCL161	D. Apoptotic modulator	Other apoptotic modulator	0.2	6.4	7.9	13.7		1.6	0.6	1.5	0	2.7	4.1	8.2	3.3	0	0.1	18.6	13.8	9.6	0	14.5	0	0.9	2.5	4.6	0.6	5.4	0.4	0.1	2.1	1.6	0.6	0	0.7	0.2	0.2	0.7	7.9	0	0.9	0	0	0.8	0.2	4.5	21	14.9	1	1	2.5	5.2	17	6.55	0.1	0.1	13.5	1.1
NVP-LGK974	X. Other	Other	0	1.2	3.4	0		0	0	0	0	0	5.6	1	0	1.3		0	0	0	0	0	0	4.3	0	0.6	0	0	0	0	0	0	0	0	0	0	0.5	0	0.1	0	0	0	0	0.8	0	0.1	0	1.1	0	2.5		0	0	0	0	0.0	0	0
NVP-RAF265	B. Kinase inhibitor	RAF inhibitor	2.1	0.3	0	3.9		0	0.8	0	2.9	0	5.4	2.6	1	5.2		4.5	0.6	6.5	5.6	0.4	7	4.4	0.1	2.6	1.8	0	4.7	0	7.4	3.5	5.3	2	1.4	0	0	5.3	1.9	1.1	13.3	1.4	3.8	0.8	2.5	2.4	0	1.8	0	2.7	0	11.3	0.5	4.5	6.5	4.3	2.7	2.8
NVP-SHP099	X. Other	Other	5.2	2.4	2.5	1.9		0	0	2.8	2.8	2.2	2.3	0	1.3	3		0	0	0.5	0.9	0	0	0.6	0	1	0	0	0.3	0.1	0	0	3.9	0	0.1	0	0	0	0	0	0	0.2	0	0.8	0	4.5	0	0.8	2.5	4.4		1.3	2.85	1.35	1.7	1.2	2.7	0
ODM-201	F. Hormone therapy	Antiandrogen	0	0	0	0.5		2.2	0	0	0	1.3	3.5	0.8	0	3.6	0	1.2	0.3	0.3	0	0.3	0	0.1	0	0	0	0	0	0	0	0	0	0	0	0	0	0	0	0	0.1	0	0	0.8	0	0	0	0.9	0.9	1.8	0	2.5	0.2	2.05	0.1	0.0	0	0.6
Olaparib	C. Differentiating/epigenetic modifier	PARP inhibitor	0	2.8	0.8	0.6	3.6	0	0.5	2.8	0.8	0.2	14	0	1	0.3		1.5	3.3	3.1	1.6	3.5	0.8	1.4	3.1	0	1.1	4.9	4.5	0	0.6	1.3	2.4	0.9	2.6	0	0	1.5	4.9	1.8	2.7	0.5	0.1	0.8	0	0	0	2.8	2	10.8	7	7	3.75	3.05	2.05	1.5	1.9	0.6
Olmutinib	B. Kinase inhibitor	EGFR or ERBB2 inhibitor	2.1	0	0.1	0		0	0	0	0	0	0	0	0.3	1.8		0.5	0	1.2	0	0	0	0	0	0	0	0.1	0	0.1	0	0.2	0	0	1.1	0	0	0	0	0	0	0	0	0.8	0	0.2	0	0	0	0.9	0	0	0	0.15	0.05	0.0	0.3	0
Omacetaxine	A. Conventional chemotherapy	Other antineoplastic	31.3	26	27.3	16.8	13.7	14.9	12.2	18	20.1	15.1	35.5	8.3	28.8	28.4	26.2	20.3	16.5	18.3	16.7	20	3.2	10.4	21.5	21.7	21.3	18.2	24.8	12.9	27	17.5	19.8	22.8	14.9	10.4	11.6	26.1	16	17.1	16.3	13	20.7	0.8	22.8	29.3	28.6	25.1	18.1	34.9	8.4	38.8	28.8	23.75	22.45	16.6	24	28.8
Omaveloxolone	X. Other	Other	16.7	16.7	18.2	11.6		10.1	11.8	15.4	16.4	14.7	22.5	18.4	12.3	18.1		16.4	13.4	11.5	3.7	17.9	7.3	7.2	10.1	8.2	9.4	10	12.8	16.7	12.9	20	8.5	10.3	15.8	11.9	12.6	6.9	16.4	12.8	14.4	13.9	13	0.8	8.1	18.7	13	14.9	10.8	23.3		18	12.2	19.6	7.75	7.7	15.6	10.6
Omipalisib	B. Kinase inhibitor	PI3K or MTOR inhibitor	31	8.8	6.4	17.3		10.7	16	16.6	21.8	12.2	28.2	17.7	18.6	25.5	16.9	22.4	17.8	20.3	15.9	15.2	13.1	14.9	16.6	15.5	14.5	14.8	17.1	16.7	17.6	21	15.3	15.5	15.2	13	13.9	18.5	17.3	12.2	15.5	14.9	12.7	0.8	16.4	18.1	12.9	14.3	16.5	28.1	14.7	10.3	12.95	17.6	17.85	15.1	22.1	10.8
Onalespib	X. Other	HSP inhibitor	15.6	13.5	11.8	4.3		5.4	14.4	8.2	12.5	1.1	20	9.9	8.5	9.1		7.6	6.7	7.5	6.5	11.6	5.8	7.5	7.4	8.3	6.8	5.8	8.5	9.7	9.1	10.9	11.9	7.9	9.2	3.1	1	4	9.5	10.9	9.6	9.1	9.8	0.8	6.2	13.2	7.9	14.9	3.3	18.6		11.5	12.8	9.45	9.4	10.1	6.3	6.9
ONX-0914	X. Other	Protease/proteasome inhibitor	17.8	22.5	26.4	16.5		11.2	10.5	12.6	11.9	15.1	19.1	0.3	19.3	17.8		16.2	13.3	14.8	13.4	16.5	8.3	14.6	14	14.8	14.9	12.6	15.4	16.1	16.5	18.4	16.3	17.6	19.3	10	11	5.2	17.2	15.6	12.9	15.9	14.1	0.8	10.6	18.2	17.1	17.6	9.2	16.7		21.1	19.95	19.45	16.75	17.4	15.9	12.3
Oprozomib	X. Other	Protease/proteasome inhibitor	13.8	16.9	18.9	12.9		9.4	1.2	13.7	7.9	16.6	25.8	6.6	21.1	19.9		18.4	15.3	12.6	7.6	17.5	2.2	3.7	17.5	7.7	11.1	15.2	10.7	10.3	11.5	9.4	11.4	11.6	11.7	8.9	11.4	11.7	19.1	11.7	7.9	11.4	10.3	0.8	9.4	21	15.4	17.2	8.8	22.4	6.7	20.3	21.1	17.95	11.9	12.4	18.6	11.5
Orteronel	F. Hormone therapy	Antiandrogen	0	0	0	0		0	0	0	0	0	0	0	0	0		0	0	0	0	0	0	0	0	0	0	0	0.9	0	0.5	0	0	0	0	0	0	0	0	0	0	0	0	0.8	0	0	0	0	0	0	0	0	0	0	0	0.0	0	0
Osimertinib	B. Kinase inhibitor	EGFR or ERBB2 inhibitor	11.9	6.6	7.1	2	4.5	1.6	5.7	7	8.4	3.9	4.9	4.1	6.4	11.7	7.3	5.9	6	7.2	6.4	2.9	1.9	2.5	6.4	5.7	5.6	7.1	3.9	4.8	3.6	3.3	5.9	3.4	7.2	0	0.3	4.5	5.1	4	1	4.5	2.1	0.8	1.5	7.2	1.2	2.4	3.9	6	4.4	4.3	4.75	5	4.15	2.7	3.6	1.8
OSU-03012	B. Kinase inhibitor	Other kinase inhibitor	1.9	0	0	0		0	0	0	0	0	2.4	0	1.2	0		0	0	0	0	2.9	0	0	0	0	0	0	0	0	0	0	0	0	0	0.3	0.1	0	1.2	0	2	0.1	0	0.8	0	0	0	0.8	0	0.3	0	0.1	0.35	0	0	0.0	0	0
OTS-964	B. Kinase inhibitor	Other kinase inhibitor	16.5	13.6	15.2	11.9		12.6	12.9	13.5	14.7	12	18.2	10.6	16.9	14.5		12.9	11	9.5	7.1	13	8.1	9.1	11.2	15.3	9.4	11.1	16.1	13.8	16.1	13.8	12	10.1	13	4.3	5	15.2	13.9	10.2	12	6.3	13.3	0.8	13.4	14.9	12.8	15.4	10.2	17.9	4.1	18.3	14.3	13.9	12.05	9.4	12.3	15.4
OTS167	B. Kinase inhibitor	Other kinase inhibitor	20.3	17	18	6.8		9.9	9.5	15	14.2	7.7	22	8.8	21.9	21.1		17	15	11.5	4.6	18.6	0.6	3.5	11.1	12.7	11.9	10.7	6.9	7.3	9.7	9.1	8.6	6.8	7.1	4.8	3.7	9	9.7	6.8	7.3	8.5	6.7	0.8	7.3	18.1	8.4	15.1	9.6	23.3	3.2	19.4	17.5	13.8	5.8	2.5	17.6	11.3
Oxaliplatin	A. Conventional chemotherapy	Other antineoplastic	18.8	10.5	10.8	6.5	2	5.6	4.3	4.9	5	9.1	26	11.2	2.2	9.3		8.4	6.7	8.3	3.8	7.9	0	4.6	8.1	7.5	5.4	8.4	4	5.4	5	4.4	4.3	0.3	6.1	5.1	6.1	5.5	5.5	2.9	3	2.5	1.1	0.8	0	13.8	11.6	10.7	2.3	25.5	2.5	8.2	8.95	10.35	3.8	0.5	11.2	7.3
PAC-1	D. Apoptotic modulator	Other apoptotic modulator	4.2	2.3	2.6	0.3		0	0.4	0	0	3.7	4.2	5.6	1.8	5		2.7	1	3.7	0	4	0.7	9.5	1	4.6	3.1	0	3.7	0	3.4	0	2.3	1.9	0.9	0	0	2.9	0.1	2	4.4	0.4	2.4	0.8	2.4	4	1.7	4.1	0.4	3.7		5.6	5.6	2.85	4.1	2.4	3.7	2.9
Paclitaxel	A. Conventional chemotherapy	Mitotic inhibitor	26.1	11	1.9	5.4	2.9	4.8	4.5	7.1	8.9	9.5	29.4	3.7	24	23	7.8	18.6	7.8	7	17.3	21.4	12	6.5	8.6	10.2	10.9	11.3	15.2	8.4	18.3	15.3	17.9	13.6	14.9	1.6	1.9	0	7.8	9.6	22.2	13.7	16.1	0.8	21.6	36.6	15.1	23.3	6.3	29.4	2.9	23.8	24.95	18.9	22	16.0	5.9	14.2
Pacritinib	B. Kinase inhibitor	JAK inhibitor	12.4	13	14.5	9.5	2.6	6.2	4.7	5.8	7.3	8.5	15	8.8	12.6	9.1		7.8	6.6	8.6	8.6	7.7	1.7	8.1	6.3	5.4	7.6	5	8.8	4.8	9.5	7.4	9.5	7	6.9	4.7	4.7	0	7.3	6.4	6.9	3.8	5.2	0.8	6.3	7	8.6	9.3	8	12.4	1.5	13.8	10.3	8.4	7.3	4.5	7.3	10.5
Palbociclib	B. Kinase inhibitor	CDK inhibitor	0.9	6.6	5.8	0	0	0	0.5	0.9	3.7	0.8	11.3	0	2.4	0.5	1.1	1.7	1.3	0	0	0	0	1	3	2.2	3.2	1.6	4.2	3.3	3.4	3.5	4.7	0	4.7	1.1	0.7	2.7	3.2	7.4	1	3.7	1.7	0.8	0	2.2	1.1	0.3	0	11.2	0	7.6	2.05	1.8	4.95	0.0	0	0.6
Palomid-529	B. Kinase inhibitor	PI3K or MTOR inhibitor	0	0	0	0		0.1	1.8	0	1.3	0.7	1.4	2.5	0	2.7		1.6	0	0.5	0	0	0	1.4	0	0	0	0	0	0.2	0	1.4	0	0.5	0	0	0	0	0	0	0	0	0	0.8	0	0	0.4	0.6	0.2	5.4	1.1	0	0	2.3	0	0.0	0	0
Panobinostat	C. Differentiating/epigenetic modifier	HDAC inhibitor	15.2	15.9	15	6.9	8.7	7.2	5.8	9	13.2	13.6	17.8	8.6	19.1	16.2		10	9.9	8.1	9.4	10.7	7.2	5.2	17.2	14.8	14.5	14.1	9.6	7.4	11.9	6.7	8.5	9.4	9.1	6.3	6.8	0	10.2	5.4	8.1	6.4	7.9	0.8	8.7	16.1	13.2	18.4	10.1	15.3	6.9	19.5	14.65	11.85	10.75	4.4	10.4	10.8
Pazopanib	B. Kinase inhibitor	Multi kinase inhibitor	0	1.5	1.8	0	0	0	0	0	3.3	0	2.2	0	0	0	0	1.6	0.3	0	7.7	0.3	3.9	2.3	2.7	3.8	1.2	2.3	5.2	0	6.6	9.3	5.9	9	0	0	0	7.3	3.9	4.1	1.6	0.6	2.4	0.8	3.4	0	0	0	0	0.6	7.1	0	0	0.1	6.95	6.2	0	0
PCI-34051	C. Differentiating/epigenetic modifier	HDAC inhibitor	1.5	0	3.3	0.7		0	0	0	0	1.7	3.3	6.4	1.4	5.2		0	0	2	4	0	0	2.7	0.7	0.4	1.8	0.2	1.2	0	3	0.2	3	0	0	0	0	0.2	0.1	0.7	0.4	0.2	0	0.8	0.3	2.5	2.8	3.2	0.8	2.6	0	2.4	0.5	0.75	2.45	0.3	0.2	1
PD0325901	B. Kinase inhibitor	MEK1/2 inhibitor	17.7	4.9	3.4	5.2		0.7	8.4	7.2	9.8	5.2	14.4	8.4	7.9	12.5		2.3	5.9	11.8	0	2.2	0.7	0.2	6.6	3.6	4.6	0	1.1	4.9	2.7	0.9	4.2	3.8	5.8	8.3	6.2	2.6	9	5.8	2	5.7	4.5	0.8	3.9	5.9	1.5	3.3	5.8	16.6	1.8	16.4	6.8	3.65	5.75	3.4	1.9	2
Peficitinib	B. Kinase inhibitor	JAK inhibitor	0.1	5.1	1	0	0	0	0	0	0	0	0.9	0	0	0		0	0	0	0	0	0	0	0	0	0	0	0.5	0	1	0.3	0.8	0.4	0	0	0	1	0	0.8	0.3	0	0	0.8	0.4	0	0	0	0	1.7	0	0	0.1	0	0.5	0.0	0	0
Pemetrexed	A. Conventional chemotherapy	Antimetabolite	9.8	0	0	0	0	0	0	0	1	2.8	13.9	4.5	1.5	1.4		0	0	0	0	1.4	0.3	0	0	0	0	0	0	0	0	0	0	0	0	0	0	0	4.1	0	0	0	0	0.8	0	0.7	0	13.1	0	15.7	0	6.5	8.3	0	0	0.0	0	0
Pentostatin	A. Conventional chemotherapy	Antimetabolite	0	0	0	0	0	0	0	0	0	0.1	1.1	0	0	0		0	0	1.1	0	0	0	0	0	0	0	0	0	0	0	0	0	0	0	0	0	0	0	0	0	0	0.5	0.8	0	3.9	0	0	0	0.9	0	0.3	0	0	0	0.0	0	0
Perifosine	B. Kinase inhibitor	AKT inhibitor	0	0.7	0	0	0	0.1	0	0	2.6	0	0	0	0	0.2		0	0	0	0	0	0	0	0.5	0	0	0.6	0	0	0	0	0	0	0	0	0	0	2	0	0	0	0	0.8	0	0.5	0	0	0.4	1.8	0	0	0.45	0	0	0.0	0	0
Pevonedistat	G. Metabolic modifier	Other metabolic modifier	17.8	5	3.3	4.7		5.9	8.5	9.8	12.9	3.8	18.8	4.3	0.7	4.3	7	2.4	0.9	0.5	10.7	16.6	7	9.2	4.6	8.2	8.1	4.9	7.8	9.8	10.1	9.6	10.2	7.7	9.9	3.7	5	3.9	13	7.8	11.3	10.3	12.1	0.8	5.7	15.3	2.4	16.8	0.8	20.2		16.1	19.1	15.15	11.55	11.5	2.4	9.2
Pexidartinib	B. Kinase inhibitor	Multi kinase inhibitor	0	0	2.3	0	1.5	0.3	0	1.7	0	0.2	2.6	0.2	0.8	0		1.8	2.5	0	6.3	1	1.9	2.5	1.1	2.6	0	2.1	1.7	3.2	3.4	3.4	2.9	2.2	3.8	0	0	2.3	0.7	4.1	3.5	1.9	0.6	0.8	2	0	1.5	0	0	3.2	3.4	0	0.55	1.75	2.95	3.0	0	0
PF-00477736	B. Kinase inhibitor	Other kinase inhibitor	12.9	9.4	7.4	10.7		8.7	3.2	11.8	4.5	7.2	16.5	1	16.5	16.9		13.3	7.8	6.2	4	7	3	4.4	9.8	9.9	7.2	11.7	10.5	2.7	9.7	3.9	6.5	3.1	3.2	4.1	4.6	0	3.9	7.8	6.5	3.7	7.1	0.8	3.2	13.2	11.6	14.5	6.1	15.8	4.5	14.9	12.45	9.5	5.6	0.9	7.5	10.7
PF-00562271	B. Kinase inhibitor	Other kinase inhibitor	8.8	4.3	4.6	7.9		4	4.3	4.1	7.2	4.6	10.3	1	7.8	8.9		6.6	3.5	7.1	7.2	8.4	0.7	7.3	3.5	3.4	5.3	2.6	7.4	6.4	12	6.6	6.9	6.6	6.2	2.4	3.6	9.5	8.2	4.8	7.4	6	5.7	0.8	5.1	9.1	6.6	5	4.2	9.9	0	8.2	5.95	5.75	7.5	5.9	6.9	6.3
PF-03758309	B. Kinase inhibitor	Other kinase inhibitor	25.6	9.1	11.5	12.4		5.4	10.6	6.9	7.6	11.8	38.8	1.9	16.1	26.1		14.1	10.2	9.3	8.6	16.6	3.5	5.1	14.3	9.9	8	5.4	15.6	9	21.7	8.8	15	10.1	12.1	6.4	6.6	15.8	12.3	9.5	13.6	6.4	10.4	0.8	12.4	27.1	16.5	14.3	15.3	33.3	3.9	32.9	22.9	15.6	16	13.0	13.7	18.4
PF-04708671	B. Kinase inhibitor	Other kinase inhibitor	0.5	0.6	1.7	0		0	0	0.4	0	0.2	5.5	2.1	0.9	0.3		1.6	0.9	0.2	0.4	1.6	3.4	0	1.2	5.6	1.3	0	0.5	0	0	0.7	0	0.1	0	0.9	1.4	0.1	4.1	0.9	0	0.5	0	0.8	0	0.2	1.1	1	1	2.3	3.6	0	0.55	0.55	0.05	0.0	1.8	0.4
PF-06463922	B. Kinase inhibitor	ALK inhibitor	2.8	0	0	0.2		1.7	1.2	0.4	1.3	1.6	1.9	0	1.2	3.8		0.2	0.9	0.8	4.7	0	0	3.7	1.5	0.9	1.8	0	2.5	1.9	7	1.8	3.1	1.7	1.9	0.4	0	4.2	3.6	2.1	3.7	0.1	1.5	0.8	3.1	1.1	0.5	0.8	1.8	3.1	0.3	1.2	2.5	0.3	3.7	3.6	0	3.2
PF-3845	X. Other	Other	0	1	1.1	0.2		0	0	0.1	0.1	0.3	5.7	6.3	0	1.4		0.2	0.3	0	0	0.6	0	4	0.3	0.2	0.6	0.5	0	0	0	0	0	0	0	0.8	2.4	0	1.7	0	0	1.3	0	0.8	0	2.5	0	3.1	0.7	4.8		0.6	0	0	0	0.0	1	0
PF-4800567	B. Kinase inhibitor	Other kinase inhibitor	0	0	0	0		1.2	0	0.5	0.8	0.6	2.2	0.1	1.1	4.1		0.5	0.7	0	0	0	0	0	0	0	0	0.4	0	0.3	0	1.9	0	0	0	0	0	0	1.6	0	0	0	0	0.8	0	0	0.2	1.1	2.2	1.2	0.2	0.1	0.7	0	0.1	0.0	0	0
PF-670462	B. Kinase inhibitor	Other kinase inhibitor	4.9	0	0	0		2.2	1.4	2.8	2.3	0	4.4	1.3	0.4	3.4		1.9	1	0	0	0	0.2	0	2.3	4.6	1.9	2.5	0	2.4	0	3.2	0	0	1.4	0	0	0	1.1	0.1	0	2	0	0.8	0	0.4	0	0.9	0.1	6	0.3	0	1.45	1.9	0	0.0	0.4	0.3
PF06650833	B. Kinase inhibitor	Other kinase inhibitor	1.3	0.5	0.3	0		1.6	0.9	1	2.2	0.7	6.2	0	1.4	4		0.8	1.1	0	0	1.1	0	0	1.6	4.7	2	0	0	0	0	0	0	0	0	0	0	0.2	0.7	0	0	0	0	0.8	0	1.4	2.5	1.9	0.9	6.1	0	2.6	1.7	1.1	0	0.0	1.3	0.6
PFI-1	C. Differentiating/epigenetic modifier	BET inhibitor	6.4	2.3	4.8	5.4		0.9	4.9	3	5.8	4.6	9.9	3.2	6.9	11		2.9	2.8	4.6	2.5	4.8	2.1	3.3	4	5.8	3.6	0.1	4.6	4.2	5	4.8	5.5	1.9	3.9	2.9	3.2	4.9	4.8	1.9	1.6	3.8	2.4	0.8	1.1	6	4.3	8.4	7.2	12.1	0	7.9	6.9	7.9	3.3	3.2	3.1	0.4
PH-797804	B. Kinase inhibitor	p38MAPK inhibitor	0	0	0	0		0	0	0	0	0	1.4	0	0	1		0	0	0	0	0	0	0	0	0	0	0	0	0	0	0	0	0	0	0	0	0	0	0	0	0	0	0.8	0	0	0	0	0	1.4	0	0	0	0.5	0.4	0.2	0	0
PHA 408	B. Kinase inhibitor	Other kinase inhibitor	3.7	4.6	6.8	4.5		2.4	0.2	3.8	0.9	4.7	6.7	1.9	2.9	5.7		3.3	4.7	4.8	4.5	3.1	0	6.3	4.8	4.6	4.2	4.8	2.5	2	2	1.9	5.3	5	3.9	0	0.2	1.9	0	2.4	0.4	3.4	0	0.8	0.2	7.9	7.3	5.4	3	8.6	0.9	0.9	1.7	6.35	2.9	4.0	2	3.3
Pictilisib	B. Kinase inhibitor	PI3K or MTOR inhibitor	21.7	7.3	4.9	14.3	6.6	7.2	6.1	10.6	8.9	6.8	19.7	7.7	15	21	11.5	13.5	11.3	8.6	15.2	5.5	6.9	8.5	8.5	8.3	7.7	9.6	11	7.4	11.8	8.9	10	8.7	6	6.6	5.9	15.4	7.8	7.4	9.9	5.9	6.9	0.8	10	12.4	6.4	8.5	10.4	20	5.5	7.7	8.45	9.8	13.85	12.5	11.2	5.7
Pilocarpine	X. Other	Other	0	0	0	0	0	0	0	0	0	0.8	2.2	0	0.2	0		0	0	0	0	0	0	0	0	0	0	0	0	0	0	0	0.1	0	0	0	0	0	1.5	0	0	0	0	0.8	0	0	0	1.3	0.8	2.9	0	0.1	0	0	0	0.0	0	0
PIM-447	B. Kinase inhibitor	Other kinase inhibitor	1.2	8.4	6.8	3.5		0.1	0.7	2.2	0.7	1.8	9	3.5	5.1	4.6		3.7	2.6	2.3	1.2	0	0	3.9	0.5	2.8	2.2	2.6	3.6	0.3	3.2	2.2	2	0.4	0.7	0	0.3	2.2	2.9	2.5	2.2	0	0	0.8	0.4	3.3	0	2.8	1.9	9.4	0.6	4.6	3.65	0.8	2.2	1.5	3.3	2
Pinometostat	C. Differentiating/epigenetic modifier	Other differentiating/epigenetic modifier	0	0	0	0		0	0	0	0	0	0	0	0	0		0	0	0	0	0	0	2.8	0	0	0	0	0	0	0.5	0	0	0	0	0	0	0	0	0	0	0	0	0.8	0	0	0	0	0	0	0	0	0	0	0	0.0	0	0
Pirfenidone	E. Immunomodulatory	Other immunomodulatory	0	0	0	0	0	0	0	0	0	0	0	0	0	0	0	0	0	0	0	0	0	0	0	0	0	0	0	0	0	0	0	0	0	0	0	0	0	0	0	0	0	0.8	0	4.3	0	0	0	0	0	0	0	0	0	0.0	0	0
Pixantrone	A. Conventional chemotherapy	Topoisomerase inhibitor	0	2.5	5.6	0	0	0	0	0	0	2.1	2.3	0	0	0.2		0.1	0	0.7	0	0	0	0	0	0	0	0	0	0	0	0	0	0	0	0	0	0	0.2	0.1	0	0	0	0.8	0	1.2	0.3	0.2	0	0.5	0	0.5	0.3	0.7	0	0.0	0	0.9
Plerixafor	E. Immunomodulatory	Other immunomodulatory	0	0	0	0	0	0	0	0	0	0	0	0	0	0	0.2	0.1	0	0	0	0	0	0.4	0	0	0	0	0	0	0	0	0	0	0	0	0	0	0	0	0	0	0	0.8	0	0	0	0	0	0.5	0	0	0	0	0	0.0	0	0
Plicamycin	A. Conventional chemotherapy	Other antineoplastic	25.5	22.1	22.1	14.4	0	0	0	0	0	12.9	26.2	1.8	9.9	6.7		5	2.8	4.5	8.2	25.2	0	1.5	7.1	3	6.6	1.2	18.4	0	0	0.6	11.9	6.2	0	0	0	4.8	21.6	0	0	0	0	0.8	0	4.2	15.6	7.2	7.3	25.1	0	14.2	9.5	9.8	10.35	4.7	9.3	15.5
Pomalidomide	E. Immunomodulatory	Other immunomodulatory	0	0	0	0.2	0	0	0	0	0	0	2.4	0	0	0		0	0	0	0	0	0	5	0	0	0	0	0	0	0	0	0	0	0	0	0	0	0	0	0	0	0	0.8	0	0	0	0	0	0	0	0	0	0	0	0.0	0.3	0
Ponatinib	B. Kinase inhibitor	Multi kinase inhibitor	4	1.5	3.4	2.4	3.5	0.6	0	2.5	0.1	4.4	9.8	2	7.6	3.6	2.3	3.3	3.1	4.7	8.9	0.4	6.1	7.9	4.1	2.7	3.8	4.7	9.2	1.5	10.3	3.6	9.3	9.6	0	1.1	0.8	0	4.8	7.1	8	1.9	7.9	0.8	5.8	4.8	5.1	3.9	5.9	9.8	6.9	8	6.8	3.95	11.2	9.6	2.1	2.1
Poziotinib	B. Kinase inhibitor	EGFR or ERBB2 inhibitor	28.4	1	0	2.1		2.9	13.6	16	13.6	1.4	5.2	11	6.6	21.1		13.7	11	17.4	0	2.2	0	0	14	9.7	5.6	11	0	10.4	0	1.8	0	0.7	8.3	0	0	0	6.8	0.6	0	7.3	0	0.8	0	13.2	1.9	3.2	4.4	5.2	1	0.8	5.6	6.55	1.1	0.7	11.3	0
Pracinostat	C. Differentiating/epigenetic modifier	HDAC inhibitor	12.5	14.4	17.3	6		6.2	5.8	8.6	10.4	12	16.7	9.6	15.3	15		10.2	8.6	9.5	4	8.3	11.9	4.3	14.5	16.9	12.6	13.9	8.5	7.2	8.6	3.6	8.1	8.1	9.8	6.7	7.2	2.2	10.3	6	6.8	6.6	8.1	0.8	6	13.4	9.8	17.9	8.2	17.2	5.1	14.7	11.1	8.75	7.3	6.4	10.7	3.8
Pravastatin	G. Metabolic modifier	HMGCR inhibitor	0	0	0	0	0	0	0	0	0	0	1.6	0	0	0		0	0	0	0	0	0	0	0	0	0	0	0	0	0	0	0	0	0	0	0	0	0.3	0	0	0	0	0.8	0	0	0	0	0	2	0	0	0	0	0	0.0	0	0
Prednisolone	E. Immunomodulatory	Glucocorticoid	0.5	0	1.3	0	0	0	0	0	0	2.5	0.9	1.3	2.3	0.4	0	0	0	0	0	0	0	0	0	0	0	0	0	0	0	0	0	0	0	0	0	0	0.4	0	0	0	0.5	0.8	0	3.5	0	3	2.3	2	0.6	0	11.85	0	0	0.0	0	0.5
Prexasertib	B. Kinase inhibitor	Other kinase inhibitor	29.4	8.3	4.1	20.1		13.5	4.7	18.9	6.6	12.9	25.8	21.8	26.5	27.8		23.7	17.6	18.8	4.1	17	5.5	0	20.4	21.1	17.6	24.3	5.4	4.4	8.9	4.9	6.5	3.9	5.3	15.9	16.1	7.8	13.3	6.7	4.8	5.9	5.2	0.8	3.6	30.4	25.5	28.8	10.5	26	8.5	27.3	26.7	22.05	8.05	2.0	18.8	27.4
PS-1145	B. Kinase inhibitor	Other kinase inhibitor	0	0	0	0		0	0	0	0.2	0.5	0.7	0	0	0.5		0.9	0	0	0.2	0	2	0	0.6	0	0	0	1.4	0	1.8	7.9	2.6	0	0	0	0	0.6	0.6	0	0.5	0.4	0	0.8	0	0	0	0	0	0.1	0	0	0	0.1	0.05	0.6	0	0
PTC-209	C. Differentiating/epigenetic modifier	Other differentiating/epigenetic modifier	12.9	6.5	4	3		1.8	3	6.9	4.9	1.8	13.4	5.6	6.3	4.6		4.2	4.3	3.8	0.6	7.7	7.5	1.8	3.3	3.3	2	2.9	8.3	2	9.4	1.6	2.9	1	4.2	3.9	5.5	2.9	7.4	4.6	4.2	3.5	7.1	0.8	3.4	2.8	3.3	5.3	2.5	12.4	3.8	3.7	4	3.15	4.3	2.5	1.6	1.6
Quisinostat	C. Differentiating/epigenetic modifier	HDAC inhibitor	12.7	13.6	15.1	6.7	2.9	5.8	2.7	7.6	9.6	11.6	15.8	5.5	16.2	14		7.9	7.4	6.5	7.1	8.4	4	3.9	12.8	10.9	10.5	11.1	7.8	5.6	10.3	4.8	8.7	8.4	6.5	5	6.4	0	8.2	4.3	9.8	4.2	6.1	0.8	6.3	12.8	9.2	15.4	8.3	13.1	2.1	16.7	11.3	9.8	9.85	3.4	7.6	7.8
Quizartinib	B. Kinase inhibitor	FLT3 inhibitor	0.5	0.3	0.8	0	1.3	0	0	0	1.4	0.9	4.3	0	0.3	0.5		0	0	0.1	1.4	0	0	0.1	0	0	0	0	0	0	0	0	0	0	0	0	0	0	2	0.1	0	0	0.2	0.8	0	0.8	0.5	2.2	0	4.3	0	2.7	0.1	0.05	0.25	0.0	0.6	0
Rabusertib	B. Kinase inhibitor	Other kinase inhibitor	0	0	0	0.5		0	0	0.7	0	0.8	0.8	0	0.6	2.4		2.1	1.7	2.1	0	0	0	0.9	1.7	1.3	0.5	0.6	0	0	0	0	0	0	0	0	0	0	0.2	0	0	0	0	0.8	0	1	0	0.1	0.8	1.7	0	4.1	3.25	0.45	0	0.0	0.5	1.3
Radotinib	B. Kinase inhibitor	BCR-ABL inhibitor	0	0	0	0		0	3.2	0	0	0	4.8	0	0.6	0		0	0	4.7	0.3	0	1.7	15.3	1.4	0	3.9	0.4	0	0	0	0	8.4	0	0	0	0	0	5.1	0	0	0	0	0.8	0	0	3.3	0	1	4.3	2.4	0	0	0	1.3	0.1	1.9	0
Ralimetinib	B. Kinase inhibitor	p38MAPK inhibitor	7.2	0	0.4	0.8		1.2	1.1	2.3	3.2	3.6	5.4	3.3	3.4	9		1.2	1.7	0	0	1.3	0	0	1.9	2.1	2.6	2.4	0.5	2.2	0	3	0	0	2.7	0	0.7	0	3.3	0	0	1.3	0	0.8	0	4.1	0.9	3.8	9.3	6.3	0.9	4.9	3.15	3.9	0	0.0	1	3
Raloxifene	F. Hormone therapy	Antiestrogen	1	1.9	1.5	1.1	0	0.5	0	2.2	0.9	2.8	5.1	2.3	4.5	3.7	1.9	3	1.4	2.9	0	2.2	0	0.3	1.6	1.3	0.4	1	1.5	1	0.1	0.3	0	1	1.1	0	0	2.7	5	0.1	0.3	0	1.9	0.8	0.6	18.7	0	3.2	3.2	4.5	0	4.2	1.55	2.4	0.15	0.9	2.1	2.1
Raltitrexed	A. Conventional chemotherapy	Antimetabolite	9.5	0	0	0	0	0	2.6	1.2	2.1	3.1	14.6	6.1	1.4	0.9		0	0	0	0	0	0	0	0	0	0	0	0	1.1	0	0	0	0	6	2.5	4.5	0.1	4.4	0.8	0	3.1	0.7	0.8	0	3.8	0	15.8	0	15	1.4	3.4	6.5	0	0	0.0	0	0
Ravoxertinib	B. Kinase inhibitor	Other kinase inhibitor	12.9	1.8	3.3	0.8		0.2	1.3	3.9	3.2	0	10.6	0	7.8	9.2		0	1.4	0	0	0	0	1.3	7.2	0.8	0	9.8	0.4	0	0	0	2.5	0	1.7	5.3	5.4	0	13.8	2.2	0	1.1	0.5	0.8	0	4.6	0	5.9	6.1	12.2	1.5	10.1	2.5	1.8	0.85	0.0	0	1.4
Regorafenib	B. Kinase inhibitor	Multi kinase inhibitor	3.5	1.1	1.4	1.7	6.3	0.1	0.4	1.4	1	2.3	10.1	6.8	1.3	3.9		2.4	1.8	3.3	2.8	2.3	1.2	3.3	5	5.8	5.9	2	3	0	4.7	0	4.1	3.8	1	4	4.2	4.2	4	2.2	1.3	1.2	1.9	0.8	2.5	3.3	1	4.7	1.3	9.8	4.1	4.3	2.9	3.3	4.1	2.5	1.9	4.4
Resatorvid	E. Immunomodulatory	TLRs ligand	0	0	0	0		0	0	0	0	0	0	0.4	0	0		0	0	0	0	0	0	0.3	0	0	0	0	0	0	0	0	0	0	0	0	0	0	0	0	0	0	0	0.8	0	0	1.8	0	0	0	0	0	0	0	0	0.0	0	0
Resiquimod	E. Immunomodulatory	TLRs ligand	0	4.3	0	0		0.8	0	0	0	0.1	2.7	0.6	0	0		0.2	0	0	0	0	0	0	1	0.7	0	0	0	0	0	0	0	0	0	0	0	0	0	0	0	0	0	0.8	0	0	0	1.1	1.3	1.6	0	0.5	0	0	0	0.0	0	1.5
Resminostat	C. Differentiating/epigenetic modifier	HDAC inhibitor	7.4	8.9	9	5		5.1	3	4	6.7	9.7	11.5	12.6	10.6	10.4		7.5	6	4.7	3.3	5.8	3.7	6.2	8.8	11.9	9.4	9.8	6.4	3.6	6.7	4.7	3.9	7.9	4.9	3	3.1	4.8	3.9	2.7	4.2	3.1	4.8	0.8	3.2	8.1	3.9	10.9	5.5	12.4	0.3	10.3	7.85	6.9	4.6	3.0	7.2	3.3
RGFP966	C. Differentiating/epigenetic modifier	HDAC inhibitor	0.2	0.3	0	0		0	0.3	1.4	1.4	0.3	7	1.5	6.2	1.2		3.8	2.6	4	4.4	3.5	4.9	0.1	2.6	1.9	1.9	2.8	2.3	1.8	1.3	8.4	1	3	4.2	0.4	0.2	1.7	2.7	1.2	0	3.5	1.5	0.8	0	1.1	2	1.3	0	9.6	2.4	0.1	1.1	2.6	4.55	3.1	4	0
Ribociclib	B. Kinase inhibitor	CDK inhibitor	0	3	1.7	0	0	0	0	0	0	0	8.4	0	0	0		0	0	0	0	0	3.4	0	0	0	0	0	2.3	1.4	1.2	1.5	1.5	0	0	0.2	0	0.9	1.3	3.9	0	1.3	1.1	0.8	0	0	0	0	0	10.6	6.3	3	0	0	2.7	0.4	0	0
Ridaforolimus	E. Immunomodulatory	Rapalog	7.5	4.3	3.1	6.3		2.2	6.8	7.2	8.3	2.4	16.3	2.4	9.1	14.8	10.7	1.4	1.7	5.3	8.4	4.3	1.1	5.3	13.4	13.5	13.8	13.3	9.3	1.4	6.3	0.6	9.4	4.3	1.8	4	4	6.3	5.3	4.8	8.4	3	2.6	0.8	2.6	11.7	7	8.3	2.6	16.4	0.4	13	7.6	6.8	8.05	2.6	3.3	2
Rigosertib	X. Other	Other	24.9	11.2	11.9	8.1		2.4	9.1	10.8	9.3	12.4	29.8	32.4	20.9	21.4	10.6	16.7	11.5	9.3	23.7	21.7	20.3	23.6	9.2	8.9	9.1	9.5	19.4	13.7	23.4	15.5	23.6	16.3	16	2.1	0	10.9	11.4	24.9	28.5	15	19.2	0.8	22.6	28.1	14.5	25.9	11.7	30.3		22.4	18.8	21.65	27.8	26.7	8.3	11.3
Ripasudil	B. Kinase inhibitor	Other kinase inhibitor	0.9	0	0	0	0	0	0	0	0	0	2.1	0	0	0		0	0	0	0	0	0	0	0	0	0	0	0	0	0	0	0	0	0	0	0	0	0	0.3	0	0	0	0.8	0	0	0	0	0	0	0	0	0	0	0	0.0	0	0
RO5126766	B. Kinase inhibitor	Other kinase inhibitor	10.4	2.5	0	1.4		0	0	1.4	0.4	2.5	8.7	2	0	2		0	0	0	0	0	0	0	2	0	0	0	1.3	0	0	0	0	0	0	3.4	2.8	0	6.2	1.5	0.2	0	0	0.8	0	0.9	0	0.6	5	6.9	0	0.2	0.6	0.25	0.2	0.0	0	0.2
Rociletinib	B. Kinase inhibitor	EGFR or ERBB2 inhibitor	10.4	1.4	2.5	7	4.3	5.6	6.2	7.6	8.4	4.3	9.5	6.9	7.6	9.6		9	6.7	8.1	7.2	3.8	3.6	4.9	5.7	2.1	3.2	5.8	7.1	6.8	6.1	8.2	6.8	7.1	6.7	3.6	4.8	8	7.4	5.5	6.4	6.3	4.7	0.8	5.6	8.3	2.6	7.2	5.4	11	1.3	4.5	6.6	6.85	7.05	7.4	8.7	5.9
Rocilinostat	C. Differentiating/epigenetic modifier	HDAC inhibitor	5.1	4.9	4.7	0.9		0.5	1.6	2.5	6	6.4	8.4	2.3	6.9	7.4		4.9	5.2	0.7	0	3.8	4	0	6.5	7.4	6.4	6.4	1.6	1.1	0.5	0	2.2	3.1	3.7	0.3	0.4	1.9	5	0.5	1.4	1.9	1.5	0.8	0.8	5.3	2.1	8.2	3.2	8.3	1.9	6.1	5.9	4	1.5	0.5	4.5	0
Romidepsin	C. Differentiating/epigenetic modifier	HDAC inhibitor	27.8	28.9	28.8	17.2	23.8	19.9	19.5	26.9	28.4	21.2	29.2	0	26.8	26.5		24.5	20.9	17.8	13.3	22.8	23.5	11.6	25	25.2	21.4	27.3	22.7	22	23.7	26.6	17.9	21.1	21.1	16.7	18.8	22.5	26.4	16.3	22.5	20.3	18.3	0.8	20	40.4	24	25.6	16.9	28.6	17	29.1	26.3	25.1	20.1	19.1	23.6	14.4
Roxadustat	C. Differentiating/epigenetic modifier	Other differentiating/epigenetic modifier	0	1.3	2.2	0		0	0	2.3	0.1	0	0	0	0	0.9		0.3	0.5	0	0	0	0.8	1.7	0	0	0	0	0	0	0	0	0	0	0.2	0	0	0	0	0	0.2	0.9	0	0.8	0	0	0	0	0.2	1.8	0	0	0.3	0	0.6	0.4	0.2	0
RSL3	G. Metabolic modifier	Other metabolic modifier	1.7	0	0	0		0	0	0	0	0	0	0	0	0		0	0	0	0	0	0	0	0	0	0	0	0	0	0	0	0	0	0	0	0	0	0	0	0	0	0	0.8	0	0	0	0	0	0		0	0	0	0	0.1	0	0
Ruboxistaurin	B. Kinase inhibitor	Other kinase inhibitor	8.3	8.4	7	5.5	0.3	1.8	1.3	6.2	3.6	7.7	9.4	8.1	8	8.2		6.2	6.4	8.3	5.6	2.4	0.7	6.9	5	5.7	6.2	7	2.1	8.1	2.8	7.4	3.3	7.5	9	3	3.8	7.3	4.1	3.8	4.7	6.6	4	0.8	0	6.7	6	7.6	3.3	9.7	3.8	7.4	8.45	6.25	4.8	6.2	3.4	3.8
Rucaparib	C. Differentiating/epigenetic modifier	PARP inhibitor	0	0	0.1	0	0	0	0	0	0	0	4.8	0	0.5	0.7		0	0	0	0	0	0	0.4	0	0	0	0	0.5	0	1.3	0	0	0	0	0	0	0	0.3	0	0.4	0	0	0.8	0	0	0	2.2	0.1	5.2	0.1	2.3	0.95	0	0	0.0	0	0
Ruxolitinib	B. Kinase inhibitor	JAK inhibitor	0.7	9.4	7.4	0	7.5	0	1.9	0	3.3	0	4.2	0	0	0		0	0	0	0	1.4	0	0	2	0.9	0	0	0.9	1.1	6.9	2.3	0	1.5	0	0	0	0	0	0	0	0.9	0	0.8	4.3	0	0	1.1	0	0	0.4	0	0.95	0	0	0.0	0	0
S-63845	D. Apoptotic modulator	BH3 mimetic	1.8	8.5	9.7	0		0	0.2	0.3	1.5	2.1	10.8	3.3	1.4	0		0	0	0	0	4.5	0	0	0	3.2	0	0	0	0	0	0	0	0	0	0	0	0	0	0	0	0	0	0.8	0	0.9	0	0	1.6	6		0.2	2.05	2.25	0	0.0	0	0
Sabutoclax	D. Apoptotic modulator	BH3 mimetic	27	13.2	13.8	12		7.7	8.3	9.6	8.9	11.6	19.7	8.9	13	13.7		9.9	10.3	7.7	6.5	17.6	6.7	6.7	7.1	8.3	7.9	6	14.5	6.9	15.8	11.5	13.7	6.2	8.4	6.1	7.1	6.6	6.1	15.2	11.1	12.4	14.3	0.8	14.4	6.4	9.4	13.5	6.9	17.1		15.7	11.9	4.95	10.25	8.7	5.8	7
Salinomycin	X. Other	Other	20.2	17.6	30	16.1	12.5	12.8	10.1	17.1	15.3	19.8	30.8	6.5	15.6	24.9		17.6	8.6	18.5	19.8	16.2	14.5	14.2	12.6	10.8	15.9	14	21.3	24.1	17.8	24.4	18.2	15.6	21.3	2.1	3.3	12.5	7.3	15.9	17.1	23.6	20.9	0.8	15.4	25.1	10.6	8.2	11.9	28.2	18.5	12	7.4	20.65	23	19.1	22.8	8
Sapanisertib	B. Kinase inhibitor	PI3K or MTOR inhibitor	19.6	7.6	4.4	15		13	13	15.6	13.7	11.9	19.6	12.6	12.6	21.6		11.8	10.1	12.4	15.6	10	7.1	12.3	13.7	13.1	14.4	10.1	15.1	11.2	16.4	14.9	14.8	13.9	14.1	8.5	6.8	16.6	14.1	12.2	16.1	10.9	10.6	0.8	15.7	16.7	13	12.8	8.8	22.7	4.4	15.8	11.55	12.4	14.65	15.0	14	9.9
Sapitinib	B. Kinase inhibitor	EGFR or ERBB2 inhibitor	15.4	0	0	0.9		1.8	6.8	6.5	8.7	0.1	0	1.8	2.5	12.6		6.7	6.9	9.2	0	2.3	0	0	4.7	5.5	0.9	4.4	0	4.5	0	3.4	0	0	4.5	0	0	0	2.7	0	0	2.2	0	0.8	0	3.4	0	0.2	1.1	0	0.4	0.1	3.15	2.2	0	0.0	5.5	0
SAR405838	D. Apoptotic modulator	p53 pathway modulator	2.6	8.6	11.3	1.1		0	5.2	0.9	4.1	1.2	15.8	2.1	4.3	0.6		1.8	2.1	2.2	3	3.4	0	6.2	2	1.4	1.5	0.5	6.8	4.1	7	6.1	5.3	5.4	6.8	0.5	0	3.6	2.2	4.9	4.5	5.1	1.8	0.8	1.4	4	0	1.9	0.9	14.9		3	2.35	5	5.55	4.7	1.1	0
Saracatinib	B. Kinase inhibitor	Other kinase inhibitor	17.5	0	0	7.8	7.2	3.4	5.3	9.3	10.9	4.2	0.6	5.4	13.2	17.4		7.1	6.3	6.3	0.3	4.9	1.3	3.3	3.2	4.3	3.4	4.1	4.9	5.7	10.3	6.5	4.5	4.9	4.8	2.6	2.3	0	8.5	2.7	7.3	3.7	3.8	0.8	5.3	10.8	5	7.5	10.7	1.1	1.7	7.4	11.6	4.4	6.3	4.6	4.5	5.9
Saridegib	X. Other	Hedgehog inhibitor	0	1.3	1.9	0.5		0	1.7	3.4	2	0	2.7	0	0	1.8		0	0	0	0	0	0	4.9	0	0.6	0.9	0	2.4	0	5	0	2	0.7	0.5	0	0	0	0	0.1	0	0	1.1	0.8	0.7	0	0	0	0	5.2		0.1	0	1	2.1	0.9	0	0
SB 743921	X. Other	Kinesin inhibitor	15.7	6.8	0	0		0	0	4.9	0	1.6	16.1	0.1	2.6	3.1		1.9	0	0.3	0	3.4	1.8	0	0.4	1.8	0.7	1.5	0.7	1	9	0	2.9	0	0.3	0	0	0	0.8	0	0	2.4	1.7	0.8	0	14.8	1.1	3.3	0	15.4		4.3	4.35	7.7	0.65	0.0	0	2.5
SCH772984	B. Kinase inhibitor	Other kinase inhibitor	20.4	3.7	5.7	8.1		3.1	8.9	11	8.7	6.4	19.9	2.7	12.2	15.3		4.9	4.7	4.6	4	6.3	7.1	3.6	9	3.7	8	0	11.5	6.4	10	2.4	14.7	12.5	7.8	9.6	8.6	7.7	20.2	15.1	9.7	6.4	13.4	0.8	11	7.1	4.1	9.2	10.9	20.2	2	20.8	11.4	6.55	12	10.4	3.3	6.3
Seliciclib	B. Kinase inhibitor	CDK inhibitor	1.5	0	0.8	1		0	0	0	0.4	0	4.1	1.9	2.5	4.1		0.6	0.6	3.2	0.7	2	0	10	1.2	1.4	1.3	0.1	1.6	0	4.1	0.5	3.1	3.3	0.6	0	0.5	0	1.2	1.5	2.6	0.2	0	0.8	0.3	2.8	0	2.5	1.6	4.6	0.3	3.1	0.5	0.3	1.55	2.2	1.6	1.3
Selinexor	D. Apoptotic modulator	Other apoptotic modulator	23	16.2	17.9	6.3		10.7	12.4	13.7	13	14.8	25.1	20.8	15.6	21.7	19	15.3	15.5	20.5	4.3	26.9	0	10.4	13.7	13	11.9	13.7	10.4	1.7	10.6	14.3	9	5.4	7.6	10.3	12.5	2.4	16.9	6.6	5	12.7	9	0.8	4	17.9	14.8	20.5	5.1	24	4.6	21.2	18	22.05	8.9	4.7	15.1	10.2
Selonsertib	B. Kinase inhibitor	Other kinase inhibitor	0	0	0	0		0	0	0	0	0	0	0	0	0	0	0	0	0.2	0	0	0	0	0	0	0	0	0	0	0	0	0	0	0	0	0	0	0	0	0	0	0	0.8	0	0	0	0	0	2.4	0	0	0	0	0	0.0	0	0
Selumetinib	B. Kinase inhibitor	MEK1/2 inhibitor	19.1	5.2	3.5	2.8	3.2	0	4.8	8.5	5.6	3.5	8.9	3.8	6.6	7.2	3.2	1.8	2.9	5	2	2.2	3.4	1.4	5.3	2.9	4	5.8	4.4	6.1	4.8	2.2	5.9	4.8	9.3	10	8.2	3	11.1	6.8	2.4	4.8	7.2	0.8	4.5	5	1.6	3.5	4.2	9.5	2.1	15.5	6.45	4.1	6.55	5.1	0.5	0.1
Senexin B	B. Kinase inhibitor	CDK inhibitor	1.7	0	0	0		3	1	0.7	2.6	0	3.5	0	0	1		2	2.5	0	0.1	0.1	0	0	0.5	1.3	0	1.2	0	0	0	0	0	0	0	0	0	0	2.6	0	0	0	0.2	0.8	0	0	0.1	1.6	4	2.7	0	1.8	1.1	0	0	0.0	1.3	0
Sepantronium bromide	D. Apoptotic modulator	Other apoptotic modulator	26.2	24.3	25.1	35.8		30.4	26.8	30.1	29.1	42.9	40.6	3.3	40.2	42		41.8	39.8	24.3	18.8	40.2	30.7	27	36.9	36.3	37.8	29.3	30.1	30.2	32.2	28.8	27.1	24.7	27.4	35	35.2	32.1	30.5	29.2	28	28.3	29.6	0.8	30	42.7	42.4	38	37.7	38.4	29.7	40.3	31.8	38.4	32.1	27.1	38.2	29.7
Serabelisib	B. Kinase inhibitor	PI3K or MTOR inhibitor	8.8	0.1	0	4.9		1.6	3	2.3	2.9	0	7.3	0	1.9	4.3		5.5	4.6	0.5	4.2	0	0	4.4	0.5	0.3	2.4	0.5	3.5	1.6	5.3	0.3	3.4	2.7	0.2	1.4	0	1	3.4	1.8	1.5	0	0.3	0.8	2.1	3.4	0	0	0.6	8	0	1.5	1.7	1.4	3.35	3.8	5.9	1
SGC-CBP30	C. Differentiating/epigenetic modifier	Other differentiating/epigenetic modifier	5.6	4.3	4.6	3		0.6	5.3	2.7	4.7	4.5	7.8	9	7.3	10.2		1.9	2.1	3.5	0	4	0.3	1.1	1.5	4.1	2.4	0.8	0.7	3.4	0.3	5.1	1.4	0.3	2.2	2.5	0.5	1.4	2	0.5	0.9	2.2	0.6	0.8	0.6	7.1	2.3	8.3	5.8	9.2	0.3	5.8	4.85	6.55	1.55	1.9	2	0.9
SGC0946	C. Differentiating/epigenetic modifier	HMT inhibitor	0	1	3.4	0		0	0.1	0	0	0.1	3.3	0	0	0		0.2	0.5	0	1.7	0	0.6	0.4	0.2	0.8	1.7	0.3	0	0	0	0	1.4	0	0	0	0	0	2.5	0.1	0	0	0	0.8	0	0.8	1.7	0	0.1	3.1	0	1.3	1.5	0.85	1.2	0.0	0.2	0
SGI-1776	B. Kinase inhibitor	Other kinase inhibitor	6.5	8.9	7.1	7		5.1	5.9	6.5	4.9	7.2	9.7	7.8	8.8	9.2		8.3	6.3	5.2	9.3	1.6	4.7	8.6	6.3	5.6	5.6	5.3	7.9	5.9	8.6	6.7	7.6	7.2	6.5	2.7	0.2	7.9	7.7	7.7	6.3	4.8	6.2	0.8	6.1	7.2	5.8	5.7	5.9	10.1	0.9	9.6	8.95	5.8	7.85	6.6	7.8	6.5
SH-4-54	X. Other	Other	5.9	11.8	15	3.7		5.9	6.8	8.4	6.3	5.9	6.7	8.4	3.5	14.6		8.5	7.6	10.4	9	6.9	5.6	7.4	7.5	10	7.9	9	8.5	5	7.3	5.2	7.2	5.8	4.9	5.5	5.9	5.6	8.3	5.6	6.3	5.5	5.7	0.8	6.2	9.9	4.5	6.4	6.3	4.6		5.7	8.35	8.5	7.7	8.2	8.3	3.5
Silmitasertib	B. Kinase inhibitor	Other kinase inhibitor	9.9	6.4	4.4	4.9		2.9	6.5	4.7	8.7	5.4	8.7	7.2	7.1	14.2	4.8	7	7.8	5	1.8	8.2	1.6	6	5.3	5	5.8	2.7	1.8	6	2.4	7.3	4	3.1	5.4	3	2.9	1.5	4.9	1.1	4.8	6	2.3	0.8	2.1	8.1	2.7	6	6.2	9.6	2.3	5	8.2	8.05	2.15	1.3	6.5	3
Simvastatin	G. Metabolic modifier	HMGCR inhibitor	0	1	1.3	0	1.6	0.4	0.5	2.1	2.1	2.3	2.5	1	0	0		0	0	0.8	0	0	1.3	0	0	0.1	1	0.2	0.9	4.9	0.3	2.5	0.4	0.9	2.5	0.3	0.8	0.9	2.4	2	0.2	4.3	1.4	0.8	0.2	0	0	2.1	0	1.9	0.3	0	0	2	1.05	0.5	0	0
Sirolimus	E. Immunomodulatory	Rapalog	5.4	2.7	1.8	4.1	0	2.6	3.9	4	5.2	2.4	9.8	2.4	4.1	7.5	7.7	1.6	1	4.4	2.6	3.2	5.9	0.4	8.4	5.4	9	4.3	4.6	3.7	5.6	2.6	3.6	1	4.7	1.7	1.4	0	2.6	2.3	4.4	4.8	0.7	0.8	2.3	0	4.3	3.7	1.5	11.3	0.2	8.4	3.35	4.3	2.2	1.2	1.9	0.4
Sitravatinib	B. Kinase inhibitor	Multi kinase inhibitor	0	0.5	0	0.9		0	0	1.1	1.6	0	4.8	0	3.8	0.5		1.4	1.8	0.4	5.5	1.9	4.7	4.9	1.6	1.5	0.2	2.6	5.6	2.1	4.8	3.3	5.8	4.8	2.3	0	0	5.3	1.6	4.9	2.6	1.2	3.9	0.8	2	1.7	0.3	0	1.9	6.6	3.9	0.4	3	1.75	5.25	4.6	1.7	0
SN-38	A. Conventional chemotherapy	Topoisomerase inhibitor	31.2	18.7	14.4	14	19.9	7	15.4	16.3	17.4	14.8	40	29.4	17.3	25.3		26.8	19.8	14	9.2	27.2	10.4	9.6	17.9	16.4	16.5	21.3	13.2	19.1	12.9	15.9	10.5	9.5	21.7	18	22.2	17.2	21.6	11.6	9.7	17.6	8.6	0.8	6.2	28.6	20.3	33.2	10.7	39.8	13.7	36.7	37.05	26.95	11.8	10.9	20.4	17.3
SNS-032	B. Kinase inhibitor	CDK inhibitor	19	23.4	20.4	14.7		13.8	21.5	15.8	24.2	17.6	29.4	7.6	20.1	19.4		16.3	11.1	20.3	10.4	19.8	5	15.4	12.4	13.3	13.1	9.9	21.6	10.9	25.9	18.2	19.6	18.2	18.2	13.1	15.7	12.8	19.7	14.2	19.5	14.3	16.9	0.8	15.4	22.6	16.5	19.9	11.1	23.8	4.5	27.1	21.8	24.75	17.35	16.5	18.3	22.3
Sonidegib	X. Other	Hedgehog inhibitor	0.1	0.3	0	0.8	4.5	0	0	0.9	0.6	0	8.8	0	0	0.8		0	0	0.3	0	0	0	0	0	0	5.1	0	0	0	0	0	0	0	0	0	0	0	3.7	0	0	0.4	0	0.8	0.1	2.1	0.8	2	2.1	10.2	0	0	0.15	0	0	0.0	0.4	0.1
Sonolisib	B. Kinase inhibitor	PI3K or MTOR inhibitor	0	0.2	1.7	0.8		0	0	0	0	1.1	0.1	0	0.1	1.4		0	0	0.7	0	0.2	0	2	0	0	0	0	0	0	0.7	0	0	0	0	0	0.2	0	0.2	0	0	0	0	0.8	0	0	0.2	0	0	2.4	0	0.8	0.1	0.55	0	0.2	0	1.6
Sorafenib	B. Kinase inhibitor	Multi kinase inhibitor	0	0	0	0	0	0	0	0	0	0	0.1	0	0	0	1.2	0	0	0	0	0	1.4	0.3	0	0	0	0.1	0	0	0	1.1	0	0	0	0	0	0	0.1	0	0	0	0	0.8	0	0	0	0	0	0.1	0.8	0	0	0	0.5	0.0	0	0
Sotrastaurin	B. Kinase inhibitor	Other kinase inhibitor	0.6	4.6	3.9	0		0	0	0	0	0	0.1	0	3.1	0		0	0	0	0	0	0	0	0	0	0	0	0	0	0	0	0	0	0	0	0	0	0	0	0	0	0	0.8	0	2	0	0.3	0	0.3	0.4	0	2	0.05	0	0.0	0	0
Spebrutinib	B. Kinase inhibitor	BTK inhibitor	0.3	0	0	0.2		0	0	0	0	0.7	2	0	1.3	1.9		1.8	0	1	1.9	1.3	1.2	2.5	1.9	0	0.6	0.4	2.2	0	2.3	0.6	2.5	2.7	0.7	0	0	3.4	0.6	3.2	2.3	0	1.5	0.8	1.5	0.8	0.6	0.1	0	5.8	0	2.9	0.6	0.65	3.15	1.6	0.7	0
StemRegenin 1	C. Differentiating/epigenetic modifier	Other differentiating/epigenetic modifier	1.9	0	0	0		1.3	3.9	3.8	2.9	2.4	4.7	4.3	1.7	0.2		3.4	1.5	1.9	0.5	1.7	0	4.7	0.3	2	3	1.8	0.1	2.5	1.4	3	0.5	1.3	5	0	0	2.1	5.7	0.9	0.5	3.6	0.8	0.8	0	1.6	0.8	1.5	1.2	6	2.1	3.7	1.7	6.95	1.3	2.3	2.3	0.6
Sunitinib	B. Kinase inhibitor	Multi kinase inhibitor	0	1.9	1.9	0	0.1	0	0	0	0	0	3.6	1.2	2.7	0	0	0	0	0	6.1	0	0	0	0	0	0.9	0	1.9	0.1	0.5	1.2	3.1	1.4	0.4	0	0	0	1.6	1.2	0	0	0	0.8	0	0	0	0	0	4.3	0	2.2	1.55	0	2.1	0.1	0.8	0.3
Tacedinaline	C. Differentiating/epigenetic modifier	HDAC inhibitor	0	0.1	0	0		0.2	0	0.1	0.1	0	0	0	0	0		0.2	0.3	0	1.7	0.8	2.9	0	0.8	3	0.2	1.5	0.1	0	0	0.8	0.5	0.5	0	0	0	0.6	0	0.6	0	1.2	0.7	0.8	0	1	0	0.5	0	0.9	0	0	0.7	0.45	0	0.8	1.3	0
Tacrolimus	E. Immunomodulatory	Other	0	1.7	1.7	0	0.8	0	0	0.2	0	0	2.3	0.7	0	2.2	0	0.2	0	0.2	0	0	2.4	0	1.5	0	0	1.2	0	0	0	0	0	0	0	0	0	0	0.9	0	0	0	0	0.8	0	0	0.7	1	1.5	3.8	0	0.8	0	2.45	0.3	0.0	0	0
TAK-285	B. Kinase inhibitor	EGFR or ERBB2 inhibitor	8.5	0	0	0		0	1	0	2.7	0	0	0.4	1.6	3.1		1.1	0.3	0.4	0	0.5	0	0	1.6	0.1	0	0.6	0	2.4	1	2.6	0	0	3.3	0	0	0	2.9	0	0	2.7	0	0.8	0	1.6	0	0.4	0.3	2	1.5	0	1.95	1.7	0.15	0.0	0	0
TAK-530	B. Kinase inhibitor	RAF inhibitor	0	0	0	0		0	0	0	0	0	0.7	1.2	0	2.1		0.7	0	0.4	0	0.2	0	3	1.6	0.3	1.3	3.5	0	0	1.1	0	1.6	0.5	0	0	0.8	1.7	5.2	0.8	2.9	0	0.1	0.8	0.7	0	0.1	0.9	0	2.9		0.6	0.85	0.6	1.5	2.7	0	0.1
TAK-901	B. Kinase inhibitor	Aurora kinase inhibitors	6.7	6.5	4.6	2.3		0.7	0.9	3.1	4.4	4.1	12.1	0	7.9	8.6		2.4	0.9	1.4	7.8	3.8	5.3	3	3.1	0.2	2.7	0	8.8	4.3	9.2	6.4	9.6	9.1	3	1.2	0.2	10	3	9.2	7.6	2	7	0.8	5.4	8.3	5.8	1.7	4.4	11.8	0.8	6.3	12.8	3.35	9	7.9	2.6	3.3
Taladegib	X. Other	Hedgehog inhibitor	0.8	0	0	1.2		0	0	0	0.5	0.6	3.2	0.8	0	0.3		0	0	0	0	0	0.6	0	0	0	0	0.6	0	0	0	0	0	0	0	0	0	0	0	0	0	0	0	0.8	0	0.4	0	0.6	0	3.6		0	1.25	0.6	0	0.0	0	0
Talazoparib	C. Differentiating/epigenetic modifier	PARP inhibitor	1.8	2	1.1	1.3		0.5	0.1	2.1	2.9	1.5	20.7	0	2.7	0.6		3.9	3.1	0	0.2	8.3	0	0	4.9	0	0	5.3	3	0.7	2.6	0.8	0.5	0.4	0	0.6	0	0	9.6	2.5	4.2	1.2	0.2	0.8	0	4.1	0.4	5.5	2.9	21.3	1.4	12.3	5.75	5.7	4.6	0.9	0	3.1
Talmapimod	B. Kinase inhibitor	p38MAPK inhibitor	0	0	0	0		0	0	0	0	0	3.5	1.9	0	0.8		0	0	0	0	0	0	0.5	0	0	0	0	0	0	0	0	0	0	0	0	0	0	0	0	0	0	0	0.8	0	0	0	0	0	1.4	0	2.2	0	0.2	0	0.0	0	0
Tamatinib	B. Kinase inhibitor	Other kinase inhibitor	8	3.6	2.4	5.7		4.2	2.8	1.4	7.6	2.4	9	2	2.2	13.1		5	3.1	1.5	14.7	2.3	7.3	7.4	0	0	0.8	0.6	3.7	3.2	12.1	7.1	10.9	13	1.2	0	0	16.2	2.4	1.5	5	3.4	0	0.8	1.9	1	0	3.2	5.2	9.4	0	0	1.6	2.05	12	11.8	2.2	0.3
Tamoxifen	F. Hormone therapy	Antiestrogen	1.8	3.9	3.7	0.5	3.7	0.3	1	3.3	2	1.9	5.3	2.1	3	2.9	1.9	2.8	1.6	4.9	0.7	3.1	0	0.8	4.8	3.8	6.2	3.7	0	1.8	0.9	0.6	0.4	0.4	1.5	0.1	0.4	1.2	6.7	0	3.4	0.5	0.3	0.8	0.4	2.5	1.1	2.8	1.3	6.1	0.2	4	2	3.1	0.25	1.7	3.6	0
Tandutinib	B. Kinase inhibitor	FLT3 inhibitor	0	0	0	0		0	0	0	0	0	0	0	0	0		0	0	0	0	0	0	1.4	0	0	0	0	0	0	0	0	0	0	0	0	0	0	0	0	0	0	0	0.8	0	0	0	0	0	0	0	0	0	0	0	0.0	0	0
Tanzisertib	B. Kinase inhibitor	Other kinase inhibitor	2.7	0	0	0		0	0	0.2	2.3	0	3.1	0.3	1.7	3		0	0	0	0	0.1	1.1	2.2	0	0	0	0	0	0.5	0	0	0.4	0.1	0.6	0	0.3	0.6	1	0.4	0	0.2	0	0.8	0	1.3	0	0.1	2.1	3.1	0	1.1	0	0	0.15	0.0	0	0
Tarenflurbil	X. Other	Other	0	0	0	0		0	0	0	0	0	0.8	0	1	0		0	0	0	0	0	0	0	0	0	0	0	0	0	0	0	0	0	0	0	0	0	6.1	0	0.4	0	0	0.8	0	0	0	0	7.3	4.5		2.8	1.1	0	0	0.0	0	0
Taselisib	B. Kinase inhibitor	PI3K or MTOR inhibitor	20.4	5.2	4	7	1.5	3.6	6.4	9.2	5.9	1.3	12.4	2.1	6.5	9.5		10.9	7.3	5.5	5.8	2.7	6.3	6.4	5.4	3.2	2.3	5.5	8.9	6.5	7.2	10	6.7	6.7	1.3	4.2	3.5	8.7	7.7	7.2	5.2	3.7	3.9	0.8	6.1	4.1	0.3	2.5	3.4	16.5	5.2	0	2.7	6.05	9.4	8.1	10.2	1.8
Tasquinimod	E. Immunomodulatory	Other immunomodulatory	0	0	0	0.1		1.5	0.8	0	0	0.8	0.6	0	0	4		3	0.7	0	4.8	1.7	0	0	1	0.7	0	0.2	1.2	0	0	0.4	0	0.2	0	0.1	0	0	0	0	2.1	0	0	0.8	0.2	0.7	0	1.3	1.6	6.4	0	3.3	0	0.05	0.2	0.0	0	0.5
Tazemetostat	C. Differentiating/epigenetic modifier	HMT inhibitor	0	0	0.1	0		0	0	0	0	0	0	0	0.2	0		0	0	0	0	0	0	0	0	0	0	0	0	0	0	0.3	0	0	0	0	0	0	0	0.8	0	0	0	0.8	0	0.3	0	0	0.1	0	0	0	0	0.2	0	0.0	0	0
Telatinib	B. Kinase inhibitor	Multi kinase inhibitor	0	0	0	0		0	0	0	0	0	0	0	0	0		0	0	0	0	0	1.3	0.8	0	0	0	0	1.1	0	1.2	0	0.2	0	0	0	0	0	0	1.5	0.2	0	0	0.8	0	0	0.6	0	0	0	0	0	0	0	1.95	0.7	0	0
Temozolomide	A. Conventional chemotherapy	Alkylating agent	0	0	0.2	0	0	0	0	0	0	0	0	0	0	0		0	0	0	0	0	0	0	0	0	0	0	0	0	0	0	0	0	0	0	0	0	0	0	0	0	0	0.8	0	0	0	0	0	0	0	5.3	0	0	0.2	0.0	0	0
Temsirolimus	E. Immunomodulatory	Rapalog	8.1	5.5	3.9	6	0	1.2	5.7	5.9	8.3	2.1	15.4	2.7	7.6	12.8	10	1.8	1	7.9	4.2	4.6	0	4.2	14.3	14.9	15.3	10.2	4.9	0.6	7.6	0.2	4.5	2.3	3.1	2.7	1.2	0.9	2.9	2.8	6.6	4.4	2.2	0.8	3.3	5	7.9	7.8	0	16.4	0	13.1	5.4	7.6	6.3	2.0	3.4	1.5
Teniposide	A. Conventional chemotherapy	Topoisomerase inhibitor	0	0	0	0	0	0	0	0	0	0	0	0	0	0		0	0	0.2	0	0	0	0.9	0	0	0	0	0	0	0	0.9	0	0	0	0	0	0	0	0	0	0	0	0.8	0	0	0	0	0	0	0	0	0	0	0	0.0	0	0
Tepotinib	B. Kinase inhibitor	MET inhibitor	0	0	0	0		0	1.5	0	1.1	0	0	0	0	0		0.4	0	0	0	0	0	0	0.1	1.6	1.1	0	0	0	0	0	0	0	0	0	0	0	3.4	0	0	0	0	0.8	0	0	0	0	0	0.1	0	0	0	0	0	0.0	0	0.8

Tesevatinib	B. Kinase inhibitor	EGFR or ERBB2 inhibitor	19.8	1.6	1.5	1.6		3.3	8	9.6	10.8	0.7	1.5	1.7	5.1	17		7.6	6.7	12.8	0.9	1.4	0	1.3	8.7	7	4.5	6.9	0	9.8	0	5.9	0.6	0	9	0	0	0	3.6	0	0	3.7	0	0.8	0	6.3	0	2.1	1.2	1.2	7.7	1	3.95	3.35	0.35	0.4	7.9	0
TEW-7197	B. Kinase inhibitor	Other kinase inhibitor	0	0	0	0		0.1	0.8	0	0.6	0	0	0	1.8	0		0	0	0	0	2.9	0	0	0	0	0	0	0	0	0	3.3	0	0	0	0	0	0	6.4	0	0	0	0	0.8	0	0	1.3	0.3	1.5	0	0	3	0.65	0	0	0.0	0	1.5
TG100-115	B. Kinase inhibitor	PI3K or MTOR inhibitor	0	0.8	1.2	0		0	0.1	0	0.1	0	0.3	0	1.5	3.7		0.1	0.1	0	0	0	0.3	0.8	0	0	0	0	0	0.1	0	0.1	1.1	0.2	0	0	0	0	0	0.2	0.3	0	0	0.8	0.3	0.3	0.2	0	0	1.2	0	0	1.45	0	1.5	1.6	0	0
TGR-1202	B. Kinase inhibitor	PI3K or MTOR inhibitor	0	0.7	1.4	0		0	0.1	0	0	0	0	0	0.8	0		0	0	0.2	0	0	0	16.8	0	0	0	0	0.4	0	0	0	0.2	0	0	0	0.1	0	0	0	0	0	0	0.8	0	1.1	0	0	0	1	0.1	0	0	0.35	0	0.1	0	0
TGX-221	B. Kinase inhibitor	PI3K or MTOR inhibitor	1.6	0.8	0.1	4.6		1.2	1.2	0.3	2.7	3.7	4.6	2.8	6.6	10.8		0.7	2.3	0	0.2	0.7	0	2.2	0	0	0	0.2	1.5	1.2	0.2	2.5	2.7	0	0.9	0	0	0.5	1.9	0	2.4	0.3	0	0.8	3	2.9	2.6	3.4	5.1	4.9	0.4	0.8	5.15	1.2	0.45	0.0	0.5	1.3
TH588	G. Metabolic modifier	Other metabolic modifier	4.7	0	0	1.3		0.3	0	0	0.6	0.5	7.7	9.4	0	6.5		2.9	1.9	2.2	2.6	2.7	0.4	4.9	1	0.5	0.1	2.5	1	0	3.3	2.7	3.7	3.5	0.2	0	0.3	4.9	1.7	0	1.6	1	0	0.8	1.4	6.4	1	3	0.3	7.5		0	3.15	4.2	1.85	4.4	0.2	0.1
Thalidomide	E. Immunomodulatory	Other immunomodulatory	0	0	0	0	0	0	0	0	0	0	0	0	0	0.6		0	0	0	0	0.8	0	0	0	0	0.7	0.6	0.2	0	0	0	0	0	0	0	0	0	0	0	0	0.2	0	0.8	0	23.1	0	0.5	0	2.3	0	0	0.5	0.85	0.25	0.0	0	0
Thioguanine	A. Conventional chemotherapy	Antimetabolite	12	4.2	3.7	2.8	0	0	0	0	0	4	13.4	14.3	5.7	9.4		0	0	0	1.4	0	0.8	0.5	0	0	0	0	3.6	0	3.3	0	3	3	0	0	0	7.1	11.6	2.5	0.9	0	3.6	0.8	2.5	28.2	6.4	12.2	3.2	13.5	0	7.8	4.65	0	2.95	3.7	0	6
THZ2	B. Kinase inhibitor	CDK inhibitor	22.6	28.5	27.5	17.1		15.7	19	16.1	22.6	21.2	29.4	1.6	22.5	25.4		18.6	15.4	13	0	20.5	0	11.6	16.5	18.3	14.9	16.1	21.5	17.3	23.4	20.2	19.3	14.9	18.3	9.2	10.9	23.3	19.7	16.5	17.9	14.9	15	0.8	14.8	26.9	13.4	23.2	13.9	27.1	0	22.3	18.05	24.85	13.95	11.7	18.7	25.5
TIC10	X. Other	Other	6	10.7	12.4	8.1		0.2	2.4	4.1	2.1	0.4	12.3	11.2	2	8.9		2.1	0.3	0.8	0	3.8	4.4	0	0.4	3.6	0.6	2.7	1.6	0.6	2.4	0.5	1	0	0.9	2.2	1.3	1.6	2.3	0.1	0.8	1.3	1.7	0.8	0	8.3	7	10.2	0.9	10.4		8.4	2.35	4.4	2	0.5	0.9	0.1
Tideglusib	B. Kinase inhibitor	Other kinase inhibitor	0	0	0	0		0	0	0	0	0	0	0.9	0	1.1		0.1	0	0.2	0.6	0	0	0	0	0	0	0	0	0	0	0	0.1	0	0	0	0	0	0	0	0.2	0	0	0.8	0	0	0	0	0	0	0	0.2	0	0	0	0.0	0	0.1
Tipifarnib	C. Differentiating/epigenetic modifier	Other differentiating/epigenetic modifier	10.5	8.2	6.9	4.2		1.5	1.9	3	2.7	4	22.8	3.5	4.4	0.8		3.9	2	6	3.5	8.7	6.6	3.6	5.3	11.8	6.5	4.9	7.5	3.7	2.3	8.5	3.1	1.9	7	1.2	0.9	0	6	6.5	1.8	2.7	4.9	0.8	2.7	7.8	6.9	3.9	2.4	23.1	3.8	8.5	6.05	7.8	8.3	3.3	4.9	1.6
Tirabrutinib	B. Kinase inhibitor	BTK inhibitor	4.6	0	0	0		0	0	0	0.1	0	0	0.4	0.8	4.4		0.8	1.8	0.5	0	0.2	0	0.2	1.8	0.9	0	0.1	0	0	0	0	0	0	0.2	0	0	0	0.6	0	0	0	0	0.8	0	3.4	0	0	0.3	0	0	0	0.15	0	0	0.0	0.1	0
Tivantinib	B. Kinase inhibitor	MET inhibitor	3.3	2.4	0.3	0.9		0.3	0.4	0.2	0.2	0.7	4	3.5	4.5	4.9		3.1	1.2	3.5	4.6	4.8	2.4	0	0.7	0	0.6	1	1.9	1.7	3.7	4	3.7	1.9	2.3	0	0	2.5	0.9	1.2	3.4	1.5	2.2	0.8	2.2	6.9	2.5	2.3	0.6	3.8	5.6	3.5	4	3.45	4.05	3.6	0.6	3.6
Tivozanib	B. Kinase inhibitor	VEGFR inhibitor	3.7	1	4.2	0.8	4.9	1	0.7	1.9	3.2	1.3	7.7	5.9	4.1	6.2		0.8	1.4	0.9	6.2	1.9	3.3	4	1.2	5.2	0	2	4	2.2	5.9	4.8	5.6	6.8	3	0.5	0.4	4.3	5.5	4.5	3	3.3	4	0.8	4	3	3.1	4.6	2.4	7.7	2.3	1.1	5.5	2.5	5.55	5.8	0.6	2.2
Tofacitinib	B. Kinase inhibitor	JAK inhibitor	0	7.4	3.1	0	0	0	0	0	0.8	0	0	0	0	0		0	0	0	0	0	0	0	0.6	0	4.9	0	0	0	0	0.1	0	0	0	0.5	0	0	0.1	0	1.4	0	0	0.8	0	0	0.3	0	0	2.6	0.3	0	0	0	0	0.0	0.5	0.3
Topotecan	A. Conventional chemotherapy	Topoisomerase inhibitor	16.4	9.5	5.3	7.7	6.6	2	6.2	6.9	6.3	8.7	26.2	13.2	7.7	11.4	7.9	11.1	9.1	5	2.5	12.8	3.7	3.1	8.3	6.3	6.1	7.8	8.3	8.4	7.2	7.6	5.1	3	12.6	9.3	11.9	3.3	12.8	6.5	4.8	7	4.2	0.8	3.7	16.1	6.1	14.7	3.8	26	8.2	18	20.45	13	4.9	4.6	8.6	6.7
Toremifene	F. Hormone therapy	Antiestrogen	4.1	4.4	2.8	2.2	2	1.5	3.1	6.9	5	3.7	8	5.5	4.1	5.5	2.1	4.9	4.7	5.5	1	3.6	0	1.6	4.6	4.3	6.6	4.4	1.3	5.7	0	3.2	0.5	2.3	5.8	0.6	0.9	3.1	8.1	0.3	1.8	4.9	1.4	0.8	1.6	3.5	0.1	5.2	4.6	6.9	3.9	3.5	2.95	5.2	0.1	1.3	5.9	0
Tosedostat	X. Other	Protease/proteasome inhibitor	3.7	1.6	2.6	1.9		0	2.8	0	6	2.6	8.4	0	1.1	6.5		10.2	6	10.1	2.9	13.3	0	12.3	4.2	7.1	3.7	13.6	2.3	1.5	2.8	3.5	0	0	5.5	2.8	1.3	1.1	17.4	0	0.1	3.6	0	0.8	0.1	11.7	7.1	6.4	9.3	7.6		6.4	10.55	14.2	0.1	0.0	4.2	4.1
Tozasertib	B. Kinase inhibitor	Aurora kinase inhibitors	7	8.7	4.8	2.5		0.5	0	2.1	1.7	0.3	17.5	0	5.6	7.1		3.2	1.2	0	3.9	1.3	3	5.5	0.7	0	0.3	0.6	7.9	2.7	7.2	5.1	7.2	6.8	2.5	3	2.3	8.7	2.6	6.2	5.1	1.6	5.1	0.8	3.9	9.4	5.7	4.3	2.3	18.4	0	6.9	18.6	3.45	8.5	6.8	1.4	3.4
TRAM-34	X. Other	Other	0	0	0	0		0	0	0	0	0	0	0	0	0		0	0	0	0	0	0	0	0	0	0	0	0	0	0	0	0	0	0	0	0	0	0	0	0	0	0	0.8	0	0	0	0	0	0		0	0	0	0	0.0	0	0
Trametinib	B. Kinase inhibitor	MEK1/2 inhibitor	19.8	8.9	8.1	7.4	7	0.9	9.2	8.5	8.5	6.3	17	4.2	10.5	12.7	5.4	2.7	3.9	6.2	1.7	4.7	2.6	2.5	9	2.6	9.2	7.3	8.3	6.9	5.2	0.7	8.4	9.1	5.1	10.7	10.3	4.6	16.6	11.4	6.4	7.7	7.4	0.8	5.8	6	3.5	9	9	18.5	0	19.2	9.6	5.05	9.15	6.3	4	2.8
Tretinoin	C. Differentiating/epigenetic modifier	Retinoic acid receptor ligand	6.7	0	0	0	0	0	0	0	0	0	0	0	0	0		0.6	0	0	1.4	0	2.5	0	0	0	0	1.4	5.1	0	5.2	0	1.9	0	1.4	0	0	0	0	3.6	0.4	0	0	0.8	0	0	0	0	0	0.1	0	0	0	0.85	1.5	1.0	0	0
Triapine	G. Metabolic modifier	Other metabolic modifier	3	2.3	2.3	0.5		0	0.8	0.1	0	1.4	7.5	3.7	0.6	1.6		2.8	0.6	0	0.1	2.3	0	5.8	1.5	0.2	1.3	2.6	0.2	0.8	3.1	1.3	0.5	0.4	0.6	1.9	2.8	1.9	1.1	1.1	0	1.6	0.1	0.8	0	2.3	0	2.6	0	5.9		8.8	3.6	1.7	1.45	0.0	0	1.5
Triciribine	B. Kinase inhibitor	AKT inhibitor	25.5	6.3	13.6	7.6		8.2	12.6	11.8	10	6.3	19.1	19.4	12.5	9.6		14.6	12.3	12.8	16.6	11.7	10	5.1	14.5	11.2	9.6	16.4	12.1	16.5	2.7	11.9	10.9	13.1	14.2	3	5	9.8	17.9	7.2	6.3	11.3	7.2	0.8	5.6	8	16.4	20.1	6.8	23.5	13.8	12.7	12.75	15.1	12.45	10.8	12.3	10.6
Trifluridine	A. Conventional chemotherapy	Antimetabolite	2.4	0	0	0	0	0	0	0	0	1.2	8.2	2.2	0	1.6		0	0	0	0	0.9	0	0.1	0.1	0	0	0.2	0	0	0	0	0	0	0	0	0	0	0	0	0	0	0	0.8	0.1	40.8	0	2.9	0.6	12.8	0	5.4	3.05	0	0	0.0	0	0.6
Tubacin	C. Differentiating/epigenetic modifier	HDAC inhibitor	3	3	0	0		0.3	0	0	0	4	12.9	0	6.3	7		1	2.4	0.9	0.4	3.7	1.5	0	3.4	4.8	1.4	5.5	2.3	0	1.2	0	0.7	0.3	0	0	0	1	0	0	1	0	3.4	0.8	0.5	2.4	3.2	3.7	1.6	13.4	3.8	4.8	3	2.4	1.25	1.8	2.4	0.2
Tubastatin A	C. Differentiating/epigenetic modifier	HDAC inhibitor	0	0	0.5	0.2		0.1	0	0	0.9	1	1.4	0.2	2.5	0		0	0	0	0.3	0.4	3.6	0	4.3	6.3	2.1	0.8	3	1.4	0.2	0	3.5	1.7	2.7	0	0	0.7	0.2	2.6	0	0.9	2.6	0.8	0	0	1.9	2.5	0.3	2.1	0.1	4.1	0.5	0	2.25	2.0	0	0
Tucatinib	B. Kinase inhibitor	EGFR or ERBB2 inhibitor	0	0	0	0		0	0	0	0	0	0	0	0	0		0.2	0	0	0	0	0	0	0	0	0	0	0	0	0	0	0	0	0	0	0	0	0	0	0	0	0	0.8	0	0.2	0	0	0	0	0	0	0	0	0	0.0	0.2	0
Tucidinostat	C. Differentiating/epigenetic modifier	HDAC inhibitor	3	8.1	9.6	1.2		0	1.3	2.4	4.6	5.6	8.8	1.5	3.4	8		8.7	6.6	2.5	2.6	4.4	6.9	1.3	5.6	9	4.6	6.6	6.2	5.8	4.8	6.1	3.8	4.4	8	0.9	0.3	5.7	3.3	3.2	2.2	6.4	5.3	0.8	3.1	6.2	2.4	6.7	0.3	9.2	7.6	4.1	3.25	6.1	4.4	3.5	8.2	0
UCN-01	B. Kinase inhibitor	Other kinase inhibitor	16.1	17.5	17.3	21.8		18.8	13.5	14.4	21.9	13.2	15.3	12.3	18.8	20.5		18.7	14.4	19.4	16.8	12.7	18.2	16.3	19.4	17.9	15.4	19.2	24.6	20.6	26	27.2	23.6	22.2	25.6	14.3	16.9	26.5	23.7	19.6	23.5	25.5	20.8	0.8	20	16.7	14.3	15.2	11	12.8	16.9	24.2	24.3	21.35	22.2	19.9	16.8	18.5
Ulixertinib	B. Kinase inhibitor	Other kinase inhibitor	15.1	5.3	1.9	3.3		1.6	5.3	6.3	5.3	4.7	17.4	4.4	9.2	11.5	3.7	3.7	3.7	4.7	0	5.9	3	2.6	9.8	0.2	4.1	5.6	5.7	6.1	5.9	3.4	7.6	7.4	7	8	4.6	0.9	16.7	9.1	4.1	6	6.6	0.8	4.4	9.6	1.8	6.2	8	17.4	0	13.2	6.25	6.4	6.25	3.9	4.6	2.2
UM729	C. Differentiating/epigenetic modifier	Other differentiating/epigenetic modifier	1.5	0.1	0	0.8		0	0	0.7	0	0	3.5	2.1	0.2	0.6		1.3	0.9	0.2	6.8	0	0	4	0.9	2.1	2.8	0.6	1.3	0	1.4	0	3.5	3.9	0.2	0	0	1.2	0	0.7	0.2	0	0	0.8	0	0.7	0	2.6	0	4.8	0	2.5	0.95	1.2	2.45	2.1	1.2	0.7
UNC0638	C. Differentiating/epigenetic modifier	Other differentiating/epigenetic modifier	4.5	5.4	7.5	4.3		0	0	2.3	0.4	2.8	5.7	0.8	5.1	4.4		0.8	5.4	0.4	4.6	0.5	0	1.6	2.4	4.7	3.2	2.4	6	0.8	5.7	0	6.6	6.7	1.4	0	0	0	2.2	6	5.1	0.1	1.4	0.8	0	2.9	2.7	6.1	1.4	5.5	0.2	6.5	5.9	1.55	6.75	5.9	5.1	2
UNC0642	C. Differentiating/epigenetic modifier	Other differentiating/epigenetic modifier	1.3	5	5.3	1.6	0	0	0	0.1	0	1.4	4.5	0.6	3.2	2.5		1.3	1.4	0	5.3	0	0	2.1	2	1.8	2.2	0.7	5.1	0	4.5	0	4.7	3.3	1.5	0	0	4.2	0.2	0.3	0.6	0	0	0.8	0.1	2.2	0.2	1.5	0.8	3	0	4.5	6.05	1.05	5.95	4.5	3.8	4.4
UNC1215	C. Differentiating/epigenetic modifier	Other differentiating/epigenetic modifier	0	0	0	0		0	0	0	0	0	0	0.1	0	0		0	0	0	1.5	0	0	4	0	0	0	0	0	0	0	0	0	0	0	0	0	0	0	0	0	0	0	0.8	0	0	0	0	0	0	0	0	0	0	0.35	0.0	0	0
UNC2881	B. Kinase inhibitor	Other kinase inhibitor	1.7	0	0	0.3		0	0	0	0	0.3	4.6	0	1.8	2.4		0.1	0	0.1	0.9	0	1.8	1.2	0	0	0	0	2.7	0	2.7	0	1.6	3.8	0	0	0	2.7	0.5	1.3	0	0	1.3	0.8	1.3	0	0	0.1	1.2	2	0	1.2	1.5	0.15	2.7	1.3	0.5	0.7
Upadacitinib	B. Kinase inhibitor	JAK inhibitor	0	6.2	1.3	0	0	0	0	0	0	0	0	0	0	0		0	0	0	0	0	0.8	0	0.2	0	0	0.1	1.4	1.8	0.6	5.2	0.2	0	0	0.2	0	1.9	0	0	0	0.1	0	0.8	0.6	0	0	0	0	2.3	0	0	0	0	0	0.0	0	0
Uprosertib	B. Kinase inhibitor	AKT inhibitor	5	1.1	0	1.2		1	8.2	13.6	5.4	0.3	7.1	0	0.9	3.9		2.5	1.9	2.5	1.8	1.4	0	4.8	6.8	4.4	10.2	8.5	3	1.7	7.7	2	4.5	2.1	0.5	0	0	0.8	8	0	2.9	0	0	0.8	2.7	3.3	0	0.1	2.1	11	1.5	2.9	0.6	6.25	3.3	1.0	2.7	0
URB597	G. Metabolic modifier	Other metabolic modifier	0.1	0.1	0	0		0	0	0.3	0.1	0	0.8	0.7	2.5	0		0	0	0	0	0	0	0.1	0	0	0	0	0	0	0	0	0	0	0	0	0	0	0	0	0	0	0	0.8	0	0	0	0	0	1.2		0	1.6	0	0	0.0	0	0
Valproic acid	C. Differentiating/epigenetic modifier	HDAC inhibitor	0	0	0	0	0	0	0	0	0	0	0	0	0	0		0	0	0	0	0	1.6	0	0	0	0	0	0	0	0	0	0	0	0	0	0	0	0	0	0	0	0	0.8	0	0	0	0	0	0	0	0	0	0	0	0.0	0	0
Valrubicin	A. Conventional chemotherapy	Topoisomerase inhibitor	11.6	13.4	15.4	2.4	0.1	2.4	7.3	5.4	6	4.6	22.1	0.5	5.3	6		7	6.6	4.9	8.1	7.7	7.1	4.1	5.5	3.3	4.8	0.1	9.9	6.6	9.7	7.4	7.5	8.7	6.2	4.1	3.4	11.2	3.6	9.6	8.1	5.1	6.6	0.8	3.9	8.7	3.8	8.8	2.4	20.1	9.1	12.2	11.85	10.15	8.65	7.7	7.3	8.7
Vandetanib	B. Kinase inhibitor	Multi kinase inhibitor	6.5	0	0	0.1	0.4	0.1	1.4	3.4	4.3	0	0.2	0	3.6	5.7		2.2	1.1	0.2	0.9	0	2.3	0	1.3	1.7	1.4	0.6	0	4.1	3.8	5.5	0.1	0.2	3.4	0	0	0	1.6	0.2	0	2.8	0.1	0.8	0	2.3	0	0	0.8	0	3.3	0	2.95	1.65	0.6	1.0	0.3	0
Varespladib	X. Other	Other	0.8	0	0	0		0	0	0.3	0	1	2.1	0	0	0	0	0	0	0	0	0	0	0	0	0	0	0	0	0	0.4	0	0.7	0	0	0	0	0	0	0	0	0	0	0.8	0	0.7	0	0	4.1	2.9		0	0	0	0	0.0	0	0
Varlitinib	B. Kinase inhibitor	EGFR or ERBB2 inhibitor	10.6	0	0.9	1.4		0.1	0.6	1.1	3.6	0	3.8	0.7	2.8	9.5		5.7	4.7	3.5	2.3	0.1	0	1.3	3.6	3.7	0.5	2.6	0	3.2	1.3	1.7	2.5	0	4.1	0	0	2.9	2.5	0	0	2.5	0	0.8	0	4.1	0.2	0.2	1	1.5	0	0.3	2.5	2.35	0.9	0.2	2.8	0
Vatalanib	B. Kinase inhibitor	VEGFR inhibitor	0	0	0	0.5	0.1	0	0	0	0	0	0.3	0	0	0.2		0	0	0	0	0.1	0	0	0	0	0	0	0	0	0	0	0	0	0	0	0	0	0	0	0	0.5	0	0.8	0	0	0	0	0	0	0	0	0	0	0	0.0	0.1	0.1
VE-821	B. Kinase inhibitor	Other kinase inhibitor	0	0	0	0		0.9	0	0.3	0	0	5.2	0	0	0.8		1.9	2.3	1	0	0.1	0	0	3.2	1.3	0.3	0.8	0	0	0	0	0	0	0	0	0	0	0.8	0	0	0	0	0.8	0	0	1.1	0	0.2	4.4	0.6	2.6	0.7	0.8	0.05	0.0	0	1.6
Veliparib	C. Differentiating/epigenetic modifier	PARP inhibitor	0	0.3	2.4	0.1		0	0.2	0	0.5	0.6	4.3	0	2.8	0.5		0	0	9.2	0	1.3	0	3.7	0.8	2.8	0	0	0	0	3.9	0	1	0.1	0	0	0	0	6.1	0	0	0.4	0	0.8	0	0	0	1.4	0.4	5.1	0.1	2.6	1.05	1.5	1.25	0.0	1.8	0
Vemurafenib	B. Kinase inhibitor	RAF inhibitor	0	0	0.1	0	0.4	1	0	0.1	1.3	0	1	0.4	0.2	1.1	3.8	2.7	1.3	3	0	0.4	0.1	4.3	1.1	2.9	3.5	2.9	3	1.6	2.4	4	1.7	0.2	3.3	0	0	2.5	3.5	0.8	2.9	1.8	0	0.8	0	0.2	0	0.7	0	0	0	1.7	0.85	2.9	1.2	0.6	1	0.2
Venetoclax	D. Apoptotic modulator	BH3 mimetic	0	0.5	6.3	0	0.5	0	0	0	0	0	0	0	0	0	0	0	0	0	0.1	0.3	0	1.2	0	0	0	0	0	0	0	0	0	0	0	0	0	0	0	0	0	0	0	0.8	0	0	0	0	0	0.4	0	0	0	0	0	0.0	0	0
VER 155008	X. Other	HSP inhibitor	9.6	1	0.2	6.4		1.4	5.2	3.2	5.1	5.8	11.1	0.9	2.3	6.2		3.4	4	2.8	0	3.3	1.1	5.1	2.2	3.1	3.2	1.2	0	3.8	0	4.7	2	0.7	3.8	1.4	2.2	0	5.1	0	0.6	4.2	1	0.8	0	4.1	1	7.7	2.7	8.6		5.5	4.65	4.35	2.55	1.0	2.4	0.3
Verdinexor	D. Apoptotic modulator	Other apoptotic modulator	14.8	7.8	10.5	2.4		5.1	8.1	7.7	8.5	6.7	13.9	11	9	11.7		7.9	7.6	8.6	0	17.2	5.6	11.2	4.4	8.1	7.2	5.8	3.2	0	5.1	4.6	6.5	6.8	6.5	4.8	6	1.7	8.8	2.6	3.6	5.9	5.1	0.8	0.6	8.7	6.7	10.9	0.1	13.9		14	8.9	11	4.75	1.9	7.3	4
Vesatolimod	E. Immunomodulatory	TLRs ligand	0	0	0	0		0	0	0	0	0	0	0.5	0	1		0	0	0	0	0	0	0	0	0	0	0	0	0	0	0	0	0	0	0	0	0	0	0	0	0	0	0.8	0	0	0	0	4.1	1	0	0	0	0	0	0.0	0	0
VGX-1027	E. Immunomodulatory	Other immunomodulatory	0	0	0	0		0	0	0	0	0	0	0	0	0		0.2	0	0	0	0	0	0	0	0	0	0.5	0	0	0	0	0	0	0	0	0	0	0	0	0.2	0	0	0.8	0	0	0.3	0	0	0	0	0	0	0	0	0.0	0	0
Vidofludimus	C. Differentiating/epigenetic modifier	Other differentiating/epigenetic modifier	1.5	0	0	0		0	0	0	0.1	1.3	1.6	1.6	0	0		0.2	0.3	0	0	0.6	0	3.4	0	0.1	0.1	0	0	0	0.1	0	0	0	0	0	0.3	0	0	0	0	0	0	0.8	0	0.1	0	1.1	0	1.8	0	0	0	0	0	0.1	0	0
Vinblastine	A. Conventional chemotherapy	Mitotic inhibitor	21.6	10.7	7.2	5.3	0	6.8	5.3	6.6	7.2	8.7	26.5	1.7	10.5	18.2	9.9	10.4	4.7	1.5	7.4	13.2	4.8	6.8	0	0.6	0.4	0.6	18.9	5.1	22.2	5.6	8	3.5	4.8	0	0	0	9.2	13.5	15.7	4.4	20.2	0.8	10.9	15.7	6.7	12.7	4.8	25.7	2	10.8	12.05	5.95	7.55	2.2	1.3	13.6
Vincristine	A. Conventional chemotherapy	Mitotic inhibitor	19.7	14.7	10	1.8	0.5	0	1.7	4.5	1.6	6.1	27.8	0	12.6	17.9	10.9	11.4	5.1	1.9	9.5	15.7	5.5	2.2	3.3	1.1	0	3	14.6	3.9	19.3	4.5	9.5	3.4	4.2	0	0.6	3	6.7	6.7	12	3.8	17.8	0.8	11.9	27.7	7.2	8.9	3.3	26	1.6	11.4	9.65	5.65	9.3	6.1	1	12.6
Vinflunine	A. Conventional chemotherapy	Mitotic inhibitor	4.3	1	0.2	0	0	0	0	0.7	0.2	0	4	0	3.7	2.9	2	2.6	2.3	1.7	4.2	4	0	0.8	1.1	0	0	1.7	2.5	1.4	4.9	1.1	4.3	3.4	0.6	0	0.6	0	0.5	1	4.1	0	2.9	0.8	3.3	16.6	2.4	1.9	0.1	3.8	0	3.5	5.25	4.9	3.4	4.6	0.1	2.7
Vinorelbine	A. Conventional chemotherapy	Mitotic inhibitor	28.6	18.2	12	6.2	7.8	5.7	7.9	6.6	5.4	13.9	38	10.2	26.4	29.9	15.1	20.9	12.3	8.9	31.4	26.9	21.1	19.4	9.8	2.6	8.5	7.8	21.3	13.4	26.2	14.6	28.4	21.9	16.7	1.2	0.4	4.1	11.5	26.9	31	14.7	25.2	0.8	25.8	41.9	21.4	26.7	7.8	38	4.8	30.3	24.15	24	32.55	32.9	7	18.3
Vismodegib	X. Other	Hedgehog inhibitor	0	0	0	0	0	0	0	0	0	0	0.4	0	0	0		1.2	0	0	0	0	0	0.5	0.2	2.4	2	0	0	0	0	0	0	0	0	0	0	0	2.6	0	0	0	0	0.8	0	0	0	0	0	0.3	0.9	0	0	0	0.35	0.0	0.5	0.6
Vistusertib	B. Kinase inhibitor	PI3K or MTOR inhibitor	21.1	7.3	5	16.2		11.5	13.5	15.7	17.6	12.5	24.1	9.8	12.7	23.4		12.8	9.7	12.8	14.6	9.1	8.1	16.7	13.9	13.3	16.2	10.7	16.1	9.1	17.2	14.3	15.8	13.8	11.5	7.6	10	16.9	16.7	12.7	14	10	12.3	0.8	13.9	17.3	13.4	12.5	10.5	26.1	7.1	17	11.8	13.3	14.35	14.8	14.7	11.5
VLX1570	X. Other	Protease/proteasome inhibitor	22.9	26.4	27	11.4		16.7	10.6	20.3	10.1	22.1	28.7	25.3	23.3	24.1		19.3	17.9	24.1	17.5	20.9	0.6	19.2	22.6	21.2	21.9	21.5	17.6	26.1	17.3	22.7	8.8	4.1	24.1	6.9	5.2	11.4	12.7	5.3	5.9	19.9	10.9	0.8	9.2	29	17.1	24.2	20.1	24.7		23.1	21.65	23	12.9	4.5	14.4	5.5
Volasertib	B. Kinase inhibitor	Other kinase inhibitor	15.5	7.2	2.2	1	0.6	2	1.3	1.9	2.9	2.7	18	5.9	18.2	11.9	0	5.9	1.3	3.5	1	9.3	0	0.1	2.9	0	0	2.7	4.8	2.3	2.8	1.6	3	0.1	1.1	0.2	0	0	1.9	0	1.2	2.1	2.7	0.8	0.3	16.7	5.1	13.1	4	17.9	0	12.8	11.85	7.75	2.4	0.8	1.9	7.5
Vorinostat	C. Differentiating/epigenetic modifier	HDAC inhibitor	8.3	5.9	7.8	1.1	5.7	2.6	4.2	3.9	9	7	11.3	16.5	8.5	10.9	9.1	7.1	7.2	3.6	0	6.4	4.4	2.1	14.2	11.5	12.4	10.3	6.4	4.4	4.3	3.6	3.4	5.8	5.8	2.9	3.3	2.9	7.2	2.1	2.8	4.2	4.5	0.8	4.4	3.8	4.8	11.9	5.8	12.6	1.7	8.3	8.6	6.45	4.4	2.7	8.3	3.3
VS-4718	B. Kinase inhibitor	Other kinase inhibitor	12.9	2.3	3.2	6.8		0.1	3.5	7.2	3.7	5.6	14.4	6.5	10.1	7		5.6	5.6	4.6	10.6	7.7	8.6	8.6	4.4	0	2.3	1	12	8.8	9.7	10.4	8.9	9	10.6	6.9	7	9.3	13.1	9.9	9.6	8.6	9.3	0.8	9.6	7.4	7	4.4	13.2	9.9	7.1	7.6	5.55	6.55	12.3	7.7	9.4	5.4
WEHI-539	D. Apoptotic modulator	BH3 mimetic	0	0	0.1	0		0	0	0	0	0	0	0.1	0.2	0.5		3.6	0.8	0.5	0.9	1.4	0	4	1	0	0.3	0.3	0	0	0.1	0.1	0	0.1	0	0	0	0	0	0	2.2	0	0	0.8	0.2	1.1	0	0	0	0		1	0.6	4.75	0.45	0.0	5.2	0
XAV-939	C. Differentiating/epigenetic modifier	Other differentiating/epigenetic modifier	0	0	0	2.1		0.8	0.5	0.4	0	0.1	0	1.5	2.3	9.1		3.7	0.7	0.4	2.3	0	0.2	6.3	1	2	0	0	2.3	0	3.3	0.6	0.2	1.5	0	0	0	1.1	3.3	2.5	5.4	0	0.2	0.8	2.3	7.1	0	0	2.4	0	0	0.5	0.7	0	0.15	1.7	1.5	0
ZSTK474	B. Kinase inhibitor	PI3K or MTOR inhibitor	19	6.4	6.9	12.6		5.7	7.1	10.3	9	8.2	15.3	10.8	14.3	22.6		14	11.9	10.2	15.6	3.2	8.6	11	9.5	9.7	8.5	11.1	14.1	9.4	16.7	12.4	14.6	12.4	10.8	5.1	5.6	15.6	10.5	9.4	11.9	8.5	9.3	0.8	11.5	15	7.8	8.1	9.5	19.4	6.4	7.7	10.5	8.8	14.95	14.0	12.2	4.4

Supplemental Table 4

DSS comparison between all PDCs derived from ascites or tissue, based on individual drugs

DRUG_NAME	p.value	mean_ascites	mean_tissue	logFC	p.adj
1-methyl-D-tryptophan	0.39	0.27	0.00	0.26	0.7563527
4-hydroxytamoxifen	0.25	4.09	3.04	1.05	0.6384922
8-amino-adenosine	0.12	21.07	19.43	1.64	0.5429712
8-chloro-adenosine	0.03	12.60	7.96	4.64	0.4960204
A-1155463	0.26	9.52	13.53	-4.01	0.6502002
A-1210477	0.63	6.57	6.33	0.24	0.8454114
A-1331852	0.46	4.88	6.26	-1.38	0.7629368
A-366	0.01	1.59	0.20	1.38	0.4828655
A-419259	0.97	10.83	11.09	-0.26	0.9905677
ABC294640	0.10	4.74	3.48	1.26	0.5429712
ABT-751	0.10	8.46	5.56	2.90	0.5429712
AMG-232	0.16	2.10	0.37	1.73	0.5663914
AMG-337	NaN	0.00	0.00	0.00	NaN
AMG-925	0.29	0.87	0.16	0.71	0.6739784
AMG319	0.13	0.68	0.09	0.59	0.5429712
APR-246	1.00	0.18	0.13	0.05	1.0000000
AR-42	0.05	10.21	8.07	2.14	0.4989832
ARV-825	0.94	10.05	9.01	1.04	0.9778587
ASP3026	0.78	5.21	5.48	-0.27	0.9096508
AT 101	0.48	14.98	14.20	0.78	0.7629368
AT-406	0.35	2.09	4.24	-2.15	0.7421939
AT13148	0.62	1.32	1.90	-0.57	0.8454114
AT7519	0.16	12.54	11.60	0.93	0.5663914
AT9283	0.89	3.00	2.92	0.08	0.9595549
AVN944	0.05	12.75	8.49	4.26	0.4989832
AZ 3146	0.65	3.14	2.53	0.61	0.8455843
AZ191	0.32	2.07	1.37	0.70	0.6961959
AZD-1080	0.39	0.03	0.10	-0.07	0.7563527
AZD-5363	0.87	4.56	4.01	0.54	0.9595549
AZD-5438	0.95	6.81	6.74	0.07	0.9865162
AZD-6482	0.48	1.76	1.79	-0.03	0.7629368
AZD-8186	0.84	2.05	1.46	0.59	0.9402133
AZD0156	0.69	1.63	1.00	0.63	0.8665021
AZD1152-HQPA	0.09	3.59	1.80	1.79	0.5244741
AZD1208	0.46	1.16	0.80	0.36	0.7629368
AZD1480	0.40	0.72	0.13	0.60	0.7563527
AZD1775	0.09	14.33	10.92	3.41	0.5244741
AZD3759	0.07	1.08	2.17	-1.09	0.5181296
AZD3965	0.92	0.66	0.03	0.63	0.9774233
AZD4547	0.14	1.15	0.00	1.15	0.5429712
AZD6738	0.12	9.04	6.91	2.13	0.5429712
AZD7545	0.11	0.33	0.06	0.27	0.5429712
AZD7762	0.01	6.12	3.54	2.58	0.4713480
AZD8055	0.08	20.49	18.12	2.37	0.5182156
Abemaciclib	0.82	4.21	2.21	2.00	0.9314794
Abexinostat	0.03	12.65	9.16	3.49	0.4960204
Abiraterone	0.46	0.28	0.50	-0.22	0.7629368
Acalabrutinib	0.14	0.13	0.00	0.13	0.5429712
Acitretin	0.52	1.39	0.66	0.73	0.7905405

Afatinib	0.07	3.98	6.63	-2.65	0.5181296
Afuresertib	0.98	3.75	3.33	0.41	0.9944730
Aldoxorubicin	0.25	7.24	5.53	1.71	0.6417486
Alectinib	0.14	0.79	0.29	0.51	0.5429712
Alisertib	0.18	6.53	3.93	2.60	0.5990458
Allopurinol	0.77	2.19	0.08	2.11	0.9096508
Alpelisib	0.06	2.56	3.64	-1.08	0.5109537
Altiratinib	0.94	4.64	4.37	0.26	0.9778587
Alvocidib	0.41	15.59	15.33	0.25	0.7563527
Amcasertib	0.12	3.78	1.67	2.11	0.5429712
Aminoglutethimide	0.74	0.04	0.15	-0.11	0.8973379
Amsacrine	0.66	6.65	4.19	2.47	0.8455843
Amuvatinib	0.06	2.67	0.91	1.76	0.5027989
Anagrelide	0.93	1.25	1.62	-0.37	0.9778587
Anastrozole	0.30	0.00	0.02	-0.02	0.6869234
Apalutamide	0.73	0.06	0.01	0.05	0.8829666
Apatinib	0.71	0.56	0.16	0.40	0.8696719
Arsenic(III) oxide	0.41	0.01	0.00	0.01	0.7563527
Asciminib	0.80	0.63	0.65	-0.02	0.9264843
Atorvastatin	0.19	1.85	2.06	-0.21	0.6012754
Auranofin	0.12	7.68	4.98	2.70	0.5429712
Axitinib	0.12	2.07	0.67	1.39	0.5429712
Azacitidine	0.01	4.93	2.64	2.29	0.4828655
BAY 87-2243	0.09	8.38	2.79	5.58	0.5244741
BAY-1436032	0.22	0.34	0.05	0.29	0.6384922
BCI	0.13	16.37	14.28	2.09	0.5429712
BGB-283	0.07	3.85	2.63	1.22	0.5181296
BGB324	0.25	4.05	2.88	1.16	0.6384922
BI 2536	0.29	10.30	5.53	4.76	0.6739784
BIIB021	0.25	12.38	10.17	2.22	0.6400935
BMS-754807	0.07	9.50	11.83	-2.33	0.5181296
BMS-777607	0.43	0.34	0.02	0.32	0.7563527
BMS-911543	0.58	0.26	0.37	-0.11	0.8347758
BMS863233	0.46	1.23	0.66	0.57	0.7629368
BRD7116	0.02	1.99	0.37	1.62	0.4828655
BX-912	0.45	3.71	3.04	0.67	0.7629368
Bafetinib	0.14	0.09	0.00	0.09	0.5429712
Baricitinib	0.27	0.00	0.05	-0.05	0.6641408
Belinostat	0.01	13.21	9.36	3.84	0.4713480
Bentamapimod	0.66	1.86	1.42	0.44	0.8455843
Bexarotene	0.59	1.03	1.23	-0.20	0.8347758
Bicalutamide	0.25	0.85	0.38	0.47	0.6405503
Bimatoprost	0.97	0.17	0.08	0.09	0.9905677
Binimetinib	0.63	3.99	3.12	0.88	0.8454114
Birabresib	0.67	9.13	7.14	1.99	0.8589130
Birinapant	0.70	3.24	3.77	-0.52	0.8686009
Bleomycin	0.70	3.69	1.72	1.97	0.8686009
Bortezomib	0.65	23.83	24.60	-0.77	0.8455843
Bosutinib	0.33	7.86	6.93	0.92	0.7064197
Brigatinib	0.09	3.18	4.37	-1.19	0.5244741
Brivanib	0.30	0.00	0.03	-0.03	0.6869234
Bryostatin 1	0.76	1.39	1.69	-0.30	0.9039728
Buparlisib	0.79	7.66	7.51	0.14	0.9162302
C646	0.18	1.17	1.56	-0.39	0.5990458

CC-115	0.97	10.64	10.25	0.39	0.9905677
CC-223	0.14	5.31	4.09	1.23	0.5429712
CC122	0.05	0.41	0.00	0.41	0.4989832
CCT196969	0.86	4.58	3.91	0.67	0.9530552
CEP-32496	0.92	1.74	1.23	0.51	0.9774233
CEP-37440	0.90	7.94	7.86	0.08	0.9700926
CPI-0610	0.17	6.20	3.50	2.70	0.5752278
CPI-360	0.24	0.04	0.00	0.04	0.6384922
CPI-613	0.95	0.09	0.08	0.00	0.9873546
CUDC-305	1.00	8.83	9.02	-0.18	1.0000000
CUDC-907	0.02	30.51	26.02	4.50	0.4828655
Cabazitaxel	0.23	15.71	10.34	5.37	0.6384922
Cabozantinib	0.60	1.49	1.41	0.08	0.8378599
Canertinib	0.96	10.55	11.18	-0.63	0.9873546
Capecitabine	0.43	0.03	0.07	-0.04	0.7563527
Capmatinib	0.21	0.07	0.20	-0.13	0.6384922
Carboplatin	0.10	4.78	3.20	1.58	0.5429712
Carfilzomib	0.46	22.43	20.49	1.94	0.7629368
Cediranib	0.06	0.52	0.05	0.47	0.5027989
Celecoxib	0.46	0.06	0.08	-0.02	0.7629368
Cerdulatinib	0.03	1.96	0.64	1.32	0.4960204
Ceritinib	0.31	2.11	2.61	-0.51	0.6961959
Chloroquine	0.53	6.06	6.36	-0.30	0.7980697
Cilengitide	0.60	1.74	1.03	0.71	0.8378599
Cisplatin	0.13	11.68	8.50	3.18	0.5429712
Cladribine	0.25	4.00	1.19	2.81	0.6384922
Clofarabine	0.12	11.82	5.38	6.43	0.5429712
Clomifene	1.00	3.94	4.07	-0.13	1.0000000
Cobimetinib	0.29	5.81	4.74	1.07	0.6739784
Copanlisib	0.73	8.99	9.93	-0.94	0.8855636
Crenolanib	0.83	4.02	4.09	-0.06	0.9358950
Crizotinib	0.82	0.08	0.10	-0.01	0.9314794
Cytarabine	0.34	5.72	2.78	2.94	0.7208606
Cytarabine/Idarubicin	0.14	9.51	4.90	4.61	0.5429712
DEL-22379	0.95	7.59	7.58	0.02	0.9865162
Dabrafenib	0.62	0.59	0.33	0.27	0.8454114
Dacomitinib	0.48	5.18	6.53	-1.35	0.7629368
Dactinomycin	0.38	24.31	22.76	1.54	0.7563527
Dactolisib	0.15	14.78	12.79	1.99	0.5607478
Danusertib	0.75	8.15	6.60	1.55	0.9003286
Daporinad	0.11	21.35	14.07	7.28	0.5429712
Darapladib	0.13	0.89	0.14	0.75	0.5429712
Dasatinib	0.25	6.01	7.68	-1.67	0.6384922
Daunorubicin	0.23	7.71	5.17	2.54	0.6384922
Decernotinib	0.36	0.23	0.07	0.16	0.7482851
Decitabine	0.27	2.07	1.26	0.81	0.6646899
Deferoxamine	0.13	2.73	1.46	1.27	0.5429712
Dexamethasone	0.28	0.37	0.74	-0.37	0.6727284
Digoxin	0.11	13.92	12.00	1.92	0.5429712
Dinaciclib	0.65	17.37	18.54	-1.17	0.8455843
Disulfiram(+CuCl2)	0.88	4.26	4.18	0.08	0.9595549
Docetaxel	0.30	15.43	11.11	4.32	0.6878734
Doramapimod	0.04	1.81	0.64	1.17	0.4960204
Dovitinib	0.14	0.30	0.04	0.26	0.5525617

Doxorubicin	0.44	6.32	4.74	1.58	0.7563527
Duvelisib	0.60	0.59	0.37	0.22	0.8378599
E7820	0.57	5.69	4.06	1.63	0.8333432
ENMD-2076	0.38	8.64	7.37	1.27	0.7563527
EPZ-5687	0.03	0.43	0.09	0.34	0.4960204
EPZ015666	0.52	1.18	0.45	0.73	0.7905405
EPZ031686	0.14	0.52	0.00	0.52	0.5429712
Eltanexor	0.69	18.60	17.39	1.21	0.8665021
Enasidenib	0.42	0.19	0.23	-0.04	0.7563527
Encorafenib	0.79	0.54	0.40	0.14	0.9170828
Ensartinib	0.66	0.98	0.87	0.11	0.8455843
Entinostat	0.11	5.82	4.38	1.44	0.5429712
Entospletinib	0.66	0.03	0.32	-0.29	0.8455843
Entrectinib	0.21	0.96	0.43	0.54	0.6384922
Enzalutamide	0.45	0.64	0.41	0.23	0.7629368
Enzastaurin	0.82	0.78	0.43	0.35	0.9314794
Epacadostat	0.40	0.29	0.08	0.22	0.7563527
Epirubicin	0.18	7.98	4.42	3.56	0.5990458
Erastin	0.00	15.21	4.88	10.33	0.4713480
Erdafitinib	0.52	1.83	0.07	1.76	0.7905405
Eribulin	0.05	17.07	8.72	8.35	0.4989832
Erlotinib	0.22	6.00	7.61	-1.61	0.6384922
Etoposide	0.22	5.23	2.86	2.38	0.6384922
Everolimus	0.04	6.75	4.00	2.75	0.4960204
Exemestane	0.34	0.38	0.11	0.28	0.7292744
FRAX486	0.33	4.27	3.38	0.89	0.7046038
Fedratinib	0.45	5.96	4.63	1.33	0.7629368
Filanesib	0.07	13.10	7.10	6.00	0.5181296
Filgotinib	0.11	1.88	0.88	1.00	0.5429712
Finasteride	0.47	0.01	0.01	-0.01	0.7629368
Fingolimod	0.02	7.38	5.95	1.43	0.4828655
Floxuridine	0.01	7.82	2.53	5.29	0.4713480
Fludarabine	0.31	1.09	0.36	0.73	0.6961959
Fluorouracil	0.01	4.06	0.78	3.27	0.4713480
Flutamide	0.08	0.33	0.04	0.29	0.5181296
Foretinib	0.61	3.25	2.31	0.94	0.8389581
Fostamatinib	0.17	2.56	2.86	-0.30	0.5829313
Fulvestrant	0.93	0.01	0.01	0.00	0.9776876
GDC-0084	0.78	10.47	10.04	0.43	0.9096508
GDC-0623	0.95	3.61	4.07	-0.46	0.9865162
GDC-0853	0.43	0.02	0.00	0.02	0.7563527
GDC-0919	0.56	0.42	0.37	0.05	0.8236341
GNE-0877	NaN	0.00	0.00	0.00	NaN
GNE-7915	0.24	0.19	0.00	0.19	0.6384922
GSK-1070916	0.19	2.55	1.58	0.96	0.6052212
GSK-2334470	0.57	1.50	1.10	0.40	0.8333432
GSK-461364	0.16	13.46	8.30	5.16	0.5694928
GSK-690693	0.59	5.19	3.60	1.58	0.8347758
GSK-J4	0.70	4.17	2.35	1.82	0.8686009
GSK2256098	0.02	0.60	1.08	-0.48	0.4828655
GSK2636771	0.75	0.77	0.43	0.34	0.9039728
GSK2656157	0.15	1.06	0.42	0.64	0.5663914
GSK269962	0.63	3.23	2.43	0.80	0.8454114
GSK2801	0.62	0.15	0.06	0.09	0.8454114

GSK2830371	0.02	1.27	0.17	1.10	0.4828655
GSK2879552	0.43	0.10	0.01	0.10	0.7563527
GSK343	NaN	0.00	0.00	0.00	NaN
GSK650394	0.88	0.28	0.43	-0.15	0.9595549
GSK923295	0.09	10.66	5.88	4.78	0.5220806
Galiellalactone	0.05	4.53	2.08	2.45	0.4989832
Galunisertib	NaN	0.00	0.00	0.00	NaN
Gandotinib	0.57	5.23	4.83	0.41	0.8333432
Ganetespib	0.60	13.11	11.84	1.27	0.8378599
Gedatolisib	0.08	12.48	10.10	2.37	0.5220806
Gefitinib	0.34	5.95	7.56	-1.62	0.7261571
Gemcitabine	0.05	10.29	4.83	5.46	0.4989832
Gilteritinib	0.65	2.11	1.68	0.43	0.8455843
Givinostat	0.08	5.72	4.01	1.71	0.5220806
Glasdegib	0.27	0.15	0.03	0.12	0.6671774
Glesatinib	0.62	0.94	0.47	0.47	0.8454114
Golvatinib	0.42	0.48	0.95	-0.47	0.7563527
Goserelin	0.77	2.66	0.02	2.64	0.9096508
Hydroxyfasudil	0.47	0.19	0.07	0.12	0.7629368
Hydroxyurea	0.08	5.08	1.61	3.48	0.5181296
I-BET151	0.70	7.04	6.18	0.86	0.8686009
IOX-1	0.32	1.85	0.85	1.00	0.7046038
IOX-2	0.17	0.27	0.35	-0.09	0.5752278
Ibrutinib	0.58	2.39	3.02	-0.63	0.8347758
Icotinib	0.12	2.91	4.24	-1.33	0.5429712
Idarubicin	0.37	9.31	6.55	2.76	0.7497097
Idasanutlin	0.44	3.68	1.19	2.49	0.7563527
Idelalisib	0.06	2.00	0.88	1.12	0.5027989
Imatinib	0.43	0.18	0.43	-0.24	0.7563527
Imiquimod	0.97	2.54	0.01	2.54	0.9905677
Indibulin	0.39	7.86	5.90	1.96	0.7563527
Infigratinib	NaN	0.00	0.00	0.00	NaN
Ipatasertib	0.70	4.58	3.69	0.89	0.8686009
Itraconazole	0.66	1.82	1.39	0.43	0.8455843
Ivosidenib	0.25	0.13	0.02	0.11	0.6384922
Ixabepilone	0.31	3.49	2.48	1.02	0.6961959
Ixazomib	0.85	11.58	10.95	0.62	0.9486213
JPH203	0.42	0.02	0.00	0.02	0.7563527
JQ1	0.22	11.61	8.64	2.97	0.6384922
KD025	0.28	3.55	2.24	1.31	0.6727284
KU-60019	0.76	1.76	1.45	0.31	0.9093617
LY-2584702	0.05	1.80	0.25	1.55	0.4989832
LY-2874455	0.12	5.33	4.85	0.48	0.5429712
LY3009120	0.30	10.11	8.68	1.43	0.6861317
LY3023414	0.25	11.00	9.65	1.35	0.6384922
Lapatinib	0.61	0.83	1.42	-0.59	0.8389581
Larotrectinib	0.14	0.18	0.00	0.18	0.5429712
Lasofoxifene	0.28	0.36	0.17	0.19	0.6727284
Lenalidomide	0.73	0.28	0.10	0.18	0.8840056
Lenvatinib	0.37	0.82	0.20	0.62	0.7497097
Letrozole	0.39	0.21	0.00	0.21	0.7563527
Linifanib	0.49	0.12	0.16	-0.04	0.7675784
Linsitinib	0.42	2.14	2.59	-0.45	0.7563527
Litronesib	0.04	13.46	6.88	6.58	0.4960204

Lomeguatrib	0.19	0.02	0.05	-0.03	0.6052212
Lonafarnib	0.12	12.36	9.53	2.84	0.5429712
Losmapimod	0.04	1.58	0.63	0.96	0.4960204
Lovastatin	0.79	0.29	0.40	-0.11	0.9156139
Lucitanib	0.09	2.19	0.72	1.47	0.5244741
Luminespib	0.63	15.31	13.88	1.43	0.8454114
MK-0752	0.28	0.00	0.02	-0.02	0.6727284
MK-2206	0.64	2.03	1.94	0.09	0.8455843
MK-8745	0.18	4.42	2.03	2.39	0.5942115
MK-8776	0.15	1.26	0.64	0.62	0.5663914
ML323	0.16	1.31	0.25	1.07	0.5663914
ML390	0.23	4.11	2.18	1.93	0.6384922
MST-312	0.05	9.79	6.55	3.25	0.4989832
Marimastat	0.70	0.36	0.34	0.02	0.8686009
Masitinib	0.48	2.03	1.64	0.39	0.7629368
Megestrol acetate	0.32	0.27	0.53	-0.27	0.6961959
Mepacrine	0.05	13.07	10.54	2.53	0.4989832
Mercaptopurine	0.22	4.25	1.42	2.83	0.6384922
Merestinib	0.66	0.48	0.68	-0.21	0.8455843
Metformin	NaN	0.00	0.00	0.00	NaN
Methotrexate	0.55	4.88	3.45	1.42	0.8211493
Methylprednisolone	0.42	0.19	0.78	-0.59	0.7563527
Midostaurin	0.30	6.48	7.98	-1.50	0.6852521
Milciclib	0.98	10.39	9.06	1.33	0.9944730
Miltefosine	0.30	6.83	8.05	-1.22	0.6890909
Mitomycin C	0.66	1.39	0.81	0.58	0.8455843
Mitotane	0.39	1.67	0.17	1.50	0.7563527
Mitoxantrone	0.07	6.50	3.69	2.82	0.5181296
Mivebresib	0.48	19.12	16.37	2.75	0.7629368
Mocetinostat	0.81	9.69	9.31	0.38	0.9289648
Molibresib	0.13	5.91	3.62	2.29	0.5429712
Momelotinib	0.61	3.49	3.25	0.24	0.8427575
Motesanib	0.83	0.85	1.17	-0.32	0.9358950
Motolimod	0.08	0.64	0.34	0.30	0.5220806
Mubritinib	0.63	3.91	1.86	2.05	0.8454114
NMS-873	0.48	9.82	8.11	1.71	0.7629368
NVP-AEW541	0.43	6.93	7.69	-0.76	0.7563527
NVP-BGT226	0.52	16.57	15.27	1.30	0.7929268
NVP-BHG712	0.30	2.31	1.46	0.85	0.6852521
NVP-CGM097	0.87	8.06	7.14	0.92	0.9595549
NVP-LCL161	0.98	4.96	7.04	-2.08	0.9944730
NVP-LGK974	0.08	0.72	0.05	0.67	0.5220806
NVP-RAF265	0.89	2.36	1.76	0.59	0.9595549
NVP-SHP099	0.59	1.47	1.27	0.19	0.8347758
Napabucasin	0.01	18.98	15.16	3.82	0.4713480
Navitoclax	0.53	2.73	5.43	-2.69	0.7944291
Necrostatin 2	0.80	0.05	0.10	-0.05	0.9264843
Neflamapimod	0.05	0.61	0.12	0.48	0.4989832
Nelarabine	0.90	0.48	0.18	0.30	0.9647279
Neratinib	0.57	4.54	5.10	-0.56	0.8333432
Nilotinib	0.01	1.80	0.21	1.58	0.4713480
Nilutamide	0.41	0.02	0.06	-0.04	0.7563527
Nintedanib	0.07	5.68	3.45	2.23	0.5181296
Niraparib	0.55	2.49	1.71	0.78	0.8172852

ODM-201	0.43	0.95	0.39	0.56	0.7563527
ONX-0914	0.24	15.18	14.27	0.91	0.6384922
OSU-03012	0.66	0.28	0.50	-0.21	0.8455843
OTS-964	0.19	13.35	12.22	1.13	0.6012754
OTS167	0.87	13.80	13.39	0.41	0.9595549
Olaparib	0.97	3.20	2.06	1.14	0.9905677
Olmutinib	0.88	0.19	0.32	-0.12	0.9595549
Omacetaxine	0.16	23.53	19.47	4.06	0.5749379
Omaveloxolone	0.46	15.28	13.98	1.29	0.7629368
Omipalisib	0.39	16.97	18.20	-1.23	0.7563527
Onalespib	0.65	9.64	8.78	0.85	0.8455843
Oprozomib	0.37	15.86	13.47	2.39	0.7497097
Orteronel	NaN	0.00	0.00	0.00	NaN
Osimertinib	0.79	4.97	5.27	-0.30	0.9156139
Oxaliplatin	0.11	10.10	7.22	2.88	0.5429712
PAC-1	0.21	2.99	1.98	1.00	0.6307466
PCI-34051	0.01	1.92	0.39	1.53	0.4713480
PD0325901	0.89	6.96	6.81	0.15	0.9595549
PF-00477736	0.03	11.12	7.55	3.57	0.4960204
PF-00562271	0.60	5.53	6.06	-0.53	0.8382060
PF-03758309	0.42	16.64	13.09	3.55	0.7563527
PF-04708671	0.73	1.46	1.05	0.41	0.8855636
PF-06463922	0.95	1.23	1.29	-0.06	0.9865162
PF-3845	0.23	1.56	0.54	1.03	0.6384922
PF-4800567	1.00	0.66	0.59	0.06	1.0000000
PF-670462	0.64	1.75	1.29	0.46	0.8455843
PF06650833	0.32	1.99	1.05	0.94	0.7046038
PFI-1	0.50	5.47	4.48	0.98	0.7684873
PH-797804	0.08	0.24	0.00	0.24	0.5220806
PHA 408	0.01	4.48	2.31	2.17	0.4828655
PIM-447	0.27	3.09	1.80	1.29	0.6646899
PS-1145	0.88	0.13	0.14	-0.01	0.9595549
PTC-209	0.78	4.89	4.89	0.00	0.9096508
Paclitaxel	0.08	16.26	10.44	5.81	0.5181296
Pacritinib	0.23	8.48	7.25	1.23	0.6384922
Palbociclib	0.10	2.70	1.01	1.69	0.5429712
Palomid-529	0.49	0.95	0.42	0.53	0.7675784
Panobinostat	0.01	13.82	10.07	3.75	0.4713480
Pazopanib	0.69	1.04	0.63	0.42	0.8665021
Peficitinb	0.92	0.14	0.01	0.13	0.9774233
Pemetrexed	0.37	3.34	1.76	1.58	0.7497097
Pentostatin	0.17	0.35	0.08	0.27	0.5829313
Perifosine	0.68	0.20	0.40	-0.20	0.8608913
Pevonedistat	0.59	9.31	8.06	1.25	0.8347758
Pexidartinib	0.18	1.18	0.60	0.58	0.5990458
Pictilisib	0.90	10.54	9.64	0.90	0.9667140
Pilocarpine	0.25	0.42	0.16	0.25	0.6400935
Pinometostat	NaN	0.00	0.00	0.00	NaN
Pirfenidone	0.39	2.39	0.00	2.39	0.7563527
Pixantrone	0.06	0.49	0.09	0.40	0.5109537
Plerixafor	0.51	0.03	0.02	0.01	0.7905405
Plicamycin	0.47	8.91	7.91	1.00	0.7629368
Pomalidomide	0.93	0.13	0.02	0.11	0.9774233
Ponatinib	0.04	4.84	2.83	2.01	0.4960204

Poziotinib	0.24	7.10	10.07	-2.97	0.6384922
Pracinostat	0.04	12.21	9.15	3.06	0.4960204
Pravastatin	0.68	0.20	0.02	0.18	0.8615785
Prednisolone	0.11	0.94	1.00	-0.06	0.5429712
Prexasertib	0.02	22.85	16.67	6.18	0.4828655
Quisinostat	0.02	10.97	7.55	3.42	0.4828655
Quizartinib	0.56	0.92	0.43	0.49	0.8266202
RGFP966	0.69	2.47	1.83	0.64	0.8665021
RO5126766	0.47	1.73	1.95	-0.23	0.7629368
RSL3	0.28	0.00	0.13	-0.13	0.6727284
Rabusertib	0.43	0.98	0.82	0.16	0.7563527
Radotinib	0.87	0.96	1.22	-0.27	0.9595549
Ralimetinib	0.13	3.40	2.64	0.76	0.5429712
Raloxifene	0.14	3.31	1.71	1.60	0.5429712
Raltitrexed	0.37	3.84	2.11	1.73	0.7497097
Ravoxertinib	0.26	5.12	3.60	1.52	0.6502002
Regorafenib	0.03	4.31	2.53	1.78	0.4960204
Resatorvid	0.24	0.12	0.00	0.12	0.6384922
Resiquimod	0.13	0.54	0.18	0.37	0.5429712
Resminostat	0.03	8.24	5.67	2.57	0.4960204
Ribociclib	0.13	1.58	0.09	1.49	0.5429712
Ridaforolimus	0.04	9.00	5.07	3.93	0.4960204
Rigosertib	0.06	18.33	11.84	6.49	0.5027989
Ripasudil	0.93	0.12	0.06	0.05	0.9774233
Rociletinib	0.57	6.13	6.81	-0.68	0.8333432
Rocilinostat	0.07	5.02	3.60	1.42	0.5181296
Romidepsin	0.20	23.83	22.67	1.16	0.6254250
Roxadustat	0.21	0.28	0.12	0.15	0.6384922
Ruboxistaurin	0.04	6.64	4.40	2.24	0.4960204
Rucaparib	0.20	0.88	0.10	0.78	0.6254250
Ruxolitinib	0.34	0.48	1.13	-0.65	0.7173344
S-63845	0.40	1.79	0.90	0.90	0.7563527
SAR405838	0.83	3.42	2.15	1.27	0.9358950
SB 743921	0.02	4.78	2.03	2.74	0.4828655
SCH772984	0.86	9.23	8.92	0.30	0.9530552
SGC-CBP30	0.12	5.05	3.30	1.75	0.5429712
SGC0946	0.54	0.69	0.39	0.30	0.8131087
SGI-1776	0.27	6.71	5.72	0.99	0.6646899
SH-4-54	0.71	7.21	7.34	-0.13	0.8693846
SN-38	0.48	24.07	20.76	3.31	0.7629368
SNS-032	0.92	17.78	17.89	-0.11	0.9774233
Sabutoclax	0.77	10.47	10.40	0.07	0.9096508
Salinomycin	0.38	15.86	13.18	2.68	0.7563527
Sapanisertib	0.35	13.72	12.22	1.50	0.7295473
Sapitinib	0.02	2.47	5.40	-2.94	0.4828655
Saracatinib	0.17	6.01	7.61	-1.60	0.5752278
Saridegib	0.15	0.87	0.28	0.59	0.5607478
Seliciclib	0.57	1.68	1.05	0.62	0.8333432
Selinexor	0.78	16.54	15.99	0.54	0.9096508
Selonsertib	0.93	0.13	0.01	0.12	0.9774233
Selumetinib	0.40	5.77	5.22	0.56	0.7563527
Senexin B	0.04	0.80	1.68	-0.88	0.4960204
Sepantronium bromide	0.13	35.34	33.23	2.11	0.5429712
Serabelisib	0.16	1.88	2.96	-1.08	0.5663914

Silmitasertib	0.49	6.00	6.39	-0.39	0.7684873
Simvastatin	0.61	0.82	0.61	0.21	0.8389581
Sirolimus	0.19	4.59	3.05	1.54	0.6012754
Sitravatinib	0.82	1.70	1.18	0.52	0.9338992
Sonidegib	0.82	1.43	0.85	0.58	0.9338992
Sonolisib	0.31	0.46	0.11	0.35	0.6961959
Sorafenib	0.59	0.06	0.09	-0.03	0.8347758
Sotrastaurin	0.24	0.35	0.20	0.15	0.6384922
Spebrutinib	0.27	1.06	0.48	0.57	0.6671774
StemRegenin 1	0.92	2.47	2.26	0.21	0.9774233
Sunitinib	0.49	0.79	0.27	0.52	0.7675784
TAK-285	0.46	0.81	1.51	-0.70	0.7629368
TAK-530	0.23	0.86	0.63	0.23	0.6384922
TAK-901	0.50	4.54	3.40	1.14	0.7684873
TEW-7197	0.22	0.44	1.00	-0.56	0.6384922
TG100-115	0.83	0.40	0.14	0.26	0.9358950
TGR-1202	0.58	0.19	0.03	0.16	0.8347758
TGX-221	0.64	2.58	1.77	0.81	0.8455843
TH588	0.64	2.96	1.61	1.35	0.8455843
THZ2	0.24	18.86	17.59	1.28	0.6384922
TIC10	0.02	5.68	1.96	3.72	0.4828655
TRAM-34	NaN	0.00	0.00	0.00	NaN
Tacedinaline	0.98	0.46	0.28	0.18	0.9944730
Tacrolimus	0.05	0.94	0.24	0.70	0.4989832
Taladegib	0.15	0.63	0.20	0.43	0.5607478
Talazoparib	0.45	5.12	2.99	2.13	0.7629368
Talmapimod	0.03	0.56	0.00	0.56	0.4960204
Tamatinib	0.06	2.59	3.53	-0.94	0.5027989
Tamoxifen	0.42	2.82	2.47	0.34	0.7563527
Tandutinib	NaN	0.00	0.00	0.00	NaN
Tanzisertib	0.76	0.77	0.65	0.12	0.9093617
Tarenflurbil	0.87	0.54	1.12	-0.58	0.9595549
Taselisib	0.36	5.32	6.55	-1.23	0.7497097
Tasquinimod	0.52	1.09	0.72	0.38	0.7929268
Tazemetostat	0.43	0.04	0.01	0.03	0.7563527
Telatinib	0.43	0.03	0.00	0.03	0.7563527
Temozolomide	0.41	0.29	0.00	0.29	0.7563527
Temsirolimus	0.04	8.22	4.10	4.12	0.4960204
Teniposide	0.28	0.00	0.01	-0.01	0.6727284
Tepotinib	0.48	0.14	0.49	-0.35	0.7629368
Tesevatinib	0.19	4.44	6.70	-2.26	0.6052212
Thalidomide	0.19	1.55	0.09	1.46	0.6052212
Thioguanine	0.04	6.72	2.25	4.47	0.4960204
Tideglusib	0.58	0.13	0.02	0.10	0.8347758
Tipifarnib	0.37	6.95	4.42	2.53	0.7497097
Tirabrutinib	0.19	0.66	0.70	-0.05	0.6052212
Tivantinib	0.15	2.84	1.76	1.08	0.5663914
Tivozanib	0.12	3.47	2.35	1.12	0.5429712
Tofacitinib	0.47	0.26	0.10	0.16	0.7629368
Topotecan	0.23	11.92	9.40	2.53	0.6384922
Toremifene	0.63	4.22	3.93	0.29	0.8454114
Tosedostat	0.64	6.11	7.30	-1.19	0.8455843
Tozasertib	0.33	4.91	3.24	1.68	0.7046038
Trametinib	0.77	8.49	7.85	0.64	0.9096508

Tretinoin	0.93	0.13	0.52	-0.39	0.9774233
Triapine	0.16	2.58	1.31	1.27	0.5663914
Triciribine	0.48	13.56	12.50	1.06	0.7629368
Trifluridine	0.07	4.22	0.50	3.73	0.5181296
Tubacin	0.01	4.32	1.41	2.91	0.4713480
Tubastatin A	0.04	1.51	0.18	1.33	0.4989832
Tucatinib	0.39	0.01	0.03	-0.02	0.7563527
Tucidinostat	0.14	5.23	3.57	1.65	0.5536097
UCN-01	0.70	16.96	17.55	-0.60	0.8686009
UM729	0.09	1.29	0.47	0.82	0.5264730
UNC0638	0.08	3.23	2.05	1.18	0.5220806
UNC0642	0.04	1.87	1.06	0.81	0.4960204
UNC1215	0.43	0.01	0.00	0.01	0.7563527
UNC2881	0.83	0.74	0.43	0.31	0.9358950
URB597	0.59	0.32	0.14	0.19	0.8347758
Ulixertinib	0.31	7.44	6.37	1.07	0.6961959
Upadacitinib	0.07	0.16	0.00	0.16	0.5181296
Uprosertib	0.98	3.92	3.18	0.74	0.9944730
VE-821	0.37	1.26	0.61	0.65	0.7497097
VER 155008	0.77	4.06	3.99	0.07	0.9096508
VGX-1027	0.73	0.04	0.02	0.03	0.8829666
VLX1570	0.01	21.33	16.66	4.67	0.4713480
VS-4718	0.98	6.58	7.07	-0.49	0.9944730
Valproic acid	NaN	0.00	0.00	0.00	NaN
Valrubicin	0.46	7.69	5.87	1.82	0.7629368
Vandetanib	0.44	1.32	1.56	-0.24	0.7599729
Varespladib	0.40	0.41	0.35	0.06	0.7563527
Varlitinib	0.41	2.03	2.90	-0.88	0.7563527
Vatalanib	0.89	0.03	0.02	0.01	0.9595549
Veliparib	0.89	1.25	1.58	-0.33	0.9595549
Vemurafenib	0.37	0.86	1.28	-0.43	0.7497097
Venetoclax	0.47	0.02	0.05	-0.03	0.7629368
Verdinexor	0.72	8.96	8.38	0.58	0.8827228
Vesatolimod	0.56	0.14	0.32	-0.18	0.8266202
Vidofludimus	1.00	0.42	0.23	0.19	1.0000000
Vinblastine	0.53	9.25	7.20	2.06	0.7944291
Vincristine	0.25	9.85	5.98	3.87	0.6384922
Vinflunine	0.51	2.77	1.58	1.20	0.7905405
Vinorelbine	0.17	19.54	12.69	6.85	0.5901206
Vismodegib	0.65	0.27	0.31	-0.04	0.8455843
Vistusertib	0.55	14.55	13.22	1.33	0.8172852
Volasertib	0.09	8.07	4.13	3.94	0.5220806
Vorinostat	0.22	8.33	6.43	1.90	0.6384922
WEHI-539	0.72	0.53	0.93	-0.40	0.8778278
XAV-939	0.41	1.33	1.08	0.26	0.7563527
ZSTK474	0.81	10.81	9.88	0.93	0.9289648

Supplemental Data 4

DSS comparison between all PDCs derived from ascites (A) or tissue (TT), based on drug subclasses

subclass	p.value	mean_ascites	mean_tissue	logFC	p.adj
AKT inhibitor	0.39	4.72	3.79	0.93	0.6872510
ALK inhibitor	0.87	2.69	2.58	0.11	0.8888971
Alkylating agent	0.43	0.84	0.41	0.44	0.7020970
Antiandrogen	0.02	0.41	0.19	0.22	0.2200095
Antiestrogen	0.12	2.68	2.18	0.49	0.3479416
Antimetabolite	0.02	5.92	2.95	2.97	0.2200095
Aromatase inhibitor	0.47	0.16	0.07	0.09	0.7020970
Aurora kinase inhibitors	0.26	5.15	3.45	1.69	0.4946228
BCR-ABL inhibitor	0.59	2.50	2.76	-0.26	0.7020970
BET inhibitor	0.28	9.29	6.87	2.42	0.5062874
BH3 mimetic	0.58	5.68	6.17	-0.50	0.7020970
BTK inhibitor	0.76	0.85	0.89	-0.04	0.8286396
CDK inhibitor	0.54	9.19	8.39	0.80	0.7020970
DNMT inhibitor	0.02	3.50	1.86	1.64	0.2200095
EGFR or ERBB2 inhibitor	0.16	3.85	5.14	-1.29	0.4016318
FGFR inhibitor	0.18	2.08	1.23	0.85	0.4190417
FLT3 inhibitor	0.96	1.76	1.66	0.10	0.9567173
Glucocorticoid	0.06	0.50	0.84	-0.34	0.2438848
HDAC inhibitor	0.04	8.86	7.08	1.78	0.2304531
HMGCR inhibitor	0.26	0.79	0.77	0.02	0.4946228
HMT inhibitor	0.06	0.24	0.10	0.14	0.2438848
HSP inhibitor	0.59	10.55	9.61	0.94	0.7020970
Hedgehog inhibitor	0.51	0.67	0.42	0.24	0.7020970
IDH1/IDH2 inhibitor	0.12	0.22	0.10	0.12	0.3479416
JAK inhibitor	0.48	2.09	1.66	0.43	0.7020970
Kinesin inhibitor	0.03	10.50	5.47	5.03	0.2200095
MEK1/2 inhibitor	0.79	5.77	5.28	0.50	0.8375438
MET inhibitor	0.51	0.65	0.50	0.14	0.7020970
Mitotic inhibitor	0.14	11.43	7.44	3.99	0.3690920
Multi kinase inhibitor	0.13	2.20	1.64	0.56	0.3507668
Other	0.04	5.14	4.11	1.03	0.2438651
Other antineoplastic	0.21	9.17	7.20	1.97	0.4522256
Other apoptotic modulator	0.69	10.43	10.22	0.21	0.7862732
Other differentiating/epigenetic modifier	0.10	2.45	1.97	0.48	0.3143516
Other hormone therapy	0.57	0.80	0.27	0.54	0.7020970
Other immunomodulatory	0.08	1.18	0.66	0.52	0.2673560
Other kinase inhibitor	0.20	4.12	3.28	0.84	0.4439292
Other metabolic modifier	0.03	5.47	3.58	1.89	0.2200095
PARP inhibitor	0.53	2.59	1.59	1.00	0.7020970
PI3K or MTOR inhibitor	0.76	7.14	6.62	0.53	0.8286396
Protease/proteasome inhibitor	0.69	16.47	15.65	0.82	0.7862732
RAF inhibitor	0.49	2.84	2.27	0.57	0.7020970
Rapalog	0.03	7.14	3.97	3.17	0.2200095
Retinoic acid receptor ligand	0.46	0.85	0.80	0.05	0.7020970
TLRs ligand	0.02	0.80	0.22	0.58	0.2200095
Topoisomerase inhibitor	0.24	7.78	5.54	2.24	0.4897961
VEGFR inhibitor	0.07	1.53	0.76	0.78	0.2556658
p38MAPK inhibitor	0.05	1.36	0.71	0.66	0.2438848
p53 pathway modulator	0.81	3.36	2.20	1.16	0.8446375

Supplemental Table 5

Epithelial differentiation genes	Mesenchyal differentiation genes	Cancer stem cells markers	gamma-secretase complex containing proteins
CDH1	ITGA5	SOX2	PSEN1
ST14	SYDE1	CD44	APH1A
GRHL2	S100A14	KLF4	APH1B
GRHL1	VIM	ALDH1A1	NCSTN
CDH3	CD99L2	PROM1	
LLGL2		ALDH1A3	
EPCAM			
DDR1			
PRSS8			
MISP			
MAL2			
AP1M2			
LSR			
PKP2			
MAP7			
SLC44A2			
RAB25			
MYO5B			
ARHGEF5			
JUP			
TC2N			
SPINT1			
HOOK1			

Supplemental Table 6

ZIP scores A133, Carboplatin, GSI

Model	drug1	drug2	ZIP_synergy	A-1331852 response group
OVSAHO	A-1331852	MK-0752	20.246	resistant
OVSAHO	A-1331852	Nirogacestat	21.195	resistant
OVSAHO	Carboplatin	MK-0752	-0.483	resistant
OVSAHO	Carboplatin	Nirogacestat	-3.320	resistant
OVSAHO	Carboplatin	A-1331852	-1.359	resistant
Gem061-A	A-1331852	MK-0752	6.405	resistant
Gem061-A	A-1331852	Nirogacestat	8.377	resistant
Gem061-A	Carboplatin	MK-0752	2.023	resistant
Gem061-A	Carboplatin	Nirogacestat	2.222	resistant
Gem061-A	Carboplatin	A-1331852	3.258	resistant
OV039-PDF	A-1331852	MK-0752	1.011	PDF
OV039-PDF	A-1331852	Nirogacestat	1.785	PDF
OV039-PDF	Carboplatin	MK-0752	1.004	PDF
OV039-PDF	Carboplatin	Nirogacestat	0.860	PDF
OV039-PDF	Carboplatin	A-1331852	1.111	PDF
OV031-PDF	A-1331852	MK-0752	-3.482	PDF
OV031-PDF	A-1331852	Nirogacestat	-5.961	PDF
OV031-PDF	Carboplatin	MK-0752	1.591	PDF
OV031-PDF	Carboplatin	Nirogacestat	1.248	PDF
OV031-PDF	Carboplatin	A-1331852	2.187	PDF
OvcaR4	A-1331852	MK-0752	-3.399	resistant
OvcaR4	A-1331852	Nirogacestat	-2.878	resistant
OvcaR4	Carboplatin	MK-0752	3.578	resistant
OvcaR4	Carboplatin	Nirogacestat	0.637	resistant
OvcaR4	Carboplatin	A-1331852	3.850	resistant
KURAMOCHI	A-1331852	MK-0752	3.700	sensitive
KURAMOCHI	A-1331852	Nirogacestat	10.113	sensitive
KURAMOCHI	Carboplatin	MK-0752	2.041	sensitive
KURAMOCHI	Carboplatin	Nirogacestat	1.418	sensitive
KURAMOCHI	Carboplatin	A-1331852	4.521	sensitive
OV022-T2	A-1331852	MK-0752	7.1614322	sensitive
OV022-T2	A-1331852	Nirogacestat	9.9306245	sensitive
OV022-T2	Carboplatin	MK-0752	0.4290571	sensitive
OV022-T2	Carboplatin	Nirogacestat	0.6221957	sensitive
OV022-T2	Carboplatin	A-1331852	2.9736026	sensitive
OV030-R	A-1331852	MK-0752	5.006	resistat
OV030-R	A-1331852	Nirogacestat	6.763	resistat
OV030-R	Carboplatin	MK0752	2.791	resistat
OV030-R	Carboplatin	Nirogacestat	0.427	resistat
OV030-R	Carboplatin	A-1331852	4.941	resistat
OV030-S	A-1331852	MK-0752	0.236	sensitive
OV030-S	A-1331852	Nirogacestat	-0.076	sensitive
OV030-S	Carboplatin	MK-0752	2.104	sensitive
OV030-S	Carboplatin	Nirogacestat	-2.728	sensitive
OV030-S	Carboplatin	A-1331852	3.541	sensitive

ZIP scores CBK03, Carboplatin, Nirogacestat

Model	drug1	drug2	ZIP_synergy
OV030-S	DT2216	Nirogacestat	2.313527824
OV030-S	DT2216	Carboplatin	10.66365933
OV030-R	DT2216	Nirogacestat	6.411799386
OV030-R	DT2216	Carboplatin	15.86327844
OV039-PDF	DT2216	Nirogacestat	-1.608453191
OV039-PDF	DT2216	Carboplatin	6.900148433

Supplemental Table 7

DRUG_NAME	Gem061-A	KURAMOCHI	OV022-T2	OV024-A	OV0031-PDF	OV039-PDF	OVCAR4	OVSAHO
A-1331852	100.80	86.52	64.88	97.57	95.04	99.53	90.26	90.52
A-1331852+MK-0752 0.1 nM	99.74	85.25	61.13	92.36	87.21	103.59	85.16	97.90
A-1331852+MK-0752 1 nM	99.03	82.17	61.48	93.86	90.00	102.44	83.62	95.72
A-1331852+MK-0752 10 nM	102.40	83.88	60.12	95.84	88.86	102.31	86.83	101.47
A-1331852+MK-0752 100 nM	97.84	81.98	57.96	94.65	87.69	103.73	81.51	92.43
A-1331852+MK-0752 1000 nM	96.85	80.89	62.98	94.08	85.94	99.76	84.65	96.31
A-1331852+MK-0752 10000 nM	96.48	70.51	45.51	91.62	75.04	96.37	76.45	90.00
A-1331852+MK-0752 20000 nM	95.41	67.73	41.88	89.20	72.18	95.02	74.09	84.30
A-1331852+Nirogacestat 0.1 nM	100.10	82.56	64.52	92.86	90.49	103.32	84.70	94.85
A-1331852+Nirogacestat 1 nM	103.08	81.46	63.73	95.30	95.45	106.13	88.07	101.40
A-1331852+Nirogacestat 10 nM	99.89	83.14	60.96	92.78	87.59	105.80	84.40	94.99
A-1331852+Nirogacestat 100 nM	96.66	79.65	59.03	95.60	86.05	101.99	82.09	95.25
A-1331852+Nirogacestat 1000 nM	99.12	74.05	52.68	93.87	84.18	102.98	82.84	92.14
A-1331852+Nirogacestat 10000 nM	81.27	50.10	30.30	81.34	75.75	99.21	58.23	67.16
A-1331852+Nirogacestat 20000 nM	51.46	29.78	10.92	59.80	62.10	91.91	44.81	48.29
Carboplatin	98.28	86.67	88.05	89.71	96.49	94.60	85.17	82.56
Carboplatin+A-1331852 0.1 nM	97.99	88.76	85.02	91.05	98.01	92.31	83.42	85.00
Carboplatin+A-1331852 1 nM	97.46	85.85	82.93	88.67	96.55	92.05	80.43	78.47
Carboplatin+A-1331852 10 nM	93.63	78.00	64.48	86.95	96.40	92.47	75.48	74.28
Carboplatin+A-1331852 100 nM	92.19	64.56	28.36	83.72	90.84	91.05	73.80	62.63
Carboplatin+A-1331852 1000 nM	85.59	37.72	10.30	79.57	85.37	86.72	65.61	46.11
Carboplatin+MK-0752 0.1 nM	100.16	86.72	88.04	89.62	96.74	93.33	81.73	81.73
Carboplatin+MK-0752 1 nM	96.12	83.86	81.65	89.03	97.22	91.15	79.73	80.12
Carboplatin+MK-0752 10 nM	99.68	87.72	86.03	89.79	97.61	92.98	84.26	86.70
Carboplatin+MK-0752 100 nM	96.44	84.54	88.39	90.60	95.00	90.91	78.84	81.47
Carboplatin+MK-0752 1000 nM	94.05	84.04	84.28	92.10	91.96	90.86	80.70	83.36
Carboplatin+MK-0752 10000 nM	95.71	82.07	81.96	87.89	78.81	86.36	77.84	81.26
Carboplatin+MK-0752 20000 nM	94.31	79.00	81.19	85.43	78.82	83.80	76.01	78.83
Carboplatin+Nirogacestat 0.1 nM	96.75	84.90	86.04	91.05	97.86	94.08	82.30	83.75
Carboplatin+Nirogacestat 1 nM	99.35	87.63	85.47	91.93	98.13	95.79	83.90	87.28
Carboplatin+Nirogacestat 10 nM	98.27	86.96	89.55	90.36	91.95	93.15	81.92	80.53
Carboplatin+Nirogacestat 100 nM	95.07	84.20	84.78	93.41	89.78	91.39	80.33	83.90
Carboplatin+Nirogacestat 1000 nM	99.58	83.84	84.72	92.83	87.72	93.15	78.46	84.49
Carboplatin+Nirogacestat 10000 nM	86.13	79.09	78.56	84.07	82.69	89.74	67.20	78.82
Carboplatin+Nirogacestat 20000 nM	64.32	64.76	59.96	68.36	73.60	87.12	59.60	70.43
MK-0752	101.65	97.82	97.98	100.80	91.95	97.86	95.08	108.55
Nirogacestat	97.37	94.21	93.30	95.10	88.70	99.92	87.70	102.40
DMSO	98.20	97.09	94.79	97.01	97.74	95.16	94.06	100.70

Supplemental Table 8

Model_name	Passage at drug testing & innitial characterization
Gem017-T	P7
Gem034-T1	P6
Gem046-T	P5
Gem061-A	P8
Gem062-T	P8
Gem096-A	P4
OV015-T	NA
OV022-A	NA
OV022-T1	NA
OV022-T2	NA
OV024-A	P7
OV026-PDF	P4
OV026-T	P9
OV027-PDF	P9
OV027-PDF-R	P9
OV027-T	P8
OV030-A2	P8
OV030-A3	P8
OV030-PDF	P7
OV030-PDF-R	P10
OV030-R	P8
OV030-S	P8
OV031-PDF	P8
OV031-PDF-R	P12
OV032-PDF	P6
OV035-PDF	P5
OV038-PDF	P5
OV039-PDF	P5
OV039-PDF-R	P9
OV042-A	P8
OV042-PDF	P5
OV042-T	P8
OV042-T2	P8
OV044-PDF	P5
OV048-PDF	P9
OV048-PDF-R	P8
OV048-PDF.T	P9
OV050-PDF	P5
OV051-PDF	P5

Supplemental Table 9

Assay	Antibody	Manufacturer	Cat. No	Dilution
Immunofluorescent staining	aSMA-Alexa647	Abcam	ab32575	1:100
	ALDH1A1	Abcam	ab52492	1:125
	CD44	Invitrogen	MA5-13890	1:100
	CK8/18	Agilent/Dako	M3652	1:300
	FSP1	Agilent/Dako	A5114	1:100
	HES1	Abcam	ab108937	1:250
	MUC16	Invitrogen	MA5-11202	1:100
	PAX8	Proteintech	10336-1-AP	1:100
	Vimentin	Invitrogen	MA5-11883	1:300
	Donkey anti-Mouse Secondary Antibody, Alexa Fluor™ 488	Invitrogen	A21202	1:2000
	Donkey anti-Mouse Secondary Antibody, Alexa Fluor™ 568	Invitrogen	A10037	1:2000
	Donkey anti-Rabbit Secondary Antibody, Alexa Fluor™ 488	Invitrogen	A21206	1:2000
	Donkey anti-Rabbit Secondary Antibody, Alexa Fluor™ 568	Invitrogen	A10042	1:2000
Western Blot	Bcl-xL	Cell Signaling	#2764	1:1000
	Vinculin	Invitrogen	700062	1:1000
	beta-tubulin	Cell Signaling	#86298	1:1000
	Donkey anti-Rabbit IgG (H+L) Highly Cross-Adsorbed Secondary Antibody, HRP	Invitrogen	A16035	1:10000
	Donkey anti-Mouse IgG (H+L) Highly Cross-Adsorbed Secondary Antibody, HRP	Invitrogen	A16017	1:10000

Supplemental Table 10
Drug screen against 528 drugs

Type	PatientID	Model	#plate	Z-prime
	OV015	Tissue PDC	1	0.59
		Tissue PDC	2	0.71
	OV022	Ascites PDC	1	0.81
		Ascites PDC	2	0.8
		Ascites PDC	3	0.8
		Ascites PDC	4	0.88
		Ascites PDC	5	0.85
		Ascites PDC	6	0.79
		Ascites PDC	7	0.82
		Ascites PDC	8	0.82
	OV022	Tissue DMEM PDC	1	0.83
		Tissue DMEM PDC	2	0.81
		Tissue DMEM PDC	3	0.87
		Tissue DMEM PDC	4	0.84
		Tissue DMEM PDC	5	0.82
		Tissue DMEM PDC	6	0.84
		Tissue DMEM PDC	7	0.84
		Tissue DMEM PDC	8	0.71
	OV022	Tissue Rockit PDC	1	0.79
		Tissue Rockit PDC	2	0.8
		Tissue Rockit PDC	3	0.18
		Tissue Rockit PDC	4	0.85
		Tissue Rockit PDC	5	0.85
		Tissue Rockit PDC	6	0.88
		Tissue Rockit PDC	7	0.88
		Tissue Rockit PDC	8	0.77
	OV024	Ascites PDC	1	0.78
		Ascites PDC	2	0.81
		Ascites PDC	3	0.86
		Ascites PDC	4	0.78
		Ascites PDC	5	0.84
		Ascites PDC	6	0.83
		Ascites PDC	7	0.83
		Ascites PDC	8	0.8
	OvCa026	Tissue PDC	1	0.85
		Tissue PDC	2	0.81
		Tissue PDC	3	0.7
		Tissue PDC	4	0.6
		Tissue PDC	5	0.81
		Tissue PDC	6	0.83
		Tissue PDC	7	0.78
		Tissue PDC	8	0.78
	OvCa026	Tissue PDF in Fibroblasts	1	0.72
		Tissue PDF in Fibroblasts	2	0.73
		Tissue PDF in Fibroblasts	3	0.74
		Tissue PDF in Fibroblasts	4	0.79
		Tissue PDF in Fibroblasts	5	0.72
		Tissue PDF in Fibroblasts	6	0.75
		Tissue PDF in Fibroblasts	7	0.52
		Tissue PDF in Fibroblasts	8	0.7
	OV027	Tissue PDC	1	0.77
		Tissue PDC	2	0.84
		Tissue PDC	3	0.84
		Tissue PDC	4	0.85
		Tissue PDC	5	0.76

	Tissue PDC	6	0.81
	Tissue PDC	7	0.83
	Tissue PDC	8	0.85
OV027	Tissue PDF in Fibroblasts	1	0.73
	Tissue PDF in Fibroblasts	2	0.73
	Tissue PDF in Fibroblasts	3	0.79
	Tissue PDF in Fibroblasts	4	0.8
	Tissue PDF in Fibroblasts	5	0.73
	Tissue PDF in Fibroblasts	6	0.77
	Tissue PDF in Fibroblasts	7	0.73
	Tissue PDF in Fibroblasts	8	0.68
OV030	Ascites resistant to Bcl-xL inhibitors	1	0.7
	Ascites resistant to Bcl-xL inhibitors	2	0.79
	Ascites resistant to Bcl-xL inhibitors	3	0.81
	Ascites resistant to Bcl-xL inhibitors	4	0.7
	Ascites resistant to Bcl-xL inhibitors	5	0.69
	Ascites resistant to Bcl-xL inhibitors	6	0.64
	Ascites resistant to Bcl-xL inhibitors	7	0.74
	Ascites resistant to Bcl-xL inhibitors	8	0.74
OV030	Ascites model A2	1	0.82
	Ascites model A2	2	0.79
	Ascites model A2	3	0.76
	Ascites model A2	4	0.79
	Ascites model A2	5	0.79
	Ascites model A2	6	0.77
	Ascites model A2	7	0.79
	Ascites model A2	8	0.79
OV030	Ascites model A3	1	0.77
	Ascites model A3	2	0.79
	Ascites model A3	3	0.76
	Ascites model A3	4	0.56
	Ascites model A3	5	0.68
	Ascites model A3	6	0.74
	Ascites model A3	7	0.82
	Ascites model A3	8	0.62
OV030	Ascites sensitive to Bcl-xL inhibitors	1	0.7
	Ascites sensitive to Bcl-xL inhibitors	2	0.8
	Ascites sensitive to Bcl-xL inhibitors	3	0.63
	Ascites sensitive to Bcl-xL inhibitors	4	0.79
	Ascites sensitive to Bcl-xL inhibitors	5	0.82
	Ascites sensitive to Bcl-xL inhibitors	6	0.78
	Ascites sensitive to Bcl-xL inhibitors	7	0.66
	Ascites sensitive to Bcl-xL inhibitors	8	0.74
OV030	Tissue PDF in Fibroblasts	1	0.79
	Tissue PDF in Fibroblasts	2	0.87
	Tissue PDF in Fibroblasts	3	0.82
	Tissue PDF in Fibroblasts	4	0.77
	Tissue PDF in Fibroblasts	5	0.83
	Tissue PDF in Fibroblasts	6	0.78
	Tissue PDF in Fibroblasts	7	0.78
	Tissue PDF in Fibroblasts	8	0.81
OV031	Tissue PDF in Fibroblasts	1	0.81
	Tissue PDF in Fibroblasts	2	0.8
	Tissue PDF in Fibroblasts	3	0.72
	Tissue PDF in Fibroblasts	4	0.82
	Tissue PDF in Fibroblasts	5	0.79
	Tissue PDF in Fibroblasts	6	0.81
	Tissue PDF in Fibroblasts	7	0.7
	Tissue PDF in Fibroblasts	8	0.74

PDM

OV032	Tissue PDF in Fibroblasts	1	0.79
	Tissue PDF in Fibroblasts	2	0.84
	Tissue PDF in Fibroblasts	3	0.79
	Tissue PDF in Fibroblasts	4	0.75
	Tissue PDF in Fibroblasts	5	0.84
	Tissue PDF in Fibroblasts	6	0.82
	Tissue PDF in Fibroblasts	7	0.83
	Tissue PDF in Fibroblasts	8	0.82
OV035	Tissue PDF (from Bpart) in Fibroblasts	1	0.83
	Tissue PDF (from Bpart) in Fibroblasts	2	0.87
	Tissue PDF (from Bpart) in Fibroblasts	3	0.83
	Tissue PDF (from Bpart) in Fibroblasts	4	0.87
	Tissue PDF (from Bpart) in Fibroblasts	5	0.87
	Tissue PDF (from Bpart) in Fibroblasts	6	0.84
	Tissue PDF (from Bpart) in Fibroblasts	7	0.8
	Tissue PDF (from Bpart) in Fibroblasts	8	0.85
OV035	Tissue PDF (from Apart) in Fibroblasts	1	0.82
	Tissue PDF (from Apart) in Fibroblasts	2	0.78
	Tissue PDF (from Apart) in Fibroblasts	3	0.72
	Tissue PDF (from Apart) in Fibroblasts	4	0.71
	Tissue PDF (from Apart) in Fibroblasts	5	0.77
	Tissue PDF (from Apart) in Fibroblasts	6	0.82
	Tissue PDF (from Apart) in Fibroblasts	7	0.75
	Tissue PDF (from Apart) in Fibroblasts	8	0.75
OV038	Tissue PDF (from No2) in Fibroblasts	1	0.74
	Tissue PDF (from No2) in Fibroblasts	2	0.89
	Tissue PDF (from No2) in Fibroblasts	3	0.84
	Tissue PDF (from No2) in Fibroblasts	4	0.86
	Tissue PDF (from No2) in Fibroblasts	5	0.74
	Tissue PDF (from No2) in Fibroblasts	6	0.79
	Tissue PDF (from No2) in Fibroblasts	7	0.79
	Tissue PDF (from No2) in Fibroblasts	8	0.82
OV038	Tissue PDF (from No1) in Fibroblasts	1	0.84
	Tissue PDF (from No1) in Fibroblasts	2	0.8
	Tissue PDF (from No1) in Fibroblasts	3	0.71
	Tissue PDF (from No1) in Fibroblasts	4	-0.15
	Tissue PDF (from No1) in Fibroblasts	5	0.75
	Tissue PDF (from No1) in Fibroblasts	6	0.79
	Tissue PDF (from No1) in Fibroblasts	7	0.8
	Tissue PDF (from No1) in Fibroblasts	8	0.78
OV038	Tissue PDF (from Adex) in Fibroblasts	1	0.82
	Tissue PDF (from Adex) in Fibroblasts	2	0.85
	Tissue PDF (from Adex) in Fibroblasts	3	0.78
	Tissue PDF (from Adex) in Fibroblasts	4	0.83
	Tissue PDF (from Adex) in Fibroblasts	5	0.84
	Tissue PDF (from Adex) in Fibroblasts	6	0.81
	Tissue PDF (from Adex) in Fibroblasts	7	0.8
	Tissue PDF (from Adex) in Fibroblasts	8	0.83
OV039	Tissue PDF in Fibroblasts	1	0.86
	Tissue PDF in Fibroblasts	2	0.87
	Tissue PDF in Fibroblasts	3	0.84
	Tissue PDF in Fibroblasts	4	0.85
	Tissue PDF in Fibroblasts	5	0.85
	Tissue PDF in Fibroblasts	6	0.85
	Tissue PDF in Fibroblasts	7	0.88
	Tissue PDF in Fibroblasts	8	0.79
OV042	Tissue PDC	1	0.82
	Tissue PDC	2	0.87
	Tissue PDC	3	0.88

	Tissue PDC	4	0.8
	Tissue PDC	5	0.78
	Tissue PDC	6	0.87
	Tissue PDC	7	0.83
	Tissue PDC	8	0.64
OV042	Ascites PDC	1	0.84
	Ascites PDC	2	0.82
	Ascites PDC	3	0.81
	Ascites PDC	4	0.84
	Ascites PDC	5	0.85
	Ascites PDC	6	0.86
	Ascites PDC	7	0.78
	Ascites PDC	8	0.82
OV042	Tissue (SM) PDC	1	0.79
	Tissue (SM) PDC	2	0.79
	Tissue (SM) PDC	3	0.81
	Tissue (SM) PDC	4	0.81
	Tissue (SM) PDC	5	0.81
	Tissue (SM) PDC	6	0.79
	Tissue (SM) PDC	7	0.79
	Tissue (SM) PDC	8	0.77
OV042	Tissue PDF in Fibroblasts	1	0.9
	Tissue PDF in Fibroblasts	2	0.83
	Tissue PDF in Fibroblasts	3	0.85
	Tissue PDF in Fibroblasts	4	-0.15
	Tissue PDF in Fibroblasts	5	0.81
	Tissue PDF in Fibroblasts	6	0.86
	Tissue PDF in Fibroblasts	7	0.87
	Tissue PDF in Fibroblasts	8	0.86
OV044	Tissue PDF (from pelvis) in Fibroblasts	1	0.84
	Tissue PDF (from pelvis) in Fibroblasts	2	0.77
	Tissue PDF (from pelvis) in Fibroblasts	3	0.82
	Tissue PDF (from pelvis) in Fibroblasts	4	0.81
	Tissue PDF (from pelvis) in Fibroblasts	5	0.82
	Tissue PDF (from pelvis) in Fibroblasts	6	0.78
	Tissue PDF (from pelvis) in Fibroblasts	7	0.8
	Tissue PDF (from pelvis) in Fibroblasts	8	0.77
OV048	Tissue PDF in Fibroblasts	1	0.84
	Tissue PDF in Fibroblasts	2	0.79
	Tissue PDF in Fibroblasts	3	0.88
	Tissue PDF in Fibroblasts	4	0.89
	Tissue PDF in Fibroblasts	5	0.84
	Tissue PDF in Fibroblasts	6	0.82
	Tissue PDF in Fibroblasts	7	0.83
	Tissue PDF in Fibroblasts	8	0.84
OV048	Ascites PDF in Fibroblasts	1	0.74
	Ascites PDF in Fibroblasts	2	0.82
	Ascites PDF in Fibroblasts	3	0.78
	Ascites PDF in Fibroblasts	4	0.82
	Ascites PDF in Fibroblasts	5	0.8
	Ascites PDF in Fibroblasts	6	0.81
	Ascites PDF in Fibroblasts	7	0.74
	Ascites PDF in Fibroblasts	8	0.8
OV051	Tissue PDF in Fibroblasts	1	0.86
	Tissue PDF in Fibroblasts	2	0.87
	Tissue PDF in Fibroblasts	3	0.77
	Tissue PDF in Fibroblasts	4	0.84
	Tissue PDF in Fibroblasts	5	0.83
	Tissue PDF in Fibroblasts	6	0.85

	Tissue PDF in Fibroblasts	7	0.87
	Tissue PDF in Fibroblasts	8	0.8
OV050	Tissue PDF in Fibroblasts	1	0.76
	Tissue PDF in Fibroblasts	2	0.83
	Tissue PDF in Fibroblasts	3	0.82
	Tissue PDF in Fibroblasts	4	0.84
	Tissue PDF in Fibroblasts	5	0.72
	Tissue PDF in Fibroblasts	6	0.84
	Tissue PDF in Fibroblasts	7	0.81
	Tissue PDF in Fibroblasts	8	0.8
Gem017	Ascites PDC	1	0.85
	Ascites PDC	2	0.82
	Ascites PDC	3	0.83
Gem034	Tissue PDC	1	0.47
	Tissue PDC	2	0.89
	Tissue PDC	3	0.75
	Tissue PDC	4	0.91
	Tissue PDC	5	0.86
	Tissue PDC	6	0.36
	Tissue PDC	7	0.84
	Tissue PDC	8	0.83
Gem046	Ascites PDC	1	0.63
	Ascites PDC	2	0.78
	Ascites PDC	3	0.9
	Ascites PDC	4	0.88
	Ascites PDC	5	0.82
	Ascites PDC	6	0.81
	Ascites PDC	7	0.91
	Ascites PDC	8	0.79
Gem061	Ascites PDC	1	0.41
	Ascites PDC	2	0.86
	Ascites PDC	3	0.88
	Ascites PDC	4	0.91
	Ascites PDC	5	0.81
	Ascites PDC	6	0.92
	Ascites PDC	7	0.88
	Ascites PDC	8	0.88
Gem062	Tissue PDC	1	0.52
	Tissue PDC	2	0.87
	Tissue PDC	3	0.71
	Tissue PDC	4	0.86
	Tissue PDC	5	0.84
	Tissue PDC	6	0.84
	Tissue PDC	7	0.81
	Tissue PDC	8	0.79
Gem096	Ascites PDC	1	0.74
	Ascites PDC	2	0.71
	Ascites PDC	3	0.7
	Ascites PDC	4	0.71
	Ascites PDC	5	0.65
	Ascites PDC	6	0.65
	Ascites PDC	7	0.61
OV026	Tissue PDF in Rockit	1	0.78
	Tissue PDF in Rockit	2	0.61
	Tissue PDF in Rockit	3	0.71
	Tissue PDF in Rockit	4	0.71
	Tissue PDF in Rockit	5	0.71
	Tissue PDF in Rockit	6	0.64
	Tissue PDF in Rockit	7	0.7

		Tissue PDF in Rockit	8	0.79
	OV030	Tissue PDF in Rockit	1	0.87
		Tissue PDF in Rockit	2	0.9
		Tissue PDF in Rockit	3	0.88
		Tissue PDF in Rockit	4	0.88
		Tissue PDF in Rockit	5	0.89
		Tissue PDF in Rockit	6	0.9
		Tissue PDF in Rockit	7	0.9
		Tissue PDF in Rockit	8	0.96
	OV031	Tissue PDF in Rockit	1	0.85
		Tissue PDF in Rockit	2	0.67
		Tissue PDF in Rockit	3	0.81
		Tissue PDF in Rockit	4	0.85
		Tissue PDF in Rockit	5	0.76
		Tissue PDF in Rockit	6	0.85
		Tissue PDF in Rockit	7	0.52
		Tissue PDF in Rockit	8	0.69
	OV039	Tissue PDF in Rockit	1	0.86
		Tissue PDF in Rockit	2	0.92
		Tissue PDF in Rockit	3	0.78
		Tissue PDF in Rockit	4	0.9
		Tissue PDF in Rockit	5	0.87
		Tissue PDF in Rockit	6	0.84
		Tissue PDF in Rockit	7	0.84
		Tissue PDF in Rockit	8	0.88
	OV048	Tissue PDF in Rockit	1	0.84
		Tissue PDF in Rockit	2	0.91
		Tissue PDF in Rockit	3	0.88
		Tissue PDF in Rockit	4	0.9
		Tissue PDF in Rockit	5	0.89
		Tissue PDF in Rockit	6	0.86
		Tissue PDF in Rockit	7	0.83
		Tissue PDF in Rockit	8	0.88
BM	BM-1690	Bone marrow	1	0.75
		Bone marrow	2	0.79
		Bone marrow	3	0.81
		Bone marrow	4	0.82
		Bone marrow	5	0.82
		Bone marrow	6	0.83
		Bone marrow	7	0.81
		Bone marrow	8	0.76
	BM-1691	Bone marrow	1	0.78
		Bone marrow	2	0.84
		Bone marrow	3	0.73
		Bone marrow	4	0.84
		Bone marrow	5	0.67
		Bone marrow	6	0.8
		Bone marrow	7	0.72
		Bone marrow	8	0.84
		Bone marrow	1	0.86
	A-2780	CCLs	2	0.85
		CCLs	3	0.87
		CCLs	4	0.79
		CCLs	5	0.83
		CCLs	6	0.83
		CCLs	7	0.87
		CCLs	8	0.64
	EFO-21	CCLs	1	0.81
		CCLs	2	0.81

CCLs

	CCLs	3	0.77
	CCLs	4	0.83
	CCLs	5	0.82
	CCLs	6	0.84
	CCLs	7	0.88
	CCLs	8	0.88
KURAMOCHI	CCLs	1	0.86
	CCLs	2	0.89
	CCLs	3	0.82
	CCLs	4	0.84
	CCLs	5	0.88
	CCLs	6	0.85
	CCLs	7	0.81
	CCLs	8	0.93
MH	CCLs	1	0.78
	CCLs	2	0.78
	CCLs	3	0.79
	CCLs	4	0.76
	CCLs	5	0.69
	CCLs	6	0.77
	CCLs	7	0.81
	CCLs	8	0.81
NCI-ADR-RES	CCLs	1	0.53
	CCLs	2	0.54
	CCLs	3	0.63
	CCLs	4	0.31
	CCLs	5	0.42
	CCLs	6	0.62
	CCLs	7	0.57
	CCLs	8	0.5
OAW-28	CCLs	1	0.85
	CCLs	2	0.8
	CCLs	3	0.8
	CCLs	4	0.81
	CCLs	5	0.81
	CCLs	6	0.85
	CCLs	7	0.85
	CCLs	8	0.79
ONCODG-1	CCLs	1	0.84
	CCLs	2	0.74
	CCLs	3	0.83
	CCLs	4	0.82
	CCLs	5	0.81
	CCLs	6	0.82
	CCLs	7	0.88
	CCLs	8	0.84
OVCAR3	CCLs	1	0.84
	CCLs	2	0.88
	CCLs	3	0.81
	CCLs	4	0.76
	CCLs	5	0.74
	CCLs	6	0.72
	CCLs	7	0.61
	CCLs	8	0.7
OVCAR4	CCLs	1	0.77
	CCLs	2	0.8
	CCLs	3	0.8
	CCLs	4	0.81
	CCLs	5	0.79

		CCLs	6	0.82
		CCLs	7	0.8
		CCLs	8	0.65
	OVCAR8	CCLs	1	0.85
		CCLs	2	0.85
		CCLs	3	0.8
		CCLs	4	0.8
		CCLs	5	0.82
		CCLs	6	0.83
		CCLs	7	0.85
		CCLs	8	0.77
		CCLs	1	0.81
	OVSAHO	CCLs	2	0.81
		CCLs	3	0.84
		CCLs	4	0.82
		CCLs	5	0.86
		CCLs	6	0.8
		CCLs	7	0.77
		CCLs	8	0.79
	OVTOKO	CCLs	1	0.59
		CCLs	2	0.59
		CCLs	3	0.61
		CCLs	4	0.59
		CCLs	5	0.61
		CCLs	6	0.59
		CCLs	7	0.67
		CCLs	8	0.64
	TYKNU	CCLs	1	0.83
		CCLs	2	0.79
		CCLs	3	0.85
		CCLs	4	0.77
		CCLs	5	0.8
		CCLs	6	0.81
		CCLs	7	0.77
		CCLs	8	0.83
	CAOV3	CCLs	1	0.81
		CCLs	2	0.85
		CCLs	3	0.82
		CCLs	4	0.8
		CCLs	5	0.85
		CCLs	6	0.86
		CCLs	7	0.87
		CCLs	8	0.56
	COV362	CCLs	1	0.79
		CCLs	2	0.82
		CCLs	3	0.83
		CCLs	4	0.8
		CCLs	5	0.86
		CCLs	6	0.83
		CCLs	7	0.77
		CCLs	8	0.82

Supplemental Data 10

Validation drug screening

Model	corresponding figures	plate	Z-prime
OV030-200	Fig. 3C, Sup. Fig. 3C	24h	0.8
OV030-200	Fig. 3C, Sup. Fig. 3C	72h	0.75
OV030-70	Fig. 3C, Sup. Fig. 3C	24h	0.54
OV030-70	Fig. 3C, Sup. Fig. 3C	72h	0.66
OV030.A3	Fig. 3C, Sup. Fig. 3C	24h	0.61
OV030.A3	Fig. 3C, Sup. Fig. 3C	72h	0.6
OV030.A2	Fig. 3C, Sup. Fig. 3C	24h	0.85
OV030.A2	Fig. 3C, Sup. Fig. 3C	72h	0.58
OV030-200	Fig.5B-D & Sup. Fig. 5B-C	A133	0.78
OV030-200	Fig.5B-D & Sup. Fig. 5B-C	Carboplatin	0.83
OV030-70	Fig.5B-D & Sup. Fig. 5B-C	A133	0.70
OV030-70	Fig.5B-D & Sup. Fig. 5B-C	Carboplatin	0.69
OV039-PDF	Fig.5B-D & Sup. Fig. 5B-C	A133	NA
OV039-PDF	Fig.5B-D & Sup. Fig. 5B-C	Carboplatin	NA
OV031-PDF	Fig.5B-D & Sup. Fig. 5B-C	A133	NA
OV031-PDF	Fig.5B-D & Sup. Fig. 5B-C	Carboplatin	
Gem061.A	Fig.5B-D & Sup. Fig. 5B-C	A133	0.79
Gem061.A	Fig.5B-D & Sup. Fig. 5B-C	Carboplatin	0.78
OV024.A	Fig.5B-D & Sup. Fig. 5B-C	A133	NA
OV024.A	Fig.5B-D & Sup. Fig. 5B-C	Carboplatin	NA
OVSAHO	Fig.5B-D & Sup. Fig. 5B-C	A133	0.79
OVSAHO	Fig.5B-D & Sup. Fig. 5B-C	Carboplatin	0.79
OVCAR4	Fig.5B-D & Sup. Fig. 5B-C	A133	NA
OVCAR4	Fig.5B-D & Sup. Fig. 5B-C	Carboplatin	NA
KURAMOCHI	Fig.5B-D & Sup. Fig. 5B-C	A133	NA
KURAMOCHI	Fig.5B-D & Sup. Fig. 5B-C	Carboplatin	NA
OV022.T2	Fig.5B-D & Sup. Fig. 5B-C	A133	0.79
OV022.T2	Fig.5B-D & Sup. Fig. 5B-C	Carboplatin	0.77
OV030-200	Fig. 5F,G	DT2216	0.54
OV030-70	Fig. 5F,G	DT2216	0.79
OV039-PDF	Fig. 5F,G	DT2216	0.60
MOLT4	Sup. Fig. 5D	DT2216	0.80

ex vivo cultures

PatientID	day	Z-prime
Gem031	3	0.6623
Gem0243	3	0.3232
Gem0232	3	0.8736
